# Copper-catalyzed synthesis of pyrazolo[1,5-*a*]pyrimidine based triazole-linked glycohybrids: mechanistic insights and bio-applications

**DOI:** 10.1038/s41598-023-50202-4

**Published:** 2024-01-04

**Authors:** Ghanshyam Tiwari, Ashish Khanna, Rajdeep Tyagi, Vinay Kumar Mishra, Chintam Narayana, Ram Sagar

**Affiliations:** 1 Department of Chemistry, Institute of Science, Banaras Hindu University, Varanasi, 221005 India; 2 Glycochemistry Laboratory, School of Physical Sciences, Jawaharlal Nehru University, New Delhi, 110067 India

**Keywords:** Medicinal chemistry, Organic chemistry, Drug discovery, Chemistry

## Abstract

Hybrid molecules maintain their stronghold in the drug market, with over 60% of drug candidates in pharmaceutical industries. The substantial expenses for developing and producing biologically privileged drugs are expected to create opportunities for producing hybrid molecule-based drugs. Therefore, we have developed a simple and efficient copper-catalyzed approach for synthesizing a wide range of triazole-linked glycohybrids derived from pyrazolo[1,5-*a*]pyrimidines. Employing a microwave-assisted copper-catalyzed approach, we developed a concise route using various 7-*O*-propargylated pyrazolo[1,5-*a*]pyrimidines and 1-azidoglycosides. This strategy afforded a series of twenty-seven glycohybrids up to 98% yield with diverse stereochemistry. All were achieved within a remarkably shortened time frame. Our investigation extends to evaluating the anticancer potential of these synthesized triazole-linked pyrazolo[1,5-*a*] pyrimidine-based glycohybrids. In-vitro assays against MCF-7, MDA-MB231, and MDA-MB453 cell lines reveal intriguing findings. (*2R,3S,4S,5R,6R*)-2-(acetoxymethyl)-6-(4-(((5-(4-chlorophenyl)pyrazolo[1,5-*a*]pyrimidin-7-yl)oxy)methyl)-1*H*-1,2,3-triazol-1-yl)tetrahydro-2*H*-pyran-3,4,5-triyl triacetate emerges as a standout with better anticancer activity against MDA-MB231 cells (IC_50_ = 29.1 µM), while (*2R,3R,4S,5R,6R*)-2-(acetoxymethyl)-6-(4-(((5-(4-chlorophenyl)pyrazolo[1,5-*a*]pyrimidin-7-yl)oxy)methyl)-1*H*-1,2,3-triazol-1-yl)tetrahydro-2*H*-pyran-3,4,5-triyl triacetate demonstrates the best inhibitory effects against MCF-7 cells (IC_50_ = 15.3 µM) in all derived compounds. These results align with our docking analysis and structure–activity relationship (SAR) investigations, further validating the in-vitro outcomes. This work not only underscores the synthetic utility of our devised protocol but also highlights the promising potential of these glycohybrids as candidates for further anticancer therapeutic exploration.

## Introduction

**Figure 1:**
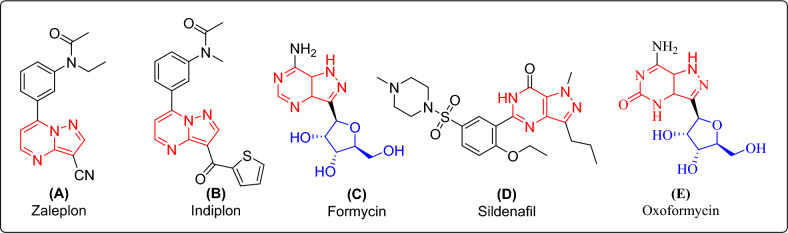
Pyrazolo-pyrimidine derived privileged scaffolds (**A**–**E**): (**A**) Zaleplon, anti-insomnia, (**B**) Indiplon, hypnotic and sedative (**C**) Formycin, antibacterial, (**D**) Sildenafil, used as a treatment of erectile dysfunction, and (**E**) Oxoformycin, antibacterial drug.

Nitrogen-containing heterocycles are abundant in nature, necessary for life, and play a crucial role in the metabolism of all living cells^1–4^. The pyrazolo[1,5-*a*]pyrimidine moiety is one of the several nitrogen-containing heterocycles and is a crucial pharmacophore found in many biologically active molecules^5^. Pyrazolo-pyrimidines and related heterocyclic compounds have a wide range of applications in medicine and agriculture^6,7^. These compounds have been found to exhibit diverse pharmacological activities, and their ability to mimic the structural features of biogenic purines makes them promising candidates for drug development^8^. Additionally, pyrazolo[1,5-*a*]pyrimidines are bioisosteres for compounds such as triazolothienopyrimidines, imidazoquinazolines, pyrimidoquinazolines, and imidazo-quinolinones, all of these have demonstrated good anticancer activity^9^. The literature has recently emphasized the cancer chemopreventive properties of pyrazolo[1,5-*a*]pyrimidine derivatives, which have been found to exhibit apoptosis and differentiation-induced anticancer activities in various in-vitro cell line models. Cancer chemoprevention is mainly aimed to preventing, delaying, or suppressing tumor incidence using synthetic or natural bioactive agents. Encouraging outcomes have been observed, and the derivatives have demonstrated potential in preventing cancer^10,11^. Figure 1 represents the pyrazolopyrimidine core containing privileged scaffolds (A–E), which shows various biological potentials such as (A) Zaleplon is hypnotic generally used as a treatment of sleeping disorder (Insomnia), (B) Indiplon, hypnotic and sedative, (C) Formycin as antibacterial agent, (D) Sildenafil, used as treatment of erectile disfunction (E) Oxoformycin, antibacterial drug^12–17^.

On the other hand, carbohydrates are a type of molecule with diverse stereochemistry and play essential roles in molecular recognition and various intracellular functions^18–22^. When combined with bioactive molecules, carbohydrates can be used to create chemical libraries for drug discovery and development^23–27^. To improve the bioavailability of carbohydrate-derived drugs, the hydroxyl group in the molecule is often masked with a hydrophobic acyl group, which is later cleaved in the blood to create a pro-drug that can be more easily absorbed. In this particular scenario, the notion of hybrid drug design is in its early stages and shows promise, as it enables the utilization of current anticancer agents to create combinations that feature two or more pharmacophores^28^. These combinations can target multiple distinct sites within the infected tissues.

Thus, the process of adding glycone molecules to bioactive aglycone molecules to produce new glycohybrids is ongoing research^29–31^. The process is widely used in drug development to enhance the pharmacological properties and ADMET parameters of drugs^32^. Examples of drug development include glycosylated paclitaxel^33,34^ and demethylepipodophyllotoxin^35,36^, which have increased water solubility and reduced toxicity. Additionally, glycosylated diphyllin^37^ is a more potent topoisomerase II inhibitor compared to the parent compound, and acyl-protected sugar units in etopophos (tafluposide) have been shown to enhance biological activity compared to etopophos alone^38^. Thus, adding the pharmacophoric moiety (*N*-heterocycles) with the glycone unit has become an effective tool for the medication of rapidly growing cancerous cells^39,40^. We conceived that a fresh combination of such compounds could exhibit greater potency than the original one, making it considerably more effective in inhibiting cancerous growth in the body.

These scaffolds stand out as a privileged heterocycle in drug discovery^41^, exhibiting a plethora of biological activities, with a particular emphasis on its significance in anticancer properties^42^. Nevertheless, further extensive studies are required to unveil potential compounds with target-based therapeutic efficacy. In light of the aforementioned literature, It was planned to synthesize the triazole-linked glycohybrids by using the structural motifs of pyrazolo[1,5-*a*]pyrimidine with acetylated glucose, galactose and mannose through a triazole ring as a linker and evaluate their anticancer activity. The copper(I)-catalyzed 1,2,3-triazole formation was chosen as the linking tool due to its stability, specificity, and biocompatibility^43,44^. The 1,2,3-triazole moiety was considered an ideal bioisosteric replacement for the amide due to its similarity in size, dipolar character, and H-bond acceptor properties, and its high chemical stability further supported its use in this context^45^. Azido glycosides derived from d-glucose, d-galactose and d-mannose were used as diversity expedient to couple with various substituted alkyne-modified pyrazolo[1,5-*a*]pyrimidine. These hybrid molecules offered structural diversity, improved solubility and valuable anticancer potentials.

## Results and discussion

### Chemistry

**Figure 2:**
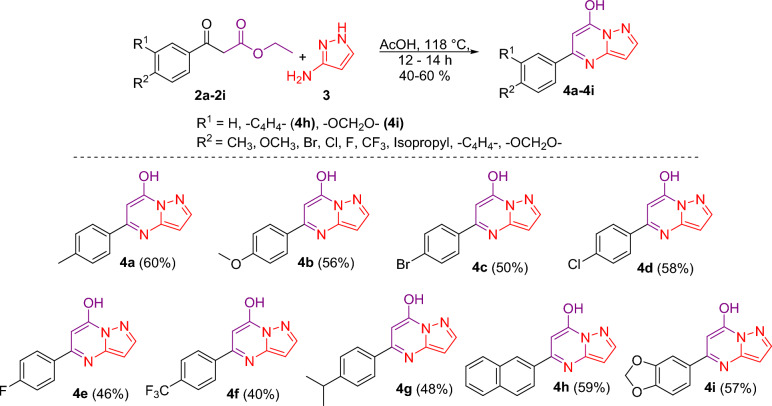
Synthesis of pyrazolo[1,5-*a*]pyrimidin-7-ol **4a**–**4i**. *Reagent and condition:* AcOH, 118 °C, 12–14 h.

**Figure 3:**
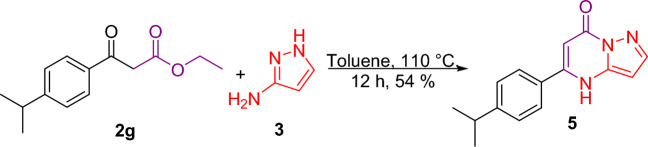
Reaction in the presence of aprotic solvent. *Reagent and condition:* Toluene, 110 °C, 12 h.

For the synthesis of designed molecules as triazole-linked glycohybrids of pyrazolo[1,5-*a*]pyrimidines, it was planned to prepare aglycone and glycone intermediates. Initially, we recognized commercially available diverse acetophenones **1a**–**1i** as a starting material, which can be transformed into diverse β-keto esters **2a**–**2i** (SI, Scheme S1). The β-keto esters **2a**–**2i** were obtained by the esterification of acetophenones **1a**–**1i**, using diethyl carbonate in the presence of a strong base^25,46^. We synthesized β-keto esters and noticed that the product yield varied depending on the various substitutions at the aryl ring. We noticed that upon substitution with electron-withdrawing groups such as fluoro- and trifluoro-methyl at the aryl ring afforded the lower yield of β-keto esters, upon the substitution with electron donating group such as methyl- and methoxy- at the aryl ring favors the process, with a higher yield of β-keto esters. To obtain diverse pyrazolopyrimidin-7-ol, β-keto esters **2a**–**2i** were treated with 3-amino pyrazole **3** under reflux conditions in acetic acid for 12–14 h, which afforded diverse pyrazolopyrimidin-7-ol **4a**–**4i** in good to very good yields (Fig. 2)^47^. Thus, the following variants of pyrazolo[1,5-*a*]pyrimidin-7-ol (**4a**–**4i)** were synthesized, and it was noticed that the product yield varied depending on the various substitutions at the aryl ring. We found that aryl rings with trifluoro methyl and isopropyl substitution had a lower yield. On the other hand, when we used the aprotic solvent toluene instead of the protic solvent acetic acid, we noticed that pyrazolo[1,5-*a*]pyrimidinone **5** was obtained as a primary product (Fig. 3). The proton NMR confirmed that protic solvent is essential for producing pyrazolo[1,5-*a*]pyrimidin-7-ol. ^1^H NMR value of OH in 5-(4-isopropylphenyl)pyrazolo[1,5-*a*]pyrimidin-7-ol (**4g**) appeared at 12.44 ppm has disappeared in 5-(4-isopropylphenyl)pyrazolo[1,5-a]pyrimidin-7(4H)-one (**5**). ^1^H NMR (500 MHz, DMSO-*d*_6_) for δ 8.43 (d, *J* = 8.0 Hz, 2H), 8.38 (s, 1H), 7.96 (d, *J* = 8.0 Hz, 2H), 6.72 (d, *J* = 2.7 Hz, 1H), 6.60–6.57 (m, 1H), 3.54 (dt, *J* = 14.4, 6.8 Hz, 1H), 1.83 (d, *J* = 6.9 Hz, 6H).

**Figure 4:**
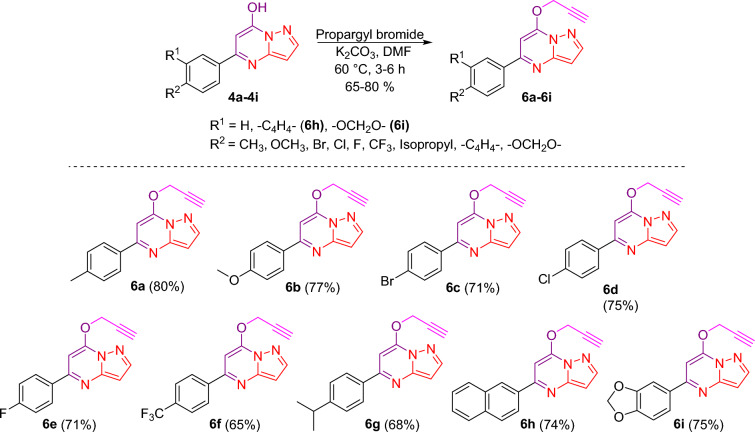
Propargylation of pyrazolo[1,5-*a*]pyrimidin-7-ol derivatives. *Reagent and condition:* Propargyl bromide, K_2_CO_3_, DMF, 60 °C, 3–6 h. In these cases, *N*-propargylated products were also formed as minor products, but yields are reported here for the required ones.

After successfully synthesizing diverse pyrazolopyrimidin-7-ol **4a**–**4i**, it was planned to synthesize alkyne-modified pyrazolopyrimidine-7-ol. Diversely substituted pyrazolopyrimidin-7-ol **4a**–**4i** were treated with propargyl bromide in DMF using K_2_CO_3_ as a base; at elevated temperatures, it furnishes 7-*O-*propargyl pyrazolo[1,5-*a*]pyrimidines **6a**–**6i** (Fig. 4) as a significant product. It is worth mentioning here that minor products were formed under these reaction conditions, which were *N*-propargylated derivatives.

After preparation of aglycone intermediate by performing propargylation of pyrazolo[1,5-*a*]pyrimidines, our focus was drawn on the synthesis of glycone intermediate, which were different azido glycosides of glucose, galactose, and mannose, respectively. The commercially available glucose, galactose, and mannose were acetylated to synthesize these azido glycosides to afford pentaacetylated products **7a**, **7b**, and **7c**, respectively. Further, these pentaacetylated products **7a**, **7b**, and **7c** were treated with trimethylsilyl azide in the presence of SnCl_4_ in dichloromethane at room temperature furnished selective β-1-azido derivatives **8a**, **8b**, and α-1-azido derivatives **8c** in good to excellent yield (SI, Scheme S2)^24^.

**Figure 5:**
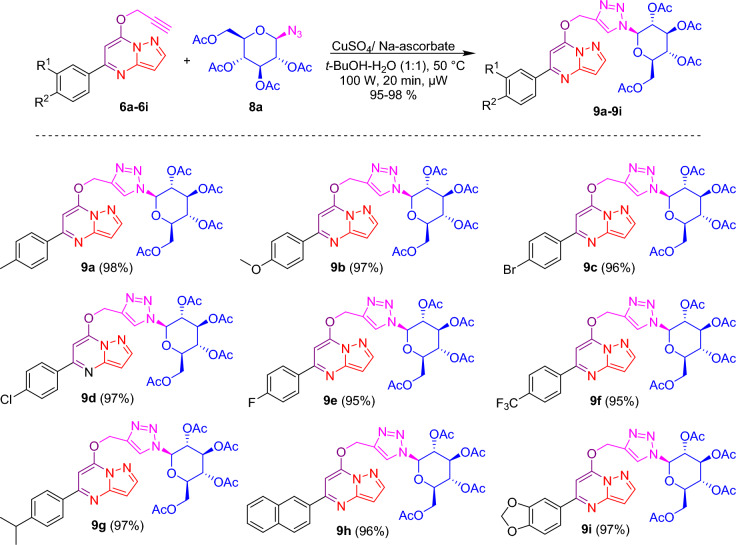
Synthesis of triazole-linked glucohybrids of pyrazolo[1,5-*a*]pyrimidines **9a**–**9i**. *Reagents and conditions:* CuSO_4_/Na-ascorbate, *t*-BuOH-H_2_O (1:1), 50 °C, 100 W, µW, 20 min.

After the successful synthesis and complete characterization of glycone and aglycone parts, it was decided to synthesize designed triazole-linked pyrazolo[1,5-*a*]pyrimidine-based glycohybrids. Herein, we have used the Cu(I) catalyzed 1,3 dipolar cycloaddition reaction (click condition) with our synthesized glycone and aglycone intermediates to transform our designed triazole-linked pyrazolo[1,5-*a*]pyrimidine-based glycohybrids. Initially, the click reaction was carried out between 7-*O*-propargylated pyrazolo[1,5-*a*]pyrimidine **6a** and azido glycoside **8a** adopting standard method using CuSO_4_·5H_2_O and sodium ascorbate in *t*-BuOH–H_2_O (1:1, v/v) at 50 °C afforded glycohybrid **9a** in 80% isolated yield. This yield was not outstanding from the perspective of click chemistry, so to get a good to excellent yield in a shorter time, we chose a new synthetic strategy, in which we carried out the subsequent reactions utilizing the microwave irradiation technique. Here, the reaction was carried out between 7-*O*-propargylated pyrazolo[1,5-*a*]pyrimidine **6a** and azido glucoside **8a** using CuSO_4_·5H_2_O and sodium ascorbate in *t*-BuOH–H_2_O (1:1, v/v), at 50 °C and 100 W for 20 min, we obtained desired glycohybrid product **9a** in excellent yield (98%). By using microwave irradiation and adopting similar reaction conditions, the diverse examples of glucohybrids of pyrazolo[1,5-*a*]pyrimidines **9b**–**9i** have been prepared in good to excellent yields (Fig. 5). To explore the advantages of microwave heating, the reaction was also performed in an oil bath at heating conditions. Still, it was a more extensive, time-consuming reaction (6 h) than the microwave conditions (20 min). Moreover, the yield obtained with conventional heating was lower, reaching 80% compared to the microwave irradiation of 98%. So, it is evident that the microwave approach offers several advantages, such as high yields, mild reaction conditions, short reaction time, and good tolerance of functional groups.

**Figure 6:**
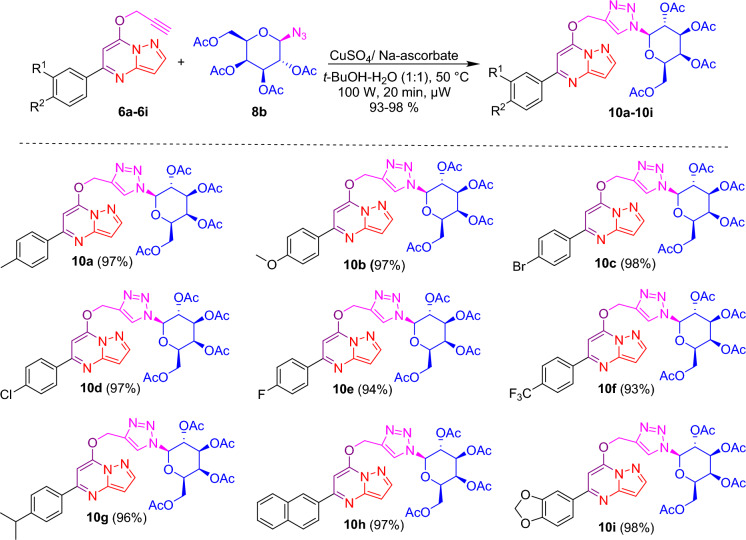
Synthesis of triazole linked galactohybrids of pyrazolo[1,5-*a*]pyrimidines **10a**–**10i**. *Reagents and conditions:* CuSO_4_/Na-ascorbate, *t*-BuOH-H_2_O (1:1), 50 °C, 100 W, µW, 20 min.

Applying a similar synthetic protocol using microwave irradiation conditions, we were also interested in synthesizing galactohybrids, combining propargylated pyrazolo[1,5-*a*]pyrimidines **6a**–**6i** and 1-azido galactoside **8b**. Good to excellent isolated yields of galactohybrids **10a**–**10i** were obtained when propargylated pyrazolo[1,5-*a*]pyrimidines **6a**–**6i** reacted with 1-azido galactoside **8b** in the presence of CuSO_4_·5H_2_O and sodium ascorbate in *t*-BuOH-H_2_O (1:1, v/v) using microwave irradiation under optimized reaction conditions (Fig. 6).

**Figure 7:**
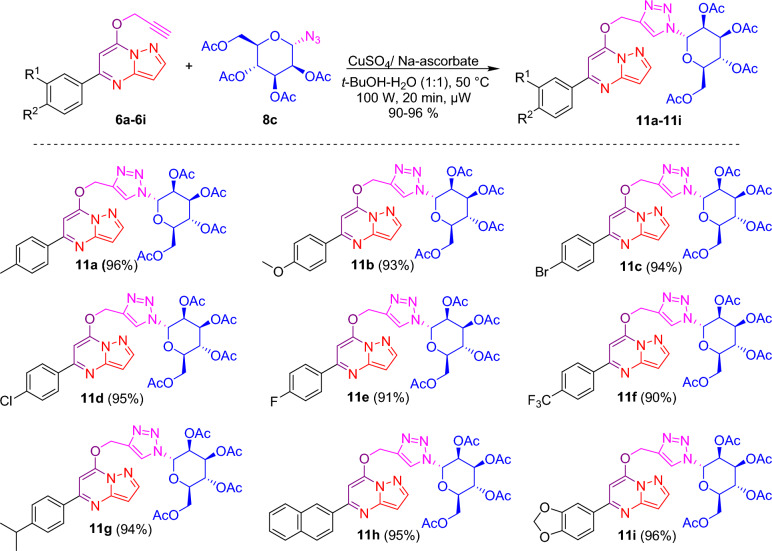
Synthesis of triazole linked mannohybrids of pyrazolo[1,5-*a*]pyrimidines **11a**–**11i**. *Reagents and conditions:* CuSO_4_/Na-ascorbate, *t*-BuOH-H_2_O (1:1), 50 °C, 100 W, µW, 20 min.

We were also interested in investigating the biological potential of mannohybrids. Thus, we have synthesized mannohybrids, integrating propargylated pyrazolo[1,5-*a*]pyrimidines **6a**–**6i** and 1-azido mannoside **8c**, using a similar synthetic approach under microwave reaction conditions. When propargylated pyrazolo[1,5-*a*]pyrimidines **6a**–**6i** interacted with 1-azido mannoside **8c** in the presence of CuSO_4_·5H_2_O and sodium ascorbate in *t*-BuOH–H_2_O (1:1, v/v) under microwave irradiation conditions, good to excellent isolated yields of mannohybrids **11a**–**11i** were achieved (Fig. 7).

### Anticancer activity

Several previous reports showed that the nucleus of pyrazolo[1,5-*a*]pyrimidine may serve as a potent anticancer activity. Thus, we screened the synthesized library of pyrazolo[1,5-*a*]pyrimidin-7-ols **4a**–**4i**. Anticancer activity (in terms of growth inhibition/decreased cell viability with respect to control) of all synthesized compounds (n = 9) was investigated by MTT assay in MDA-MB-231 (human breast cancer) cell line at different concentrations for 72 h. Anticancer drugs YM155 and menadione were used as positive controls, and activity is summarized in Table 1.

**Table 1:** Anticancer activity results.

Compounds	Cell Viability (%) ± SD		
	50 µM	25 µM	10 µM
4a	84.37 ± 2.16	85.49 ± 3.74	91.40 ± 4.38
4b	70.46 ± 5.69	80.07 ± 3.51	85.14 ± 8.53
4c	96.54 ± 2.89	99.13 ± 3.93	100.25 ± 4.01
4d	87.22 ± 9.22	95.24 ± 1.96	98.58 ± 2.25
4e	69.46 ± 5.69	78.07 ± 3.51	86.61 ± 9.76
4f	72.75 ± 7.02	88.80 ± 8.75	92.24 ± 8.51
4g	70.46 ± 5.69	98.07 ± 2.51	101.13 ± 5.51
4h	84.43 ± 7.36	86.48 ± 2.58	89.10 ± 4.09
4i	73.46 ± 6.69	89.07 ± 3.51	96.58 ± 3.36
Menadione (20 µM)	21.76 ± 4.70		
YM155 (20 nM)	20.39 ± 5.13		

The results summarized in Table 1, showed that among the library of pyrazolo[1,5-*a*]pyrimidin-7-ols, none of these compounds did have any growth inhibitory activity against MDA-MB 231 (human breast cancer). Thus, it was aimed to investigate the anticancer activity of a synthesized library of triazole-linked glycohybrids of pyrazolo[1,5-*a*]pyrimidines. Hence, after synthesizing the library of triazole-linked glycohybrids of pyrazolo[1,5-*a*]pyrimidines, they were screened for their anticancer activity using different cell lines. The MTT assay was used to investigate the anticancer activity of twenty-seven synthesized compounds on the growth inhibition and decrement in cell viability of MDA-MB-231 (human breast cancer). Different concentrations were tested for 72 h, and the results were compared to a control. Positive controls, namely YM155 and menadione, were also utilized, and the findings are presented in Table 2.

**Table 2:** Anticancer activity results.

Compounds	Cell viability (%) ± SD			Compounds	Cell viability (%) ± SD		
	50 µM	25 µM	10 µM		50 µM	25 µM	10 µM
9a	**35**.**32 **±** 4**.**29**	**51**.**03 **±** 9**.**19**	70.14 ± 9.53	**10f**	**41**.**20 **±** 1**.**26**	**45**.**10 **±** 3**.**32**	69.37 ± 5.36
9b	83.37 ± 4.16	85.49 ± 6.74	95.40 ± 5.38	10g	78.74 ± 2.56	79.51 ± 8.96	85.23 ± 3.50
9c	99.54 ± 2.89	100.13 ± 8.93	100.25 ± 8.01	10h	57.70 ± 10.82	89.59 ± 9.91	75.04 ± 8.31
9d	**58**.**56 **±** 5**.**61**	**60**.**98 **±** 8**.**47**	75.61 ± 9.76	**10i**	98.50 ± 1.88	99.25 ± 0.34	100.28 ± 9.67
9e	81.22 ± 9.22	92.24 ± 1.96	98.58 ± 2.25	**11a**	96.74 ± 9.12	91.81 ± 7.09	98.05 ± 7.70
9f	73.75 ± 7.02	88.80 ± 8.75	92.24 ± 8.51	**11b**	70.36 ± 10.49	95.16 ± 10.62	99.50 ± 10.20
9g	68.46 ± 17.69	103.07 ± 3.51	110.13 ± 13.51	**11c**	72.55 ± 8.45	76.52 ± 6.58	81.32 ± 7.65
9h	**42**.**82 **±** 5**.**04**	**55**.**17 **±** 7**.**06**	96.58 ± 3.36	**11d**	88.98 ± 1.10	92.06 ± 6.00	95.84 ± 5.85
9i	80.43 ± 7.36	82.48 ± 2.58	85.10 ± 4.09	**11e**	88.57 ± 6.27	90.13 ± 6.77	98.41 ± 3.62
10a	68.81 ± 10.40	78.47 ± 6.73	81.68 ± 8.36	**11f**	86.54 ± 7.27	74.49 ± 4.44	79.63 ± 4.70
10b	70.48 ± 6.24	85.20 ± 8.63	92.51 ± 9.39	**11g**	**45**.**28 **±** 4**.**11**	**54**.**28 **±** 6**.**12**	66.70 ± 8.21
10c	74.04 ± 2.62	77.76 ± 2.43	79.46 ± 2.43	**11h**	82.53 ± 5.45	89.82 ± 2.22	100.77 ± 9.28
10d	**40**.**98 **±** 4**.**91**	**53**.**28 **±** 6**.**32**	56.70 ± 8.23	**11i**	93.89 ± 9.08	95.31 ± 3.60	97.45 ± 4.91
10e	71.33 ± 6.17	68.62 ± 10.05	78.40 ± 9.97	**YM155 (20 nM)**	26.39 ± 3.93		
Menadione (20 µM)	20.77 ± 6.70						

Significant values are in [bold].

**Figure 8:**
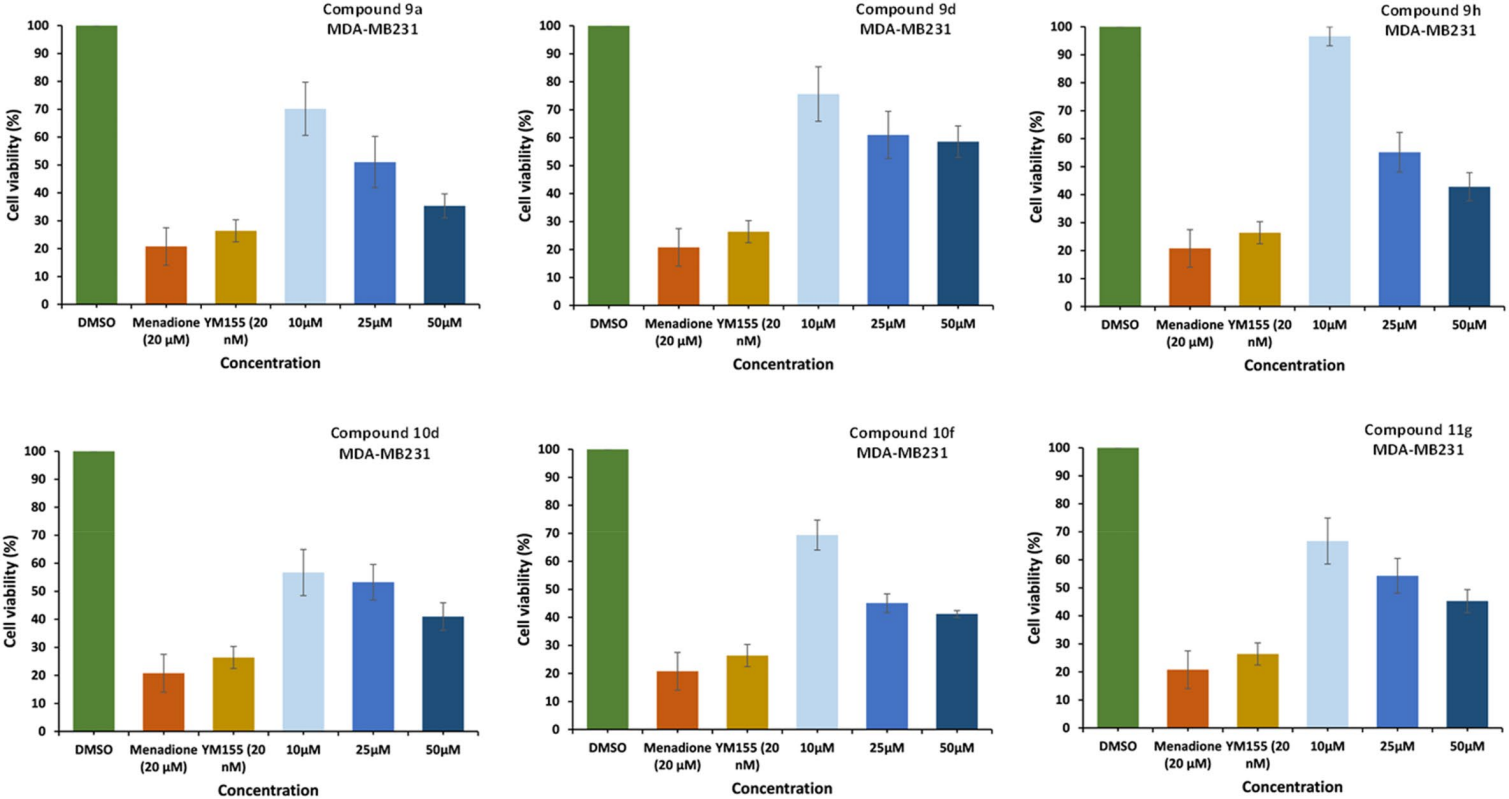
Anticancer activities at different dilutions for active compounds were found at 10 µM, 25 µM, and 50 µM for compounds **9a**, **9d**, **9h**, **10d**, **10f**, and **11g** respectively, compared to DMSO control.

The results summarized in Table 2 showed moderate growth inhibition in all tested compounds with better activity with compounds **9a**, **9d**, **9h**, **10d**, **10f** and **11g** in MDA-MB-231 breast cancer cells. These compounds show almost 50% inhibition at 25 μM concentration. These six compounds were further screened, and results were taken to identify IC_50_ (Fig. 8).

**Figure 9:**
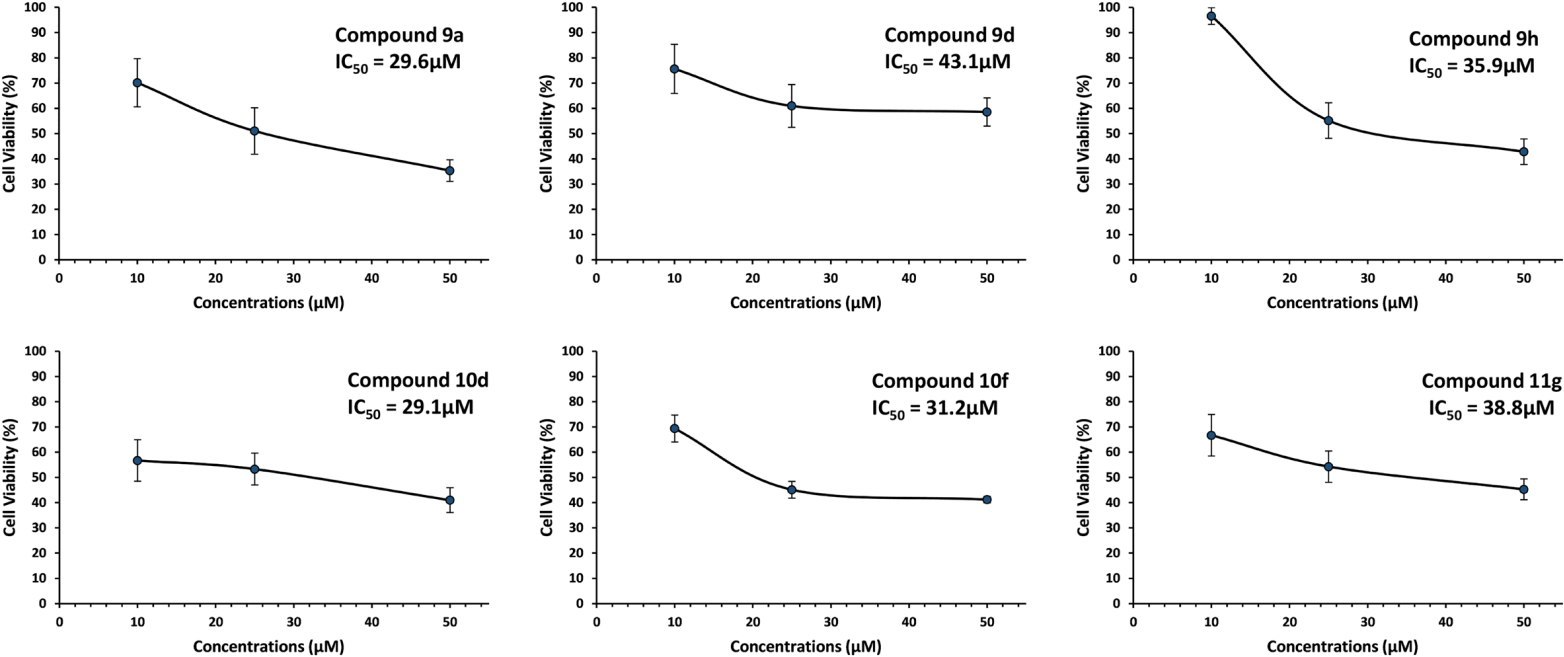
IC_50_ of the most active compounds. To calculate half maximal inhibitory concentration (IC_50_) of compounds **9a**, **9d**, **9h**, **10d**, **10f**, and **11g** MDA-MB-231 cells were treated for 72 h with different concentrations (10, 25 and 50 µM) of the respective drugs and tested for cell viability by the MTT assay. IC_50_ values were determined by plotting values of percent cell viability against the concentration of each of these compounds. IC_50_ values for compounds **9a**, **9d**, **9h**, **10d**, **10f** and **11g** against MDA-MB-231 breast cancer cell line were found 29.6 µM, 43.1 µM, 35.9 µM, 29.1 µM, 31.2 µM and 38.8 µM respectively. The experiments were performed in triplicates, n = 3 and ± SD value was calculated for each data point.

MDA-MB-231 represents a specific subtype known as triple-negative breast cancer (TNBC). The investigation study was extended with active six hit compounds (**9a**, **9d**, **9h**, **10d**, **10f**, and **11g**) in two other different cancer cell lines (MCF-7 and MDA-MB-453), each representing a separate class of breast cancers (MCF-7: hormone receptor/HR-positive; MDA-MB-453: human epidermal growth factor 2 receptor/HER2 positive). The IC_50_ values for the most active compounds **9a**, **9d**, **9h**, **10d**, **10f**, and **11g** were calculated and presented in Fig. 9 against MDA-MB-231 breast cancer cell line were found 29.6 µM, 43.1 µM, 35.9 µM, 29.1 µM, 31.2 µM and 38.8 µM, respectively.

We also included normal mammary epithelial MCF-10A cells in our experiments to confirm if the growth-inhibitory activities of the selected compounds are truly cancer cell-specific. The activity data from MCF-7 and MDA-MB-453 cell lines (tested at 1 µM, 10 µM and 25 µM) and activity data from MCF-10A cell line (tested at 25 µM, 50 µM and 100 µM) are summarized in Table 3.

**Table 3:** Anticancer activity screening results.

Compounds	Cell viability (%) ± SD								
	MCF-7			MDA-MB-453			MCF-10A		
	25 µM	10 µM	1 µM	25 µM	10 µM	1 µM	25 µM	50 µM	100 µM
9a	**21**.**7 **±** 4**.**0**	41.3 ± 3.6	68.7 ± 1.18	78.1 ± 5.7	86.4 ± 5.4	80.6 ± 8.0	88.6 ± 1.5	84.3 ± 2.9	84.2 ± 3.0
9d	**62**.**8 **±** 3**.**0**	69.8 ± 1.8	100.0 ± 4.5	65.3 ± 5.8	82.2 ± 11.5	80.8 ± 3.2	87.8 ± 2.8	85.6 ± 0.7	82.0 ± 1.5
9h	**42**.**3 **±** 7**.**6**	58.7 ± 2.4	79.2 ± 2.6	66.4 ± 3.2	74.8 ± 1.5	105 ± 8.1	86.8 ± 5.3	88.8 ± 3.0	68.5 ± 3.4
10d	**20**.**0 **±** 2**.**9**	34.0 ± 2.5	66.0 ± 4.7	69.6 ± 5.3	78.3 ± 4.7	88.8 ± 5.2	89.6 ± 2.2	85.6 ± 1.7	84.4 ± 2.6
10f	**21**.**1 **±** 4**.**4**	28.4 ± 0.9	51.3 ± 3.1	66.8 ± 1.7	80.9 ± 1.2	92.5 ± 4.6	86.6 ± 1.5	88.1 ± 2.8	84.2 ± 3.0
11g	**49**.**1 **±** 3**.**5**	68.2 ± 3.8	70.8 ± 4.2	96.1 ± 35.7	76.4 ± 5.4	95.4 ± 4.8	89.8 ± 2.8	84.2 ± 5.8	88.0 ± 1.5
YM155 (20 nM)	32.2 ± 1.7	32.2 ± 1.7	32.2 ± 1.7	35.2 ± 1.5	35.2 ± 1.5	35.2 ± 1.5	38.2 ± 1.6	38.2 ± 1.6	38.2 ± 1.6

Significant values are in [bold].

**Figure 10:**
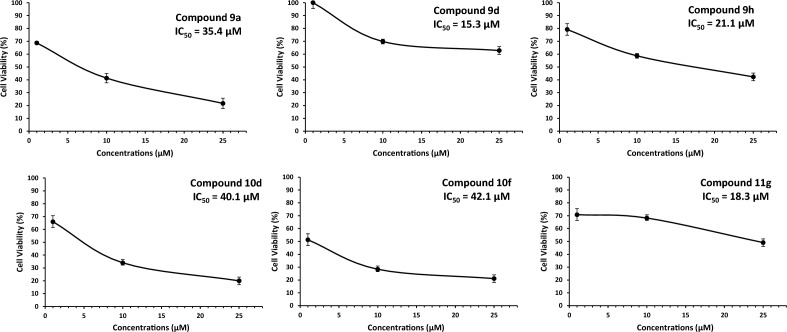
IC_50_ of the most active compounds. To calculate half maximal inhibitory concentration (IC_50_) of compounds **9a**, **9d**, **9h**, **10d**, **10f** and **11g**, MCF-7 cells were treated for 72 h with different concentrations (10 µM, 25 µM and 50 μM) of the respective drugs and tested for cell viability by the MTT assay. The IC_50_ values for compounds **9a**, **9d**, **9h**, **10d**, **10f** and **11g** against MCF-7 breast cancer cells line were found 35.4 µM, 15.3 µM, 21.1 µM, 40.1 µM, 42.1 µM, and 18.3 μM, respectively. While these compounds did not reach up to IC_50_ value against MDA-MB-453 cells. Furthermore, none of these compounds did have any growth inhibitory activity against normal breast epithelial cell (MCF-10A).

The results summarized in Table 3 showed better inhibition of cell viability was observed with compounds **9a**, **9d**, **9h**, **10d**, **10f** and **11g** at 25 µM in MCF-7 (hormone receptor/HR-positive breast cancer cells). These compounds show almost 50% inhibition at 25 μM concentration. The IC_50_ values of the most active compounds are calculated and presented in Fig. 10.

Thus, through screening against various cell lines such as MDA-MB231, MCF-7, MDA-MB453 and MCF-10A, it has been found that among the derived library of compounds, most of the compounds show moderate to good anticancer activity. While compound **10d** shows the best inhibitory activity against MDA-MB231 cell line with an IC_50_ value at 29.1 µM, and compound **9d** shows the best inhibitory activity against the MCF-7 cell line with an IC_50_ value of 15.3 µM.

### Molecular docking and SAR studies

**Figure 11:**
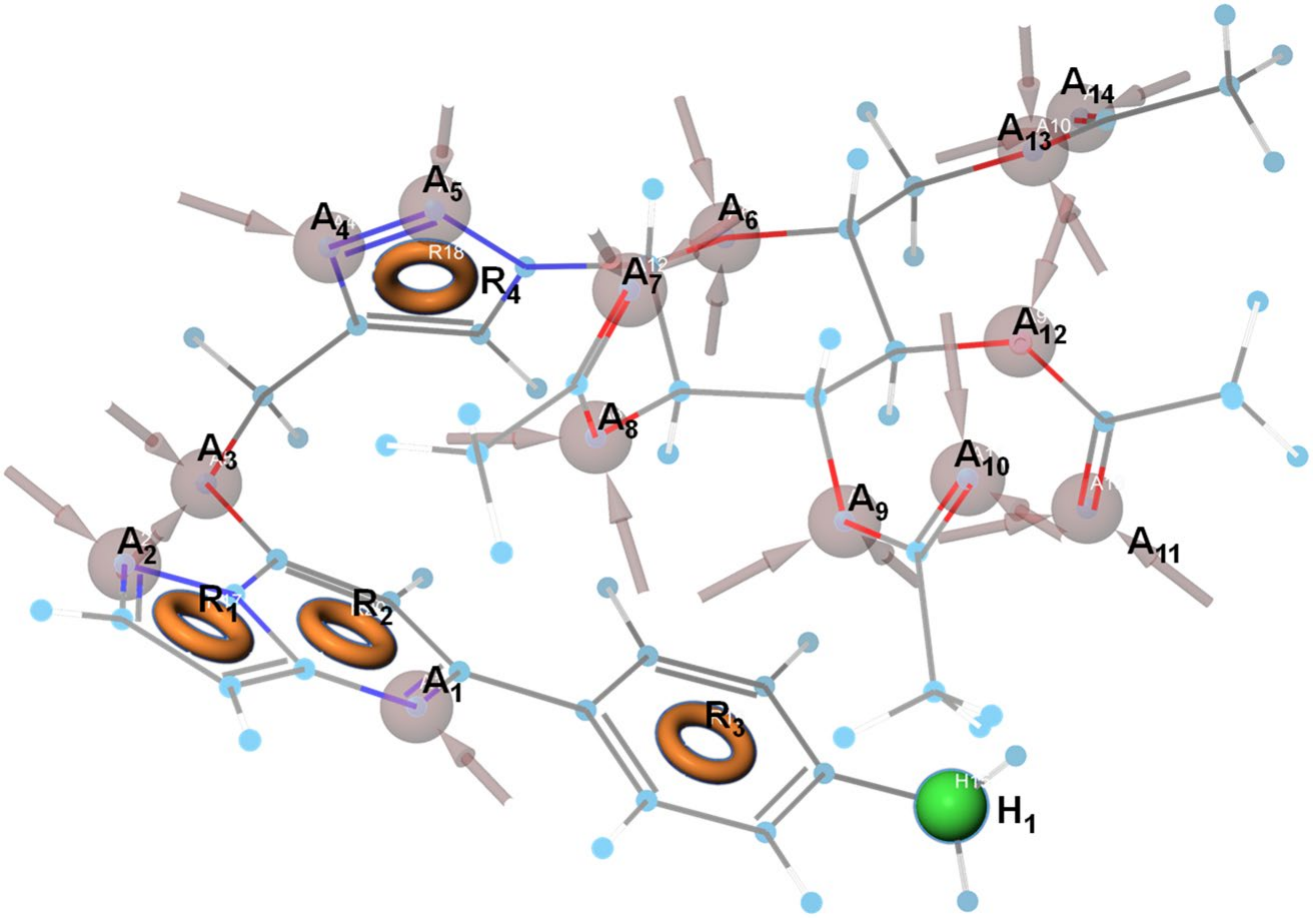
3D-Pharmacophore model showing different constitutional molecular descriptors triazole linked hybrids of pyrazolo[1,5-*a*]pyrimidines. The grey circle with the arrow represents the hydrogen acceptor sites (A_1_–A_14_), the brown circle represents various aromatic rings (R_1_–R_3_), green circle represents the hydrophobic (H_1_) site.

Docking plays a crucial role in drug design by analyzing the binding interactions between a protein and a ligand. As a result, it necessitates the use of 3-D structures for both the ligand and protein. This is important in identifying potential targets for developing substrate and cofactor-based inhibitors, which may be effective as anticancer and antiproliferative drugs. To this end, we utilized the Schrödinger maestro tool to perform SAR studies and develop a ligand-based pharmacophore model hypothesis for the determination of ligand potencies and pharmacological properties. Our analysis revealed that the designed molecule exhibits diverse constitutional molecular descriptors, including aromatic rings (R_1_–R_3_), hydrogen bond acceptor sites (A_1_–A_14_), and hydrophobic site (H_1_). By substituting various electron-donating or withdrawing groups (H_1_) on the R_3_ aromatic ring, we achieved a range of ligand potency and intrinsic activity. The aromatic rings (R_1_–R_3_) aided in successful binding to the hydrophobic pocket of the target protein, as shown in Fig. 11.

In several previous reports, it has been reported that heterocycle containing pyrazolo[1,5-*a*]pyrimidine nucleus displays crucial drug-like properties such as anticancer, anti-neoplastic, antiproliferative, and many more. These molecules were found to have potential anticancer activity against MCF-7, which are HER2 positive cell lines. Thus, to identify the anticancer properties of pyrazolo[1,5-*a*]pyrimidine nucleus, we performed molecular docking between the designed compounds with the Human HER2 protein of tyrosine kinase domain (Protein Data Bank ID: **3PP0**). The HER2 (Human epidermal growth factor receptor-2) protein of the tyrosine kinase domain is a membrane oncogene and serves as a major driver for the tumor development and proliferation of breast cancer. Thus, HER2 protein has always served as a desirable target site for researchers to evaluate the potency of most anticancer and anti-proliferative drugs. Most of the anticancer drugs inhibit the kinase activity of HER2 protein, suppressing the proliferation of cancerous cells.

**Figure 12:**
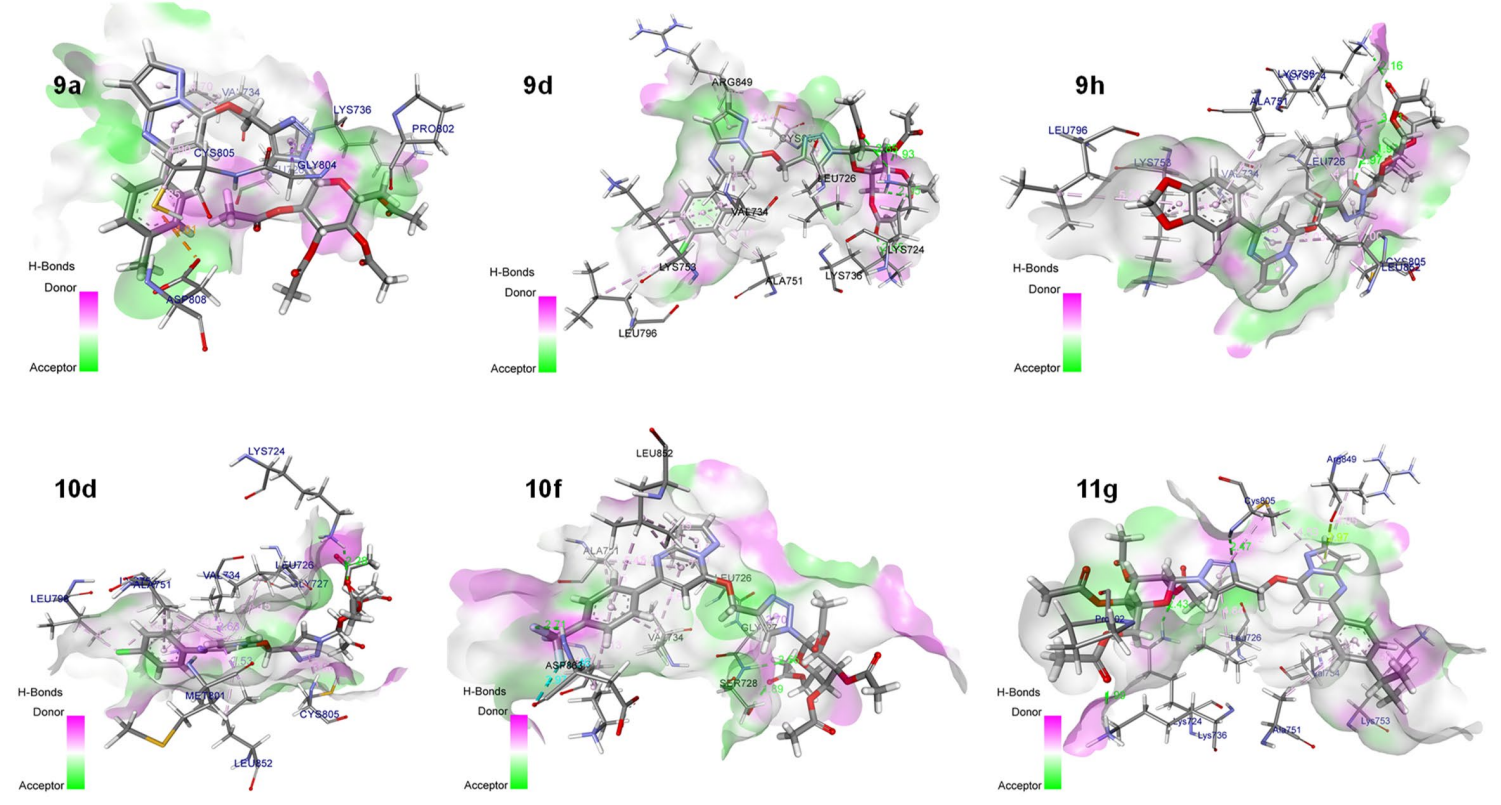
Docking analysis of triazole-linked pyrazolo[1,5-*a*] pyrimidine-based glycohybrids (Compounds **9a**, **9d**, **9h**, **10d**, **10f** and **11g**) and catalytic site of HER2 protein with a 3D representation of hydrogen bond donor/acceptor surface (shown in pink and green color) and hydrogen bond (green color). The docking analysis was conducted using the collected set of compounds (gray) into the proposed binding pocket of the X-ray crystallographic structure of HER2 protein (Protein Data Bank ID: **3PP0**, resolution: 2.4 Å).

Active site residues of the protein receptors have been identified as LEU785, LYS753, LEU852, GLY804, LEU800, MET801, ALA751, THR862, ASP863, THR798, VAL734, SER783, and PHE864. The parameters of the molecule in the active region were determined with the following values: grid box sizes of 34, 25, and 32 Å^3^, and x, y, z centers of 17.882, 15.918, and 28.054, respectively. The docking study reveals that the interaction between synthesized triazole-linked pyrazolo[1,5-*a*]pyrimidine-based glycohybrids and catalytic site HER2 protein occurs primarily through hydrogen bonds, hydrophobic bonds and π-stacking (as shown in Fig. 12).

**Figure 13:**
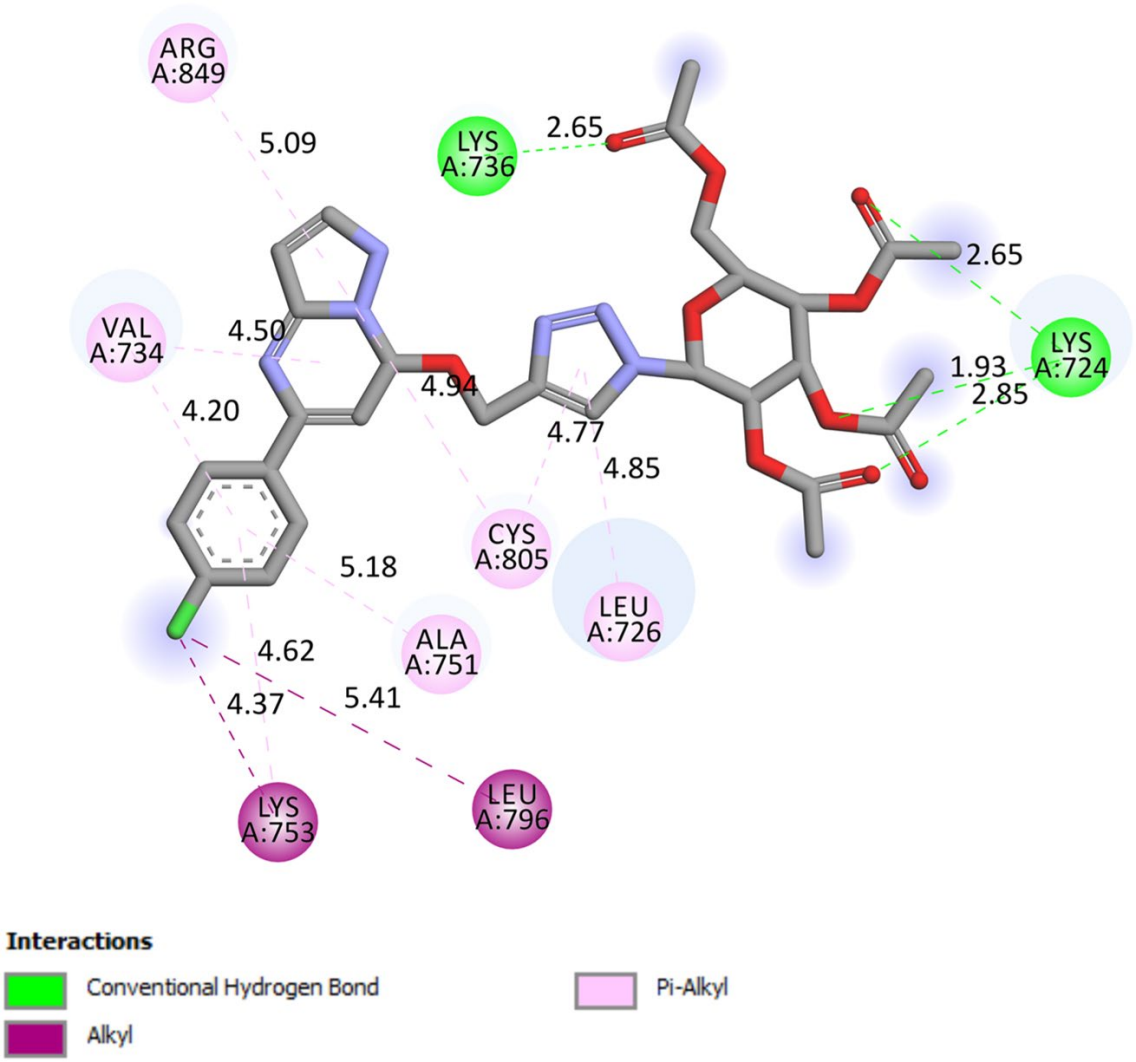
2D representation of docked results of compound **9d**.

Based on the docking results, the best docking poses, docking score with high binding affinity/energies and a number of favorable interactions suggest that most of the designed triazole-linked pyrazolo[1,5-*a*]pyrimidine-based glycohybrids may serve as effective anticancer candidates. Among the derived library of compounds, compound **9d** displays the best docking pose with binding energy – 42.73 kcal/mol in mode 1 with minimum root mean square deviation value and perfectly fits into the active binding pocket of the protein. In Fig. 13 shows four conventional hydrogen bonds between the LYS736, and LYS724 active residues, respectively. Along with this, π-alkyl interactions have been found between the CYS805 and LEU726 active residues and the triazole ring and heterocyclic ring of the designed molecule. Also, other interactions, such as alkyl C–H interactions, have also been found. These hydrophobic and hydrophilic interactions displayed the binding versatility of the designed molecules, and the results obtained from in-silico docking suggest that most of the molecules perfectly fit into the binding pocket of the docked protein and may possess anticancer activity.

These results obtained from in-silico studies further validate the biological potential of our designed molecules as effective anticancer agents and support the results obtained from in-vitro studies.

## Conclusions

In this investigation, we have designed and developed a series of pyrazolo[1,5-*a*] pyrimidine-based triazole-linked glycohybrids. The process involved microwave-assisted synthesis adopting copper-catalyzed click reaction, in which newly synthesized different 7-*O*-propargylated pyrazolo[1,5-*a*]pyrimidines were developed in excellent yields. This 7-*O*-propargylated pyrazolo[1,5-*a*]pyrimidines reacted with 1-azido-2,3,4,6-tetra-*O*-acetyl- d-glucose, d-galactose and d-mannose respectively under click reaction conditions they afforded diverse library of glycohybrids. Herein, we have synthesized a series of twenty-seven diverse substituted glycohybrids containing electron-donating and electron-withdrawing groups with inherited stereochemical diversity in a shorter reaction time. In this investigation, we have also evaluated the anticancer potential of our newly synthesized pyrazolo[1,5-*a*]pyrimidine-based triazole-linked glycohybrids. Anticancer activity was performed in-vitro against MCF-7, MDA-MB231, and MDA-MB453 cell-lines in cell-based assays. It was found that among the derived library of compounds, **10d** shows better anticancer activity with IC_50_ value of 29.1 µM against MDA-MB231 cell line and **9d** shows the best inhibitory activity against MCF-7 cell line with IC_50_ value of 15.3 µM. The in-silico docking analysis and SAR studies well supported the outcomes obtained from in-vitro study.

## Experimental

### General experimental methods

All experiments were conducted using anhydrous solvents and oven-dried glassware and were carried out using a CEM microwave synthesizer. High-resolution mass spectra were recorded using an ESI source and a quadrupole/TOF mass spectrometer. Solvents were distilled using standard methods and stored in 4Å and 3Å molecular sieves. JEOL JNM-ECZ500R/S1 instrument was used to record ^1^H (500 MHz) and ^13^C (126 MHz) spectra. The chemical shifts for ^1^H and ^13^C were referenced to the residual signals of CDCl_3_^1^H NMR δ 7.26 and δ 77.16 for ^13^C NMR, and DMSO-d_6_^1^H NMR δ 2.5 and δ 39.52 for ^13^C NMR, reported in parts per million (ppm) at 25 °C. Coupling constants were expressed in hertz (Hz). Thin-layer chromatography was used to monitor reactions, carried out on 0.25 mm Merck silica gel plates (60F-254), with spots visualized using phosphomolybdic acid and 10% H_2_SO_4_ in ethanol. Reagents were purchased from TCI, Merck, Sigma Aldrich, and other sources.

### Synthesis of triazole-linked glucohybrids of pyrazolo[1,5-***a***]pyrimidines 9a–9i

In a microwave vial, a mixture of 50 mg (0.189 mmol) of *O*-propargylated pyrazolo[1,5-*a*]pyrimidine **6a** and 70.89 mg (0.189 mmol) of 1-azido glucoside **8a** in 2 ml of solvent (1:1, v/v mixture of *t*-BuOH-H_2_O) was treated with CuSO_4_.5H_2_O (1.24 mg, 0.0056 mmol) and sodium ascorbate (2.23 mg, 0.011 mmol), and then subjected to microwave heating for 20 min at 50 °C (100 W). The reaction was monitored by TLC to confirm completion, and upon completion, the reaction mixture was extracted with 5 ml of water and 3 ml of EtOAc. The organic layer was then dried over Na_2_SO_4_ and evaporated via a rotary evaporator to obtain the crude residue, which was purified by flash column chromatography to yield pure compound **9a** in 98% isolated yield. Using similar methods, with 50 mg of propargylated reactants compounds **9b**–**9i** were synthesized in good to excellent yields utilizing 1-azido glucoside.

***(2R,3R,4S,5R,6R)-2-(acetoxymethyl)-6-(4-(((5-(p-tolyl)pyrazolo[1,5-a]pyrimidin-7-yl)oxy)methyl)-1H-1,2,3-triazol-1-yl)tetrahydro-2H-pyran-3,4,5-triyl triacetate* (9a):** yellow colored sticky solid; yield 118.39 mg (98%), Rf = 0.32 (EtOAc); ^1^H NMR (500 MHz, CDCl_3_) δ 8.01 (s, 1H), 7.86 (d, *J* = 3.3 Hz, 1H), 7.82 (d, *J* = 7.9 Hz, 2H), 7.21 (d, *J* = 7.3 Hz, 2H), 6.50 (s, 1H), 6.39 (d, *J* = 3.8 Hz, 1H), 6.10 (d, *J* = 15.7 Hz, 1H), 5.99 (d, *J* = 15.6 Hz, 1H), 5.79 (d, *J* = 8.0 Hz, 1H), 5.36 (t, *J* = 8.0 Hz, 1H), 5.35–5.32 (m, 1H), 5.21 (t, *J* = 9.3 Hz, 1H), 4.23 (dd, *J* = 12.7, 5.5 Hz, 1H), 4.09 (dd, *J* = 11.3 Hz, 3.5 Hz, 1H), 3.97–3.94 (m, 1H), 2.35 (s, 3H), 2.01 (s, 3H), 2.00 (s, 3H), 1.96 (s, 3H), 1.62 (s, 3H). ^13^C NMR (125 MHz, CDCl_3_) δ 170.5, 169.9, 169.3, 168.4, 162.2, 158.3, 153.9, 141.5, 140.2, 139.9, 134.9, 129.4, 127.1, 123.4, 100.2, 98.6, 85.8, 75.2, 72.4, 70.3, 67.6, 61.4, 47.0, 21.3, 20.6, 20.5, 19.8. HRMS (ESI-TOF), m/z calcd. C_30_H_33_N_6_O_10_ [M + H]^+^ 637.2253; Found: 637.2228.

***(2R,3R,4S,5R,6R)-2-(acetoxymethyl)-6-(4-(((5-(4-methoxyphenyl)pyrazolo[1,5-a]pyrimidin-7-yl)oxy)methyl)-1H-1,2,3-triazol-1-yl)tetrahydro-2H-pyran-3,4,5-triyl triacetate* (9b):** red colored sticky solid; yield: 113.32 mg (97%), Rf = 0.33 (EtOAc); ^1^H NMR (500 MHz, CDCl_3_) δ 7.98 (s, 1H), 7.92 (d, *J* = 8.0 Hz, 2H), 7.83 (d, *J* = 4.0 Hz, 1H), 6.95 (d, *J* = 8.0 Hz, 2H), 6.48 (s, 1H), 6.39 (d, *J* = 4.0 Hz, 1H), 6.10 (d, *J* = 14.7 Hz, 1H), 6.00 (d, *J* = 14.7 Hz, 1H), 5.76 (d, *J* = 9.3 Hz, 1H), 5.36 (t, *J* = 10.0 Hz, 1H), 5.34–5.33 (m, 1H), 5.20 (t, *J* = 9.5 Hz, 1H), 4.25 (dd, *J* = 13.3, 5.5 Hz, 1H), 4.11 (dd, *J* = 12.0 Hz, 3.5 Hz, 1H), 3.96–3.93 (m, 1H), 3.84 (s, 3H), 2.03 (s, 6H), 1.98 (s, 3H), 1.65 (s, 3H). ^13^C NMR (125 MHz, CDCl_3_) δ 170.6, 170.0, 169.3, 168.5, 161.9, 161.4, 158.4, 153.9, 141.6, 139.9, 130.3, 128.8, 123.3, 114.1, 100.3, 98.0, 85.9, 75.3, 72.5, 70.3, 67.7, 61.5, 55.4, 47.1, 20.7, 20.6, 19.9. HRMS (ESI-TOF), m/z calcd. C_30_H_33_N_6_O_11_[M + H]^+^ 653.2202; Found: 653.2179.

***(2R,3R,4S,5R,6R)-2-(acetoxymethyl)-6-(4-(((5-(4-bromophenyl)pyrazolo[1,5-a]pyrimidin-7-yl)oxy)methyl)-1H-1,2,3-triazol-1-yl)tetrahydro-2H-pyran-3,4,5-triyl triacetate* (9c):** light yellow colored sticky solid; yield 99.57 mg (96%), Rf = 0.31 (EtOAc); ^1^H NMR (500 MHz, CDCl_3_) δ 7.99 (s, 1H), 7.87 (d, *J* = 5.3 Hz, 1H), 7.83 (d, *J* = 8.0 Hz, 2H), 7.56 (d, *J* = 8.0 Hz, 2H), 6.50 (s, 1H), 6.42 (d, *J* = 5.3 Hz, 1H), 6.14 (d, *J* = 14.7 Hz, 1H), 6.00 (d, *J* = 14.7 Hz, 1H), 5.77 (d, *J* = 9.3 Hz, 1H), 5.36 (t, *J* = 10.0 Hz, 1H), 5.32 (t, *J* = 10.0 Hz, 1H), 5.20 (t, *J* = 10.0 Hz, 1H), 4.26 (dd, *J* = 13.3, 5.5 Hz, 1H), 4.11 (dd, *J* = 12.0 Hz, 3.5 Hz, 1H), 3.96–3.93 (m, 1H), 2.04 (s, 6H), 1.99 (s, 3H), 1.65 (s, 3H). ^13^C NMR (125 MHz, CDCl_3_) δ 170.5, 169.9, 169.3, 168.4, 161.0, 158.2, 153.9, 141.5, 140.0, 136.7, 131.8, 128.8, 124.6, 123.3, 100.2, 98.8, 85.9, 75.2, 72.4, 70.3, 67.7, 61.5, 47.1, 20.7, 20.5, 19.8. HRMS (ESI-TOF), m/z calcd. C_29_H_30_BrN_6_O_10_ [M + H]^+^ 701.1201; Found: 701.1177.

***(2R,3R,4S,5R,6R)-2-(acetoxymethyl)-6-(4-(((5-(4-chlorophenyl)pyrazolo[1,5-a]pyrimidin-7-yl)oxy)methyl)-1H-1,2,3-triazol-1-yl)tetrahydro-2H-pyran-3,4,5-triyl triacetate* (9d):** off white colored sticky solid; yield 116.42 mg (97%), Rf = 0.31 (EtOAc); ^1^H NMR (500 MHz, CDCl_3_) δ 7.99 (s, 1H), 7.88 (m, 3H), 7.39 (d, *J* = 8.1 Hz, 2H), 6.49 (s, 1H), 6.40 (d, *J* = 4.0 Hz, 1H), 6.13 (d, *J* = 14.8 Hz, 1H), 5.99 (d, *J* = 15.4 Hz, 1H), 5.79 (d, *J* = 8.3 Hz, 1H), 5.37 (t, *J* = 9.5 Hz, 1H), 5.32 (t, *J* = 9.5 Hz, 1H), 5.21 (t, *J* = 9.5 Hz, 1H), 4.25 (dd, *J* = 13.3, 5.0 Hz, 1H), 4.11 (dd, *J* = 12.0 Hz, 3.5 Hz, 1H), 3.97–3.94 (m, 1H), 2.03 (s, 6H), 1.97 (s, 3H), 1.63 (s, 3H). ^13^C NMR (125 MHz, CDCl_3_) δ 170.5, 169.9, 169.3, 168.5, 160.9, 158.2, 153.9, 141.5, 140.0, 136.3, 136.2, 128.9, 128.6, 123.3, 100.2, 98.9, 85.9, 75.3, 72.4, 70.3, 67.7, 61.4, 47.2, 20.7, 20.5, 19.8. HRMS (ESI-TOF), m/z calcd. C_29_H_30_ClN_6_O_10_ [M + H]^+^ 657.1706; Found: 657.1687.

***(2R,3R,4S,5R,6R)-2-(acetoxymethyl)-6-(4-(((5-(4-fluorophenyl)pyrazolo[1,5-a]pyrimidin-7-yl)oxy)methyl)-1H-1,2,3-triazol-1-yl)tetrahydro-2H-pyran-3,4,5-triyl triacetate* (9e):** white colored sticky solid; yield 132.74 mg (95%) Rf = 0.31 (EtOAc); ^1^H NMR (500 MHz, CDCl_3_) δ 8.00 (s, 1H), 7.92 (dd, *J* = 9.0, 5.1 Hz, 2H), 7.87 (d, *J* = 3.9 Hz, 1H), 7.09 (m, 2H), 6.46 (s, 1H), 6.39 (d, *J* = 3.9 Hz, 1H), 6.12 (d, *J* = 14.8 Hz, 1H), 5.99 (d, *J* = 14.8 Hz, 1H), 5.80 (d, *J* = 8.1 Hz, 1H), 5.37 (t, *J* = 9.5 Hz, 1H), 5.32 (t, *J* = 9.0 Hz, 1H), 5.21 (t, *J* = 9.5 Hz, 1H), 4.23 (dd, *J* = 13.3, 5.0 Hz, 1H), 4.11 (dd, *J* = 12.0 Hz, 3.5 Hz, 1H), 3.98–3.95 (m, 1H), 2.02 (s, 3H), 2.01 (s, 3H), 1.96 (s, 3H), 1.62 (s, 3H). ^13^C NMR (125 MHz, CDCl_3_) δ 170.52, 169.9, 169.3, 168.4, 165.0, 163.1, 161.1, 158.2, 153.9, 141.5, 140.0, 134.0, 129.2, 129.2, 123.3, 115.7, 115.5, 100.1, 98.6, 85.8, 75.2, 72.4, 70.3, 67.7, 61.4, 47.1, 20.6, 20.5, 20.5, 19.8. ^13^C–^19^F couplings in ^13^C NMR (125 MHz, DMSO-*d*_6_) δ 164.1 (d, *J*_*C-F*_ = 249.48 Hz, C_1_), 129.2 (d, *J*_*C-F*_ = 8.82 Hz, C_3_), 115.6 (d, *J*_*C-F*_ = 21.42 Hz, C_2_). HRMS (ESI-TOF), m/z calcd. C_29_H_30_FN_6_O_10_ [M + H]^+^ 641.2002; Found: 641.1975.

***(2R,3R,4S,5R,6R)-2-(acetoxymethyl)-6-(4-(((5-(4-(trifluoromethyl)phenyl)pyrazolo[1,5-a]pyrimidin-7-yl)oxy)methyl)-1H-1,2,3-triazol-1-yl)tetrahydro-2H-pyran-3,4,5-triyl triacetate* (9f):** off-white colored sticky solid; yield: 117.47 mg (95%), Rf = 0.29 (EtOAc); ^1^H NMR (500 MHz, CDCl_3_) ^1^H NMR (500 MHz, CDCl_3_) δ 8.03 (m, 3H), 7.91 (d, *J* = 4.0 Hz, 1H), 7.65 (d, *J* = 8.0 Hz, 2H), 6.52 (s, 1H), 6.41 (d, *J* = 4.0 Hz, 1H), 6.16 (d, *J* = 14.7 Hz, 1H), 5.99 (d, *J* = 14.7 Hz, 1H), 5.82 (d, *J* = 8.0 Hz, 1H), 5.37 (t, *J* = 9.5 Hz, 1H), 5.32 (t, *J* = 9.5 Hz, 1H), 5.21 (t, *J* = 9.0 Hz, 1H), 4.23 (dd, *J* = 12.7, 4.0 Hz, 1H), 4.11 (dd, *J* = 12.0 Hz, 3.5 Hz, 1H), 3.99–3.96 (m, 1H), 2.01 (s, 3H), 2.00 (s, 3H), 1.95 (s, 3H), 1.60 (s, 3H). ^13^C NMR (125 MHz, CDCl_3_) δ 170.5, 169.9, 169.3, 168.4, 160.5, 158.1, 153.9, 141.4, 141.3, 140.1, 132.0, 131.7, 131.5, 131.2, 127.6, 125.6, 125.1, 123.3, 123.0, 100.2, 99.5, 85.8, 75.2, 72.3, 70.3, 67.7, 61.5, 47.1, 20.6, 20.5, 20.5, 19.8. ^13^C–^19^F couplings in ^13^C NMR (125 MHz, DMSO-*d*_6_) δ 131.6 (q, *J*_*C-F*_ = 31.5 Hz, C_2_), 125.5 (d, *J*_*C-F*_ = 3.78 Hz, C_3_), 124.0 (q, *J*_*C-F*_ = 272.16 Hz, C_1_). HRMS (ESI-TOF), m/z calcd. C_30_H_30_F_3_N_6_O_10_ [M + H]^+^ 691.1970; Found: 691.1949.

***(2R,3R,4S,5R,6R)-2-(acetoxymethyl)-6-(4-(((5-(4-isopropylphenyl)pyrazolo[1,5-a]pyrimidin-7-yl)oxy)methyl)-1H-1,2,3-triazol-1-yl)tetrahydro-2H-pyran-3,4,5-triyl triacetate* (9g):** gray colored sticky solid; yield: 123.81 mg (97%), Rf = 0.32 (EtOAc); ^1^H NMR (500 MHz, CDCl_3_) δ 8.00 (s, 1H), 7.85 (d, *J* = 8.0 Hz, 3H), 7.27 (d, *J* = 8.0 Hz, 2H), 6.50 (s, 1H), 6.39 (d, *J* = 3.9 Hz, 1H), 6.11 (d, *J* = 15.3 Hz, 1H), 5.99 (d, *J* = 15.0 Hz, 1H), 5.79 (d, *J* = 9.0 Hz, 1H), 5.37 (t, *J* = 10.0 Hz, 1H), 5.33 (t, *J* = 9.5 Hz, 1H), 5.20 (t, *J* = 9.5 Hz, 1H), 4.24 (dd, *J* = 13.0, 5.0 Hz, 1H), 4.09 (dd, *J* = 12.0 Hz, 3.5 Hz, 1H), 3.97–3.94 (m, 1H), 2.92 (hept, *J* = 7.5, 1H), 2.01 (s, 3H), 2.01 (s, 3H), 1.97 (s, 3H), 1.63 (s, 3H), 1.23 (d, *J* = 7.5 Hz, 6H). ^13^C NMR (125 MHz, CDCl_3_) δ 170.5, 169.9, 169.3, 168.4, 162.3, 158.4, 153.9, 151.1, 141.5, 139.8, 135.4, 127.3, 126.8, 123.4, 100.2, 98.6, 85.8, 75.2, 72.4, 70.3, 67.7, 61.4, 47.1, 34.0, 23.9, 20.6, 20.5, 19.8. HRMS (ESI-TOF), m/z calcd. C_32_H_37_N_6_O_10_ [M + H]^+^ 665.2566; Found: 665.2540.

***(2R,3R,4S,5R,6R)-2-(acetoxymethyl)-6-(4-(((5-(naphthalen-2-yl)pyrazolo[1,5-a]pyrimidin-7-yl)oxy)methyl)-1H-1,2,3-triazol-1-yl)tetrahydro-2H-pyran-3,4,5-triyl triacetate* (9h):** white colored sticky solid; yield: 123.57 mg (96%), Rf = 0.32 (EtOAc); ^1^H NMR (500 MHz, CDCl_3_) δ 8.47 (s, 1H), 8.09 (s, 1H), 8.01 (d, *J* = 9.3 Hz, 1H), 7.91 (d, *J* = 5.3 Hz, 2H), 7.87 (d, *J* = 8.0 Hz, 1H), 7.83–7.80 (m, 1H), 7.50–7.45 (m, 2H), 6.68 (s, 1H), 6.46 (d, *J* = 4.0 Hz, 1H), 6.15 (d, *J* = 15.8 Hz, 1H), 6.00 (d, *J* = 15.8 Hz, 1H), 5.81 (d, *J* = 9.1 Hz, 1H), 5.38–5.36 (m, 2H), 5.23–5.20 (m, 1H), 4.24 (dd, *J* = 12.7, 4.0 Hz, 1H), 4.10 (dd, *J* = 12.0 Hz, 3.5 Hz, 1H), 3.97–3.94 (m, 1H), 2.01 (s, 3H), 2.00 (s, 3H), 1.95 (s, 3H), 1.61 (s, 3H). ^13^C NMR (125 MHz, CDCl_3_) δ 170.5, 169.9, 169.3, 168.4, 162.0, 158.4, 153.9, 141.5, 140.0, 135.0, 134.1, 133.2, 128.9, 128.3, 127.6, 127.3, 127.0, 126.4, 124.4, 123.6, 100.1, 99.2, 85.8, 75.1, 72.4, 70.3, 67.6, 61.4, 47.1, 20.6, 20.5, 19.8. HRMS (ESI-TOF), m/z calcd. C_33_H_33_N_6_O_10_ [M + H]^+^ 673.2253; Found: 673.2235.

***(2R,3R,4S,5R,6R)-2-(acetoxymethyl)-6-(4-(((5-(benzo[d][1,3]dioxol-5-yl)pyrazolo[1,5-a]pyrimidin-7-yl)oxy)methyl)-1H-1,2,3-triazol-1-yl)tetrahydro-2H-pyran-3,4,5-triyl triacetate* (9i):** white colored sticky solid; yield: 126.67 mg (97%), Rf = 0.31 (EtOAc); ^1^H NMR (500 MHz, CDCl_3_) δ 7.98 (s, 1H), 7.84 (d, *J* = 5.3 Hz, 1H), 7.49 (d, *J* = 9.3 Hz, 1H), 7.44 (s, 1H), 6.84 (d, *J* = 8.0 Hz, 1H), 6.43 (s, 1H), 6.38 (d, *J* = 4.0 Hz, 1H), 6.10 (d, *J* = 14.7 Hz, 1H), 6.00 (d, *J* = 8.0 Hz, 1H), 5.99 (s, 2H) 5.78 (d, *J* = 9.3 Hz, 1H), 5.37 (t, *J* = 9.0 Hz, 1H), 5.33 (t, *J* = 9.5 Hz, 1H), 5.20 (t, *J* = 9.5 Hz, 1H), 4.24 (dd, *J* = 12.7, 5.5 Hz, 1H), 4.11 (dd, *J* = 12.0 Hz, 3.5 Hz, 1H), 3.97–3.93 (m, 1H), 2.04 (s, 3H), 2.03 (s, 3H), 1.98 (s, 3H), 1.65 (s, 3H). ^13^C NMR (125 MHz, CDCl_3_) δ 170.5, 170.0, 169.3, 168.5, 161.7, 158.3, 153.8, 149.3, 148.2, 141.5, 139.9, 132.1, 123.3, 121.8, 108.4, 107.6, 101.5, 100.2, 98.3, 85.9, 75.3, 72.4, 70.3, 67.7, 61.4, 47.1, 20.7, 20.6, 19.9. HRMS (ESI-TOF), m/z calcd. C_30_H_31_N_6_O_12_ [M + H]^+^ 667.1994; Found: 667.1964.

### Synthesis of triazole-linked galactohybrids of pyrazolo[1,5-***a***]pyrimidines 10a–10i

In a microwave vial, a mixture of 50 mg (0.189 mmol) of *O*-propargylated pyrazolo[1,5-*a*]pyrimidine **6a** and 70.89 mg (0.189 mmol) of 1-azido galactoside **8b** in 2 ml of solvent (1:1, v/v mixture of *t*-BuOH-H_2_O) was treated with CuSO_4_·5H_2_O (1.24 mg, 0.005 mmol) and sodium ascorbate (2.23 mg, 0.011 mmol), and then subjected to microwave heating for 20 min at 50 °C (100 W). The reaction was monitored by TLC to confirm completion, and upon completion, the reaction mixture was extracted with 5 ml of water and 3 ml of EtOAc. The organic layer was then dried over Na_2_SO_4_ and evaporated via a rotary evaporator to obtain the crude residue, which was purified by flash column chromatography to yield pure compound **10a** in 98% isolated yield. Using similar methods, with 50 mg of propargylated reactants, compounds **10b**–**10i** were synthesized in good to excellent yields utilizing 1-azido galactose tetraacetate.

***(2R,3S,4S,5R,6R)-2-(acetoxymethyl)-6-(4-(((5-(p-tolyl)pyrazolo[1,5-a]pyrimidin-7-yl)oxy)methyl)-1H-1,2,3-triazol-1-yl)tetrahydro-2H-pyran-3,4,5-triyl triacetate* (10a):** yellow colored sticky solid; yield: 117.19 mg (97%), Rf = 0.32 (EtOAc);^1^H NMR (500 MHz, CDCl_3_) δ 8.01 (s, 1H), 7.86 (d, *J* = 4.0 Hz, 1H), 7.84 (d, *J* = 8.0 Hz, 2H), 7.23 (d, *J* = 8.0 Hz, 2H), 6.52 (s, 1H), 6.41 (d, *J* = 4.0 Hz, 1H), 6.05 (br s, 2H), 5.72 (d, *J* = 9.0 Hz, 1H), 5.49 (dd, *J* = 9.0 Hz, 4.0 Hz, 1H), 5.42 (t, *J* = 10.0 Hz, 1H), 5.18 (dd, *J* = 10.7, 4.0 Hz, 1H), 4.18–4.13 (m, 2H), 4.10–4.06 (m, 1H), 2.37 (s, 3H), 2.21 (s, 3H), 1.96 (s, 3H), 1.95 (s, 3H), 1.66 (s, 3H). ^13^C NMR (125 MHz, CDCl_3_) δ 170.3, 170.1, 169.8, 168.6, 162.2, 158.4, 153.9, 141.4, 140.2, 140.0, 135.0, 129.4, 127.2, 123.2, 100.3, 98.6, 86.4, 74.2, 70.6, 67.8, 66.8, 61.2, 47.0, 21.3, 20.7, 20.6, 20.5, 19.9. HRMS (ESI-TOF), m/z calcd. C_30_H_33_N_6_O_10_ [M + H]^+^ 637.2253; Found: 637.2281.

***(2R,3S,4S,5R,6R)-2-(acetoxymethyl)-6-(4-(((5-(4-methoxyphenyl)pyrazolo[1,5-a]pyrimidin-7-yl)oxy)methyl)-1H-1,2,3-triazol-1-yl)tetrahydro-2H-pyran-3,4,5-triyl triacetate* (10b):** yellow colored sticky solid; yield 113.32 mg (97%), Rf = 0.31 (EtOAc); ^1^H NMR (500 MHz, CDCl_3_) δ 8.01 (s, 1H), 7.90 (d, *J* = 8.0 Hz, 2H), 7.85 (d, *J* = 5.3 Hz, 1H), 6.93 (d, *J* = 8.0 Hz, 2H), 6.48 (s, 1H), 6.39 (d, *J* = 4.0 Hz, 1H), 6.04 (s, 2H), 5.72 (d, *J* = 9.3 Hz, 1H), 5.49 (dd, *J* = 9.0 Hz, 4.0 Hz, 1H), 5.42 (t, *J* = 10.0 Hz, 1H), 5.17 (dd, *J* = 10.0, 4.0 Hz, 1H), 4.17–4.12 (m, 2H), 4.10–4.04 (m, 1H), 3.82 (s, 3H), 2.21 (s, 3H), 1.96 (s, 3H), 1.95 (s, 3H), 1.66 (s, 3H). ^13^C NMR (125 MHz, CDCl_3_) δ 170.3, 170.1, 169.8, 168.7, 161.8, 161.3, 158.4, 153.9, 141.4, 140.0, 130.2, 128.7, 123.2, 114.0, 100.2, 98.0, 86.4, 74.2, 70.6, 67.8, 66.8, 61.2, 55.4, 47.0, 20.7, 20.6, 20.5, 19.9. HRMS (ESI-TOF), m/z calcd. C_30_H_33_N_6_O_11_[M + H]^+^ 653.2202; Found: 653.2311.

***(2R,3S,4S,5R,6R)-2-(acetoxymethyl)-6-(4-(((5-(4-bromophenyl)pyrazolo[1,5-a]pyrimidin-7-yl)oxy)methyl)-1H-1,2,3-triazol-1-yl)tetrahydro-2H-pyran-3,4,5-triyl triacetate* (10c):** yellow colored sticky solid; yield: 101.64 mg (98%), Rf = 0.33 (EtOAc); ^1^H NMR (500 MHz, CDCl_3_) δ 8.01 (s, 1H), 7.89 (d, *J* = 3.7 Hz, 1H), 7.81 (d, *J* = 8.0 Hz, 2H), 7.55 (d, *J* = 9.2 Hz, 2H), 6.50 (s, 1H), 6.41 (d, *J* = 3.9 Hz, 1H), 6.06 (br s, 2H), 5.73 (d, *J* = 9.3 Hz, 1H), 5.50 (dd, *J* = 9.5 Hz, 4.0 Hz, 1H), 5.42 (t, *J* = 10.0 Hz, 1H), 5.18 (dd, *J* = 10.0, 4.0 Hz, 1H), 4.18–4.13 (m, 2H), 4.10–4.05 (m, 1H), 2.21 (s, 3H), 1.97 (s, 3H), 1.96 (s, 3H), 1.66 (s, 3H). ^13^C NMR (125 MHz, CDCl_3_) δ 170.3, 170.1, 169.8, 168.7, 161.0, 158.3, 153.9, 141.4, 140.1, 136.8, 131.9, 128.8, 124.6, 123.2, 100.2, 98.9, 86.5, 74.3, 70.6, 67.9, 66.8, 61.2, 47.1, 20.7, 20.6, 20.5, 19.9. HRMS (ESI-TOF), m/z calcd. C_29_H_30_BrN_6_O_10_ [M + H]^+^ 701.1201; Found: 701.1226.

***(2R,3S,4S,5R,6R)-2-(acetoxymethyl)-6-(4-(((5-(4-chlorophenyl)pyrazolo[1,5-a]pyrimidin-7-yl)oxy)methyl)-1H-1,2,3-triazol-1-yl)tetrahydro-2H-pyran-3,4,5-triyl triacetate* (10d):** yellow colored sticky solid; yield: 116.42 mg (97%), Rf = 0.33 (EtOAc); ^1^H NMR (500 MHz, CDCl_3_) δ 8.00 (s, 1H), 7.88 (d, *J* = 5.3 Hz, 2H), 7.86 (s, 1H), 7.37 (d, *J* = 8.0 Hz, 2H), 6.48 (s, 1H), 6.39 (d, *J* = 4.0 Hz, 1H), 6.05 (br s, 2H), 5.74 (d, *J* = 9.3 Hz, 1H), 5.49 (dd, *J* = 9.0 Hz, 4.0 Hz, 1H), 5.40 (t, *J* = 10.5 Hz, 1H), 5.18 (dd, *J* = 10.4, 4.0 Hz, 1H), 4.19–4.12 (m, 2H), 4.08–4.04 (m, 1H), 2.20 (s, 3H), 1.95 (s, 3H), 1.94 (s, 3H), 1.64 (s, 3H). ^13^C NMR (125 MHz, CDCl_3_) δ 170.2, 170.0, 169.8, 168.6, 160.8, 158.2, 153.9, 141.4, 140.1, 136.3, 136.1, 128.8, 128.5, 123.2, 100.2, 98.8, 86.4, 74.2, 70.5, 67.9, 66.8, 61.1, 47.1, 20.7, 20.6, 20.4, 19.9. HRMS (ESI-TOF), m/z calcd. C_29_H_30_ClN_6_O_10_ [M + H]^+^ 657.1706; Found: 657.1728.

***(2R,3S,4S,5R,6R)-2-(acetoxymethyl)-6-(4-(((5-(4-fluorophenyl)pyrazolo[1,5-a]pyrimidin-7-yl)oxy)methyl)-1H-1,2,3-triazol-1-yl)tetrahydro-2H-pyran-3,4,5-triyl triacetate* (10e):** light yellow colored sticky solid; yield: 131.34 mg (96%), Rf = 0.30 (EtOAc); ^1^H NMR (500 MHz, CDCl_3_) δ 7.99 (s, 1H), 7.90 (t, *J* = 6.8 Hz, 2H), 7.87 (d, *J* = 4.0 Hz, 1H), 6.03 (br s, 2H), 5.74 (d, *J* = 9.0 Hz, 1H), 5.49 (dd, *J* = 9.0 Hz, 5.0 Hz, 1H), 5.39 (t, *J* = 10.0 Hz, 1H), 5.18 (dd, *J* = 10.1, 4.0 Hz, 1H), 4.19–4.10 (m, 2H), 4.07–4.03 (m, 1H), 2.17 (s, 3H), 1.92 (s, 6H), 1.62 (s, 3H). ^13^C NMR (125 MHz, CDCl_3_) δ 170.2, 170.0, 169.7, 168.6, 165.0, 163.0, 161.0, 158.2, 153.8, 141.3, 140.0, 133.9, 129.2, 129.1, 123.1, 115.6, 115.4, 100.1, 98.5, 86.3, 74.1, 70.5, 67.8, 66.8, 61.1, 47.0, 20.6, 20.5, 20.4, 19.8. ^13^C–^19^F couplings in ^13^C NMR (125 MHz, DMSO-*d*_6_) δ 164.0 (d, *J*_*C-F*_ = 250.74 Hz, C_1_), 129.1 (d, *J*_*C-F*_ = 8.82 Hz, C_3_), 115.5 (d, *J*_*C-F*_ = 21.42 Hz, C_2_). HRMS (ESI-TOF), m/z calcd. C_29_H_30_FN_6_O_10_ [M + H]^+^ 641.2002; Found: 641.2027.

***(2R,3S,4S,5R,6R)-2-(acetoxymethyl)-6-(4-(((5-(4-(trifluoromethyl)phenyl)pyrazolo[1,5-a]pyrimidin-7-yl)oxy)methyl)-1H-1,2,3-triazol-1-yl)tetrahydro-2H-pyran-3,4,5-triyl triacetate* (10f):** white colored sticky solid; yield: 115 mg (93%), Rf = 0.29 (EtOAc); ^1^H NMR (500 MHz, CDCl_3_) δ 8.06 (d, *J* = 8.0 Hz, 2H), 8.03 (s, 1H), 7.91 (d, *J* = 4.0 Hz, 1H), 6.08 (br s, 2H), 5.74 (d, *J* = 9.0 Hz, 1H), 5.51 (dd, *J* = 9.0 Hz, 4.0 Hz, 1H), 5.42 (t, *J* = 10.0 Hz, 1H), 5.19 (dd, *J* = 10.0, 5.5 Hz, 1H), 4.19–4.14 (m, 2H), 4.11–4.06 (m, 1H), 2.22 (s, 3H), 1.98 (s, 3H), 1.96 (s, 3H), 1.67 (s, 3H). ^13^C NMR (125 MHz, CDCl_3_) δ 170.3, 170.1, 169.8, 168.7, 160.6, 158.3, 154.0, 141.4, 141.4, 140.1, 131.9, 131.6, 131.4, 127.6, 125.7, 125.2, 123.2, 100.3, 99.6, 86.5, 74.3, 70.6, 67.9, 66.8, 61.2, 47.2, 20.8, 20.6, 20.5, 20.0. ^13^C–^19^F couplings in ^13^C NMR (125 MHz, DMSO-*d*_6_) δ 131.53 (q, *J*_*C-F*_ = 31.5 Hz, C_2_), 125.70 (d, *J*_*C-F*_ = 3.78 Hz, C_3_), 124.13 (q, *J*_*C-F*_ = 272.16 Hz, C_1_). HRMS (ESI-TOF), m/z calcd. C_30_H_30_F_3_N_6_O_10_ [M + H]^+^ 691.1970; Found: 691.1990.

***(2R,3S,4S,5R,6R)-2-(acetoxymethyl)-6-(4-(((5-(4-isopropylphenyl)pyrazolo[1,5-a]pyrimidin-7-yl)oxy)methyl)-1H-1,2,3-triazol-1-yl)tetrahydro-2H-pyran-3,4,5-triyl triacetate* (10g):** yellow colored sticky solid; yield: 122.53 mg (96%), Rf = 0.33 (EtOAc); ^1^H NMR (500 MHz, CDCl_3_) δ 8.01 (s, 1H), 7.86 (s, 1H), 7.85 (s, 2H), 7.27 (d, *J* = 8.0 Hz, 2H), 6.52 (s, 1H), 6.39 (d, *J* = 4.0 Hz, 1H), 6.04 (br s, 2H), 5.72 (d, *J* = 9.0 Hz, 1H), 5.49 (dd, *J* = 10.0 Hz, 4.0 Hz, 1H), 5.41 (t, *J* = 10.0 Hz, 1H), 5.18 (dd, *J* = 10.5, 5.5 Hz, 1H), 4.18–4.12 (m, 2H), 4.10–4.05 (m, 1H), 2.91 (hept, *J* = 6.7 Hz, 1H), 2.20 (s, 3H), 1.95 (s, 6H), 1.65 (s, 3H), 1.23 (d, *J* = 6.8 Hz, 6H). ^13^C NMR (125 MHz, CDCl_3_) δ 170.3, 170.1, 169.8, 168.6, 162.3, 158.4, 153.9, 151.1, 141.4, 139.9, 135.4, 127.2, 126.8, 123.2, 100.3, 98.7, 86.4, 74.2, 70.6, 67.8, 66.8, 61.2, 47.0, 34.0, 23.8, 20.7, 20.6, 20.5, 19.9. HRMS (ESI-TOF), m/z calcd. C_32_H_37_N_6_O_10_ [M + H]^+^ 665.2566; Found: 665.2656.

***(2R,3S,4S,5R,6R)-2-(acetoxymethyl)-6-(4-(((5-(naphthalen-2-yl)pyrazolo[1,5-a]pyrimidin-7-yl)oxy)methyl)-1H-1,2,3-triazol-1-yl)tetrahydro-2H-pyran-3,4,5-triyl triacetate* (10h):** yellow colored sticky solid; yield: 124.86 mg (97%), Rf = 0.32 (EtOAc); ^1^H NMR (500 MHz, CDCl_3_) δ 8.48 (s, 1H), 8.02 (d, *J* = 12.0 Hz, 2H), 7.89 (dt, *J* = 17.3, 6.7 Hz, 3H), 7.84–7.80 (m, 1H), 7.50–7.44 (m, 2H), 6.68 (s, 1H), 6.45 (d, *J* = 5.3 Hz, 1H), 6.06 (br s, 2H), 5.74 (d, *J* = 9.5 Hz, 1H), 5.49 (dd, *J* = 10.0 Hz, 4.0 Hz, 1H), 5.43 (t, *J* = 9.5 Hz, 1H), 5.19 (dd, *J* = 10.7, 5.5 Hz, 1H), 4.18–4.12 (m, 2H), 4.10–4.05 (m, 1H), 2.20 (s, 3H), 1.93 (s, 6H), 1.65 (s, 3H). ^13^C NMR (125 MHz, CDCl_3_) δ 170.2, 170.0, 169.8, 168.6, 161.9, 158.3, 153.9, 141.4, 140.0, 135.0, 134.1, 133.2, 128.9, 128.3, 127.6, 127.2, 127.0, 126.4, 124.4, 123.2, 100.2, 99.3, 86.3, 74.1, 70.5, 67.8, 66.8, 61.2, 47.0, 20.7, 20.5, 20.4, 19.9. HRMS (ESI-TOF), m/z calcd. C_33_H_33_N_6_O_10_ [M + H]^+^ 673.2253; Found: 673.2346.

***(2R,3S,4S,5R,6R)-2-(acetoxymethyl)-6-(4-(((5-(benzo[d][1,3]dioxol-5-yl)pyrazolo[1,5-a]pyrimidin-7-yl)oxy)methyl)-1H-1,2,3-triazol-1-yl)tetrahydro-2H-pyran-3,4,5-triyl triacetate* (10i):** yellow colored sticky solid; yield: 127.97 mg (98%), Rf = 0.33 (EtOAc); ^1^H NMR (500 MHz, CDCl_3_) δ 8.00 (s, 1H), 7.84 (d, *J* = 3.1 Hz, 1H), 7.46 (d, *J* = 8.3 Hz, 1H), 7.42 (s, 1H), 6.82 (d, *J* = 8.0 Hz, 1H), 6.41 (s, 1H), 6.35 (d, *J* = 3.8 Hz, 1H), 6.02 (br s, 2H), 5.73 (d, *J* = 9.0 Hz, 1H), 5.48 (dd, *J* = 10.0 Hz, 3.5 Hz, 1H), 5.41 (t, *J* = 9.5 Hz, 1H), 5.17 (dd, *J* = 10.5, 3.0 Hz, 1H), 4.18–4.11 (m, 2H), 4.08–4.04 (m, 1H), 2.19 (s, 3H), 1.95 (s, 3H), 1.93 (s, 3H), 1.64 (s, 3H). ^13^C NMR (125 MHz, CDCl_3_) δ 170.2, 170.0, 169.8, 168.6, 161.5, 158.3, 153.7, 149.3, 148.1, 141.4, 140.0, 132.0, 123.2, 121.7, 108.3, 107.5, 101.4, 100.1, 98.2, 86.3, 74.1, 70.6, 67.8, 66.8, 61.1, 47.0, 20.7, 20.5, 20.4, 19.9. HRMS (ESI-TOF), m/z calcd. C_30_H_31_N_6_O_12_ [M + H]^+^ 667.1994; Found: 667.2114.

### Synthesis of triazole-linked mannohybrids of pyrazolo[1,5-***a***]pyrimidines 11a–11i

In a microwave vial, a mixture of 50 mg (0.189 mmol) of *O*-propargylated pyrazolo[1,5-*a*]pyrimidine **6a** and 70.89 mg (0.189 mmol) of 1-azido mannoside **8c** in 2 ml of solvent (1:1, v/v mixture of *t*-BuOH–H_2_O) was treated with CuSO_4_·5H_2_O (1.24 mg, 0.005 mmol) and sodium ascorbate (2.238 mg, 0.011 mmol), and then subjected to microwave heating for 20 min at 50 °C (100 W). The reaction was monitored by TLC to confirm completion, and upon completion, the reaction mixture was extracted with 5 ml of water and 3 ml of EtOAc. The organic layer was then dried over Na_2_SO_4_ and evaporated via a rotary evaporator to obtain the crude residue, which was purified by flash column chromatography to yield pure compound **11a** in 98% isolated yield. Using similar methods, with 50 mg of propargylated reactants, compounds **11b**–**11i** were synthesized in good to excellent yields utilizing 1-azido mannose tetraacetate.

***(2R,3R,4S,5S,6S)-2-(acetoxymethyl)-6-(4-(((5-(p-tolyl)pyrazolo[1,5-a]pyrimidin-7-yl)oxy)methyl)-1H-1,2,3-triazol-1-yl)tetrahydro-2H-pyran-3,4,5-triyl triacetate* (11a):** brown colored sticky solid; yield: 115.98 mg (96%), Rf = 0.31 (EtOAc); ^1^H NMR (500 MHz, CDCl_3_) δ 7.95 (s, 1H), 7.89 (s, 1H), 7.82 (d, *J* = 8.0 Hz, 2H), 7.21 (d, *J* = 8.0 Hz, 2H), 6.46 (s, 1H), 6.42 (s, 1H), 6.16 (d, *J* = 14.7 Hz, 1H), 5.91 (d, *J* = 2.5 Hz, 1H), 5.36 (t, *J* = 4.0 Hz, 1H), 5.76 (dd, *J* = 8.0 Hz, 4.0 Hz, 1H), 5.27 (t, *J* = 9.5 Hz, 1H), 4.30 (dd, *J* = 12.6, 6.0 Hz, 1H), 3.97 (dd, *J* = 12.0 Hz, 3.0 Hz, 1H), 3.77–3.74 (m, 1H), 2.35 (s, 3H), 2.07 (s, 3H), 2.01 (s, 3H), 2.00 (s, 3H), 1.99 (s, 3H). ^13^C NMR (125 MHz, CDCl_3_) δ 170.4, 169.6, 169.5, 169.3, 162.3, 158.2, 153.8, 141.8, 140.3, 139.6, 134.9, 129.4, 127.1, 124.9, 100.0, 98.1, 83.6, 72.3, 68.6, 68.0, 66.0, 61.4, 47.0, 21.3, 20.6, 20.5. HRMS (ESI-TOF), m/z calcd. C_30_H_33_N_6_O_10_ [M + H]^+^ 637.2253; Found: 637.2230.

***(2R,3R,4S,5S,6S)-2-(acetoxymethyl)-6-(4-(((5-(4-methoxyphenyl)pyrazolo[1,5-a]pyrimidin-7-yl)oxy)methyl)-1H-1,2,3-triazol-1-yl)tetrahydro-2H-pyran-3,4,5-triyl triacetate* (11b):** yellow colored sticky solid; yield: 108.65 mg (93%) Rf = 0.30 (EtOAc); ^1^H NMR (500 MHz, CDCl_3_) δ 7.95 (s, 1H), 7.91 (d, *J* = 8.5 Hz, 2H), 7.88 (d, *J* = 3.9 Hz, 1H), 6.94 (d, *J* = 8.2 Hz, 2H), 6.44 (s, 1H), 6.43 (d, *J* = 2.9 Hz, 1H), 6.18 (d, *J* = 14.8 Hz, 1H), 5.99 (d, *J* = 15.3 Hz, 1H), 5.92 (d, *J* = 3.5 Hz, 1H), 5.87 (t, *J* = 4.5 Hz, 1H), 5.78 (dd, *J* = 8.8 Hz, 4.0 Hz, 1H), 5.29 (t, *J* = 9.5 Hz, 1H), 4.32 (dd, *J* = 12.2, 5.5 Hz, 1H), 4.00 (dd, *J* = 12.0 Hz, 2.5 Hz, 1H), 3.84 (s, 3H), 3.79–3.76 (m, 1H), 2.10 (s, 3H), 2.03 (s, 3H), 2.02 (s, 3H), 2.01 (s, 3H). ^13^C NMR (125 MHz, CDCl_3_) δ 170.5, 169.6, 169.6, 169.4, 162.1, 161.4, 158.4, 153.8, 141.8, 139.7, 130.1, 128.8, 125.0, 114.1, 100.1, 97.5, 83.7, 72.5, 68.7, 68.1, 66.1, 61.5, 55.4, 47.1, 20.7, 20.6. HRMS (ESI-TOF), m/z calcd. C_30_H_33_N_6_O_11_[M + H]^+^ 653.2202; Found: 653.2157.

***(2R,3R,4S,5S,6S)-2-(acetoxymethyl)-6-(4-(((5-(4-bromophenyl)pyrazolo[1,5-a]pyrimidin-7-yl)oxy)methyl)-1H-1,2,3-triazol-1-yl)tetrahydro-2H-pyran-3,4,5-triyl triacetate* (11c):** yellow colored sticky solid; yield: 97.49 mg (94%), Rf = 0.32 (EtOAc); ^1^H NMR (500 MHz, CDCl_3_) δ 7.95 (s, 1H), 7.92 (d, *J* = 4.0 Hz, 1H), 7.80 (d, *J* = 8.0 Hz, 2H), 7.54 (d, *J* = 9.3 Hz, 2H), 6.43 (s, 2H), 6.20 (d, *J* = 15.7 Hz, 1H), 5.98 (d, *J* = 14.8 Hz, 1H), 5.91 (d, *J* = 2.5 Hz, 1H), 5.85 (t, *J* = 3.5 Hz, 1H), 5.77 (dd, *J* = 9.2 Hz, 4.0 Hz, 1H), 5.29 (t, *J* = 9.5 Hz, 1H), 4.30 (dd, *J* = 12.6, 5.5 Hz, 1H), 3.99 (dd, *J* = 12.5 Hz, 2.5 Hz, 1H), 3.79–3.76 (m, 1H), 2.09 (s, 3H), 2.02 (s, 3H), 2.01 (s, 3H), 2.00 (s, 3H). ^13^C NMR (125 MHz, CDCl_3_) δ 170.5, 169.6, 169.3, 161.1, 158.2, 153.7, 141.8, 139.7, 136.6, 131.8, 128.8, 124.9, 124.6, 100.0, 98.3, 83.7, 72.4, 68.6, 68.1, 66.0, 61.4, 47.2, 20.6, 20.6. HRMS (ESI-TOF), m/z calcd. C_29_H_30_BrN_6_O_10_ [M + H]^+^ 701.1201; Found: 701.1158.

***(2R,3R,4S,5S,6S)-2-(acetoxymethyl)-6-(4-(((5-(4-chlorophenyl)pyrazolo[1,5-a]pyrimidin-7-yl)oxy)methyl)-1H-1,2,3-triazol-1-yl)tetrahydro-2H-pyran-3,4,5-triyl triacetate* (11d):** brown colored sticky solid; yield: 114.02 mg (95%), Rf = 0.31 (EtOAc); ^1^H NMR (500 MHz, CDCl_3_) δ 7.94 (s, 1H), 7.92 (d, *J* = 4.0 Hz, 1H), 7.87 (d, *J* = 9.3 Hz, 2H), 7.38 (d, *J* = 8.0 Hz, 2H), 6.43 (s, 2H), 6.19 (d, *J* = 14.7 Hz, 1H), 5.92 (d, *J* = 2.5 Hz, 1H), 5.85 (t, *J* = 4.0 Hz, 1H), 5.77 (dd, *J* = 9.0 Hz, 4.0 Hz, 1H), 5.29 (t, *J* = 9.0 Hz, 1H), 4.30 (dd, *J* = 12.6, 5.5 Hz, 1H), 3.99 (dd, *J* = 12.5 Hz, 2.5 Hz, 1H), 3.79–3.76 (m, 1H), 2.08 (s, 3H), 2.02 (s, 3H), 2.01 (s, 3H), 2.00 (s, 3H). ^13^C NMR (125 MHz, CDCl_3_) δ 170.5, 169.6, 169.3, 161.1, 158.2, 153.7, 141.8, 139.7, 136.2, 136.2, 128.9, 128.6, 124.9, 100.0, 98.3, 83.7, 72.4, 68.6, 68.0, 66.0, 61.4, 47.1, 20.6, 20.6. HRMS (ESI-TOF), m/z calcd. C_29_H_30_ClN_6_O_10_ [M + H]^+^ 657.1706; Found: 657.1640.

***(2R,3R,4S,5S,6S)-2-(acetoxymethyl)-6-(4-(((5-(4-fluorophenyl)pyrazolo[1,5-a]pyrimidin-7-yl)oxy)methyl)-1H-1,2,3-triazol-1-yl)tetrahydro-2H-pyran-3,4,5-triyl triacetate* (11e):** off-white colored sticky solid; yield: 127.16 mg (91%), Rf = 0.31 (EtOAc); ^1^H NMR (500 MHz, CDCl_3_) δ 7.94 (s, 1H), 7.92 (m, 3H), 7.09 (d, *J* = 8.7 Hz, 2H), 6.42 (d, *J* = 2.7 Hz, 1H), 6.41 (s, 1H), 6.19 (d, *J* = 16.0 Hz, 1H), 5.98 (d, *J* = 14.7 Hz, 1H), 5.92 (d, *J* = 4.0 Hz, 1H), 5.85 (t, *J* = 4.0 Hz, 1H), 5.77 (dd, *J* = 9.0 Hz, 4.0 Hz, 1H), 5.29 (t, *J* = 9.0 Hz, 1H), 4.30 (dd, *J* = 12.0, 5.5 Hz, 1H), 3.98 (dd, *J* = 12.0 Hz, 2.5 Hz, 1H), 3.78–3.75 (m, 1H), 2.08 (s, 3H), 2.02 (s, 3H), 2.00 (s, 3H), 1.99 (s, 3H). ^13^C NMR (125 MHz, CDCl_3_) δ 170.5, 169.6, 169.3, 165.1, 163.1, 161.3, 158.2, 153.7, 141.8, 139.7, 133.9, 129.3, 129.2, 124.9, 115.7, 115.5, 100.0, 98.1, 83.7, 72.4, 68.6, 68.0, 66.0, 61.4, 47.1, 20.6, 20.6. ^13^C–^19^F couplings in ^13^C NMR (125 MHz, DMSO-*d*_6_) δ 164.16 (d, *J*_*C-F*_ = 249.48 Hz, C_1_), 129.49 (d, *J*_*C-F*_ = 8.82 Hz, C_3_), 115.67 (d, *J*_*C-F*_ = 21.42 Hz, C_2_). HRMS (ESI-TOF), m/z calcd. C_29_H_30_FN_6_O_10_ [M + H]^+^ 641.2002; Found: 641.1956.

***(2R,3R,4S,5S,6S)-2-(acetoxymethyl)-6-(4-(((5-(4-(trifluoromethyl)phenyl)pyrazolo[1,5-a]pyrimidin-7-yl)oxy)methyl)-1H-1,2,3-triazol-1-yl)tetrahydro-2H-pyran-3,4,5-triyl triacetate* (11f):** yellow colored sticky solid; 111.29 mg (90%), Rf = 0.30 (EtOAc); ^1^H NMR (500 MHz, CDCl_3_) δ 8.03 (s, 1H), 8.01 (s, 1H), 7.96 (d, *J* = 5.3 Hz, 2H), 7.65 (d, *J* = 8.0 Hz, 2H), 6.46 (s, 1H), 6.44 (d, *J* = 4.0 Hz, 1H), 6.20 (d, *J* = 16.0 Hz, 1H), 5.99 (d, *J* = 14.7 Hz, 1H), 5.92 (d, *J* = 4.0 Hz, 1H), 5.84 (t, *J* = 4.0 Hz, 1H), 5.76 (dd, *J* = 9.0 Hz, 4.0 Hz, 1H), 5.28 (t, *J* = 9.0 Hz, 1H), 4.29 (dd, *J* = 12.0, 5.5 Hz, 1H), 3.98 (dd, *J* = 12.0 Hz, 2.5 Hz, 1H), 3.78–3.76 (m, 1H), 2.07 (s, 3H), 2.01 (s, 3H), 1.99 (s, 3H), 1.98 (s, 3H). ^13^C NMR (125 MHz, CDCl_3_) δ 170.4, 169.5, 169.3, 160.6, 158.1, 153.7, 141.7, 141.2, 139.8, 132.0, 131.8, 131.5, 131.3, 127.5, 127.3, 125.6, 125.1, 124.9, 122.9, 120.8, 100.0, 98.9, 83.6, 72.4, 68.6, 68.0, 65.9, 61.4, 47.1, 20.6, 20.5. ^13^C–^19^F couplings in ^13^C NMR (125 MHz, DMSO-*d*_6_) δ 131.69 (q, *J*_*C-F*_ = 32.76 Hz, C_2_), 125.60 (d, *J*_*C-F*_ = 3.78 Hz, C_3_), 124.05 (q, *J*_*C-F*_ = 273.42 Hz, C_1_). HRMS (ESI-TOF), m/z calcd. C_30_H_30_F_3_N_6_O_10_ [M + H]^+^ 691.1970; Found: 691.1908.

***(2R,3R,4S,5S,6S)-2-(acetoxymethyl)-6-(4-(((5-(4-isopropylphenyl)pyrazolo[1,5-a]pyrimidin-7-yl)oxy)methyl)-1H-1,2,3-triazol-1-yl)tetrahydro-2H-pyran-3,4,5-triyl triacetate* (11g):** yellow colored sticky solid; yield: 120 mg (94%), Rf = 0.33 (EtOAc); ^1^H NMR (500 MHz, CDCl_3_) δ 7.93 (s, 1H), 7.88 (d, *J* = 4.0 Hz, 1H), 7.83 (d, *J* = 8.0 Hz, 2H), 7.25 (d, *J* = 8.0 Hz, 2H), 6.44 (s, 1H), 6.41 (d, *J* = 4.0 Hz, 1H), 6.14 (d, *J* = 16.0 Hz, 1H), 5.90 (d, *J* = 4.0 Hz, 1H), 5.84 (t, *J* = 4.0 Hz, 1H), 5.74 (dd, *J* = 9.0 Hz, 4.0 Hz, 1H), 5.26 (t, *J* = 9.0 Hz, 1H), 4.29 (dd, *J* = 12.5, 5.5 Hz, 1H), 3.96 (dd, *J* = 12.0 Hz, 3.0 Hz, 1H), 3.75–3.72 (m, 1H), 2.90 (hept, *J* = 6.7 Hz, 1H), 2.06 (s, 3H), 2.00 (s, 3H), 1.98 (s, 6H), 1.22 (d, *J* = 6.7 Hz, 6H). ^13^C NMR (125 MHz, CDCl_3_) δ 170.5, 169.6, 169.5, 169.3, 162.4, 158.3, 153.7, 151.2, 141.8, 139.6, 135.2, 127.3, 126.8, 124.9, 100.1, 98.1, 83.6, 72.4, 68.7, 68.0, 66.0, 61.4, 47.1, 34.0, 23.8, 20.6, 20.5. HRMS (ESI-TOF), m/z calcd. C_32_H_37_N_6_O_10_ [M + H]^+^ 665.2566; Found: 665.2513.

***(2R,3R,4S,5S,6S)-2-(acetoxymethyl)-6-(4-(((5-(naphthalen-2-yl)pyrazolo[1,5-a]pyrimidin-7-yl)oxy)methyl)-1H-1,2,3-triazol-1-yl)tetrahydro-2H-pyran-3,4,5-triyl triacetate* (11h):** yellow colored sticky solid; yield: 122.28 mg (95%), Rf = 0.32 (EtOAc); ^1^H NMR (500 MHz, CDCl_3_) δ 8.47 (s, 1H), 8.01 (d, *J* = 8.0 Hz, 1H), 7.98 (s, 1H), 7.94–7.90 (m, 2H), 7.88 (d, *J* = 8.0 Hz, 1H), 7.85–7.81 (m, 1H), 7.51–7.45 (m, 2H), 6.63 (s, 1H), 6.48 (d, *J* = 4.0 Hz, 1H), 6.20 (d, *J* = 14.7 Hz, 1H), 6.00 (d, *J* = 16.0 Hz, 1H), 5.93 (d, *J* = 3.0 Hz, 1H), 5.87 (t, *J* = 4.0 Hz, 1H), 5.78 (dd, *J* = 9.0 Hz, 4.0 Hz, 1H), 5.29 (t, *J* = 8.0 Hz, 1H), 4.31 (dd, *J* = 12.0, 5.0 Hz, 1H), 3.99 (dd, *J* = 13.0 Hz, 3.0 Hz, 1H), 3.79–3.76 (m, 1H), 2.08 (s, 3H), 2.02 (s, 3H), 2.00 (s, 3H), 1.99 (s, 3H). ^13^C NMR (125 MHz, CDCl_3_) δ 170.5, 169.6, 169.5, 169.3, 162.2, 158.2, 153.8, 141.8, 139.7, 135.0, 134.2, 133.2, 129.0, 128.4, 127.7, 127.3, 127.0, 126.4, 124.9, 124.4, 100.1, 98.8, 83.6, 72.4, 68.6, 68.0, 66.0, 61.4, 47.1, 20.6, 20.5. HRMS (ESI-TOF), m/z calcd. C_33_H_33_N_6_O_10_ [M + H]^+^ 673.2253; Found: 673.2241.

***(2R,3R,4S,5S,6S)-2-(acetoxymethyl)-6-(4-(((5-(benzo[d][1,3]dioxol-5-yl)pyrazolo[1,5-a]pyrimidin-7-yl)oxy)methyl)-1H-1,2,3-triazol-1-yl)tetrahydro-2H-pyran-3,4,5-triyl triacetate* (11i):** yellow colored sticky solid; yield: 125.36 mg (96%), Rf = 0.31 (EtOAc); ^1^H NMR (500 MHz, CDCl_3_) δ 7.94 (s, 1H), 7.89 (d, *J* = 4.0 Hz, 1H), 7.47 (d, *J* = 8.0 Hz, 1H), 7.43 (s, 1H), 6.83 (d, *J* = 8.0 Hz, 1H), 6.40 (d, *J* = 4.0 Hz, 1H), 6.37 (s, 1H), 6.17 (d, *J* = 16.0 Hz, 1H), 5.97 (br s, 3H), 5.92 (d, *J* = 3.0 Hz, 1H), 5.86 (t, *J* = 4.0 Hz, 1H), 5.77 (dd, *J* = 9.0 Hz, 4.0 Hz, 1H), 5.28 (t, *J* = 9.0 Hz, 1H), 4.30 (dd, *J* = 13.0, 5.5 Hz, 1H), 3.98 (dd, *J* = 12.0 Hz, 3.0 Hz, 1H), 3.78–3.75 (m, 1H), 2.08 (s, 3H), 2.02 (s, 3H), 2.01 (s, 3H), 2.00 (s, 3H). ^13^C NMR (125 MHz, CDCl_3_) δ 170.5, 169.6, 169.5, 169.3, 161.8, 158.2, 153.6, 149.4, 148.2, 141.8, 139.6, 132.0, 124.9, 121.7, 108.4, 107.6, 101.5, 100.0, 97.7, 83.6, 72.4, 68.7, 68.0, 66.0, 61.4, 47.1, 20.6, 20.6. HRMS (ESI-TOF), m/z calcd. C_30_H_31_N_6_O_12_ [M + H]^+^ 667.1994; Found: 667.1921.

### Cell culture

MDA-MB-231, a human breast cancer cell line, was cultured under standard conditions. Specifically, they were grown in Dulbecco’s Modified Eagle’s Medium (DMEM) supplemented with 10% heat-inactivated Fetal Bovine Serum (FBS) and 1% penicillin–streptomycin. The cells were maintained as a monolayer in a 100 mm culture plate and were used for experiments before reaching their 8th passage. Subculturing was performed every third-day using trypsin EDTA treatment. All incubation and maintenance procedures were carried out in a humidified CO_2_ incubator at 37 °C.

### Cell viability assay

To evaluate cell viability, the MTT assay was conducted following standard procedures. After 72 h of cell incubation with or without each compound, cell viability was assessed using MTT (3-(4,5-dimethylthiazol-2-yl)-2,5-diphenyltetrazolium bromide), which is a colorimetric method for determining the number of viable cells in various assays including proliferation, cytotoxicity, or chemosensitivity. The MTT reagent was added to the cells after removal of the medium and incubated for 3 h at 37 °C in the CO_2_ incubator. The formazan product, which is soluble in the tissue culture medium, was dissolved in DMSO, and the absorbance of the formazan product was directly measured at 595 nm using a multimode plate reader without additional processing. The absorbance values are directly proportional to the number of viable cells in culture. The percentage of viable cells in each group was determined relative to the untreated control cells.

### Docking analysis

The docking studies were carried out using various derived triazole-linked pyrazolo[1,5-*a*]pyrimidine-based glycohybrids with proposed binding pocket of X-ray crystallographic structure (Protein Data Bank ID: **3PP0**, resolution: 2.4 Å). Docking was performed using Autodock Vina 4.0, and the interaction between the ligands and protein after docking was visualized and analyzed using PyMol software. The Biovia Discovery Studio Visualizer v20.1.0.19295 was used for 2D visualization and detailed ligand interaction visualization. The Schrödinger Maestro tool was utilized for QSAR and SAR studies.

### Supplementary Information

Supplementary Information.

## Data Availability

All data generated or analysed during this study are included in this published article and its supplementary information files.
