# Supplementary material for: Copper-catalyzed synthesis of pyrazolo[1,5-a]pyrimidine based triazole-linked glycohybrids: mechanistic insights and bio-applications
Source: Sci Rep. 2024 Jan 4;14:529. doi: 10.1038/s41598-023-50202-4 (PMC10766964; doi:10.1038/s41598-023-50202-4)
Supplement: Supplementary file 1 — Supplementary Information. [file 41598_2023_50202_MOESM1_ESM.pdf]

Supplementary information

Copper catalyzed synthesis of pyrazolo[1,5-*a*]pyrimidine based triazole-linked glycohybrids: Mechanistic insights and bio-applications

Ghanshyam Tiwari,[a] Ashish Khanna,[a] Rajdeep Tyagi,[b] Vinay Kumar Mishra,[a] Chintam Narayana,[a] Ram Sagar[*,a,b]

[a]*Department of Chemistry, Institute of Science, Banaras Hindu University, Varanasi – 221005.*

[b]*Glycochemistry Laboratory, School of Physical Sciences, Jawaharlal Nehru University, New Delhi – 110067*

** Corresponding Author*

Email id: ram.sagar@jnu.ac.in

S. No.	Contents	Page No.
1	Synthetic scheme for synthesis of β-keto esters **2a-2i**	S2
2	Experimental method for synthesis of β-keto esters **2a-2i**	S2
3	Spectral data of β-keto esters **2a-2i**	S3-S5
4	Typical method for synthesis of pyrazolo[1,5-*a*]pyrimidine-7-ol derivatives **4a-4i**	S6
5	Spectral data of pyrazolo[1,5-*a*]pyrimidine-7-ol derivatives **4a-4i**	S6-S8
6	Typical method synthesis of propargylated derivatives of pyrazolo[1,5-*a*]pyrimidine-7-ol **6a-6i**	S8
7	Spectral data of propargylated derivatives of pyrazolo[1,5-*a*]pyrimidine-7-ol **6a-6i**	S8-S11
8	Synthetic scheme for synthesis of azido glycosides **8a-8c**	S11
9	Experimental method for synthesis of Glucose, Galactose and Mannose derived azido glycosides **8a-8c**	S11-S12
10	Spectral data of Glucose, Galactose and Mannose derived azido glycosides **8a-8c**	S12
11	Copies of ^{1}H NMR and ^{13}C NMR of selected pyrazolo[1,5-*a*]pyrimidine-7-ol derivatives **4a-4i**	S13- S21
12	Copies of ^{1}H NMR and ^{13}C NMR of propargylated pyrazolo[1,5-*a*]pyrimidine-7-ol **6a-6i**	S22- S30
13	Copies of ^{1}H NMR and ^{13}C NMR of azido glycosides **8a-8c**	S31- S33
14	Copies of ^{1}H NMR and ^{13}C NMR of pyrazolo[1,5-*a*]pyrimidine glycohycohybrids **9a-9i, 10a-10i** & **11a-11i**	S34- S60
15	Results of cell viability assays	S61-62

Scheme S1. Synthesis of β-keto esters 2a-2i

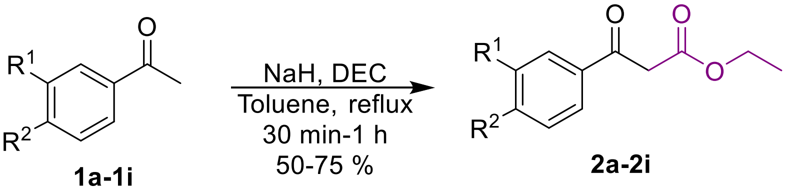

R^1 = H, -C_4H_4- (**2h**), -OCH_2O- **(2i)**
R^2 = CH_3, OCH_3, Br, Cl, F, CF_3, Isopropyl, -C_4H_4-, -OCH_2O-

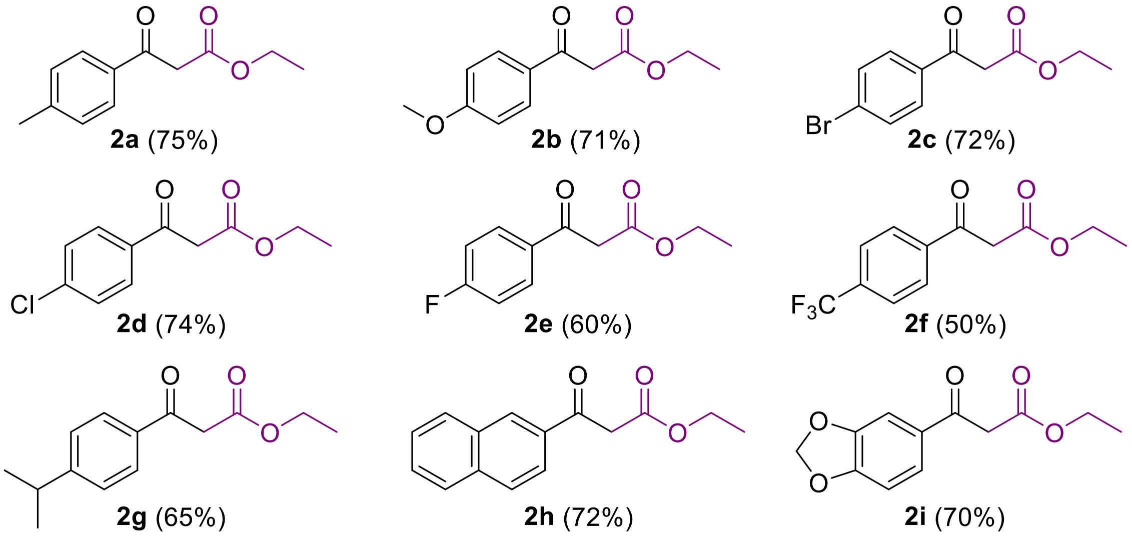

Experimental method for synthesis of β-keto esters 2a-2i:

A toluene solution of diethyl carbonate 1.80 ml (14.9 mmol) and sodium hydride (60% in mineral oil,) 0.54 g (22.35 mmol) were firstly heated to reflux after that a toluene solution of 4-methyl acetophenone 1.0 g (7.45 mmol) was added to the mixture dropwise while staying to the reflux, yellow colored pasty solid will start to appear after 30 min of reflux. The mixture was left to cool at room temperature. After cooling at room temperature, the glacial acetic acid was added dropwise with cold water. Then the mixture was extracted with EtOAc and dried over Na_2SO_4, after evaporation a yellow-colored liquid was obtained, which was purified by column chromatography using 1:9 EtOAc/hexane and yellow colored oil was obtained as desired product **2a** (75 % yield). The same reaction protocol followed to synthesize compounds **2b-2i** using different derivatives of acetophenones **1a-1i** and found moderate to good yields.

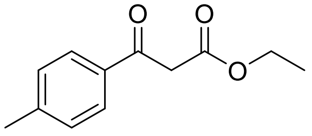

2a

Ethyl 3-oxo-3-(p-tolyl)propanoate **(2a):** yellow colored oil; yield: 1.15 g (75%), Rf = 0.38 (1:9 EtOAc/hexane); ^{1}H-NMR (500 MHz, $CDCl_3$) (keto : enol tautomer = 90:10) δ 12.58 (s, 0.1H, *enolic -OH*), 7.85 (d, *J* = 8.6 Hz, 2H, Ar-*H*), 7.28-7.26 (m, 2H, Ar-*H*), 5.63 (s, 0.1H, *enolic - H*), 4.21 (q, *J* = 7.0 Hz, 2H, -OC$H_2$$CH_3$), 3.97 (s, 2H, -COC$H_2$CO), 2.42 (s, 3H, Ar-C$H_3$), 1.26 (t, *J* = 7.2 Hz, 3H, -CH_2CH_3). ^{13}C-NMR (126 MHz, $CDCl_3$) δ 192.1 (-Ar*CO*CH_2), 167.7 (-CH_2*CO*Et), 144.7, 133.6, 129.4, 129.2, 128.7, 126.1 (Ar-*C*), 86.7 (enolic-*C*-*H*), 61.5, 60.3 (-*C*$H_2$$CH_3$), 45.9 (-CO*C*$H_2$CO), 21.7 (Ar-*C*$H_3$), 14.1 (*C*$H_3$-$CH_2$-) HRMS (ESI-TOF), m/z calcd. $C_{12}H_{15}O_3$ $[M+H]^+$ 207.1016; Found: 207.1043.

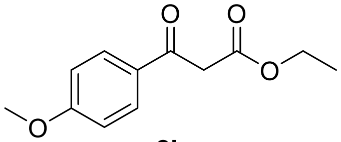

2b

Ethyl 3-(4-methoxyphenyl)-3-oxopropanoate **(2b):** yellow colored oil; yield: 1.05 g (71%), Rf = 0.37 (1:9 EtOAc/hexane); ^{1}H-NMR (500 MHz, $CDCl_3$) (keto : enol tautomer = 95:05) δ 12.63 (s, 0.05H, *enolic - OH*), 7.93 (d, *J* = 8.6 Hz, 2H, Ar-*H*), 6.95 (d, *J* = 8.6 Hz, 2H, Ar-*H*), 5.58 (s, 0.05H, *enolic - H*), 4.21 (q, *J* = 7.3 Hz, 2H, -OC$H_2$$CH_3$), 3.94 (s, 2H, -COC$H_2$CO), 3.86 (d, *J* = 13.4 Hz, 3H, -OCH_3), 1.25 (t, *J* = 7.2 Hz, 3H, -CH_2CH_3). ^{13}C-NMR (126 MHz, $CDCl_3$) δ 191.1 (-Ar*CO*CH_2), 167.8 (-CH_2*CO*Et), 164.1, 131.1, 129.3, 127.9, 114.1 (Ar-*C*), 61.6 (-*C*$H_2$$CH_3$), 55.7 (-O*C*$H_3$), 45.9 (-CO*C*$H_2$CO), 14.2 (*C*$H_3$-$CH_2$-). HRMS (ESI-TOF), m/z calcd. $C_{12}H_{15}O_4$ $[M+H]^+$ 223.0965; Found: 223.0997.

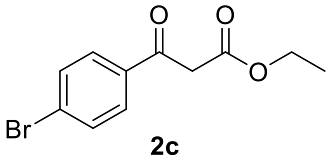

2c

Ethyl 3-(bromophenyl)3-oxopropanoate **(2c):** light brown colored oil; yield: 0.98 g (72%), Rf = 0.40 (1:9 EtOAc/hexane); ^{1}H-NMR (500 MHz, $CDCl_3$) (keto : enol tautomer = 75:25) δ 12.53 (s, 0.25H, *enolic – OH*) 7.81 (d, *J* = 8.6 Hz, 2H, Ar-*H*), 7.65-7.54 (m, 2H, Ar-*H*), 5.64 (s, 0.25H, *enolic- H*), 4.27-4.19 (m, 2H, -OC$H_2$$CH_3$), 3.95 (s, 2H, -COC$H_2$CO), 1.25 (t, *J* = 7.2 Hz, 3H, -CH_2CH_3). ^{13}C-NMR (126 MHz, $CDCl_3$) δ 191.4 (-Ar*CO*CH_2), 167.2 (-CH_2*CO*Et), 134.7, 132.1, 131.9, 130.0, 129.2, 127.7 (Ar-*C*), 87.7 (*enolic-C-H*), 61.7, 60.6 (-*C*$H_2$$CH_3$), 45.8 (-CO*C*$H_2$CO), 14.1, 13.9 (*C*$H_3$-$CH_2$-). HRMS (ESI-TOF), m/z calcd. $C_{11}H_{12}BrO_3$ $[M+H]^+$ 270.9964; Found: 270.9989.

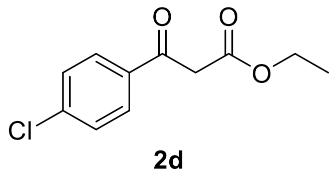

2d

***Ethyl 3-(chlorophenyl)3-oxopropanoate* (2d):** yellow colored oil; yield: 1.09 g (74%), Rf = 0.41 (1:9 EtOAc/hexane); ^{1}H-NMR (500 MHz, $CDCl_3$) (keto : enol tautomer = 75:25) δ 12.570 (s, 0.25H, *enolic – OH)*, 7.89 (d, *J* = 7.6 Hz, 2H, Ar-*H*), 7.71 (d, *J* = 8.6 Hz, 1H, Ar-*H*), 7.42 (dd, *J* = 34.3, 8.6 Hz, 1H, Ar-*H*), 5.63 (s, 0.25H, *enolic H*), 4.24 (q, *J* = 7.2 Hz, 2H, -OC$H_2$$CH_3$), 3.96 (s, 2H, -COC$H_2$CO), 1.29 (dt, *J* = 39.4, 7.2 Hz, 3H, -CH_2CH_3). ^{13}C-NMR (126 MHz, $CDCl_3$) δ 191.3 (-Ar*CO*CH_2), 173.1, 170.2, 167.2 (-CH_2*CO*Et), 140.3, 137.3, 134.3, 131.9, 129.9, 128.8, 127.3 (Ar-*C*), 87.7 (*enolic C-H)*, 61.7, 60.6 (-*C*$H_2$$CH_3$), 45.9 (-CO*C*$H_2$CO), 14.4, 14.2 (*C*$H_3$-$CH_2$-). HRMS (ESI-TOF), m/z calcd. $C_{11}H_{12}ClO_3$ $[M+H]^+$ 227.0469; Found: 227.0499.

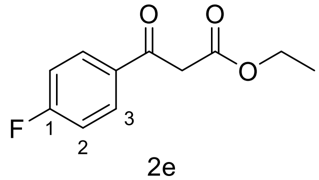

2e

***Ethyl 3-(fluorophenyl)3-oxopropanoate* (2e):** yellow colored oil; yield 0.91 g (60%), Rf = 0.40 (1:9 EtOAc/hexane); ^{1}H-NMR (500 MHz, $CDCl_3$)) (keto : enol tautomer = 85:15) δ 12.6 (s, 0.15H, *enolic-OH*), 7.98 (q, *J* = 4.5 Hz, 2H, Ar-*H*), 7.12 (td, *J* = 18.1, 9.2 Hz, 2H, Ar-*H*), 5.62 (s, 0.15H, *enolic H*), 4.25 (q, *J* = 7.2 Hz, 2H, *CH_2*-CH_3), 3.96 (s, 2H, *CH_2*), 1.33 (t, *J* = 7.2 Hz, 3H, CH_2-*CH_3*). ^{13}C-NMR (126 MHz, $CDCl_3$) δ 191.1 (-Ar*CO*CH_2), 167.4 (-CH_2*CO*Et), 167.1, 165.5, 163.5, 132.5, 131.3, 131.2, 129.6, 128.2, 116.0, 115.8 (Ar-*C*), 87.2 (enolic *C-H*), 61.5 (*CH_2*-CH_3), 45.9 (CO*CH$_2$*CO), 14.2 (CH_2-*CH_3*). ^{13}C – ^{19}F Couplings in ^{13}C NMR (126 MHz, $CDCl_3$) δ 164.58 (d, $J_{C\text{-}F}$ = 253.26 Hz, C_1), 131.28 (d, $J_{C\text{-}F}$ = 10.08 Hz, C_3), 115.95 (d, $J_{C\text{-}F}$ = 22.68 Hz, C_2). HRMS (ESI-TOF), m/z calcd. $C_{11}H_{12}FO_3$ $[M+H]^+$ 211.0765; Found: 211.0791.

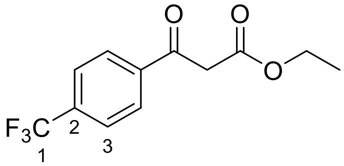

2f

***Ethyl 3-oxo-3-(4-(trifluoromethyl)phenyl)propanoate* (2f):** yellow colored oil; yield: 0.69 g (50%), Rf = 0.44 (1:9 EtOAc/hexane); ^{1}H-NMR (500 MHz, $CDCl_3$) (keto : enol tautomer = 55:45) δ 12.57 (s, 0.45H, enolic -*OH*), 8.06 (d, *J* = 8.6 Hz, 2H, Ar-*H*), 7.88 (d, *J* = 8.6 Hz, 0.9H, Ar-*H*), 7.76 (d, *J* = 8.6 Hz, 2H, Ar-*H*), 7.68 (d, *J* = 7.6 Hz, 0.9H, Ar-H), 5.72 (s, 0.45H, *enolic- H*), 4.25 (dq, *J* = 32.4, 7.3 Hz, 2H, -OC*H$_2$*CH_3), 4.02 (s, 6H, -COC*H$_2$*CO, enolic), 1.35 (t, *J* = 7.2 Hz, 3H, -CH_2C*H$_3$*), 1.30-1.25 (m, 1.35H, -CH_2C*H$_3$* (*enolic*)). ^{13}C-NMR (126 MHz, $CDCl_3$) δ 191.5 (-Ar*CO*CH_2), 172.7, 169.6, 167.1 (-CH_2*CO*Et), 138.7, 136.9, 135.4, 135.2, 134.9, 134.6, 134.4, 128.9, 126.3, 125.9, 125.7, 125.5, 124.8, 124.5, 122.8, 122.4 (Ar-*C*), 89.1 (*enolic-C-H*), 61.8, 60.8 (-*C*$H_2$$CH_3$), 46.2 (-CO*C*$H_2$CO), 29.7, 14.4, 14.1 (*C*H_3-CH_2-). ^{13}C – ^{19}F Couplings in ^{13}C NMR (126 MHz, $CDCl_3$) δ 134.8 (q, $J_{C\text{-}F}$ = 32.76 Hz, C_2), 125.72 (d, $J_{C\text{-}F}$ = 3.78 Hz, C_3), 123.32

(q, $J_{C\text{-}F}$ = 273.42 Hz, C_1). HRMS (ESI-TOF), m/z calcd. $C_{12}H_{12}F_3O_3$ $[M+H]^+$ 261.0733; Found: 261.0759.

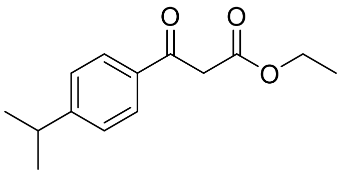

2g

***Ethyl 3-(isopropylphenyl)3-oxopropanoate* (2g):** light yellow colored oil; yield: 0.93 g (65%), Rf = 0.40 (1:9 EtOAc/hexane); ^{1}H-NMR (500 MHz, $CDCl_3$) (keto : enol tautomer = 77:23) δ 12.57 (s, 0.23H, *enolic OH*), 7.88 (d, *J* = 7.6 Hz, 2H, Ar-*H*), 7.33 (d, *J* = 8.6 Hz, 2H, Ar-*H*), 5.64 (s, 0.23H, *enolic -H*), 4.23 (td, *J* = 15.3, 7.6 Hz, 2H, -OC*H*$_2$CH$_3$), 3.97 (s, 2H, -COC*H*$_2$CO), 2.96 (q, *J* = 7.0 Hz, 1H, Ar-C*H*-(CH$_3$)$_2$), 1.26 (t, *J* = 7.6 Hz, 9H, Ar-CH-(C*H*$_3$)$_2$, -CH$_2$C*H*$_3$). ^{13}C-NMR (126 MHz, $CDCl_3$) δ 192.3 (-Ar*CO*CH$_2$), 167.7 (-CH$_2$*CO*Et), 155.5, 134.1, 128.9, 126.9, 126.7, 126.1 (Ar-*C*), 86.7 (*enolic-C-H)*, 61.5, 60.3 (-*C*H$_2$CH$_3$), 46.1 (-CO*C*H$_2$CO), 34.4, 34.1 (-*C*H-(CH$_3$)$_2$), 23.7, 23.6 (-CH-(*C*H$_3$)$_2$), 14.4, 14.1 (*C*H$_3$-CH$_2$-). HRMS (ESI-TOF), m/z calcd. $C_{14}H_{19}O_3$ $[M+H]^+$ 235.1329; Found: 235.1349.

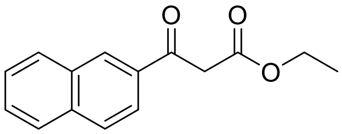

2h

***Ethyl 3-(naphthalene-2-yl)-3-oxopropanoate* (2h):** yellow colored oil; yield: 1.02 g (72%), Rf = 0.39 (1:9 EtOAc/hexane); ^{1}H-NMR (500 MHz, $CDCl_3$) (keto : enol tautomer = 85:15) δ 12.67 (s, 0.15H, *enolic - OH*), 8.46 (s, 1H, Ar-*H*), 8.03-7.85 (m, 4H, Ar-*H*), 7.64-7.54 (m, 2H, Ar-*H*), 5.81 (s, 0.15H, *enolic-H*), 4.23 (q, *J* = 7.0 Hz, 2H, -OC*H*$_2$CH$_3$), 4.12 (s, 2H, -COC*H*$_2$CO), 2.07 (d, *J* = 25.7 Hz, 1H), 1.26 (t, *J* = 6.7 Hz, 3H, -CH$_2$C*H*$_3$). ^{13}C-NMR (126 MHz, $CDCl_3$) δ 192.6 (-Ar*CO*CH$_2$), 167.7 (-CH$_2$*CO*Et), 135.9, 133.5, 132.6, 130.8, 129.8, 129.2, 129.04, 128.8, 128.4, 128.0, 127.8, 127.6, 127.1, 126.9, 124.0, 122.7 (Ar-*C*), 88.0 (enolic-*C*-H), 61.7, 60.5 (-*C*H$_2$CH$_3$), 46.2 (-CO*C*H$_2$CO), 14.4, 14.2 (*C*H$_3$-CH$_2$-). HRMS (ESI-TOF), m/z calcd $C_{15}H_{15}O_3$ $[M+H]^+$ 243.1016; Found: 243.1037.

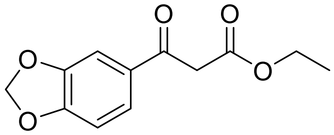

2i

***Ethyl 3-(benzo[d][1,3]dioxol-5-yl)-3-oxopropanoate*(2i):** light yellow colored oil; yield: 1 g (70%), Rf = 0.38 (1:9 EtOAc/hexane); ^{1}H-NMR (500 MHz, $CDCl_3$) (keto : enol tautomer = 93:7) δ 12.61 (s, 0.07H, *enolic -OH*), 7.51 (dd, *J* = 7.6, 1.9 Hz, 1H, Ar-*H*), 7.40 (d, *J* = 1.9 Hz, 1H, Ar-*H*), 6.84 (d, *J* = 8.6 Hz, 1H, Ar-*H*), 6.04 (s, 2H, -O-C*H*$_2$-O-), 5.52 (s, 0.07H, *enolic -H*), 4.19 (q, *J* = 7.0 Hz, 2H, -OC*H*$_2$CH$_3$), 3.89 (s, 2H, -COC*H*$_2$CO), 1.24 (t, *J* = 7.2 Hz, 3H, -CH$_2$C*H*$_3$). ^{13}C-NMR (126 MHz, $CDCl_3$) δ 190.7, 167.8 (-CH$_2$*CO*Et), 152.5 (Ar-*C*-O-CH$_2$-O-), 148.5 (Ar-*C*-O-CH$_2$-O-), 131.0, 125.3, 121.2, 108.2, 108.1, 106.4 (Ar-*C*), 102.2, 101.8 (O-*C*H$_2$-

O), 86.4 (*enolic* – *C*-H), 61.6, 60.4 (-*C*H_2CH_3), 46.0 (-CO*C*H_2CO), 14.4, 14.2 (*C*H_3-CH_2-). HRMS (ESI-TOF), m/z calcd. $C_{12}H_{13}O_5$ $[M+H]^+$ 237.0757; Found: 237.0779.

Typical method for synthesis of pyrazolo[1,5-*a*]pyrimidine-7-ol derivatives 4a-4i:

3-amino pyrazole (**3**) 0.5 g (6.017 mmol) was taken in an oven dried 100 ml round bottom flask and dissolved in AcOH (20 ml) under N_2 atmosphere. After that, ethyl 3-oxo-3-(*p*-tolyl)propanoate **(2a)** 1.49 g (7.22 mmol) was added to the reaction mixture and then it was heated to reflux at 118 °C for 12-14 h. The solvent was evaporated from the reaction mixture, using toluene as azeotropic solvent, after that it was triturated with diethyl ether. Finally, it was dried under high vacuum to afford solid product **4a** with 60% yield, which was used as in further reactions without any purification. The similar protocol followed to synthesize compounds **4b-4i,** which give moderate to good yields.

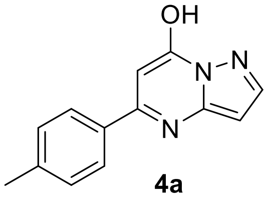

4a

***5-(p-tolyl)pyrazolo[1,5-a]pyrimidin-7-ol* (4a):** light brown colored solid; yield 0.81 g (60%), Rf = 0.26 (1:9 MeOH/DCM); ^{1}H NMR (500 MHz, DMSO-*d*$_6$) δ 12.48 (s, 1H), 7.89 (s, 1H), 7.73 (d, *J* = 7.6 Hz, 2H), 7.39 (d, *J* = 8.6 Hz, 2H), 6.22 (s, 1H), 6.03 (s, 1H), 2.39 (s, 3H); ^{13}C NMR (126 MHz, DMSO-*d*$_6$) δ 156.6, 149.8, 143.1, 141.9, 141.3, 129.7, 129.4, 127.1, 93.1, 89.6, 20.9. HRMS (ESI-TOF), m/z calcd. $C_{13}H_{11}N_3O$ $[M+H]^+$ 226.0975; Found: 226.0993.

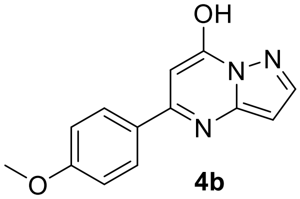

4b

***5-(4-methoxyphenyl)pyrazolo[1,5-a]pyrimidin-7-ol* (4b):** white colored solid; yield 0.81 g (56%), Rf = 0.25 (1:9 MeOH/DCM); ^{1}H NMR (500 MHz, DMSO-*d*$_6$) δ 12.37 (s, 1H), 7.88 (s, 1H), 7.81 (d, *J* = 8.0 Hz, 2H), 7.13 (d, *J* = 9.3 Hz, 2H), 6.20 (s, 1H), 6.01 (s, 1H), 3.85 (s, 3H); ^{13}C NMR (126 MHz, DMSO-*d*$_6$) δ 161.7, 156.6, 149.5, 143.0, 142.0, 128.9, 124.3, 114.6, 92.6, 89.5, 55.6. HRMS (ESI-TOF), m/z calcd. $C_{13}H_{12}N_3O_2$ $[M+H]^+$ 242.0924; Found: 242.0939.

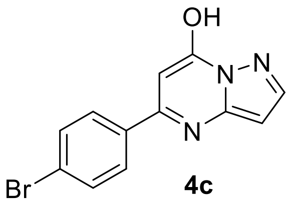

4c

***5-(4-bromophenyl)pyrazolo[1,5-a]pyrimidin-7-ol* (4c):** light brown colored solid; yield 0.87 g (50%), Rf = 0.26 (1:9 MeOH/DCM); ^{1}H NMR (500 MHz, DMSO-*d*$_6$) δ 12.57 (s, 1H), 7.91 (s, 1H), 7.80 (s, 4H), 6.23 (s, 1H), 6.09 (s, 1H); ^{13}C NMR (126 MHz, DMSO-*d*$_6$) δ 156.3, 148.6, 143.2, 141.8, 132.0, 131.4, 129.3, 124.7, 93.8, 89.6. HRMS (ESI-TOF), m/z calcd. $C_{12}H_9BrN_3O$ $[M+H]^+$ 289.9924; Found: 289.9943.

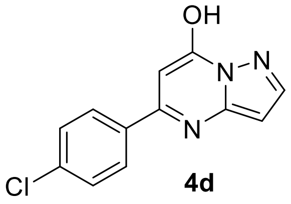

4d

***5-(4-chlorophenyl)pyrazolo[1,5-a]pyrimidin-7-ol* (4d):** light brown colored solid; yield 0.85 g (58%), Rf = 0.27 (1:9 MeOH/DCM); ^{1}H NMR (500 MHz, DMSO-d_6) δ 12.85 (s, 1H), 7.91 (m, 3H), 7.64 (d, J = 8.0 Hz, 2H), 6.27 (d, J = 4.0 Hz, 1H), 6.09 (s, 1H). ^{13}C NMR (126 MHz, DMSO-d_6) δ 156.4, 148.6, 143.1, 141.9, 135.8, 131.0, 129.2, 129.0, 93.7, 89.7. HRMS (ESI-TOF), m/z calcd. $C_{12}H_9ClN_3O$ [M+H]$^+$ 246.0429; Found: 246.0451.

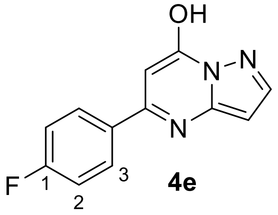

4e

***5-(4-fluorophenyl)pyrazolo[1,5-a]pyrimidin-7-ol* (4e):** brown colored solid; yield: 0.63 (46%), Rf = 0.24 (1:9 MeOH/DCM); ^{1}H NMR (500 MHz, DMSO-d_6) δ 12.53 (s, 1H), 7.91 (m, 3H), 7.43 (dd, J = 8.7 Hz, 2H), 6.22 (s, 1H), 6.05 (s, 1H); ^{13}C NMR (126 MHz, DMSO-d_6) δ 164.7, 162.7, 156.4, 148.7, 143.1, 141.8, 129.8, 129.7, 128.7, 116.1, 116.0, 93.6, 89.5. ^{13}C-^{19}F couplings in ^{13}C NMR (126 MHz, DMSO-d_6) δ 163.7 (d, $J_{C\text{-}F}$ = 249.48 Hz, C_1), 129.8 (d, $J_{C\text{-}F}$ = 8.82 Hz, C_3), 116.0 (d, $J_{C\text{-}F}$ = 21.42 Hz, C_2). HRMS (ESI-TOF), m/z calcd. $C_{12}H_9FN_3O$ [M+H]$^+$ 230.0724; Found: 230.0751.

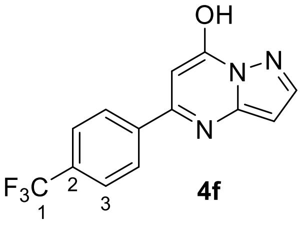

4f

***5-(4-(trifluoromethyl)phenyl)pyrazolo[1,5-a]pyrimidine-7-ol* (4f):** light brown colored solid; yield: 0.67 g (40%), Rf = 0.26 (1:9 MeOH/DCM); ^{1}H NMR (500 MHz, DMSO-d_6) δ 12.69 (s, 1H), 8.07 (d, J = 8.0 Hz, 2H), 7.98 – 7.92 (m, 3H), 6.26 (s, 1H), 6.16 (s, 1H); ^{13}C NMR (126 MHz, DMSO-d_6) δ 156.30, 148.22, 143.26, 141.85, 136.28, 131.33, 131.08, 130.82, 130.57, 128.31, 125.90, 125.88, 124.93, 122.76, 94.68, 89.72. ^{13}C-^{19}F couplings in ^{13}C NMR (126 MHz, DMSO-d_6) δ 130.95 (q, $J_{C\text{-}F}$ = 32.76 Hz, C_2), 125.89 (d, $J_{C\text{-}F}$ = 2.52 Hz, C_3), 123.84 (q, $J_{C\text{-}F}$ = 273.42 Hz, C_1). HRMS (ESI-TOF), m/z calcd. $C_{13}H_9F_3N_3O$ [M+H]$^+$ 280.0692; Found: 280.0704.

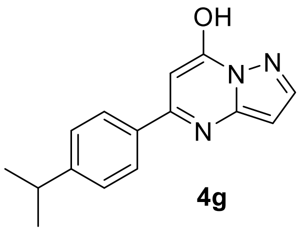

4g

***5-(4-isopropylphenyl)pyrazolo[1,5-a]pyrimidin-7-ol* (4g):** light brown colored solid; yield: 0.73 g (48%), Rf = 0.26 (1:9 MeOH/DCM); ^{1}H NMR (500 MHz, DMSO-d_6) δ 12.44 (s, 1H), 7.89 (s, 1H), 7.77 (d, J = 7.9 Hz, 2H), 7.45 (d, J = 7.9 Hz, 2H), 6.21 (s, 1H), 6.04 (s, 1H), 2.98 (h, J = 6.7 Hz, 1H), 1.24 (d, J = 6.8 Hz, 6H); ^{13}C NMR (126 MHz, DMSO-

d$_6$) δ 156.49, 151.85, 149.71, 143.04, 141.88, 129.79, 127.28, 127.05, 93.16, 89.48, 33.32, 23.61. HRMS (ESI-TOF), m/z calcd. $C_{15}H_{16}N_3O$ $[M+H]^+$ 254.1288; Found: 254.1307.

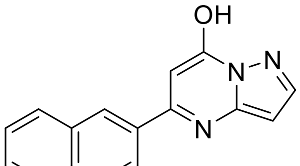

4h

***5-(naphthalen-2-yl)pyrazolo[1,5-a]pyrimidin-7-ol* (4h):** brown or gray colored solid; yield: 0.92 g (59 %), Rf = 0.26 (1:9 MeOH/DCM); ^{1}H NMR (500 MHz, DMSO-*d*$_6$) δ 12.66 (s, 1H), 8.48 (s, 1H), 8.12 (d, *J* = 8.0 Hz, 1H), 8.09 – 8.06 (m, 1H), 8.05 – 8.01 (m, 1H), 7.94 (d, *J* = 5.3 Hz, 2H), 7.67 – 7.63 (m, 2H), 6.27 (s, 1H), 6.23 (s, 1H); ^{13}C NMR (126 MHz, DMSO-*d*$_6$) δ 156.84, 150.03, 143.51, 142.39, 134.18, 132.80, 129.96, 129.14, 129.07, 128.20, 128.08, 127.63, 127.52, 124.42, 94.29, 89.97. HRMS (ESI-TOF), m/z calcd. $C_{16}H_{12}N_3O$ $[M+H]^+$ 262.0975; Found: 262.0996.

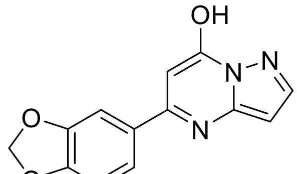

4i

***5-(benzo[d][1,3]dioxol-5yl)pyrazolo[1,5-a]pyrimidin-7-ol* (4i):** white colored solid; yield 0.87 g (57%), Rf = 0.25 (1:9 MeOH/DCM); ^{1}H NMR (500 MHz, DMSO-*d*$_6$) δ 12.39 (s, 1H), 7.88 (s, 1H), 7.42 (s, 1H), 7.38 (d, *J* = 8.6 Hz, 1H), 7.11 (d, *J* = 7.6 Hz, 1H), 6.20 (s, 1H), 6.14 (s, 2H), 6.01 (s, 1H); ^{13}C NMR (126 MHz, DMSO-*d*$_6$) δ 156.73, 149.96, 149.59, 148.26, 143.31, 142.02, 126.20, 122.14, 108.97, 107.61, 102.21, 93.20, 89.70. HRMS (ESI-TOF), m/z calcd. $C_{13}H_{10}N_3O_3$ $[M+H]^+$ 256.0717; Found: 256.0741.

Typical method for synthesis of propargylated derivatives of pyrazolo[1,5-*a*]pyrimidine-7-ol 6a-6i: In an oven dried 100 ml two necked round bottom flask taken 0.5 g (2.221 mmol) of 5-(*p*-tolyl)pyrazolo[1,5-*a*]pyrimidin-7-ol **(4a)** in dry DMF 15 ml then K_2CO_3 0.36 g (2.65 mmol) was added and stirred for half an hour. After that propargyl bromide 0.22 ml (2.65 mmol) was added dropwise and reaction mixture refluxed for 2h. The completion of reaction was monitored by TLC, after completion of reaction the reaction mixture was quenched by aqueous solution of $NaHCO_3$ and extracted with EtOAc. The organic layer dried over Na_2SO_4 and the solvent from the mixture was evaporated under vacuum. After that the crude mixture was subjected for column chromatography to afford purified solid product **6a**. The similar protocol followed to synthesize compounds **6b-6i,** which give moderate to good yields.

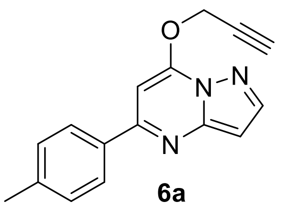

6a

7-(prop-2-yn-1-yloxy)-5-(p-tolyl)pyrazolo[1,5-a]pyrimidine **(6a)**: light yellow colored solid; yield 0.46 g (80%), Rf = 0.42 (EtOAc); ^{1}H NMR ^{1}H (500 MHz, $CDCl_3$) δ 7.89 (d, *J* = 8.0 Hz, 2H), 7.76 (d, *J* = 3.8 Hz, 1H), 7.27 (d, *J* = 7.8 Hz, 2H), 6.57 (s, 1H), 6.52 (d, *J* = 4.0 Hz, 1H), 5.56 (s, 2H), 2.47 (s, 1H), 2.41 (s, 3H); ^{13}C NMR (126 MHz, $CDCl_3$) δ 162.51, 158.25, 154.40, 140.42, 140.13, 134.87, 129.51, 127.23, 101.86, 99.13, 76.40, 75.01, 41.97, 21.44. HRMS (ESI-TOF), m/z calcd. $C_{16}H_{13}N_3O$ $[M+H]^+$ 264.1131; Found: 264.1157.

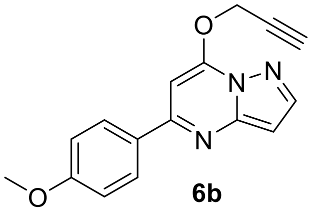

6b

5-(4-methoxyphenyl)-7-(prop-2-yn-1-yloxy)pyrazolo[1,5 a]pyrimidine **(6b):** light yellow colored solid; yield 0.44 g (77%), Rf = 0.4 (EtOAc); ^{1}H NMR (500 MHz, $CDCl_3$) ^{1}H NMR (500 MHz, CHLOROFORM-*D*) δ 7.90 (d, *J* = 8.1 Hz, 2H), 7.74 (d, *J* = 3.9 Hz, 1H), 6.93 (d, *J* = 8.0 Hz, 2H), 6.47 (s, 1H), 6.46 (d, *J* = 5.2 Hz, 1H), 5.51 (s, 2H), 3.81 (s, 3H), 2.44 (s, 1H); ^{13}C NMR (126 MHz, $CDCl_3$) δ 162.08, 161.38, 158.21, 154.28, 140.23, 129.94, 128.75, 114.05, 101.63, 98.30, 76.35, 74.95, 55.38, 41.92. HRMS (ESI-TOF), m/z calcd. $C_{16}H_{14}N_3O_2$ $[M+H]^+$ 280.1081; Found: 280.1198.

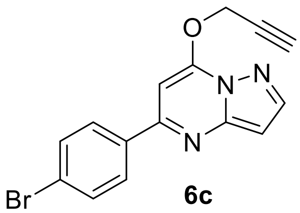

6c

5-(4-bromophenyl)-7-(prop-2-yn-1-yloxy)pyrazolo[1,5 a]pyrimidine **(6c):** white colored solid; yield 0.41 g (71%), Rf = 0.42 (EtOAc); ^{1}H NMR (500 MHz, $CDCl_3$) δ 7.85 (d, *J* = 9.3 Hz, 2H), 7.79 (d, *J* = 3.9 Hz, 1H), 7.58 (d, *J* = 8.0 Hz, 2H), 6.53 (s, 1H), 6.52 (d, *J* = 2.5 Hz, 1H), 5.58 (s, 2H), 2.48 (s, 1H); ^{13}C NMR (126 MHz, $CDCl_3$) δ 161.31, 158.11, 154.43, 140.15, 136.68, 131.97, 128.93, 124.79, 101.80, 99.40, 76.60, 74.90, 42.13. HRMS (ESI-TOF), m/z calcd. $C_{15}H_{11}BrN_3O$ $[M+H]^+$ 328.0080; Found: 328.0102.

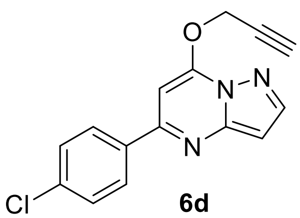

6d

5-(4-chlorophenyl)-7-(prop-2-yn-1-yloxy)pyrazolo[1,5 a]pyrimidine **(6d):** light yellow colored solid; yield 0.41 g (75%), Rf = 0.43 (EtOAc); ^{1}H NMR (500 MHz, $CDCl_3$) δ 7.92 (d, *J* = 8.0 Hz, 2H), 7.79 (d, *J* = 3.9 Hz, 1H), 7.42 (d, *J* = 8.9 Hz, 2H), 6.53 (s, 1H), 6.52 (d, *J* = 5 Hz, 1H), 5.58 (s, 2H), 2.48 (s, 1H); ^{13}C NMR (126 MHz, $CDCl_3$) δ 161.28, 158.12, 154.44, 140.16, 136.38, 136.22, 129.02, 128.68, 101.81, 99.43, 76.59, 74.90, 42.12. HRMS (ESI-TOF), m/z calcd. $C_{15}H_{11}ClN_3O$ $[M+H]^+$ 284.0585; Found: 284.0603.

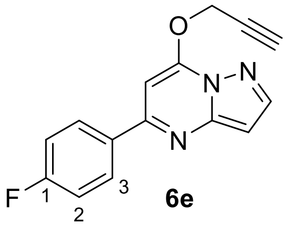

6e

***5-(4-fluorophenyl)-7-(prop-2-yn-1-yloxy)pyrazolo[1,5 a]pyrimidine* (6e):** white colored solid; yield 0.41 g (71%), Rf = 0.43 (EtOAc); ^{1}H NMR (500 MHz, $CDCl_3$) δ 7.98 (dd, *J* = 7.4 Hz, 2H), 7.78 (s, 1H), 7.14 (d, *J* = 8.6 Hz, 2H), 6.52 (s, 1H), 6.52 (d, *J* = 2.5 Hz, 1H), 5.58 (s, 2H), 2.47 (s, 1H); ^{13}C NMR (126 MHz, $CDCl_3$) δ 165.23, 163.25, 161.57, 158.21, 154.37, 140.26, 133.79, 129.39, 129.32, 115.86, 115.69, 101.68, 99.19, 76.57, 74.85, 42.11. ^{13}C-^{19}F couplings in ^{13}C NMR (126 MHz, DMSO-d_6) δ 164.24 (d, $J_{C\text{-}F}$ = 249.4 Hz, C_1), 129.35 (d, $J_{C\text{-}F}$ = 8.82 Hz, C_3), 115.77 (d, $J_{C\text{-}F}$ = 21.42 Hz, C_2). HRMS (ESI-TOF), m/z calcd. $C_{15}H_{11}FN_3O$ $[M+H]^+$ 268.0881; Found: 268.0901.

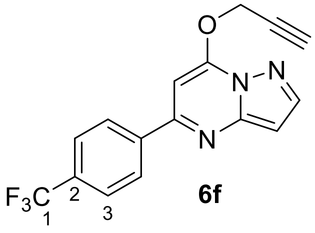

6f

***5-(4-(trifluoromethyl)phenyl)-7-(prop-2-yn-1-yloxy)pyrazolo[1,5 a]pyrimidine* (6f):** yellow colored crystalline solid; yield 0.36 g (65%), Rf = 0.43 (EtOAc); ^{1}H NMR (500 MHz, $CDCl_3$) δ 8.08 (d, *J* = 8.0 Hz, 2H), 7.81 (d, *J* = 3.9 Hz, 1H), 7.70 (d, *J* = 8.0 Hz, 2H), 6.57 (s, 1H), 6.53 (d, *J* = 4.0 Hz, 1H), 5.59 (s, 2H), 2.49 (s, 1H); ^{13}C NMR (126 MHz, $CDCl_3$) δ 160.85, 158.01, 154.47, 141.22, 140.15, 132.23, 131.98, 131.71, 131.46, 127.69, 125.70, 125.22, 123.05, 101.75, 100.08, 76.65, 74.85, 42.14. ^{13}C-^{19}F couplings in ^{13}C NMR (126 MHz, DMSO-d_6) δ 131.84 (q, $J_{C\text{-}F}$ = 34.02 Hz, C_2), 125.68 (d, $J_{C\text{-}F}$ = 3.78 Hz, C_3), 124.13 (q, $J_{C\text{-}F}$ = 273.42 Hz, C_1). HRMS (ESI-TOF), m/z calcd. $C_{16}H_{11}F_3N_3O$ $[M+H]^+$ 318.0849; Found: 318.0873.

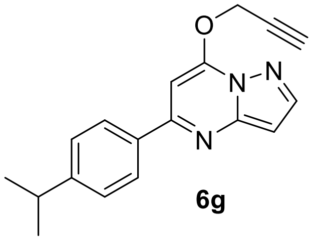

6g

***5-(4-isopropylphenyl)-7-(prop-2-yn-1-yloxy)pyrazolo[1,5 a]pyrimidine* (6g):** light yellow colored solid; yield 0.39 g (68%), Rf = 0.42 (EtOAc); ^{1}H NMR (500 MHz, $CDCl_3$) δ 7.89 (d, *J* = 8.0 Hz, 2H), 7.76 (d, *J* = 4.0 Hz, 1H), 7.31 (d, *J* = 8.2 Hz, 2H), 6.55 (s, 1H), 6.52 (d, *J* = 3.8 Hz, 1H), 5.55 (s, 2H), 2.94 (hept, *J* = 6.7 Hz, 1H), 2.46 (s, 1H), 1.26 (d, *J* = 6.7 Hz, 6H); ^{13}C NMR (126 MHz, $CDCl_3$) δ 162.69, 158.34, 154.40, 151.38, 140.15, 135.23, 127.39, 126.91, 101.82, 99.20, 76.45, 74.97, 42.02, 34.08, 23.92. HRMS (ESI-TOF), m/z calcd. $C_{18}H_{18}N_3O$ $[M+H]^+$ 292.1444; Found: 292.1469.

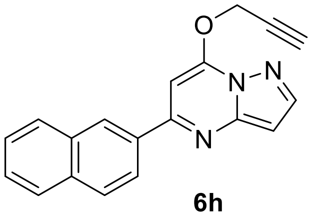

6h

***5-(naphthalen-2-yl)-7-(prop-2-yn-1-yloxy)pyrazolo[1,5-a]pyrimidine* (6h):** yellow colored solid; yield 0.42 g (74%), Rf = 0.4 (EtOAc); ^{1}H NMR (500 MHz, DMSO-d_6) δ 8.71 (s, 1H), 8.41 (d, *J* = 3.9 Hz, 1H), 8.21 (d, *J* = 8.3 Hz, 1H), 8.09 – 8.04 (m, 1H), 8.00 (d, *J* = 8.2 Hz, 1H), 7.98 – 7.93 (m, 1H), 7.60 – 7.53 (m, 2H), 6.80 (s, 1H), 6.77 (d, *J* = 3.7

Hz, 1H), 5.60 (s, 2H), 3.47 (s, 1H); ^{13}C NMR (126 MHz, DMSO-d_6) δ 160.58, 156.94, 154.38, 143.27, 134.45, 133.69, 132.80, 128.88, 128.12, 127.52, 127.15, 126.92, 126.52, 124.33, 101.49, 98.23, 77.85, 76.39, 41.32. HRMS (ESI-TOF), m/z calcd. $C_{19}H_{14}N_3O$ $[M+H]^+$ 300.1131; Found: 300.1154.

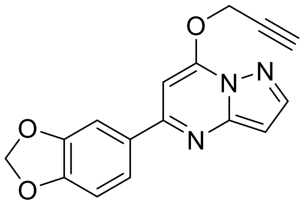

6i

5-(benzo[d][1,3]dioxol-5-yl)-7-(prop-2-yn-1-yloxy)pyrazolo[1,5-*a*]pyrimidine (6i): yellow colored solid; yield 0.43 g (75%) Rf = 0.4 (EtOAc); ^{1}H NMR (500 MHz, $CDCl_3$) δ 7.75 (s, 1H), 7.48 (d, *J* = 8.3 Hz, 1H), 7.44 (s, 1H), 6.84 (d, *J* = 8.1 Hz, 1H), 6.47 (s, 1H), 6.43 (s, 1H), 5.98 (s, 2H), 5.52 (s, 2H), 2.46 (s, 1H); ^{13}C NMR (126 MHz, $CDCl_3$) δ 161.96, 158.19, 154.20, 149.45, 148.21, 140.22, 131.85, 121.85, 108.43, 107.59, 101.65, 101.54, 98.64, 76.43, 74.93, 41.98. HRMS (ESI-TOF), m/z calcd. $C_{16}H_{12}N_3O_3$ $[M+H]^+$ 294.0873; Found: 294.0895.

Scheme S2. Synthetic scheme for synthesis of azido glycosides 8a-8c

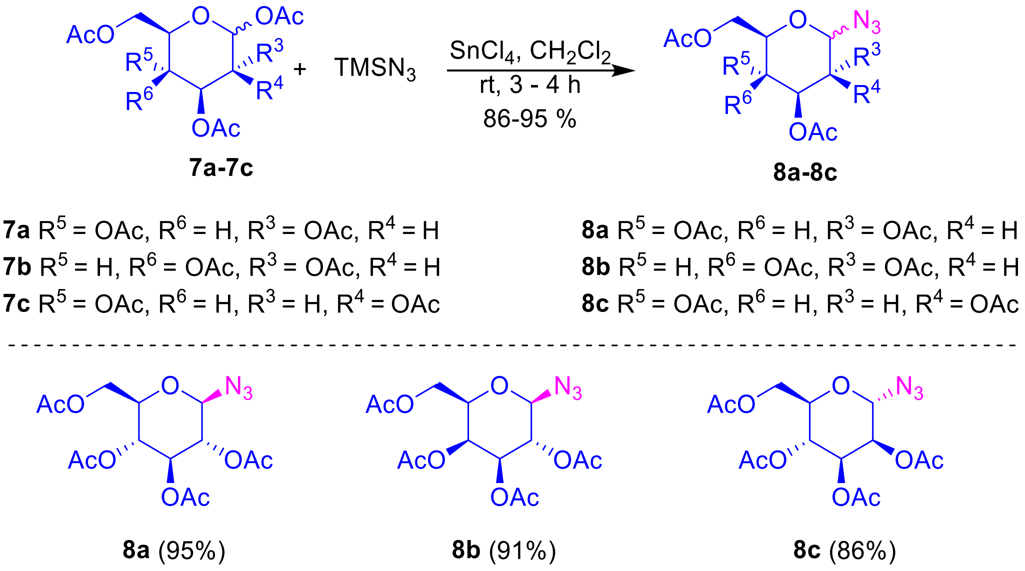

Experimental method for synthesis of Glucose, Galactose and Mannose derived azido glycosides 8a-8c: In this experiment, 5 grams (12.82 mmol) of 1,2,3,4,6-penta-O-acetyl-β-D-glucopyranose **7a** was taken in an oven-dried round-bottom flask. Anhydrous dichloromethane was added to the flask, which was then placed under a nitrogen atmosphere. Next, $SnCl_4$ was added dropwise (1.29 mL, 11.02 mmol) at an ice bath and stirred for 30 minutes. $TMSN_3$ was added next (1.91 mL, 16.66 mmol) at room temperature, and the stirring was continued for 3-4 hours. The

reaction was monitored using TLC until completion. After the reaction was complete, the mixture was quenched with ice-cold water and an aqueous solution of $NaHCO_3$ was added. The mixture was extracted with dichloromethane, and the organic layer was washed with a brine solution. The obtained mixture was dried using anhydrous Na_2SO_4 and the solvent was evaporated under vacuum to obtain a crude residue. The crude residue was purified by column chromatography, which resulted in the isolation of a white-colored solid azido glycoside **8a** in 95 % yield. The same reaction procedure was used to synthesize glycosides **8b** and **8c**, starting with 1,2,3,4,6-penta-*O*-acetyl-β-D-galactopyranose and 1,2,3,4,6-penta-*O*-acetyl-β-D-mannopyranose, respectively.

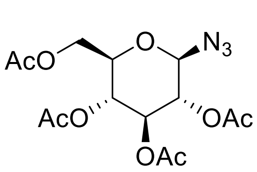

8a

***(2R,3R,4S,5R,6R)-2-(acetoxymethyl)-6-azidotetrahydro-2H-pyran-3,4,5-triyl triacetate* (8a):** white colored solid; yield: 4.54 g (95%), Rf = 0.30 (3:7 EtOAc/hexane); ^{1}H NMR (500 MHz, $CDCl_3$) δ 5.20 (t, *J* = 9.5 Hz, 1H), 5.08 (t, *J* = 9.5 Hz, 1H), 4.93 (t, *J* = 9.1 Hz, 1H), 4.63 (d, *J* = 8.6 Hz, 1H), 4.25 (dd, *J* = 12.4, 4.8 Hz, 1H), 4.15 (d, *J* = 12.4 Hz, 1H), 3.82 – 3.75 (m, 1H), 2.08 (s, 3H), 2.06 (s, 3H), 2.01 (s, 3H), 1.99 (s, 3H); ^{13}C NMR (126 MHz, $CDCl_3$) δ 170.73, 170.23, 169.42, 169.32, 87.99, 74.09, 72.68, 70.71, 67.95, 61.74, 20.79, 20.64. HRMS (ESI-TOF), m/z calcd. $C_{14}H_{19}N_3O_9Na$ $[M+Na]^+$ 396.1014; Found: 396.1038.

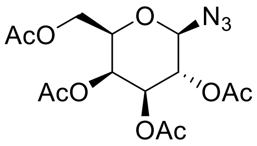

8b

***(2R,3S,4S,5R,6R)-2-(acetoxymethyl)-6-azidotetrahydro-2H-pyran-3,4,5-triyl triacetate* (8b):** white colored solid; yield: 4.35 g (91%), Rf = 0.30 (3:7 EtOAc/hexane); ^{1}H NMR (500 MHz, $CDCl_3$) δ 5.40 (d, *J* = 4.0 Hz, 1H), 5.14 (t, *J* = 10.0 Hz, 1H), 5.04 – 4.99 (m, 1H), 4.58 (d, *J* = 9.3 Hz, 1H), 4.13 (dt, *J* = 12.0, 6.7 Hz, 2H), 4.00 (t, *J* = 6.7 Hz, 1H), 2.15 (s, 3H), 2.07 (s, 3H), 2.04 (s, 3H), 1.96 (s, 3H); ^{13}C NMR (126 MHz, $CDCl_3$) δ 170.44, 170.19, 170.05, 169.44, 88.38, 72.96, 70.82, 68.18, 66.97, 61.32, 20.73, 20.67, 20.58. HRMS (ESI-TOF), m/z calcd. $C_{14}H_{19}N_3O_9Na$ $[M+Na]^+$ 396.1014; Found: 396.1041.

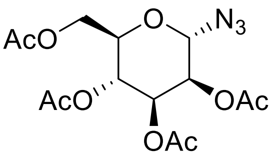

8c

***(2R,3R,4S,5S,6S)-2-(acetoxymethyl)-6-azidotetrahydro-2H-pyran-3,4,5-triyl triacetate* (8c):** white colored sticky solid; yield: 4.11 g (86%), Rf = 0.30 (3:7 EtOAc/hexane); ^{1}H NMR (500 MHz, $CDCl_3$) δ 5.34 (s, 1H), 5.26 – 5.16 (m, 2H), 5.11 – 5.08 (m, 1H), 4.25 (dd, *J* = 13.3, 5.3 Hz, 1H), 4.13 – 4.07 (m, 2H), 2.11 (s, 3H), 2.05 (s, 3H), 2.00 (s, 3H), 1.93 (s, 3H).^{13}C NMR (126 MHz, $CDCl_3$) δ 170.54, 169.80, 169.71, 169.61, 87.46, 70.64, 69.15, 68.25, 65.61, 62.13, 20.75, 20.66, 20.62, 20.55. HRMS (ESI-TOF), m/z calcd. $C_{14}H_{19}N_3O_9Na$ $[M+Na]^+$ 396.1014; Found: 396.1030.

Copies of ^{1}H NMR and ^{13}C NMR of selected pyrazolo[1,5-*a*]pyrimidine-7-ol derivatives 4a-4i: ^{1}H-NMR (500 MHz, DMSO-d_6)

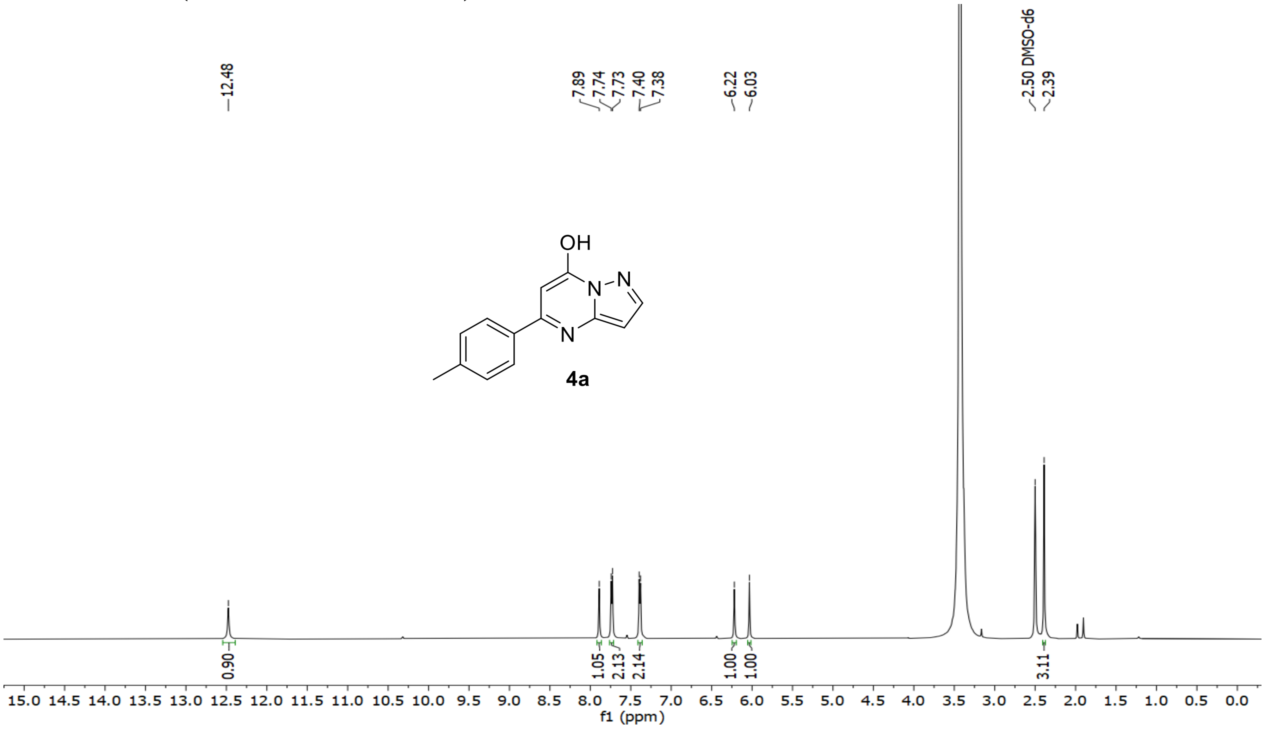

^{13}C NMR (126 MHz, DMSO-d_6)

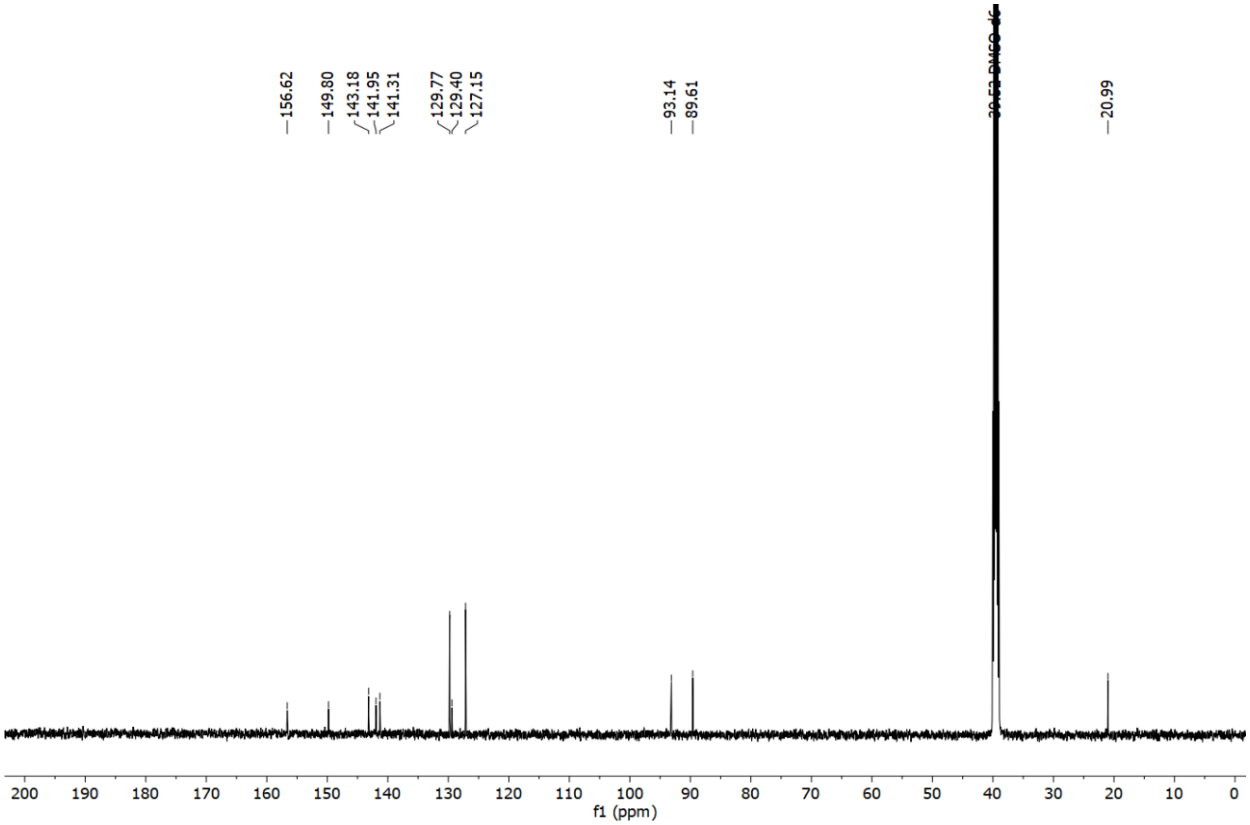

^{1}H-NMR (500 MHz, DMSO-d_6)

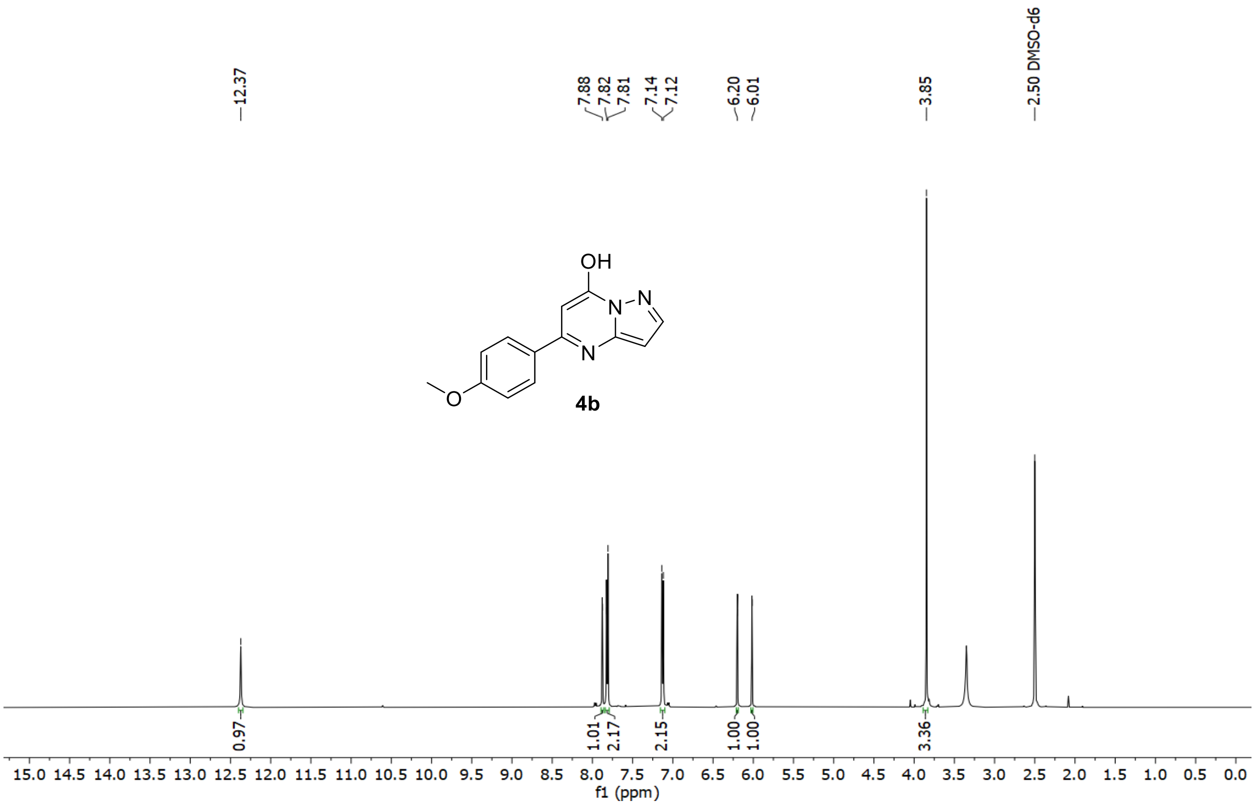

^{13}C NMR (126 MHz, DMSO-d_6)

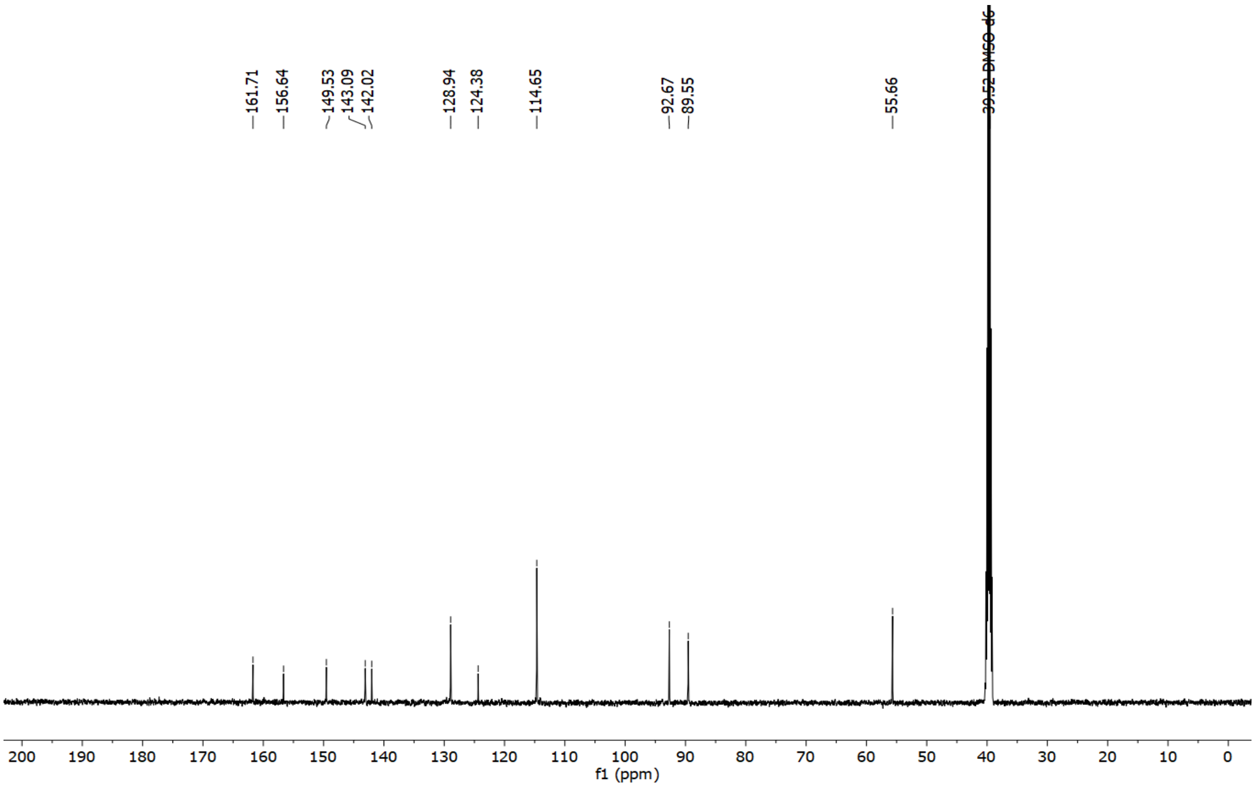

^{1}H-NMR (500 MHz, DMSO-d_6)

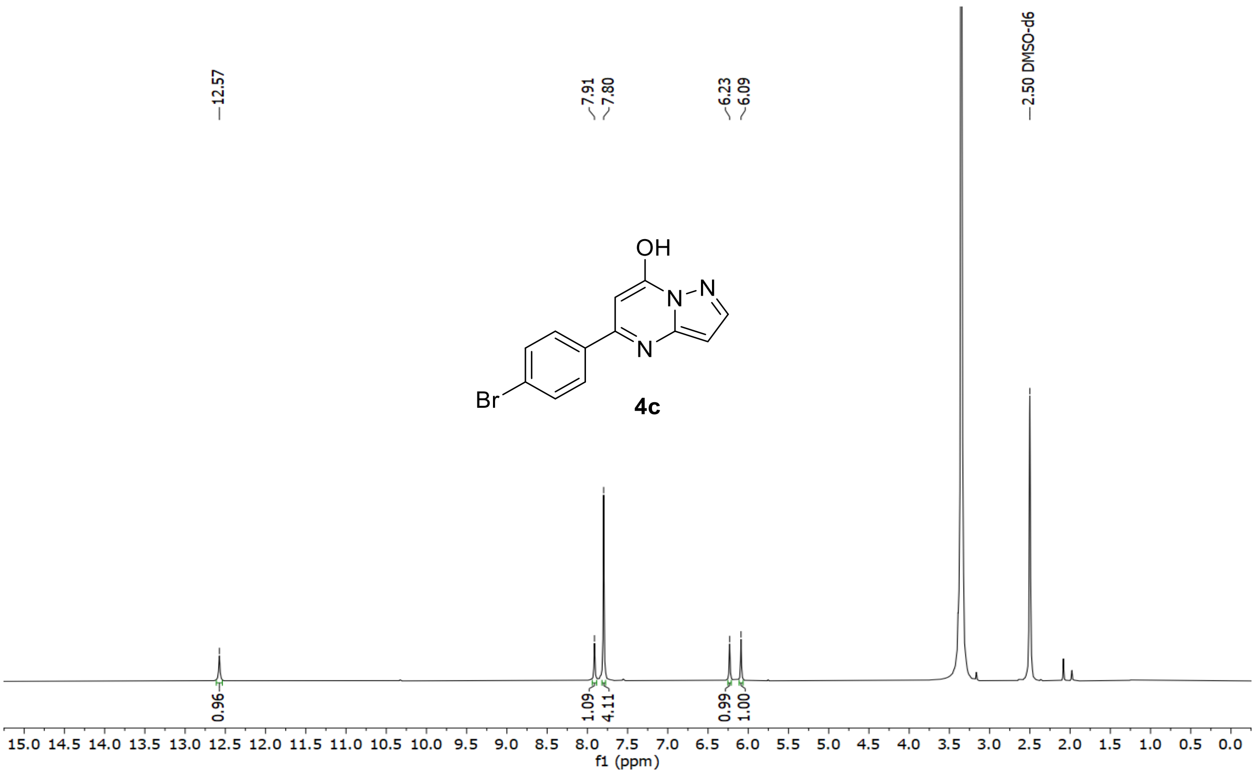

^{13}C NMR (126 MHz, DMSO-d_6)

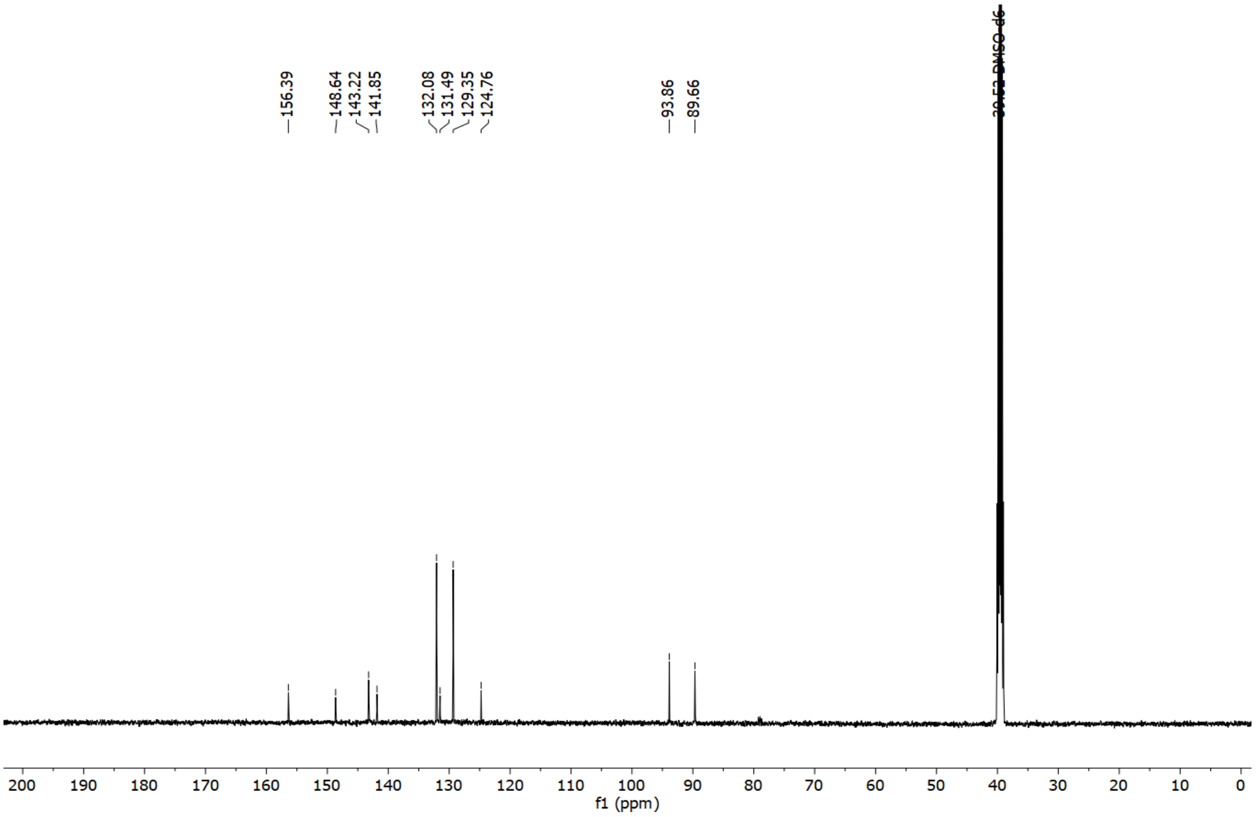

^{1}H-NMR (500 MHz, DMSO-d_6)

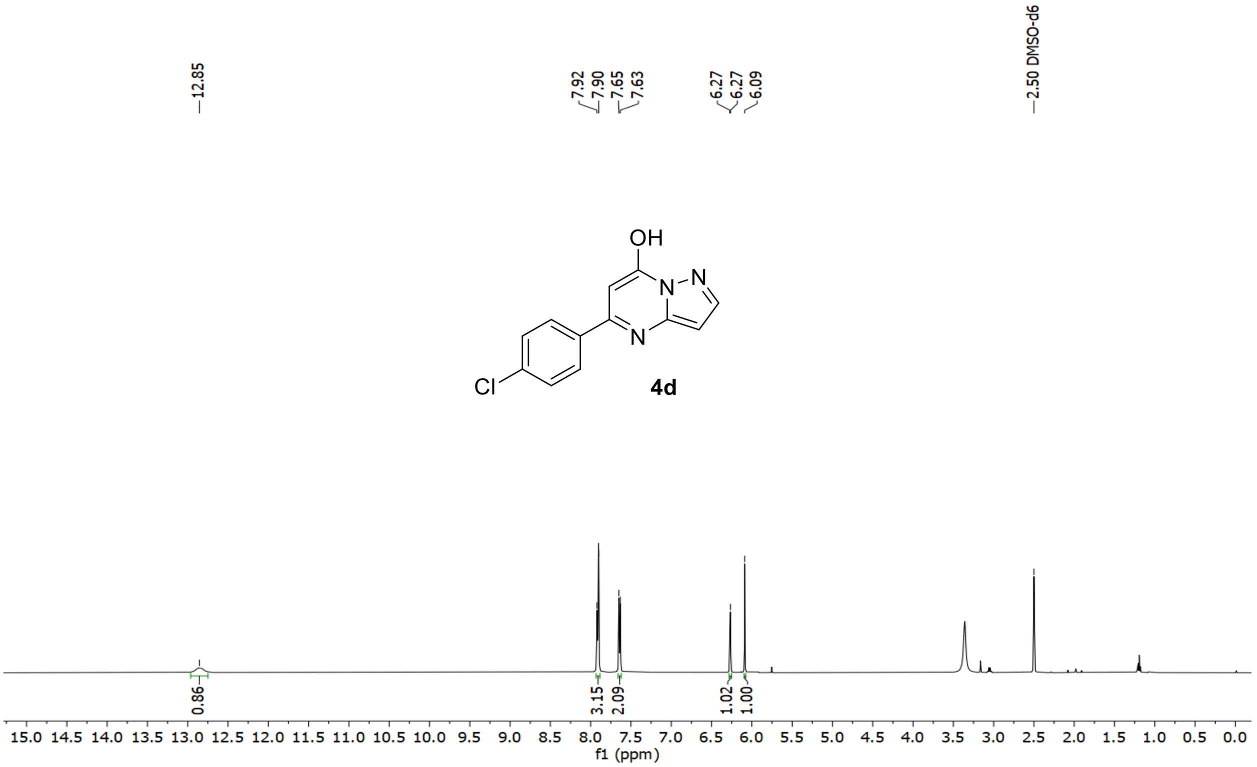

^{13}C NMR (126 MHz, DMSO-d_6)

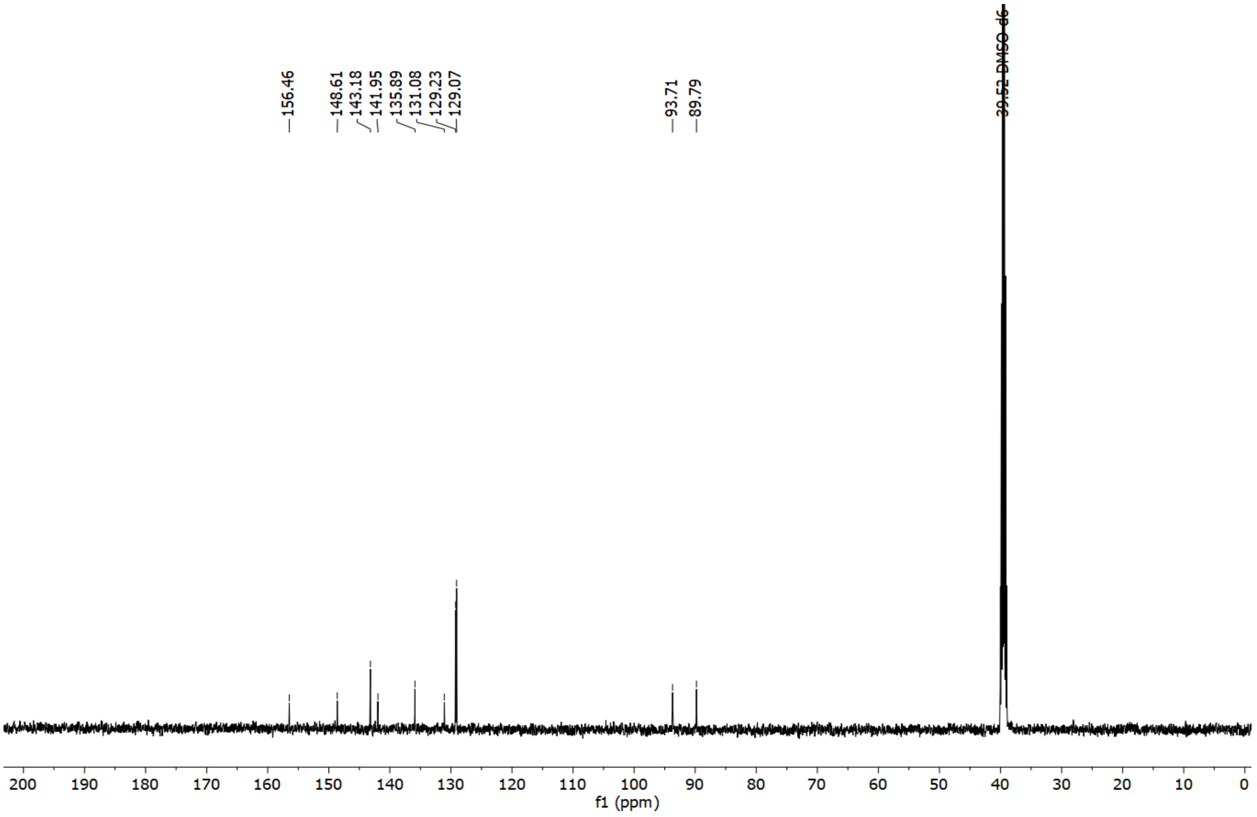

^{1}H-NMR (500 MHz, DMSO-*d*$_6$)

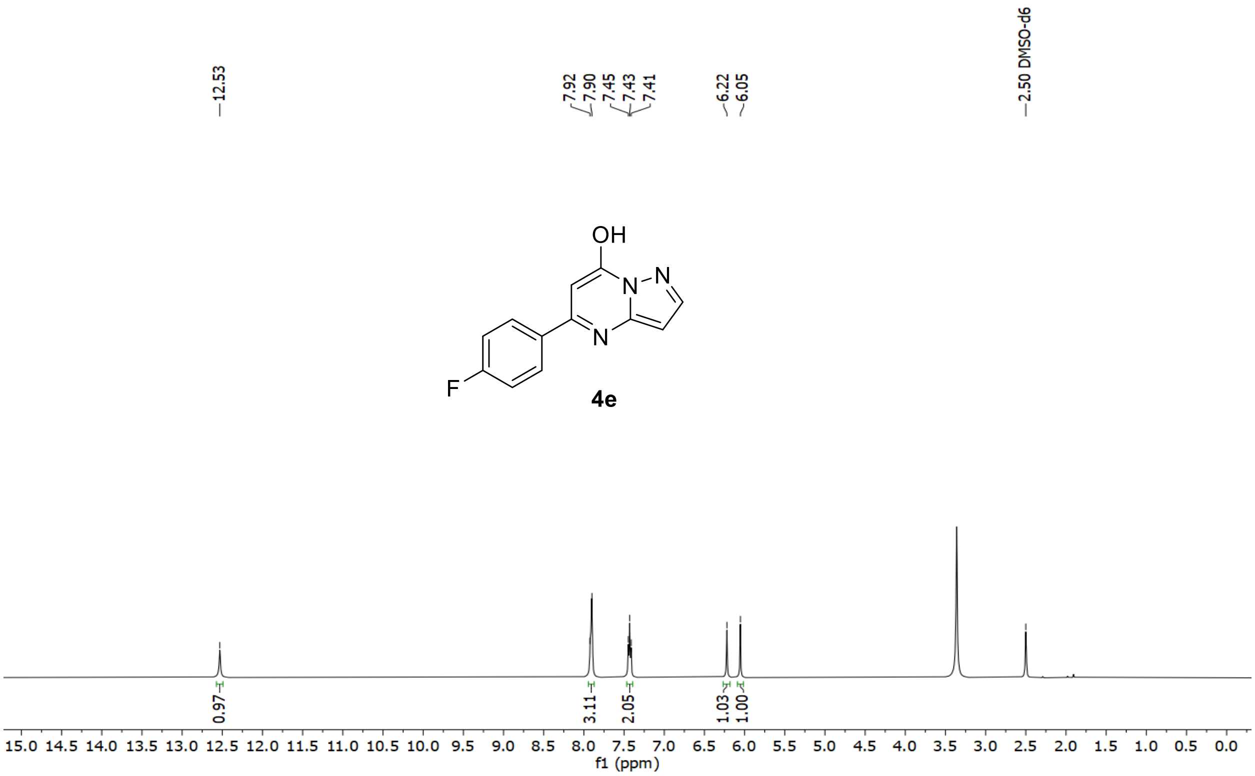

^{13}C NMR (126 MHz, DMSO-*d*$_6$)

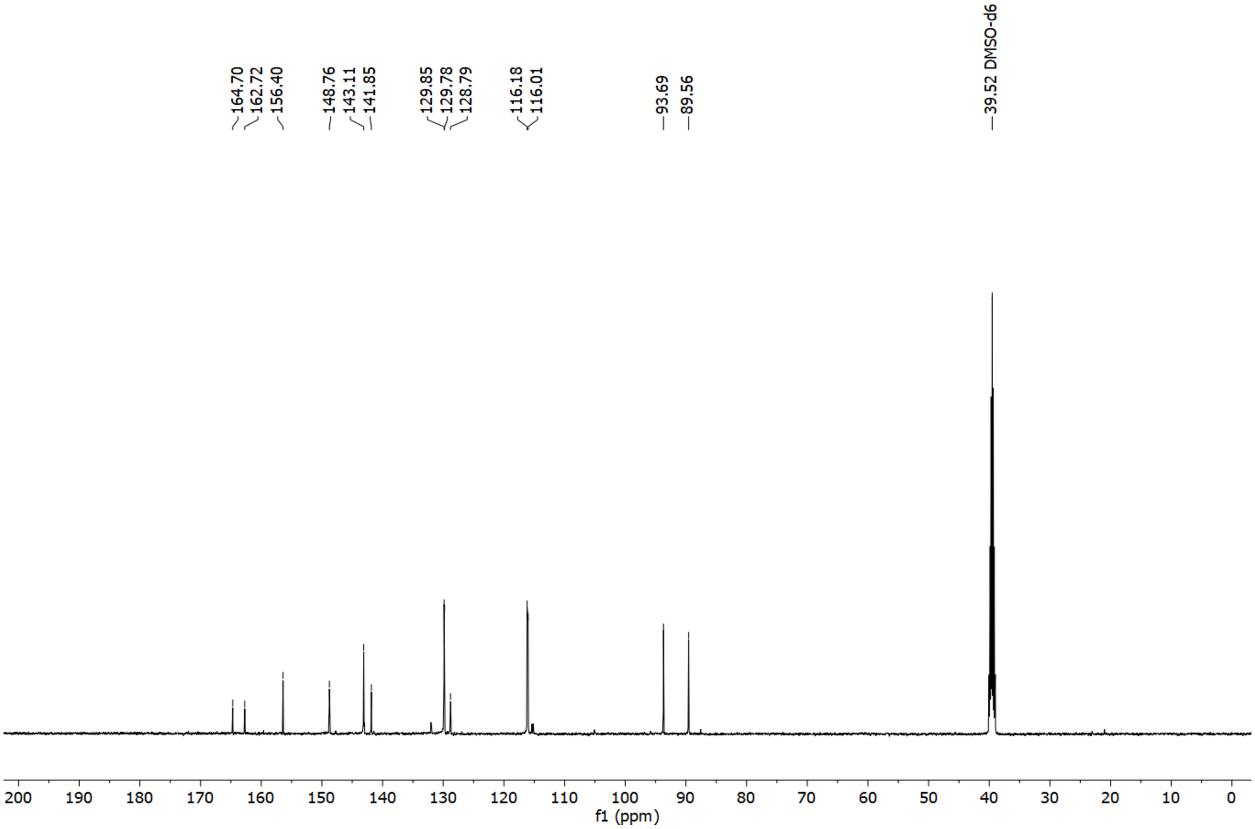

^{1}H-NMR (500 MHz, DMSO-d_6)

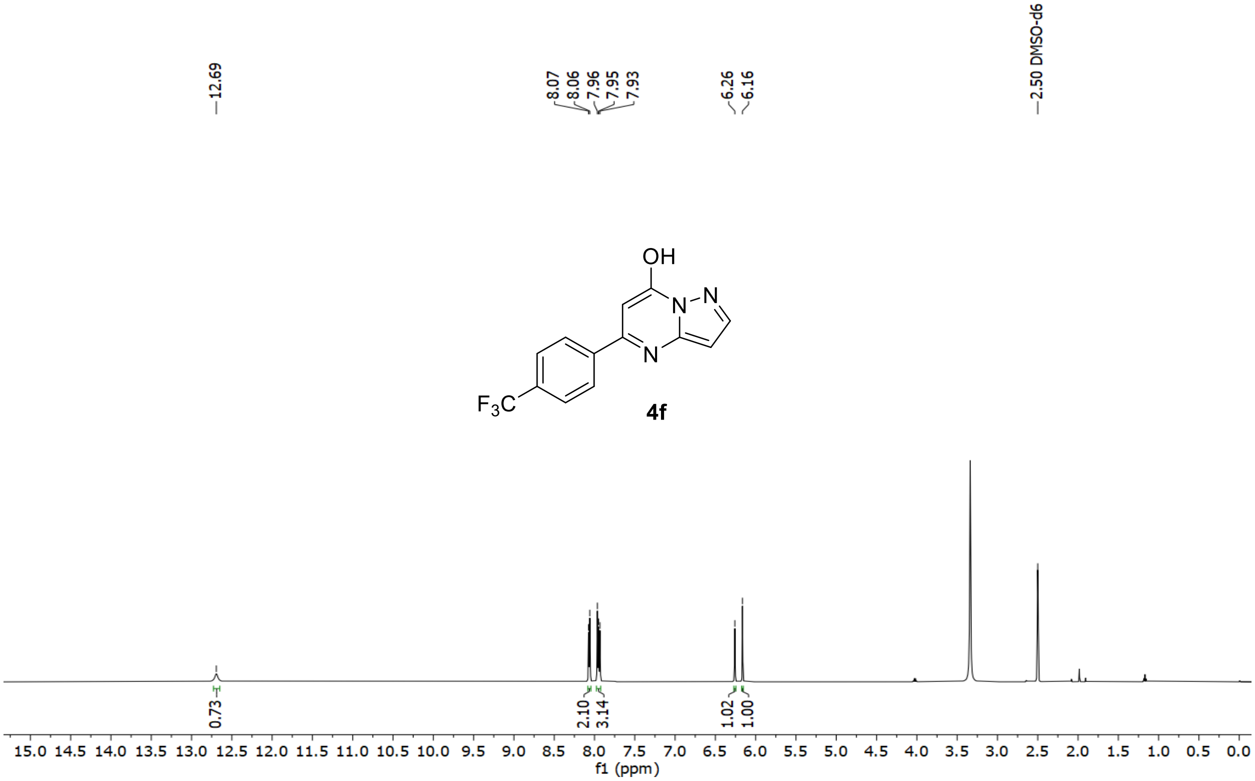

^{13}C NMR (126 MHz, DMSO-d_6)

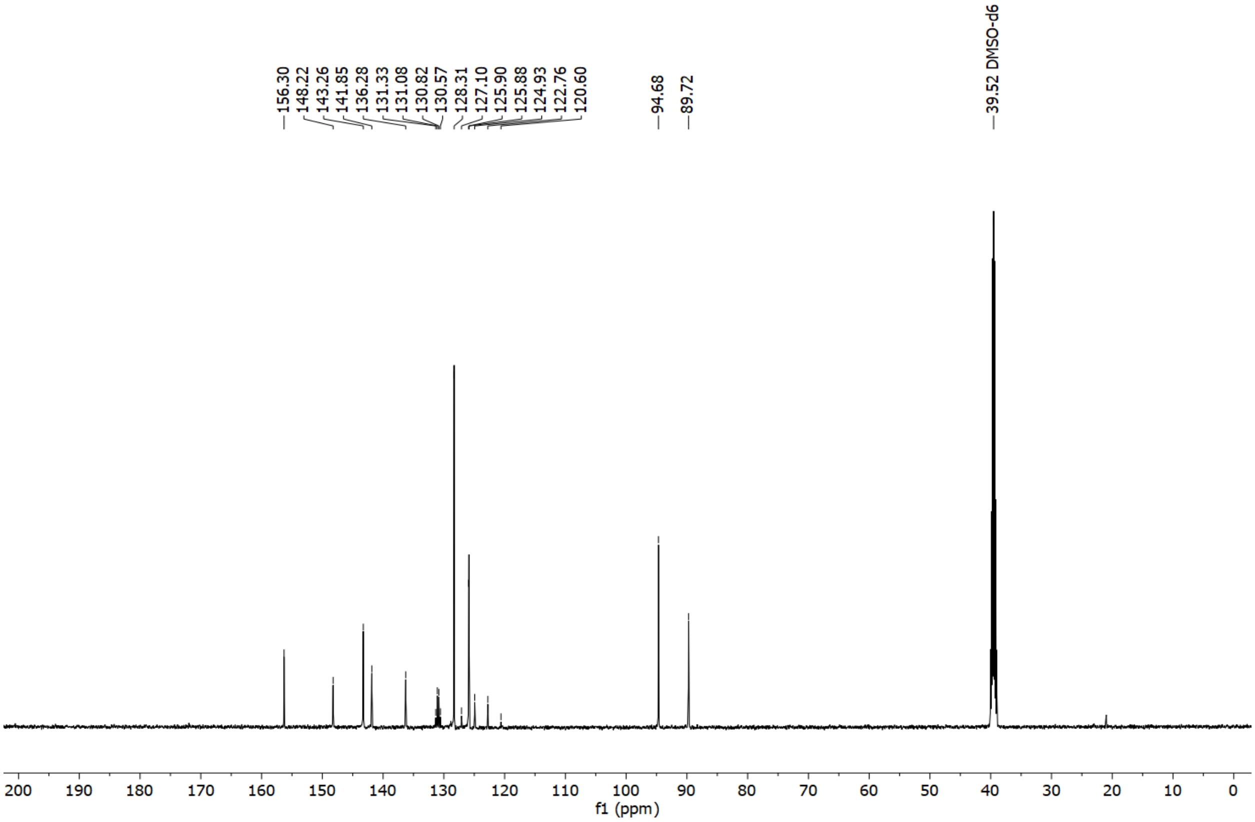

^{1}H-NMR (500 MHz, DMSO-d_6)

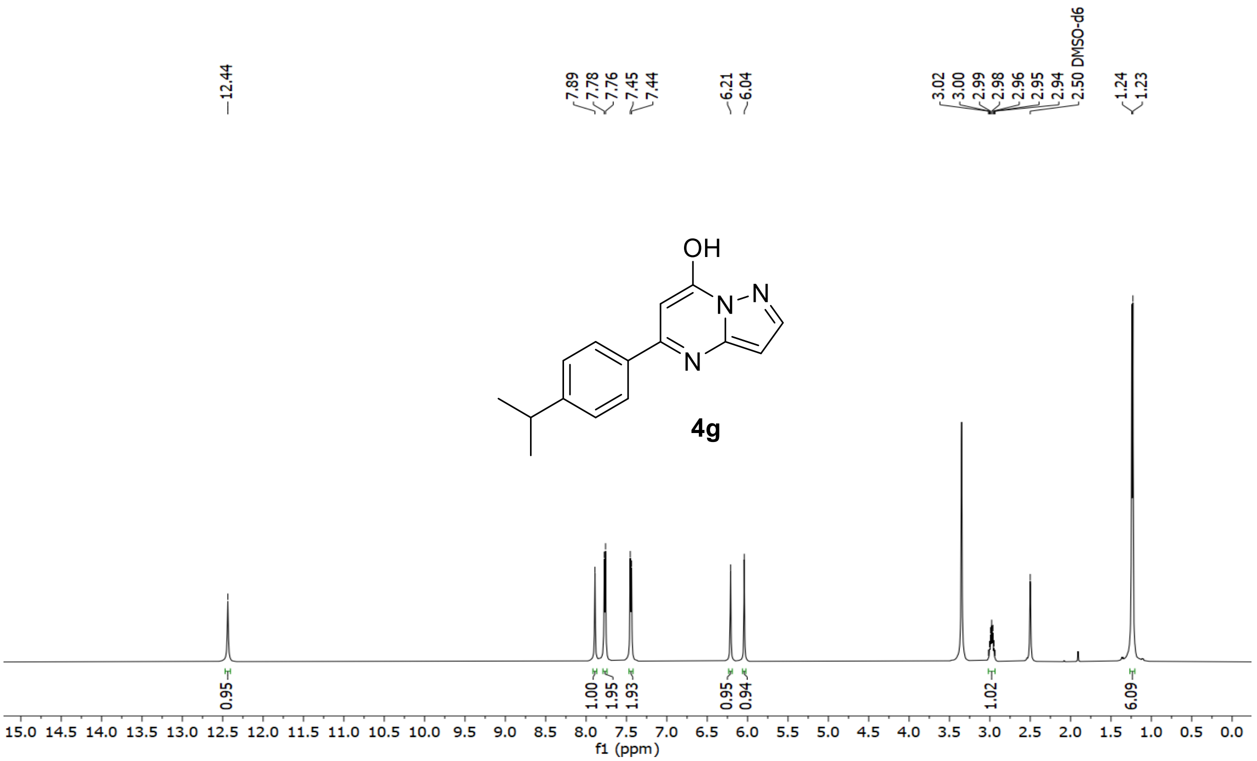

^{13}C NMR (126 MHz, DMSO-d_6)

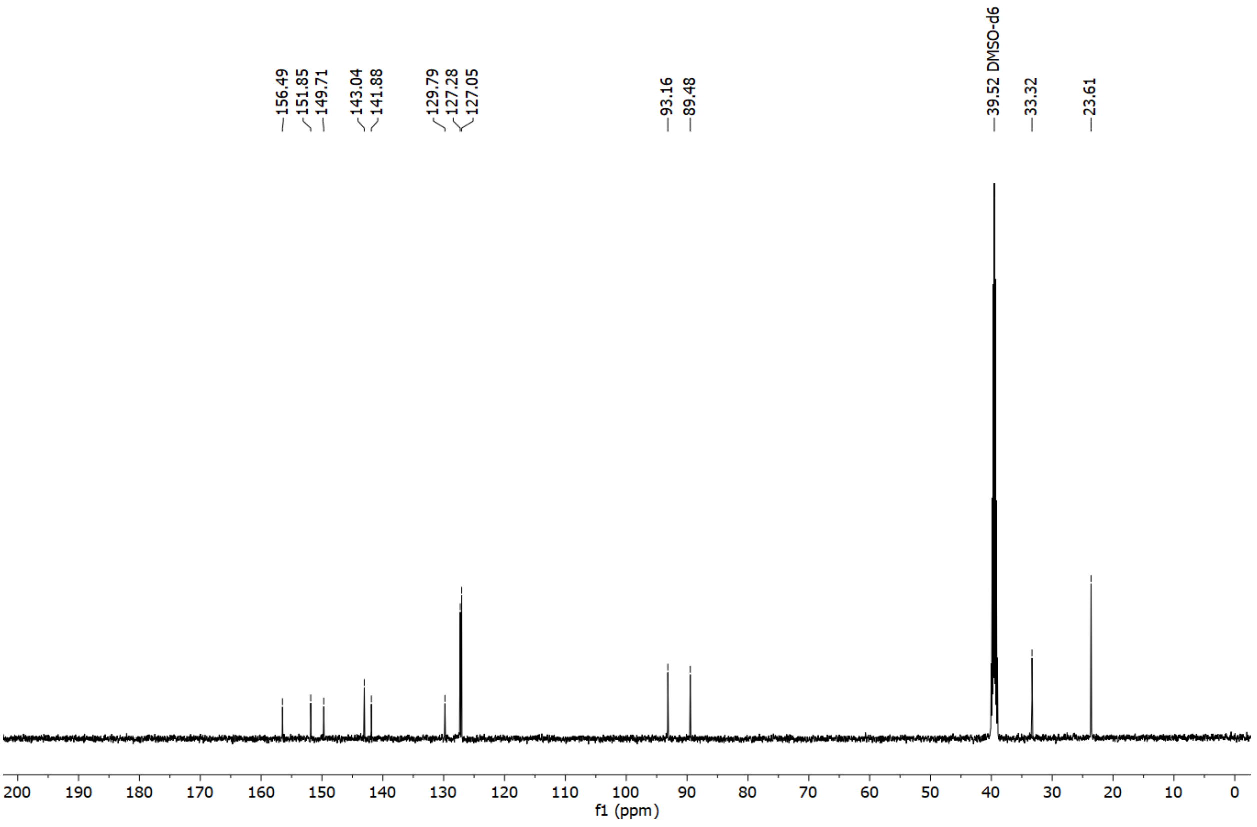

^{1}H-NMR (500 MHz, DMSO-d_6)

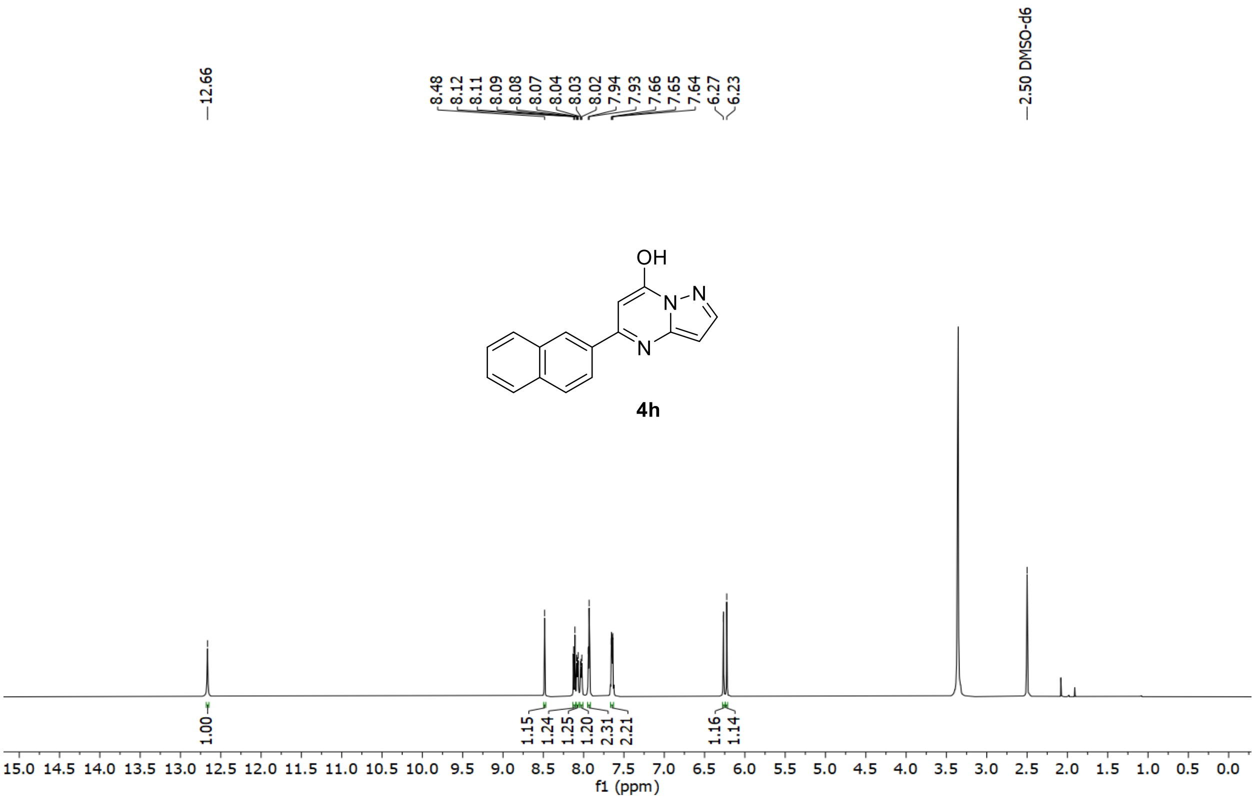

^{13}C NMR (126 MHz, DMSO-d_6)

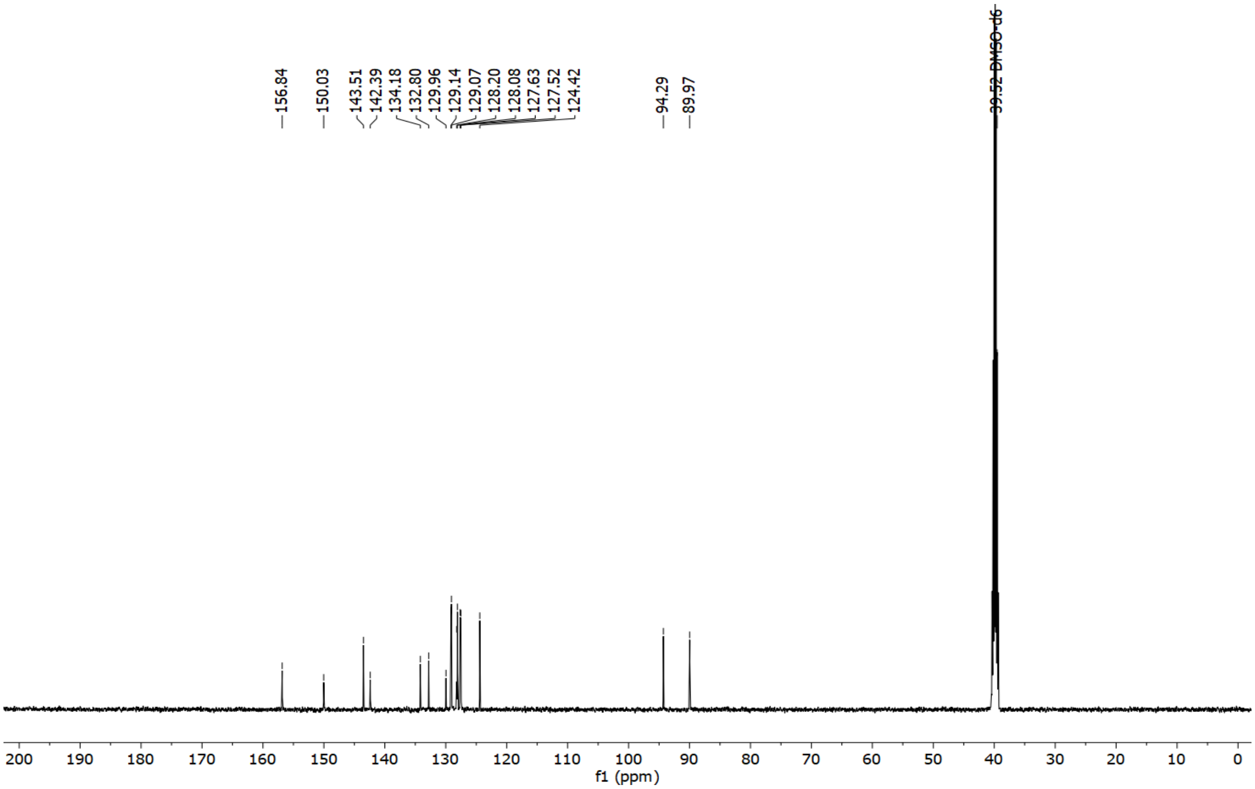

^{1}H-NMR (500 MHz, DMSO-d_6)

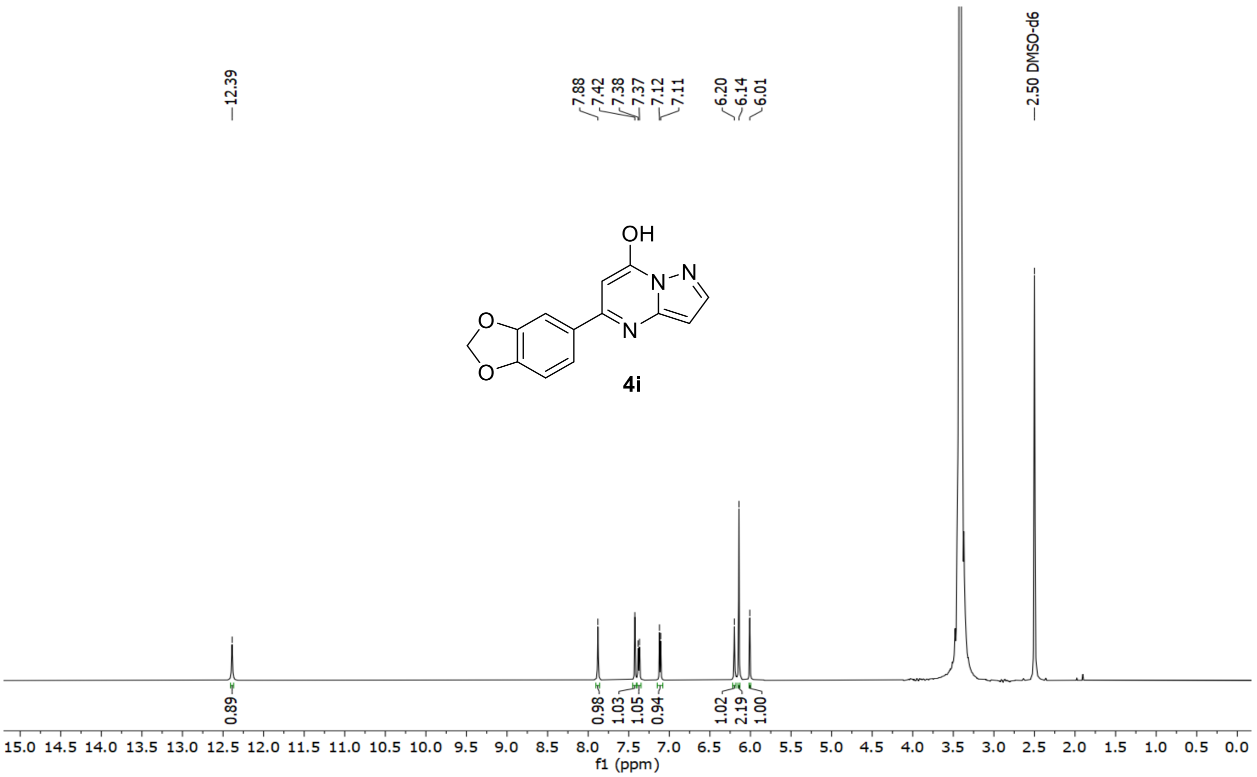

^{13}C NMR (126 MHz, DMSO-d_6)

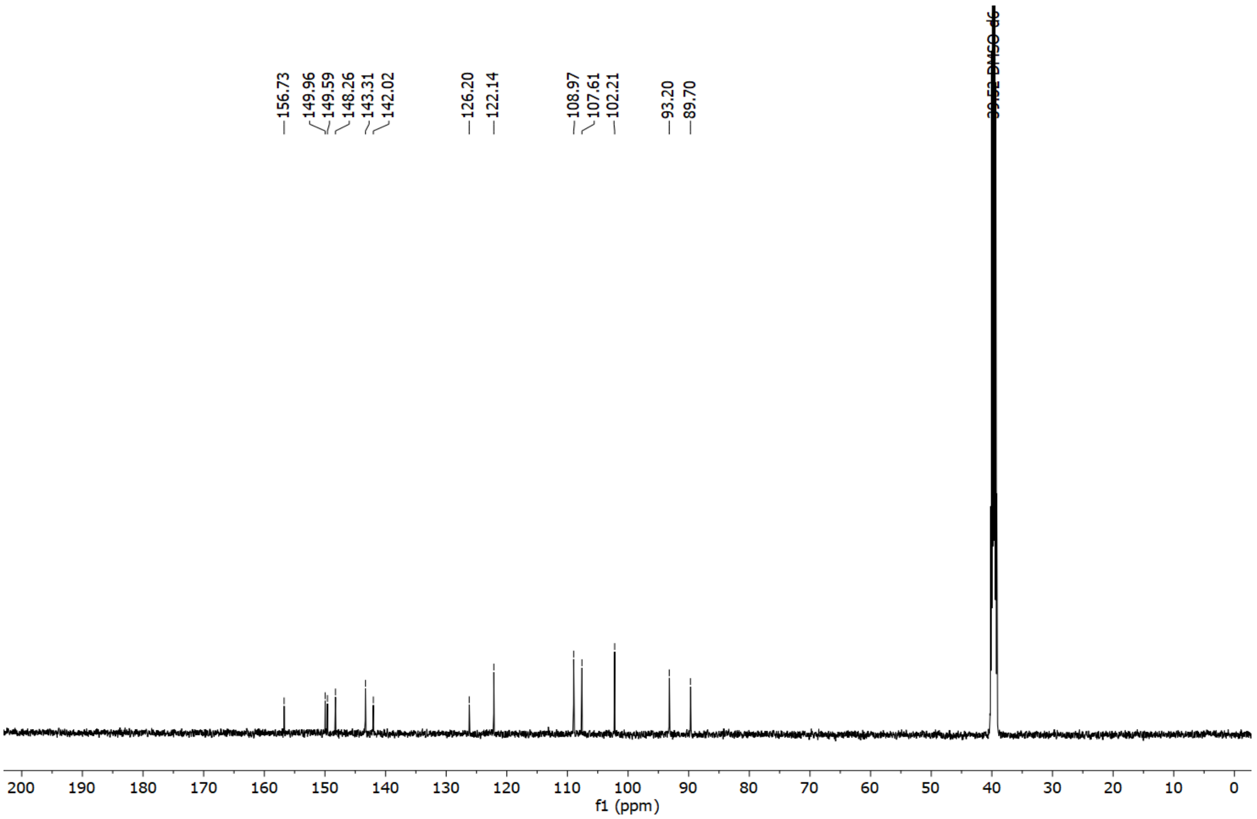

Copies of ^{1}H NMR and ^{13}C NMR of propargylated pyrazolo[1,5-*a*]pyrimidine-7-ol 6a-6i: ^{1}H-NMR (500 MHz, $CDCl_3$)

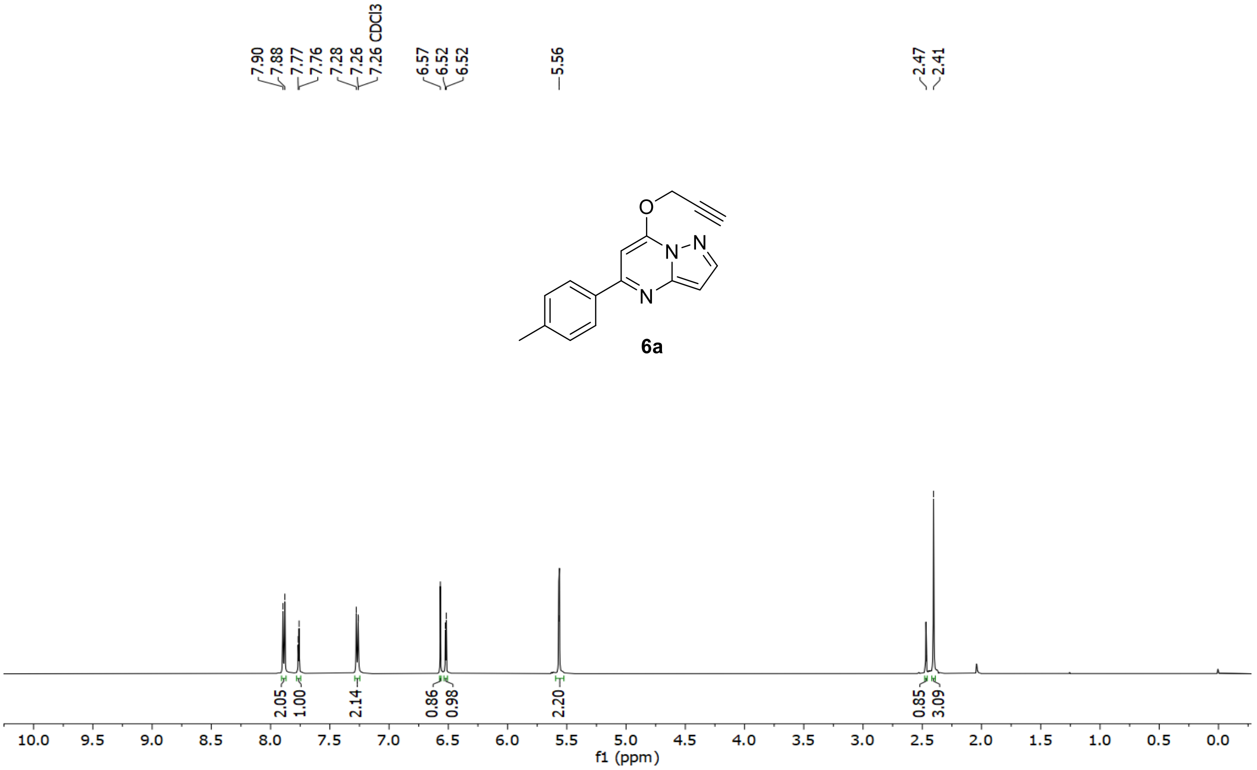

^{13}C NMR (126 MHz, $CDCl_3$)

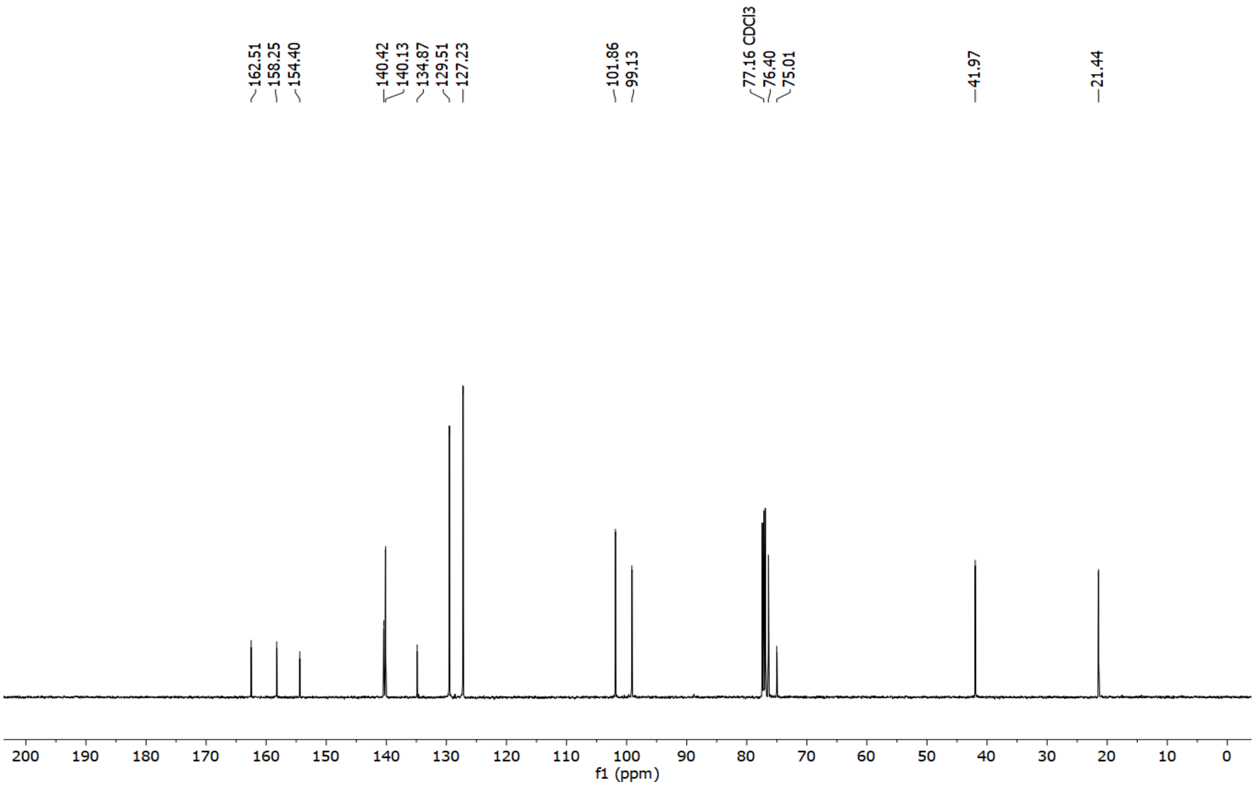

^{1}H-NMR (500 MHz, $CDCl_3$)

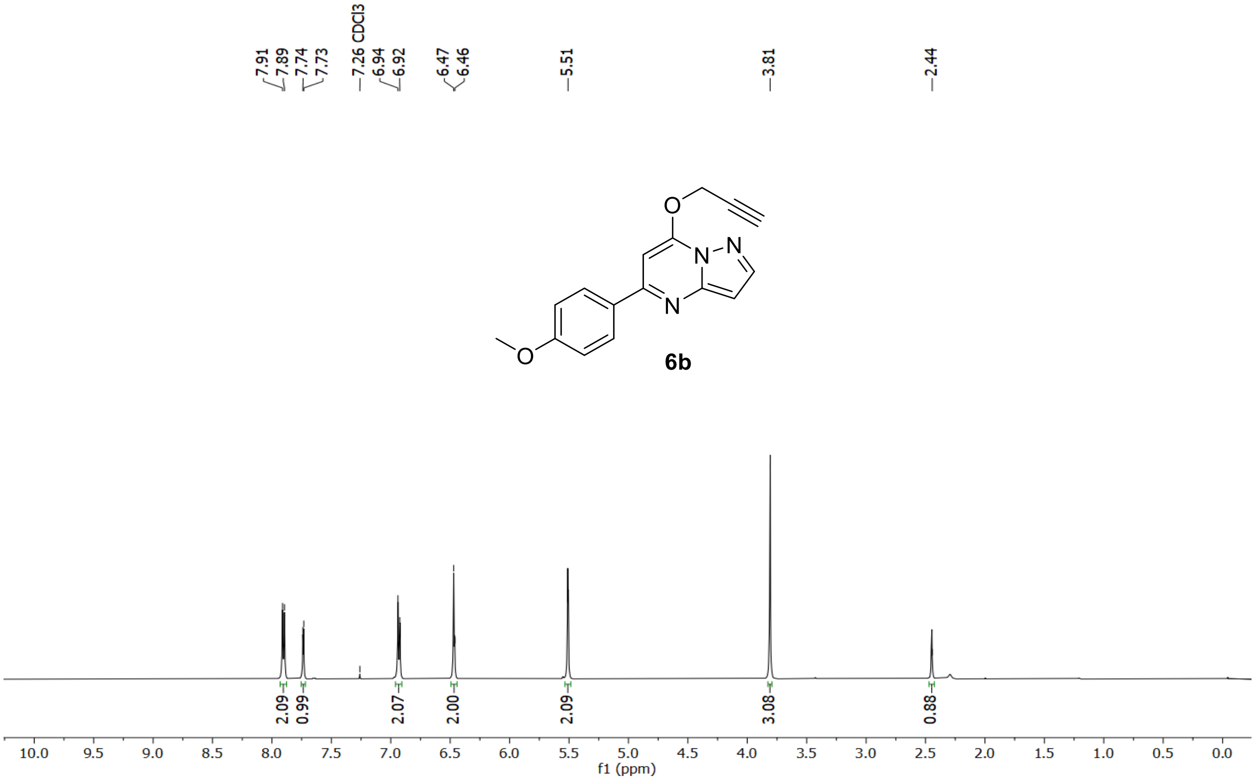

^{13}C NMR (126 MHz, $CDCl_3$)

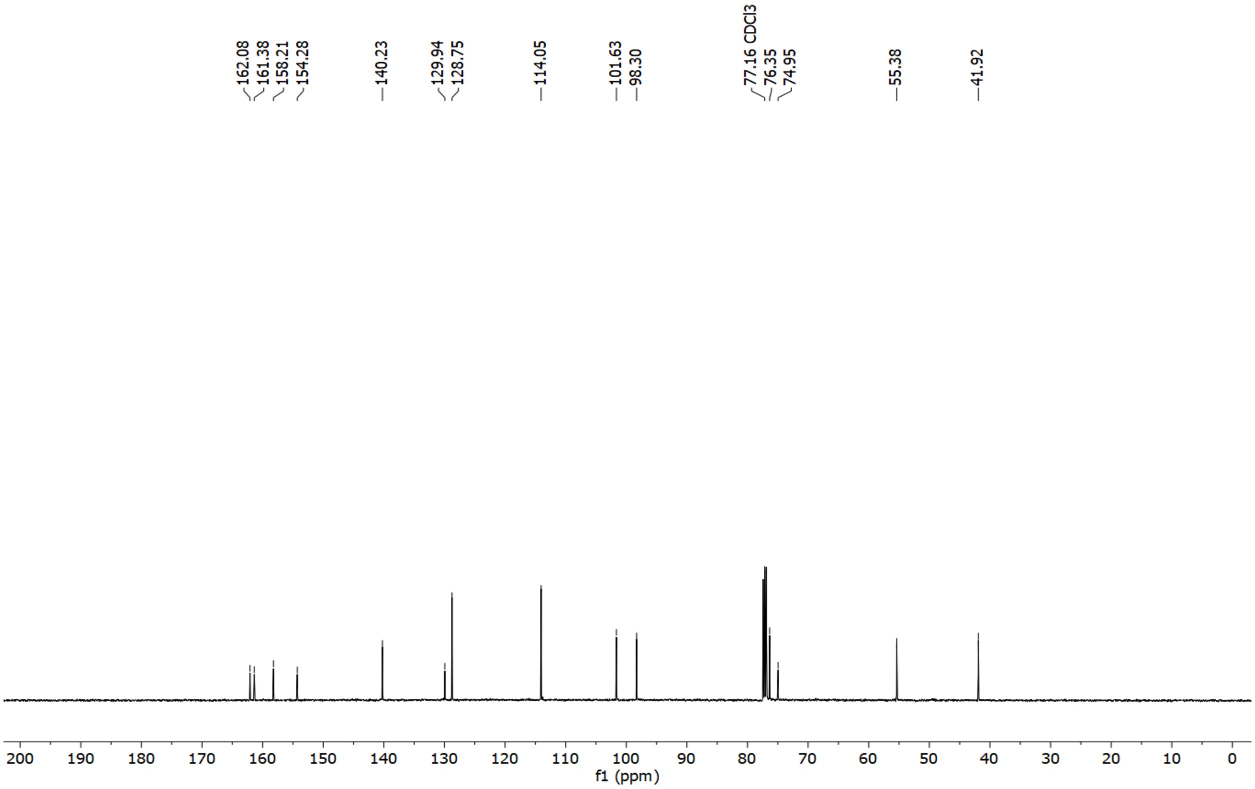

^{1}H-NMR (500 MHz, $CDCl_3$)

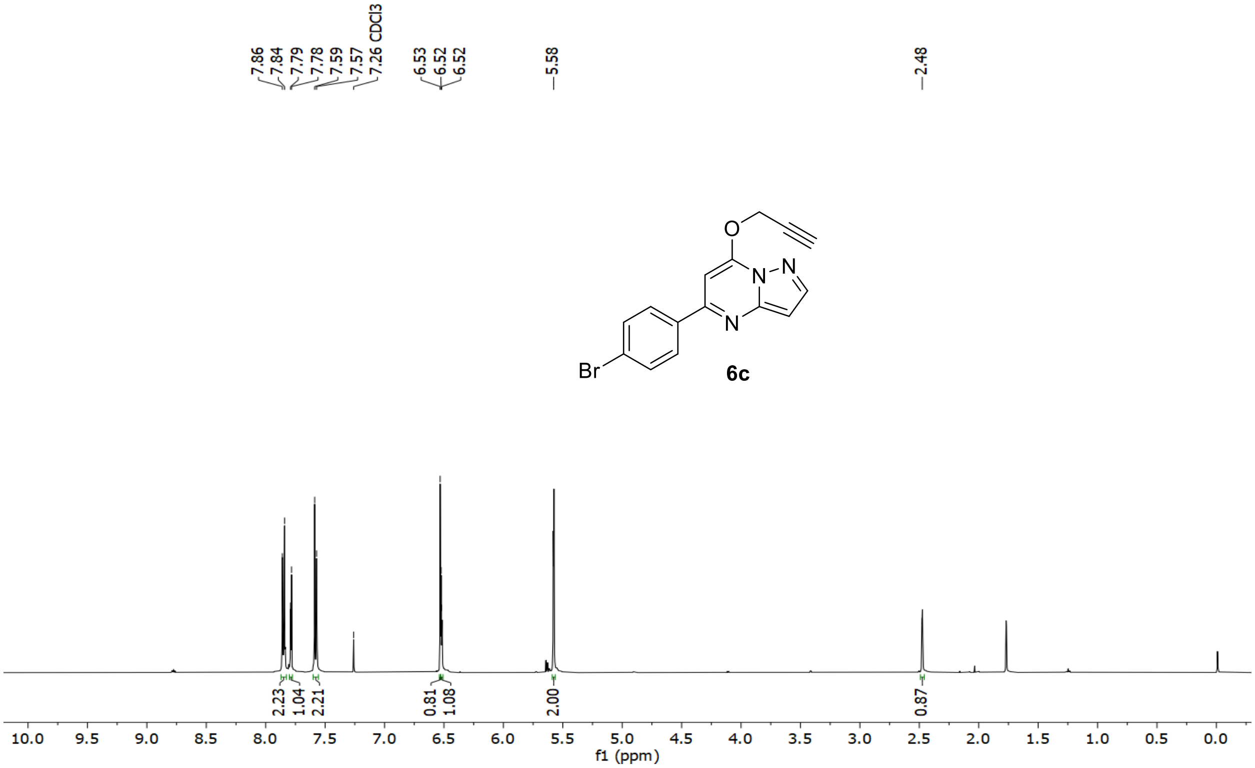

6c

^{13}C NMR (126 MHz, $CDCl_3$)

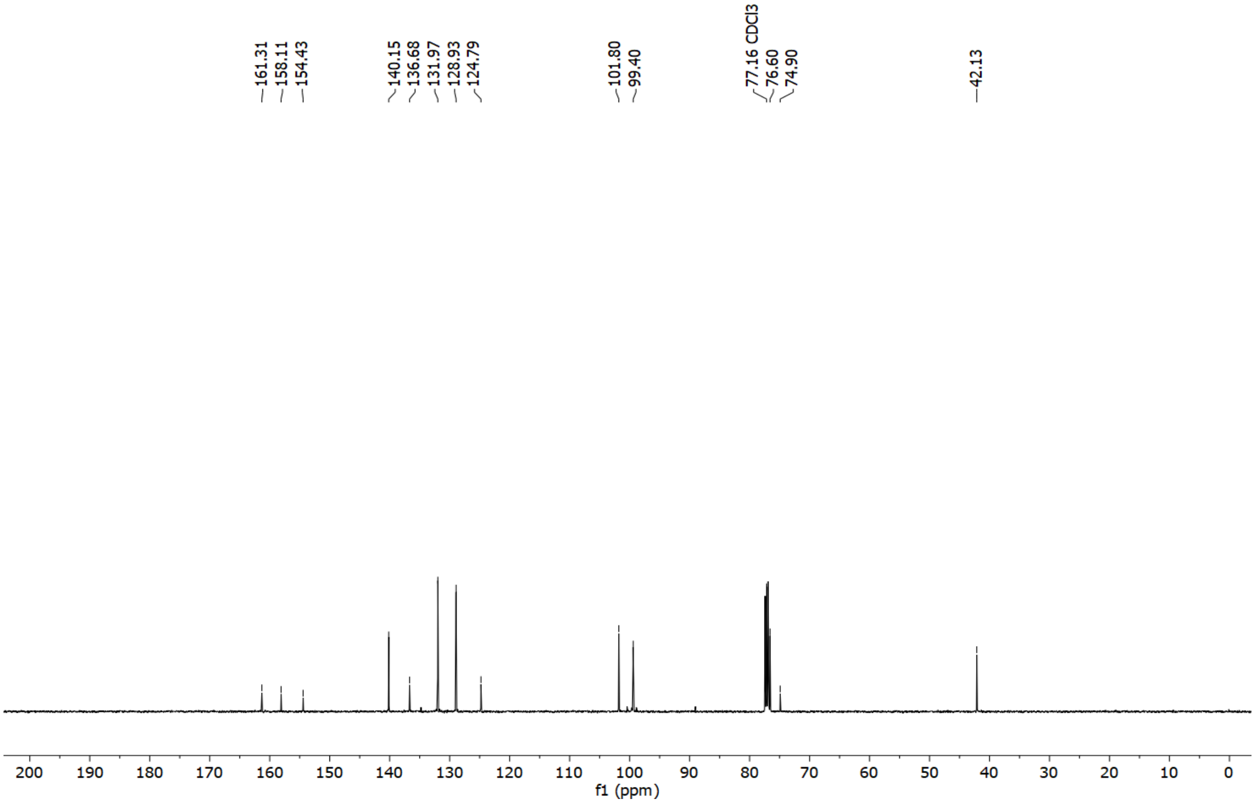

^{1}H-NMR (500 MHz, $CDCl_3$)

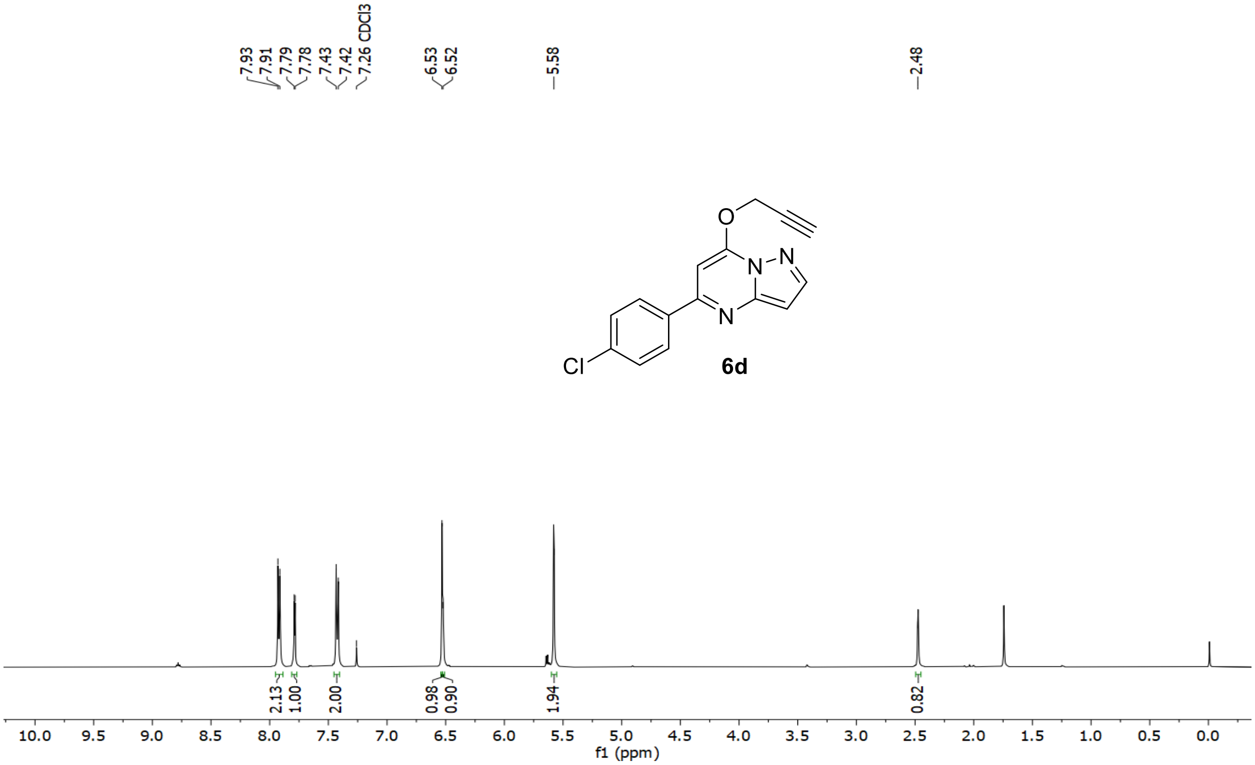

^{13}C NMR (126 MHz, $CDCl_3$)

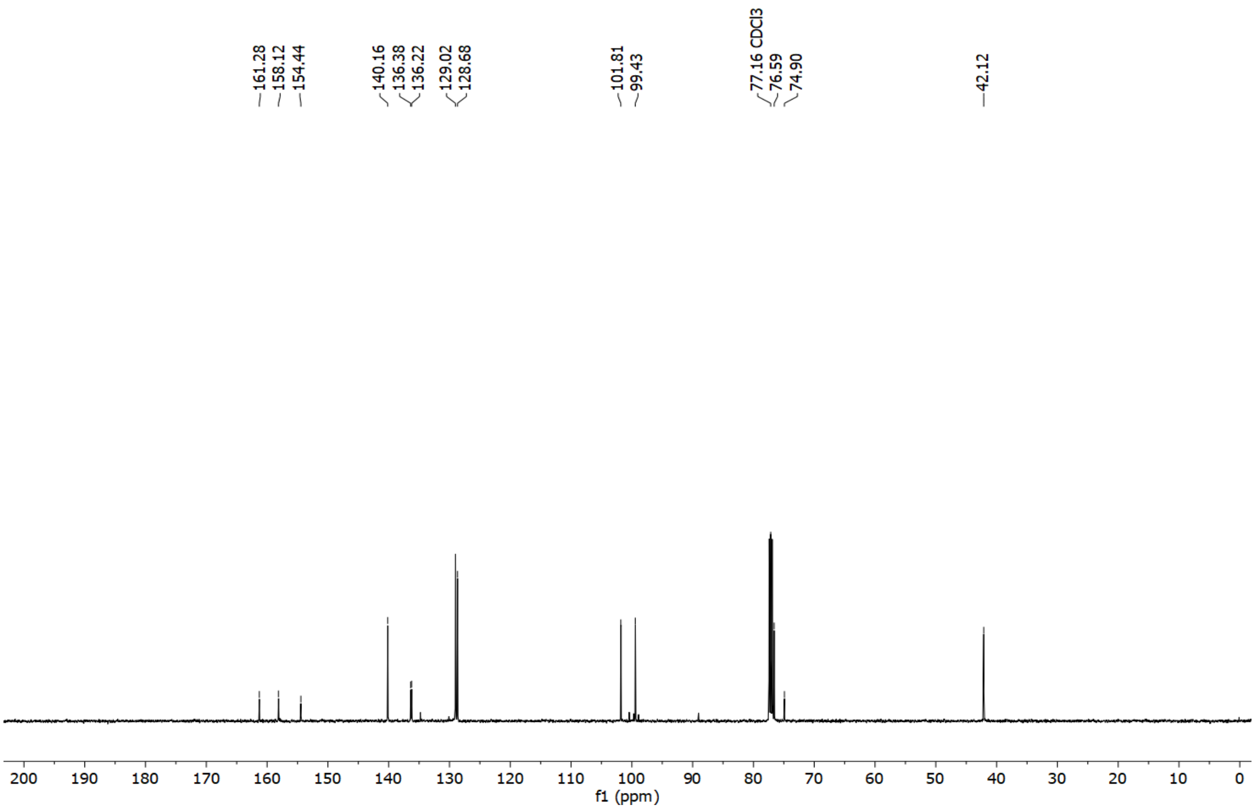

^{1}H-NMR (500 MHz, $CDCl_3$)

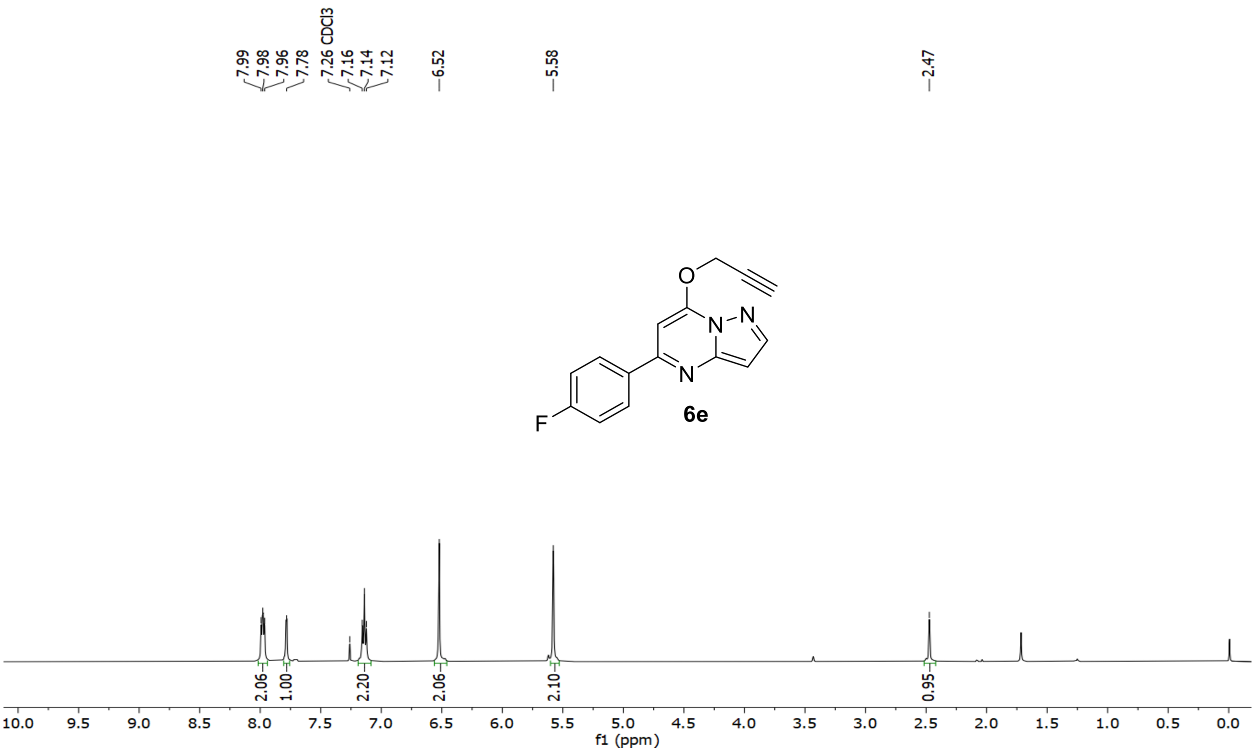

^{13}C NMR (126 MHz, $CDCl_3$)

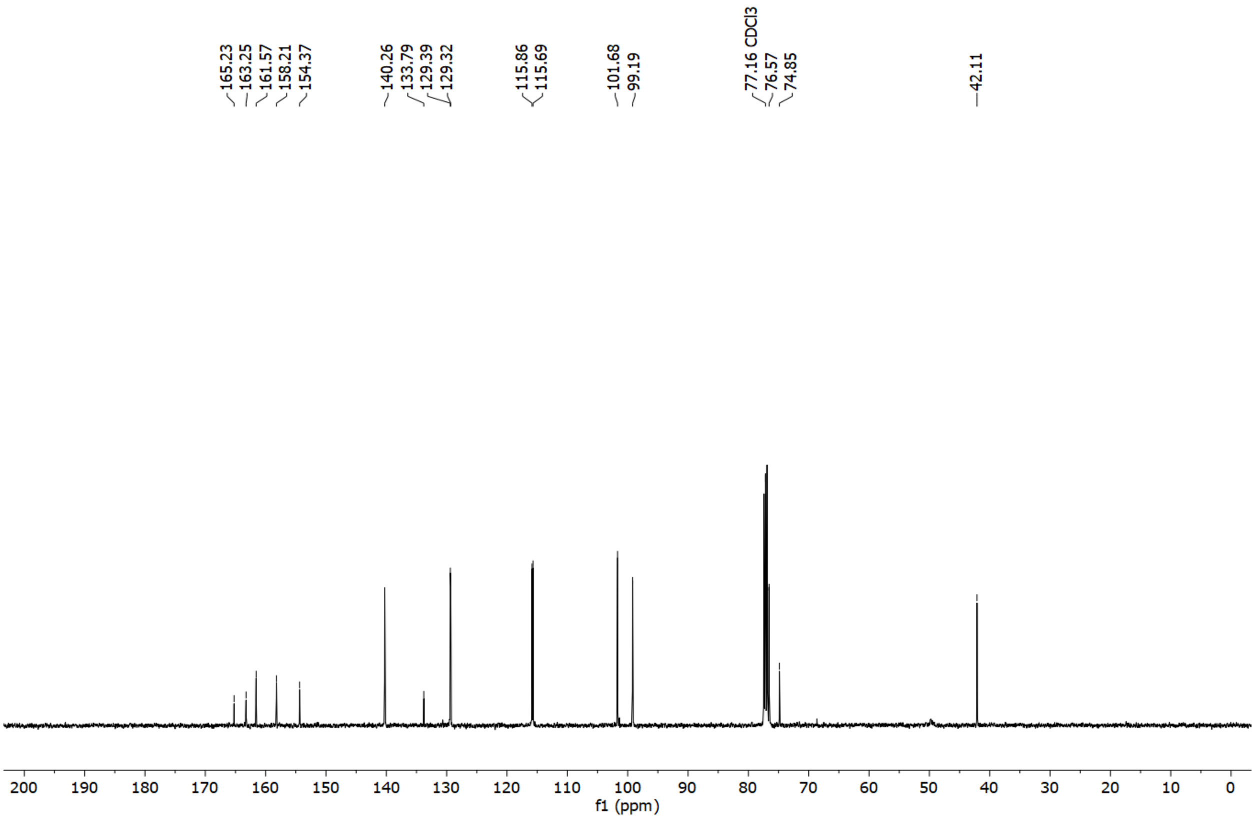

^{1}H-NMR (500 MHz, $CDCl_3$)

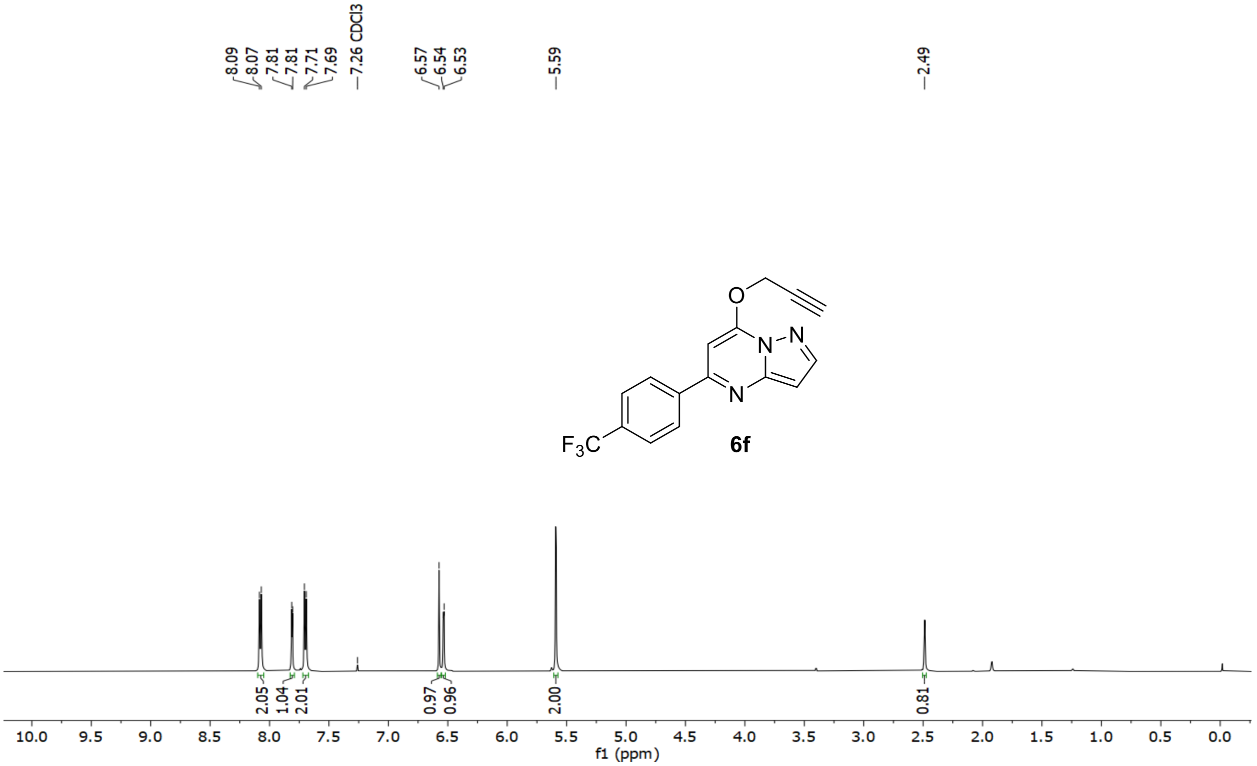

^{13}C NMR (126 MHz, $CDCl_3$)

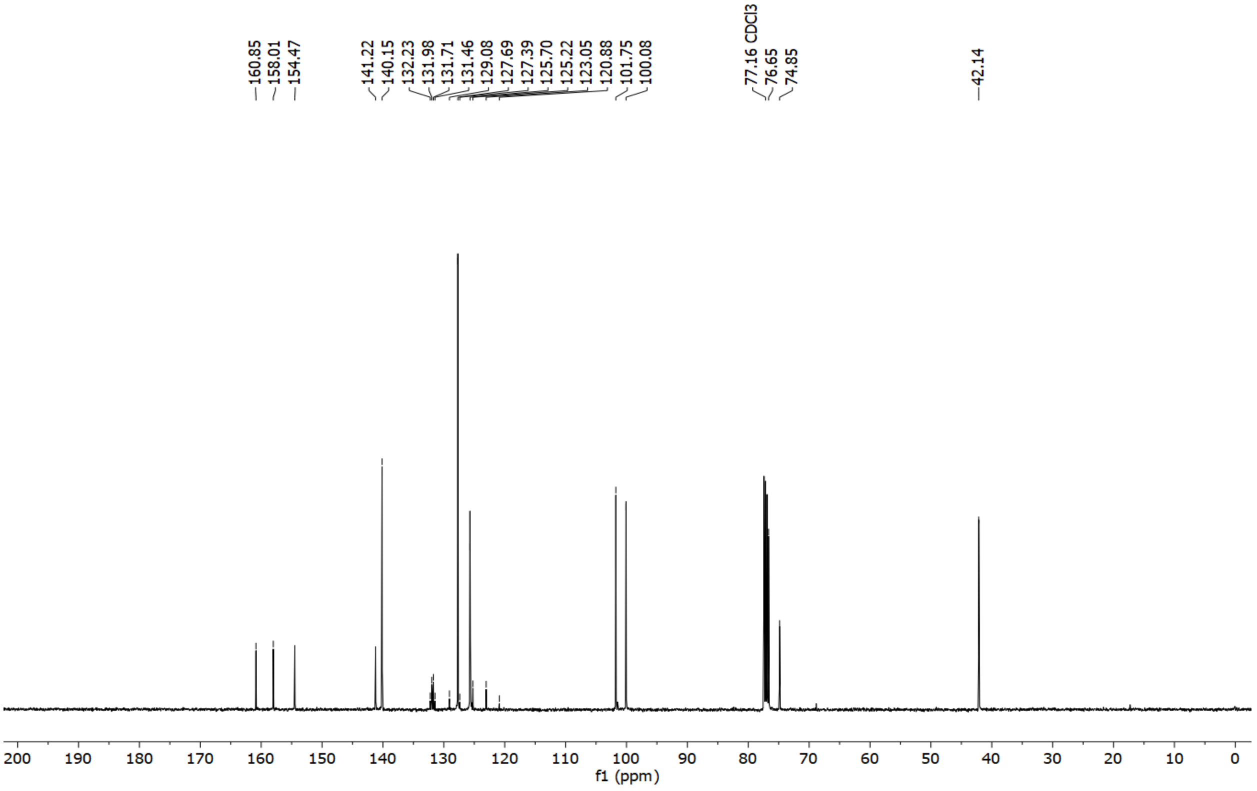

^{1}H-NMR (500 MHz, CDCl$_3$)

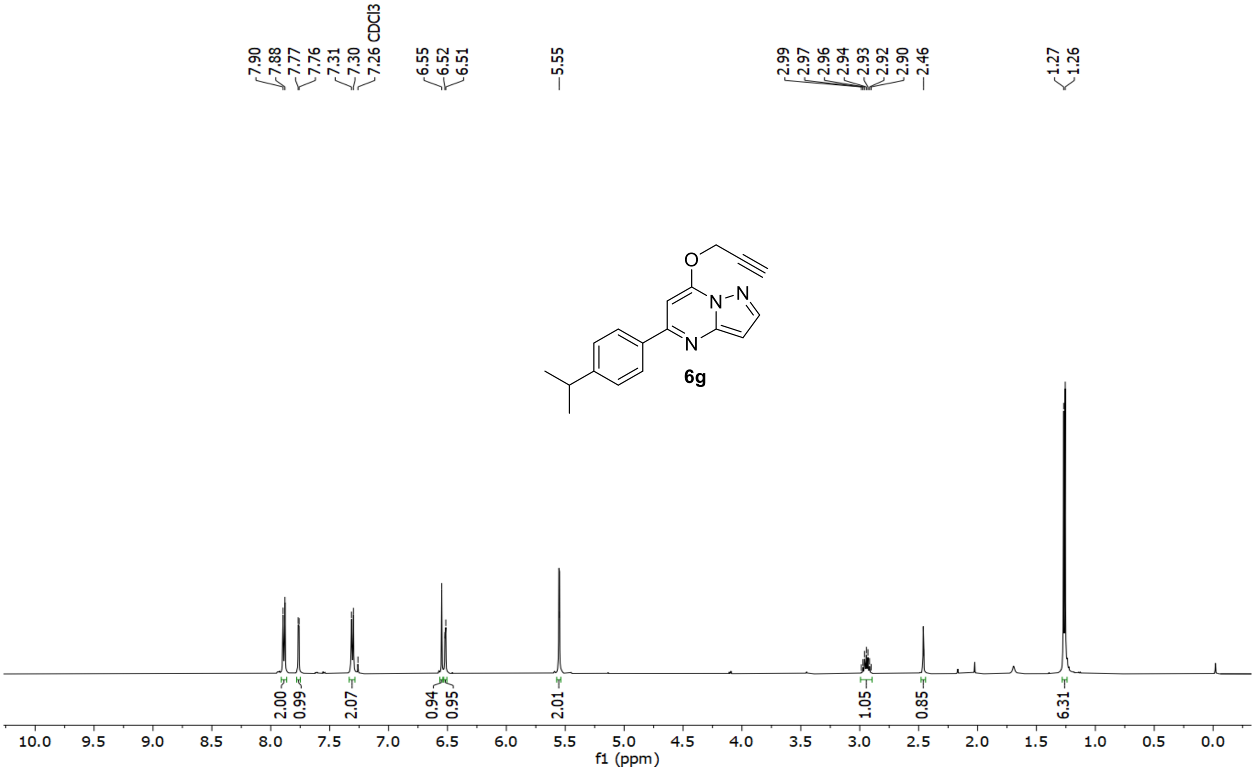

^{13}C NMR (126 MHz, CDCl$_3$)

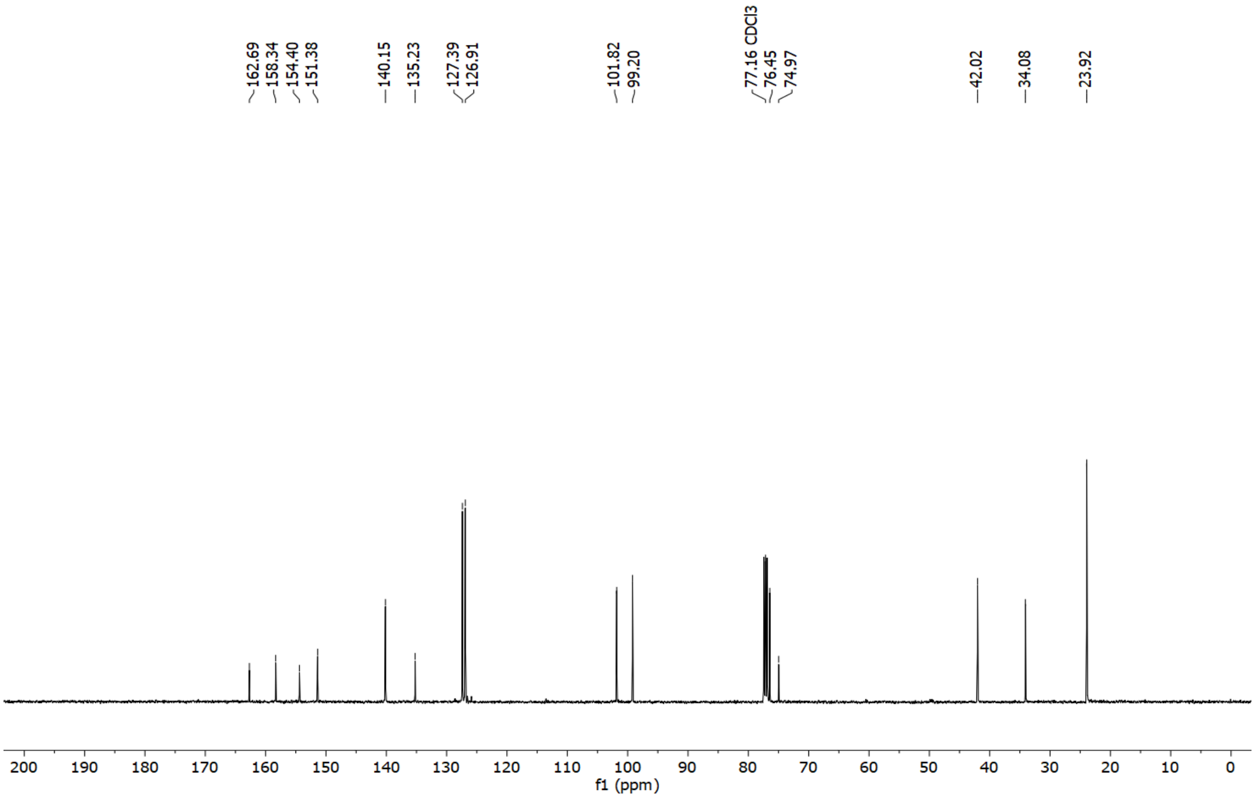

^{1}H-NMR (500 MHz, DMSO-*d*6)

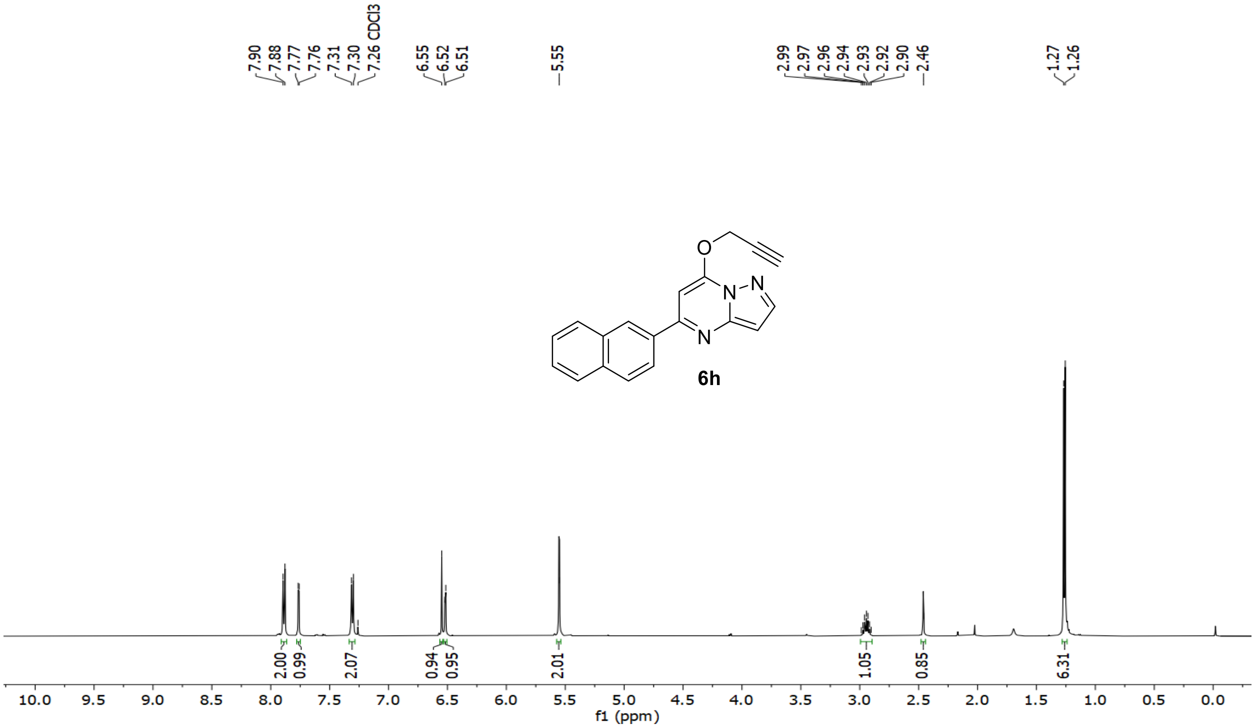

^{13}C NMR (126 MHz, DMSO-*d*6)

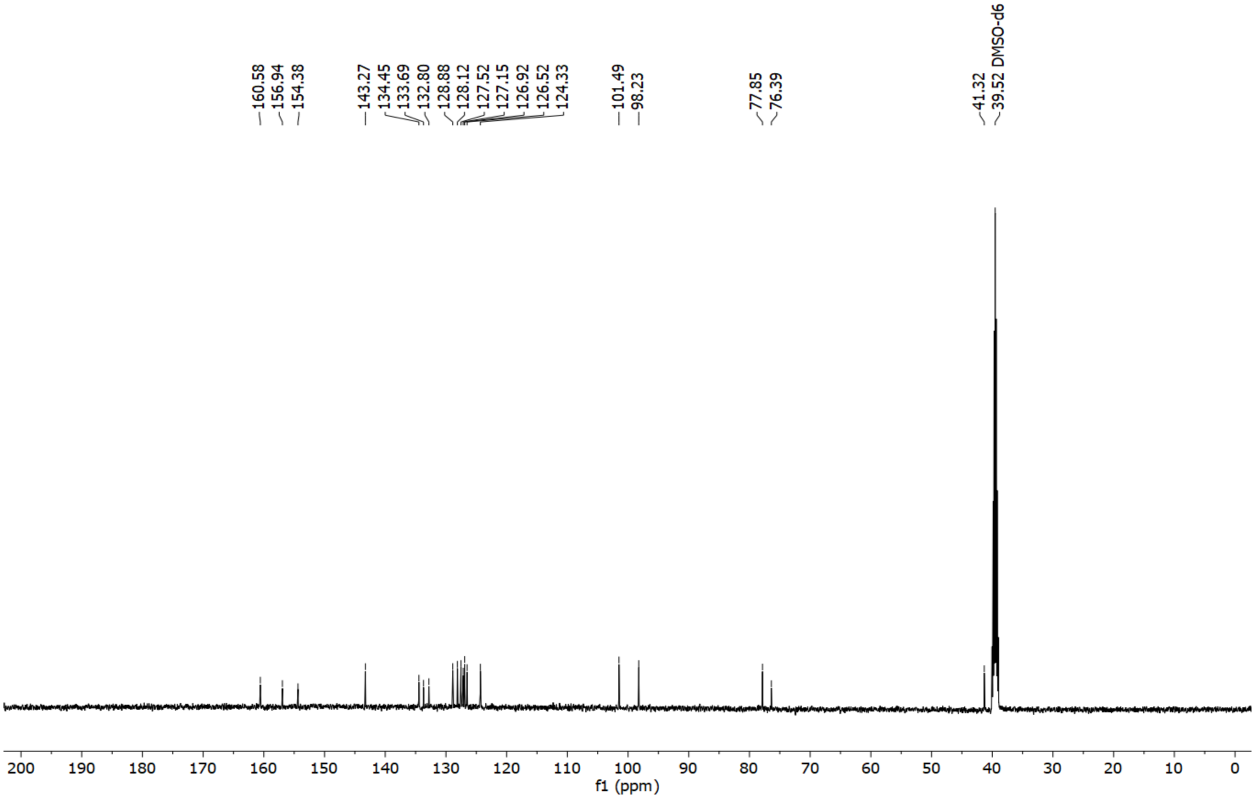

^{1}H-NMR (500 MHz, $CDCl_3$)

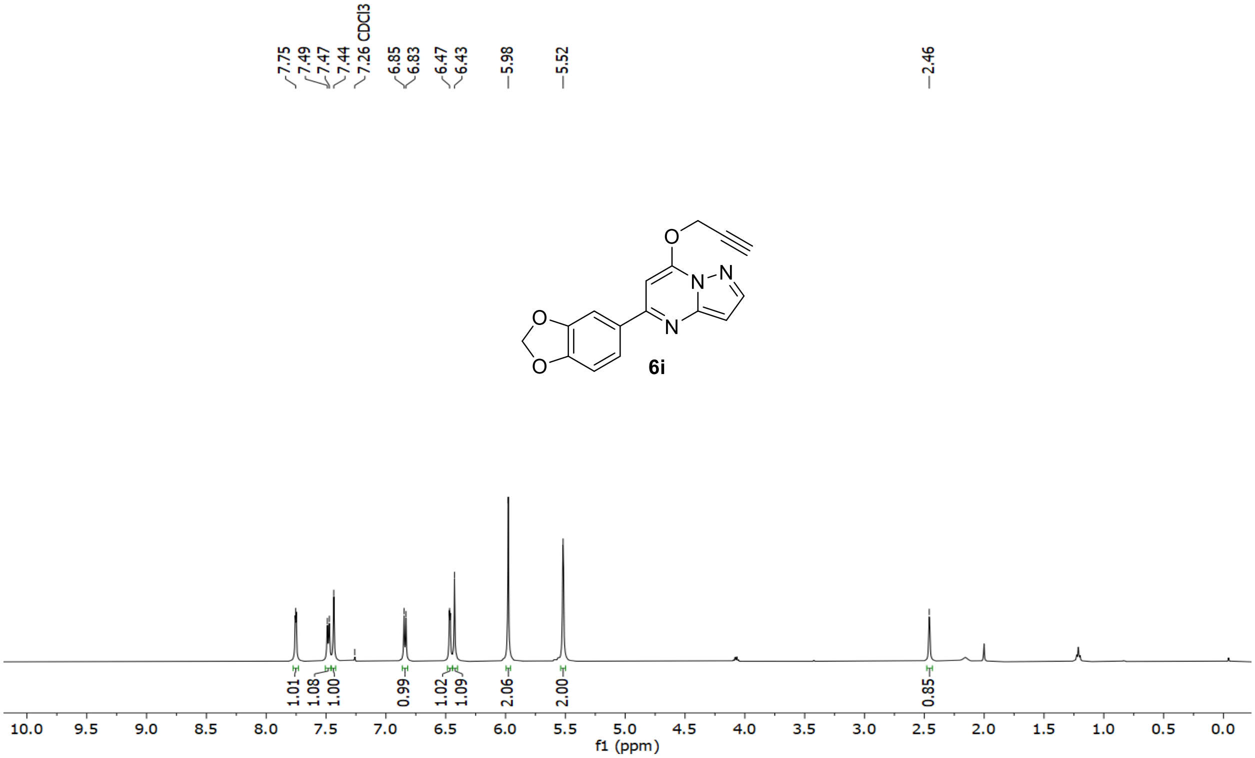

^{13}C NMR (126 MHz, $CDCl_3$)

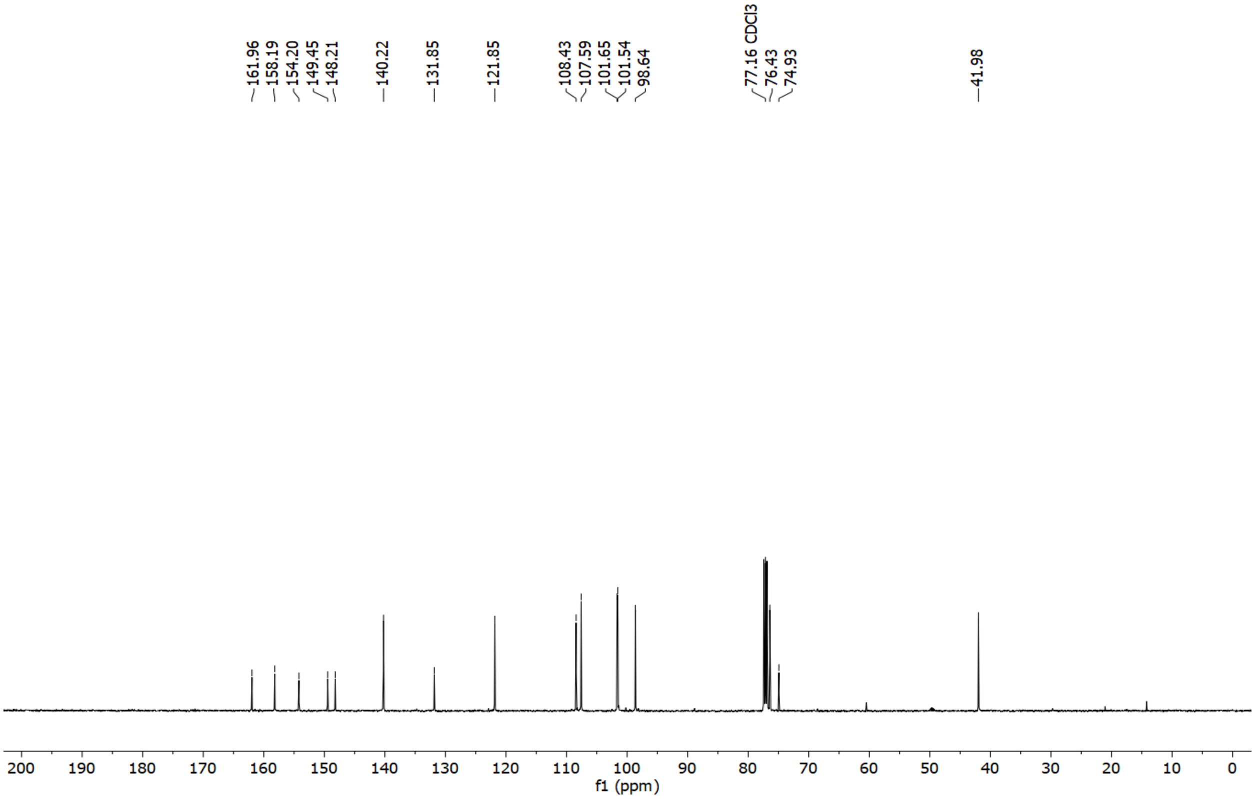

Copies of ^{1}H NMR and ^{13}C NMR of azido glycosides 8a-8c:

^{1}H-NMR (500 MHz, CDCl$_3$)

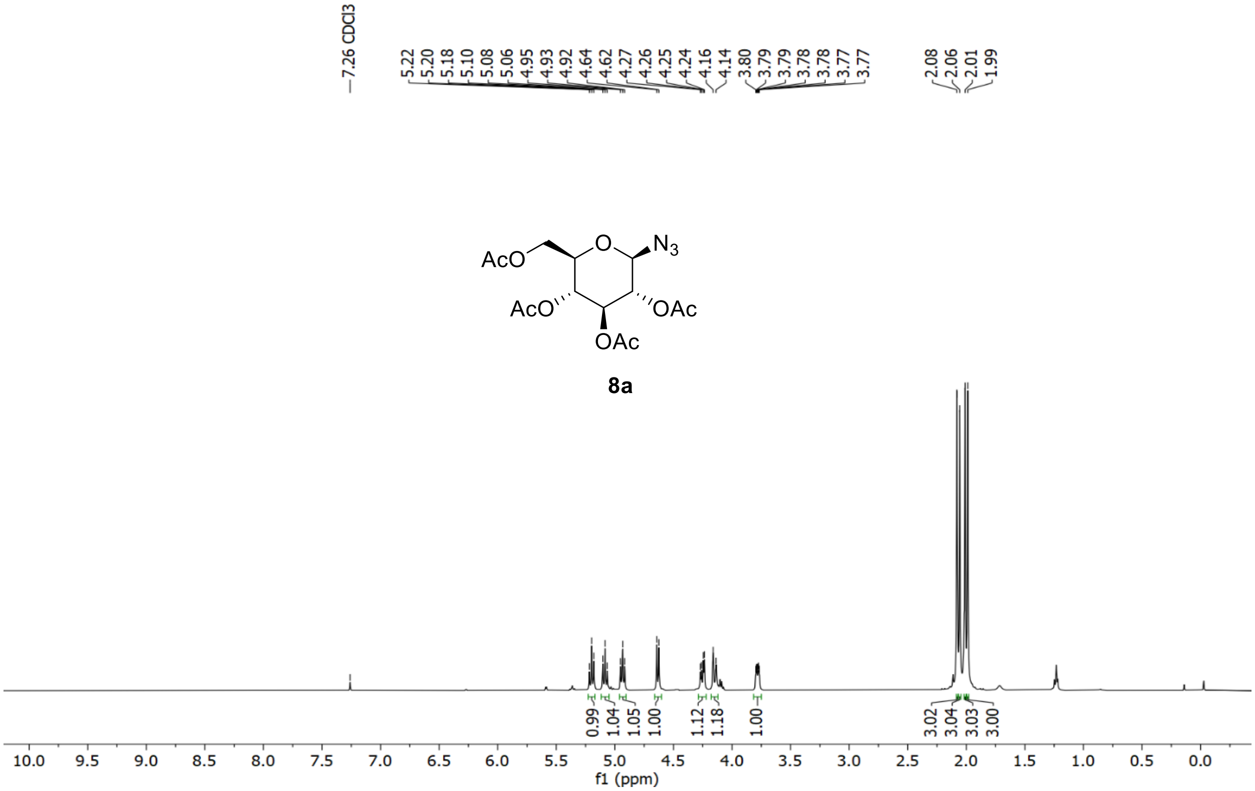

8a

^{13}C NMR (126 MHz, CDCl$_3$)

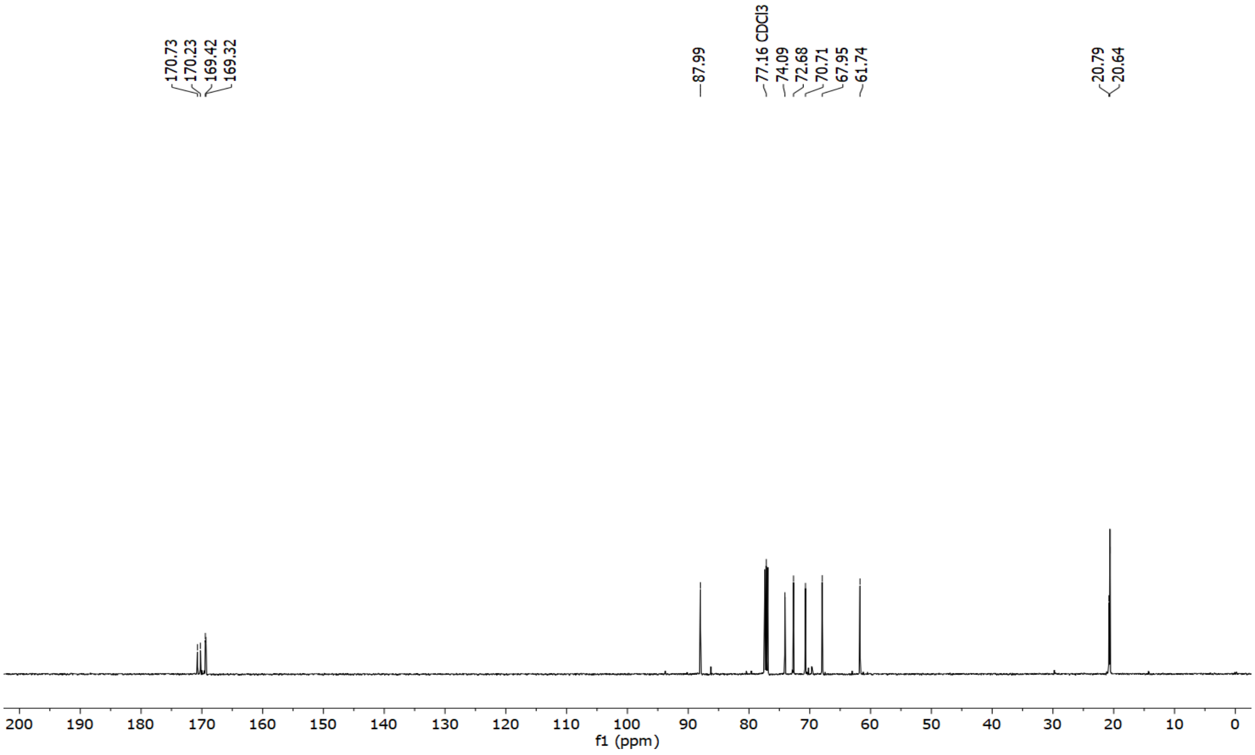

^{1}H-NMR (500 MHz, $CDCl_3$)

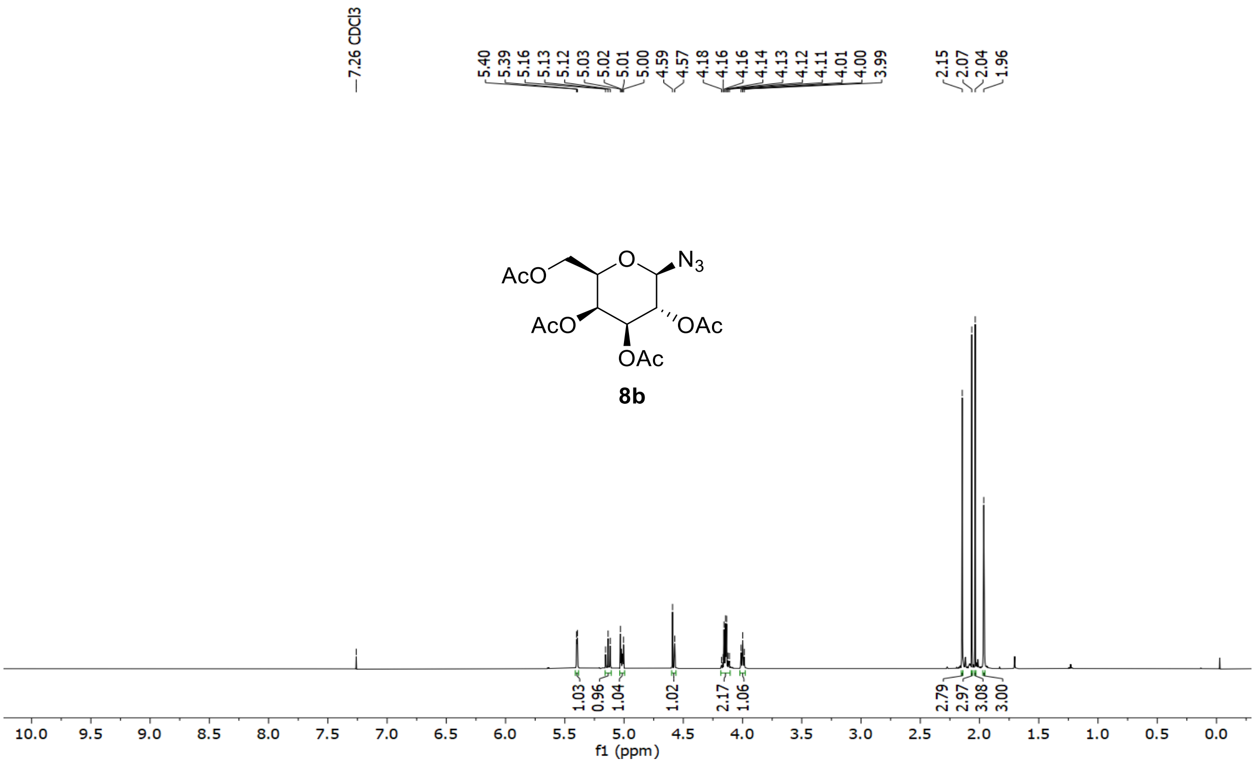

^{13}C NMR (126 MHz, $CDCl_3$)

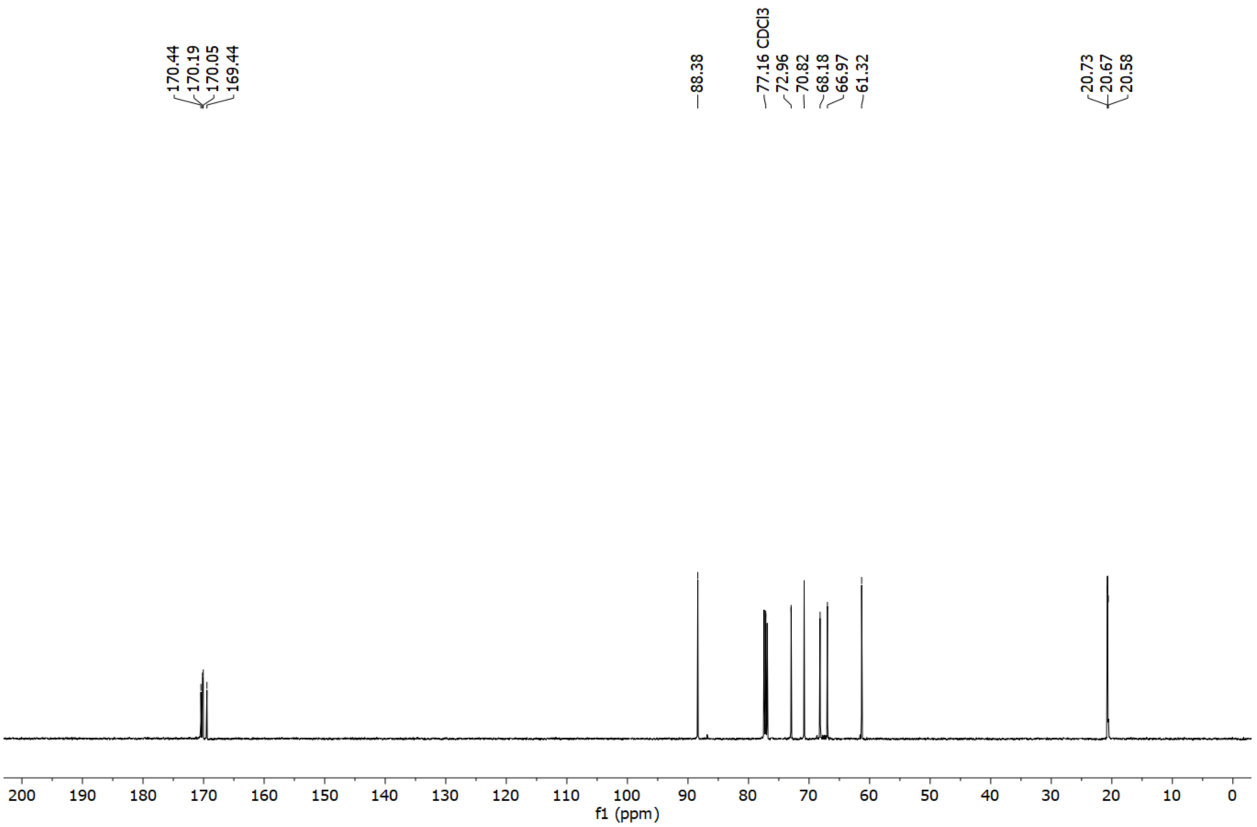

^{1}H-NMR (500 MHz, $CDCl_3$)

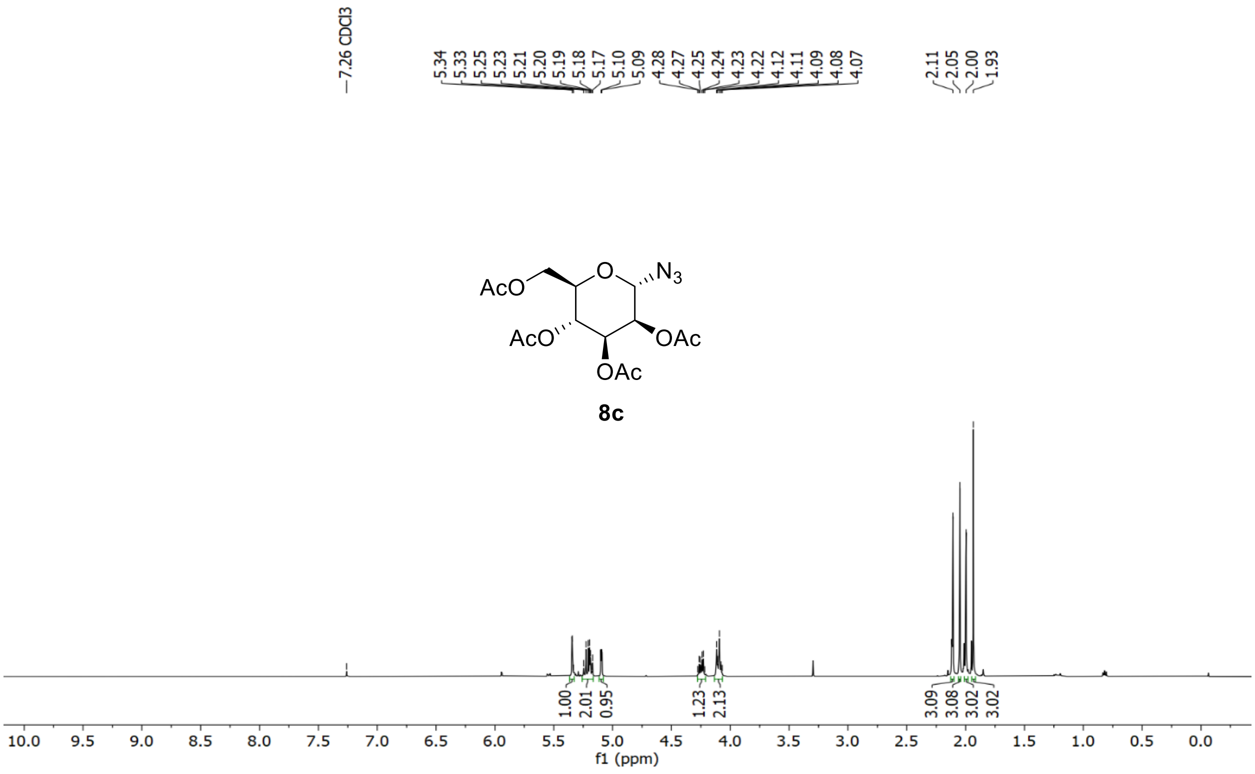

8c

^{13}C NMR (126 MHz, $CDCl_3$)

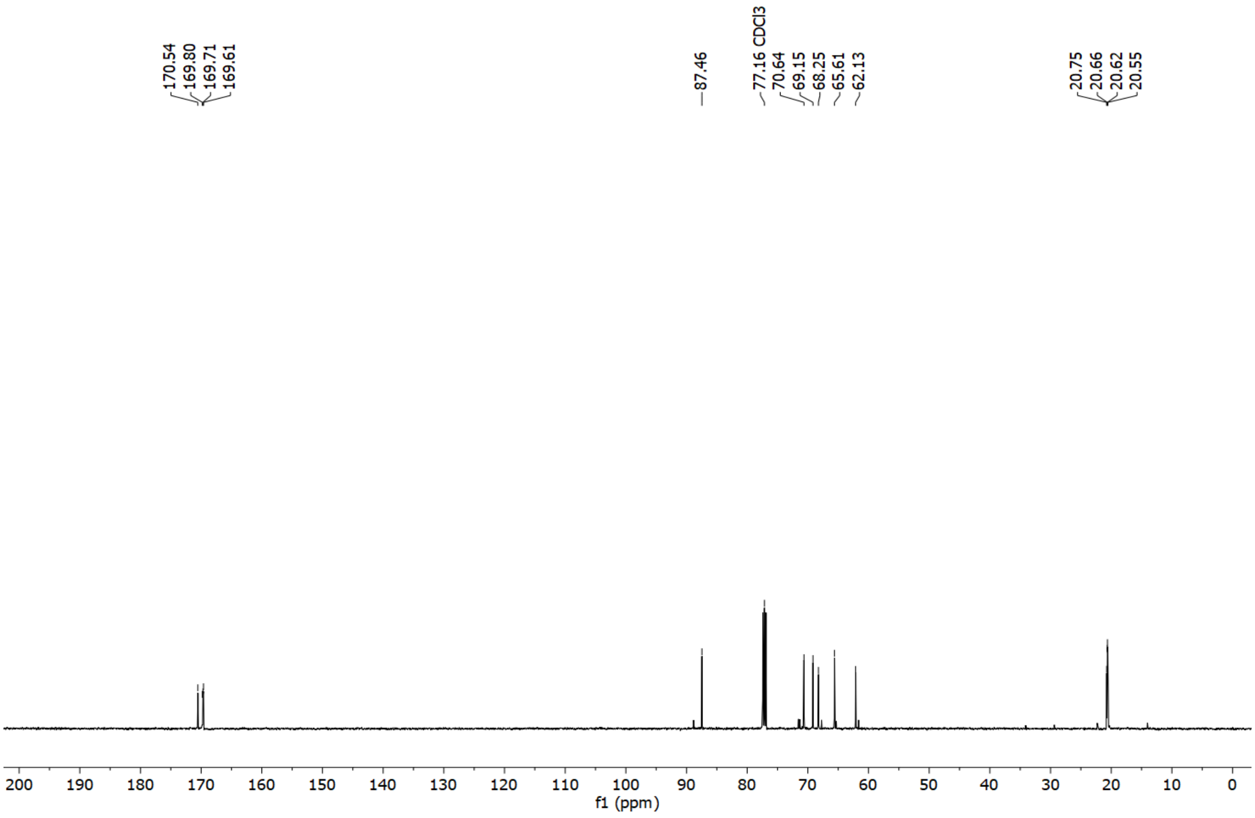

Copies of ^{1}H NMR and ^{13}C NMR of pyrazolo[1,5-*a*]pyrimidine glycohycohybrids 9a-9i, 10a-10i, 11a-11i: ^{1}H-NMR (500 MHz, $CDCl_3$)

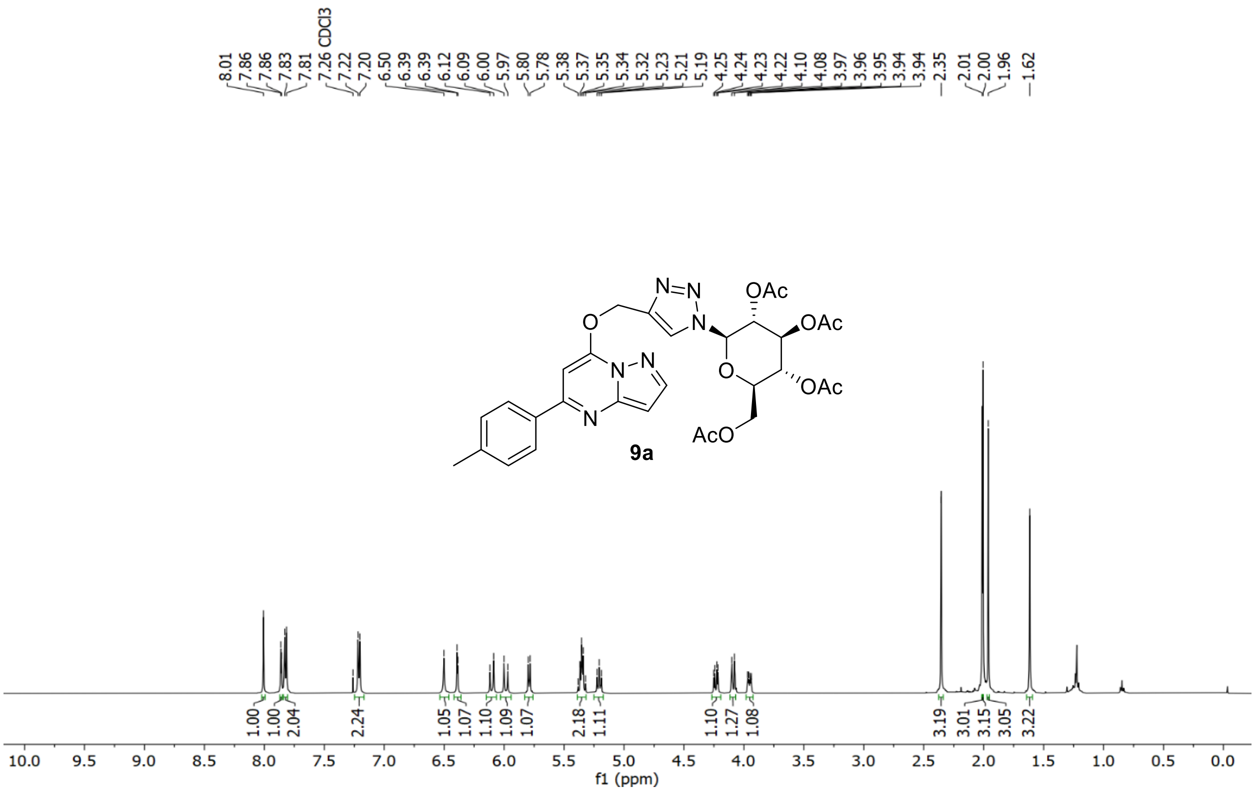

^{13}C NMR (126 MHz, $CDCl_3$)

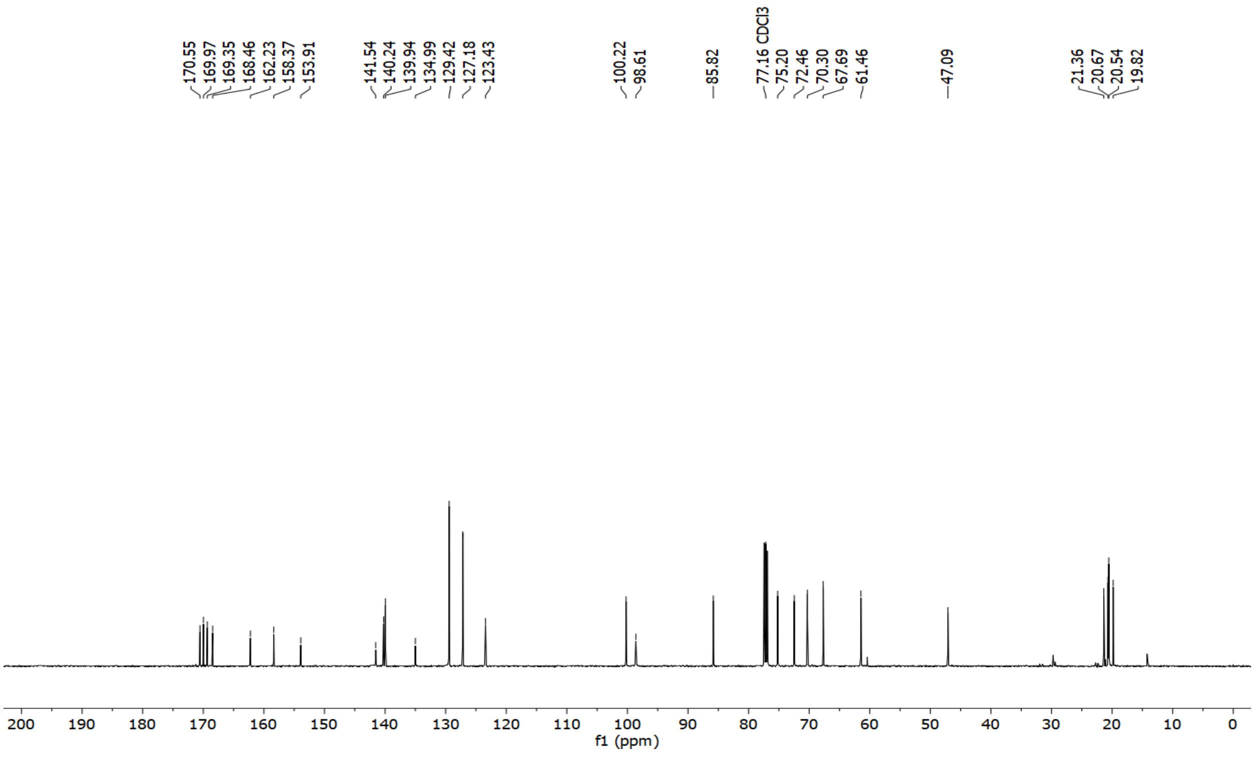

^{1}H-NMR (500 MHz, $CDCl_3$)

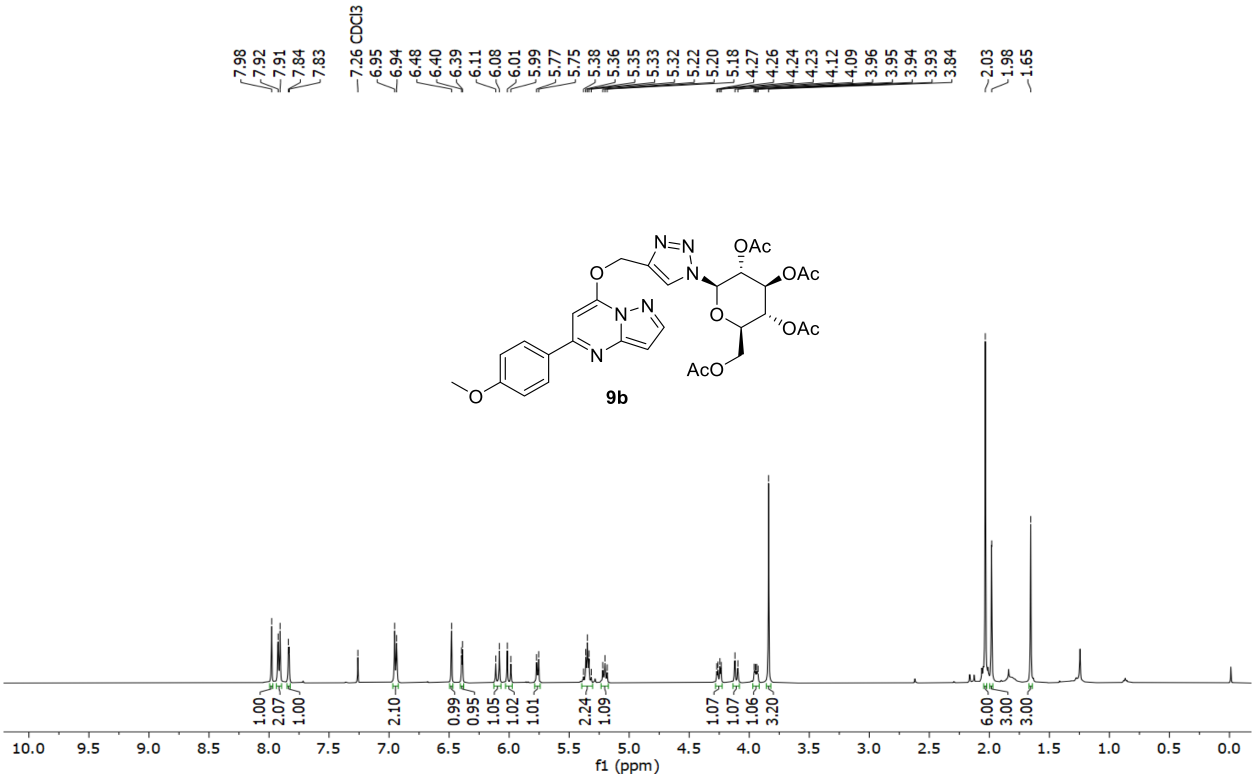

^{13}C NMR (126 MHz, $CDCl_3$)

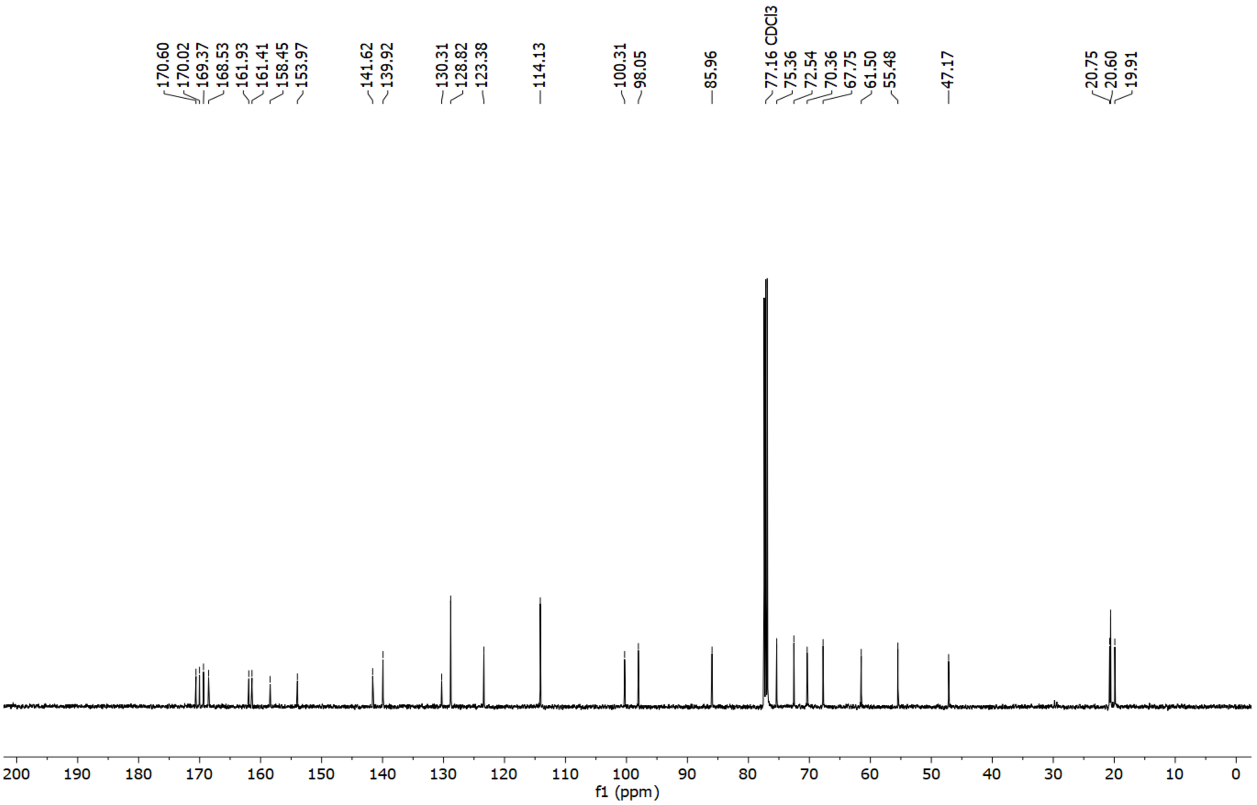

^{1}H-NMR (500 MHz, $CDCl_3$)

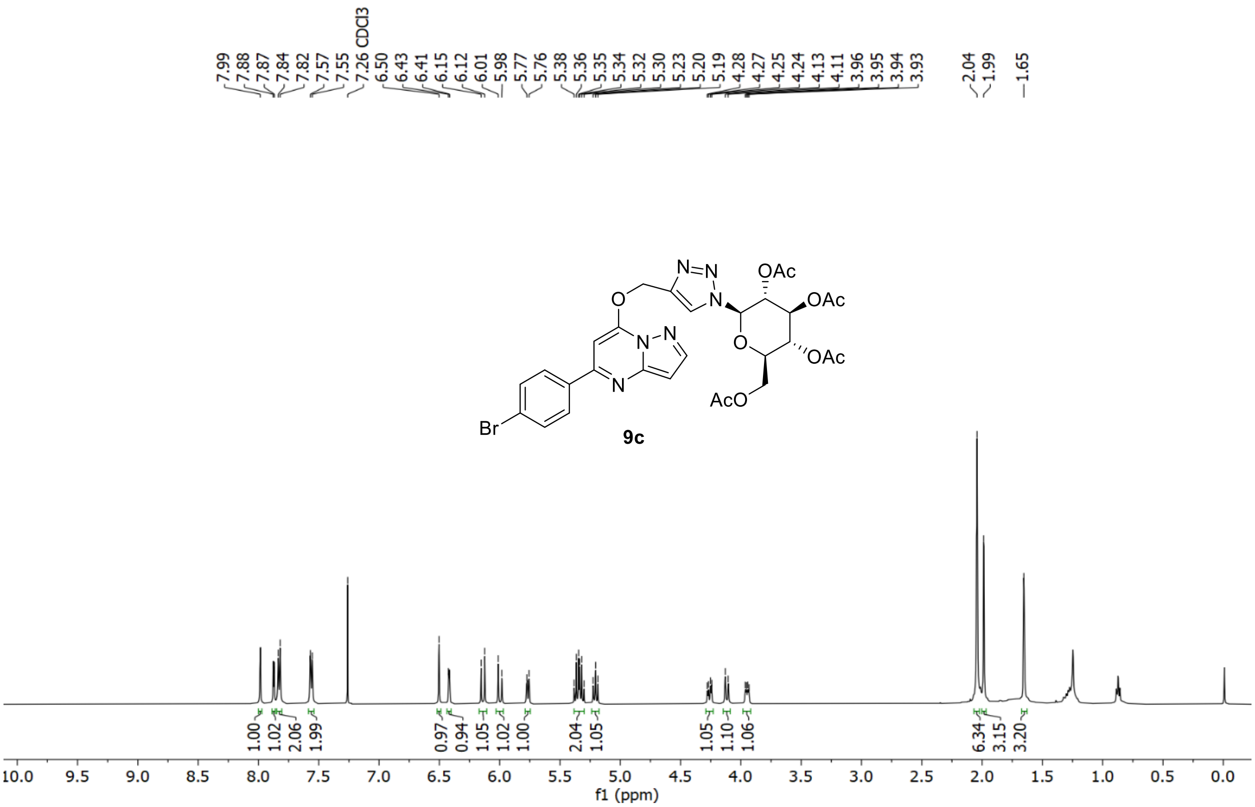

^{13}C NMR (126 MHz, $CDCl_3$)

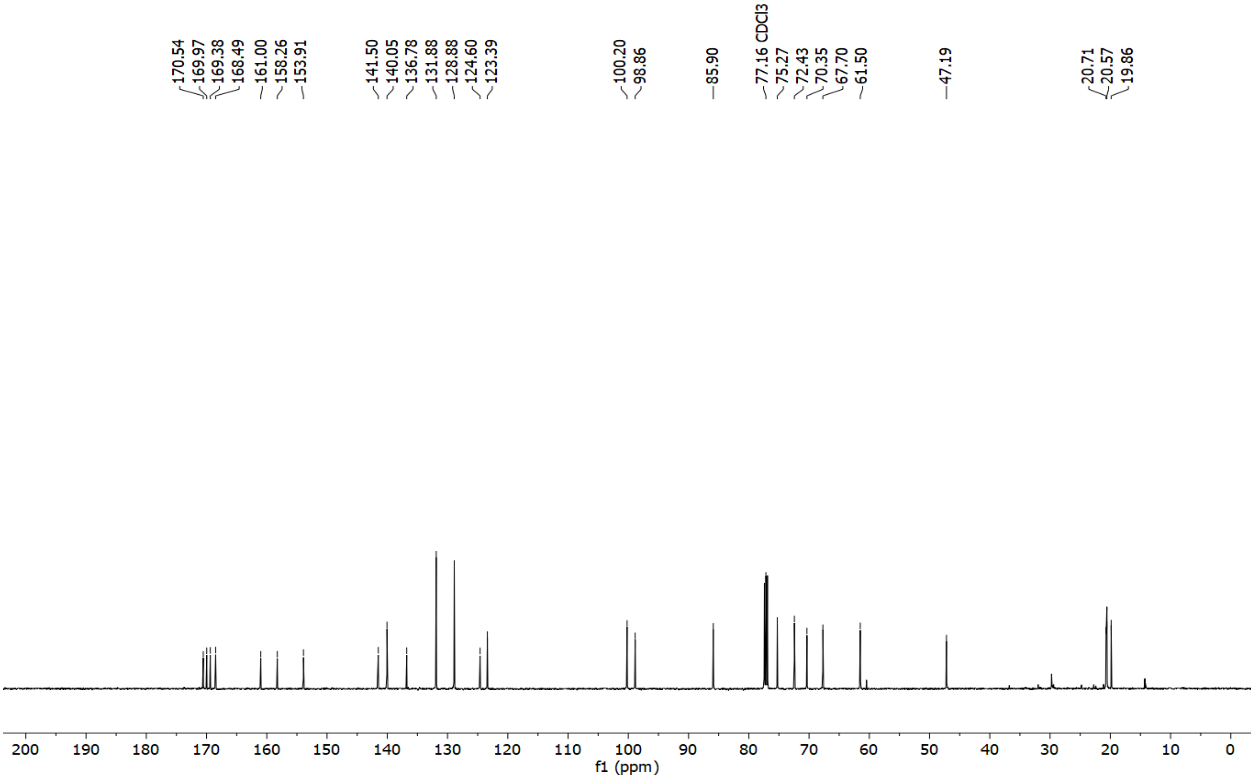

^{1}H-NMR (500 MHz, CDCl₃)

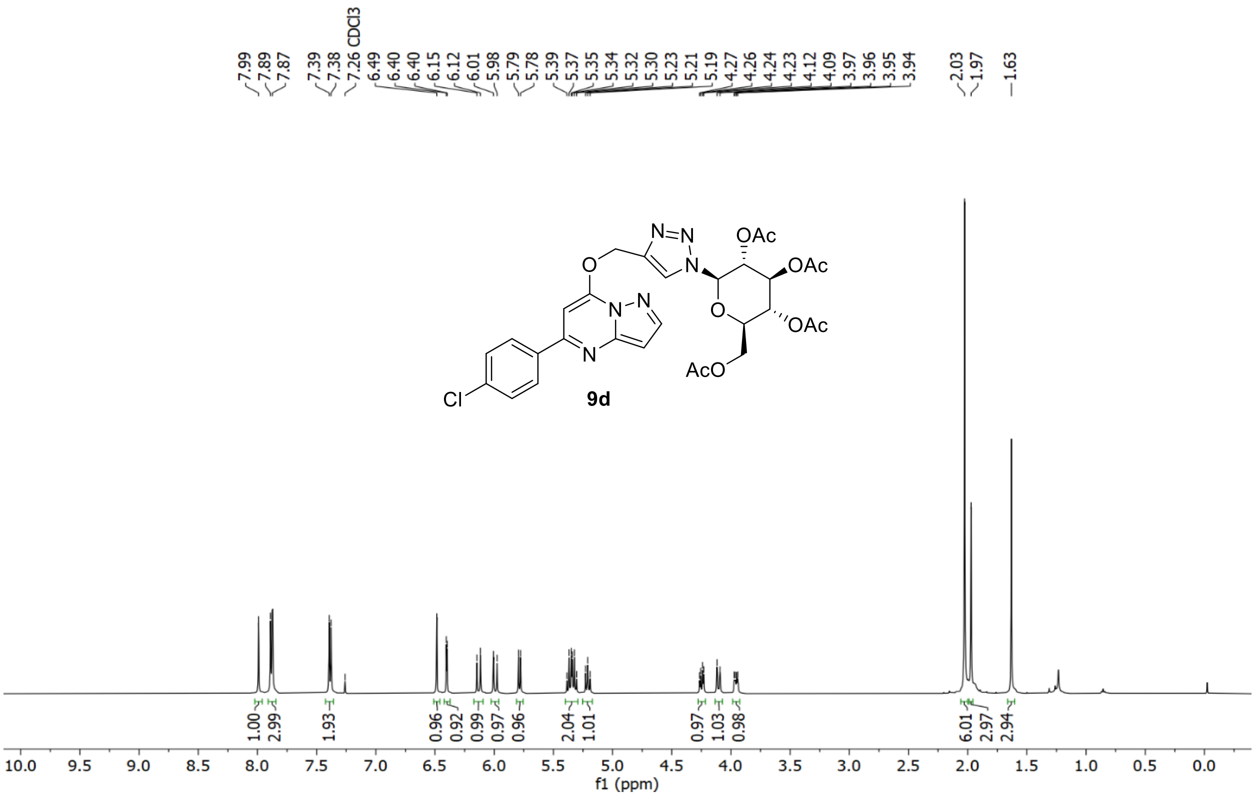

^{13}C NMR (126 MHz, CDCl₃)

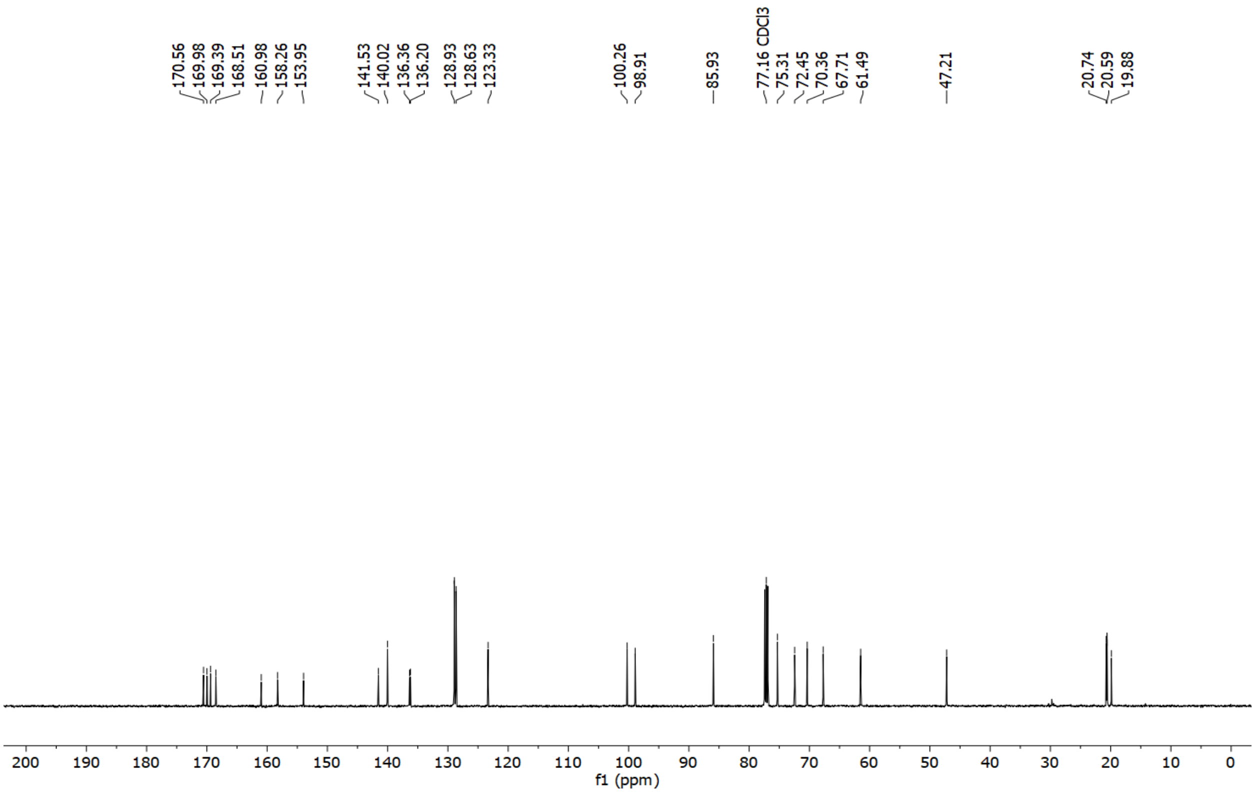

^{1}H-NMR (500 MHz, $CDCl_3$)

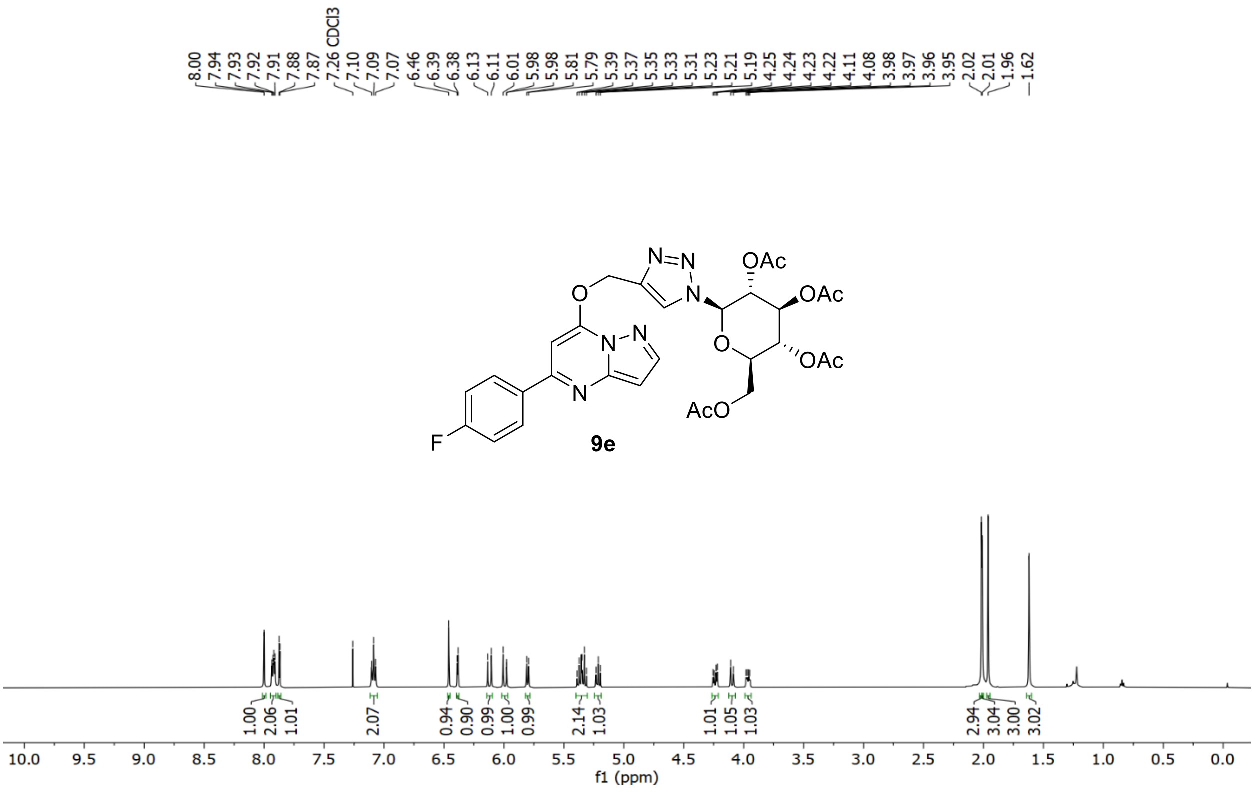

^{13}C NMR (126 MHz, $CDCl_3$)

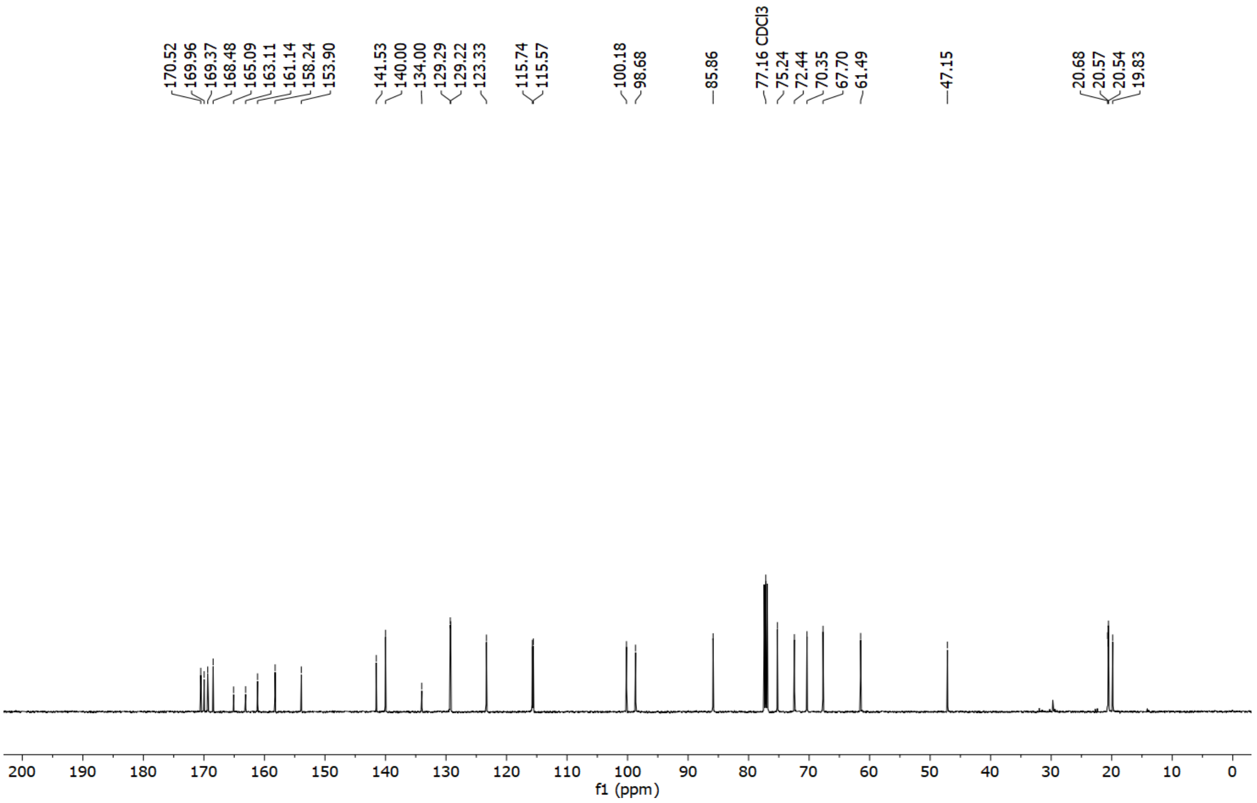

^{1}H-NMR (500 MHz, $CDCl_3$)

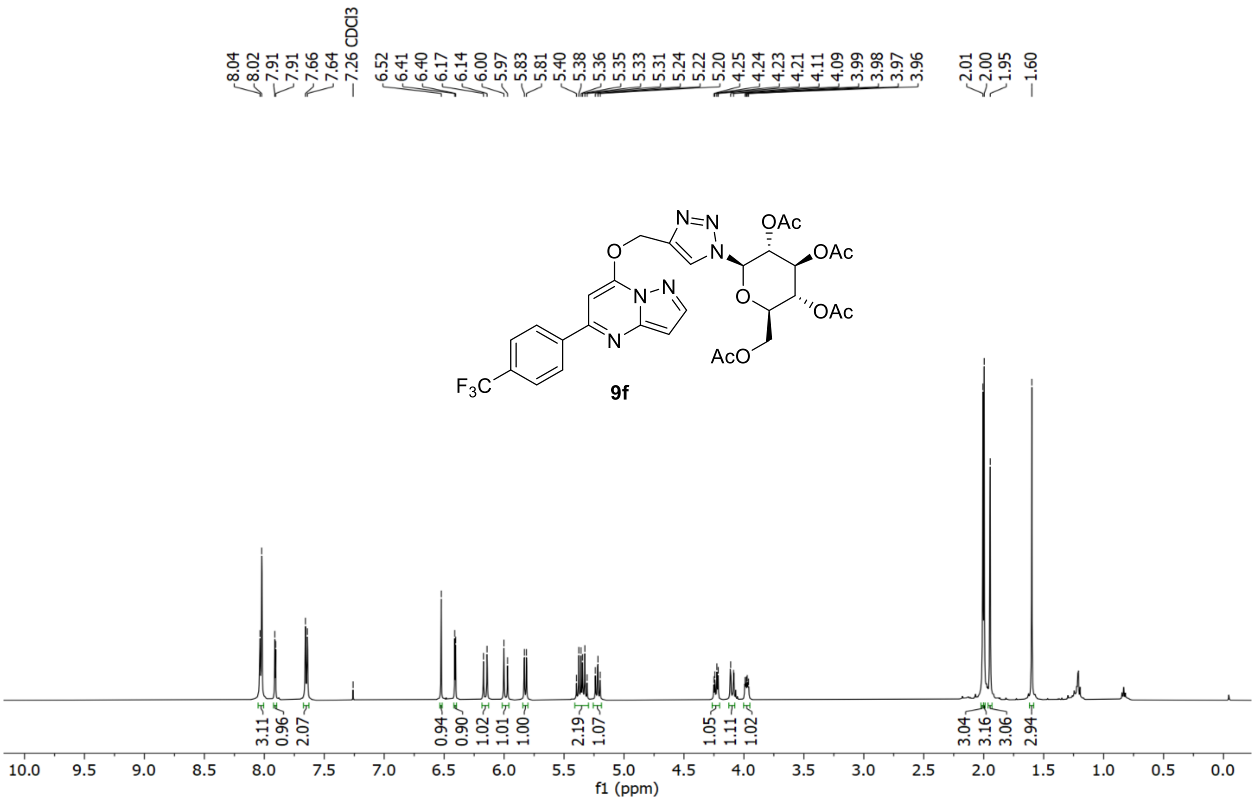

^{13}C NMR (126 MHz, $CDCl_3$)

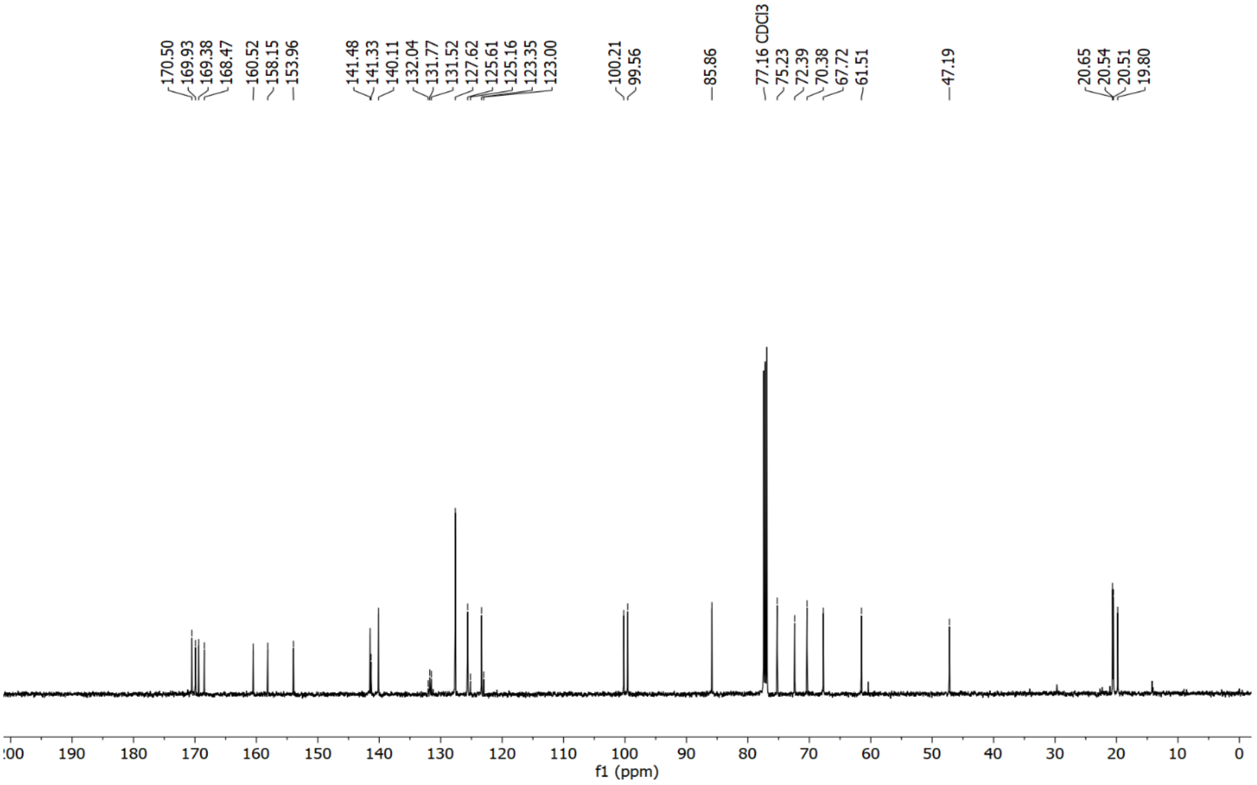

^{1}H-NMR (500 MHz, $CDCl_3$)

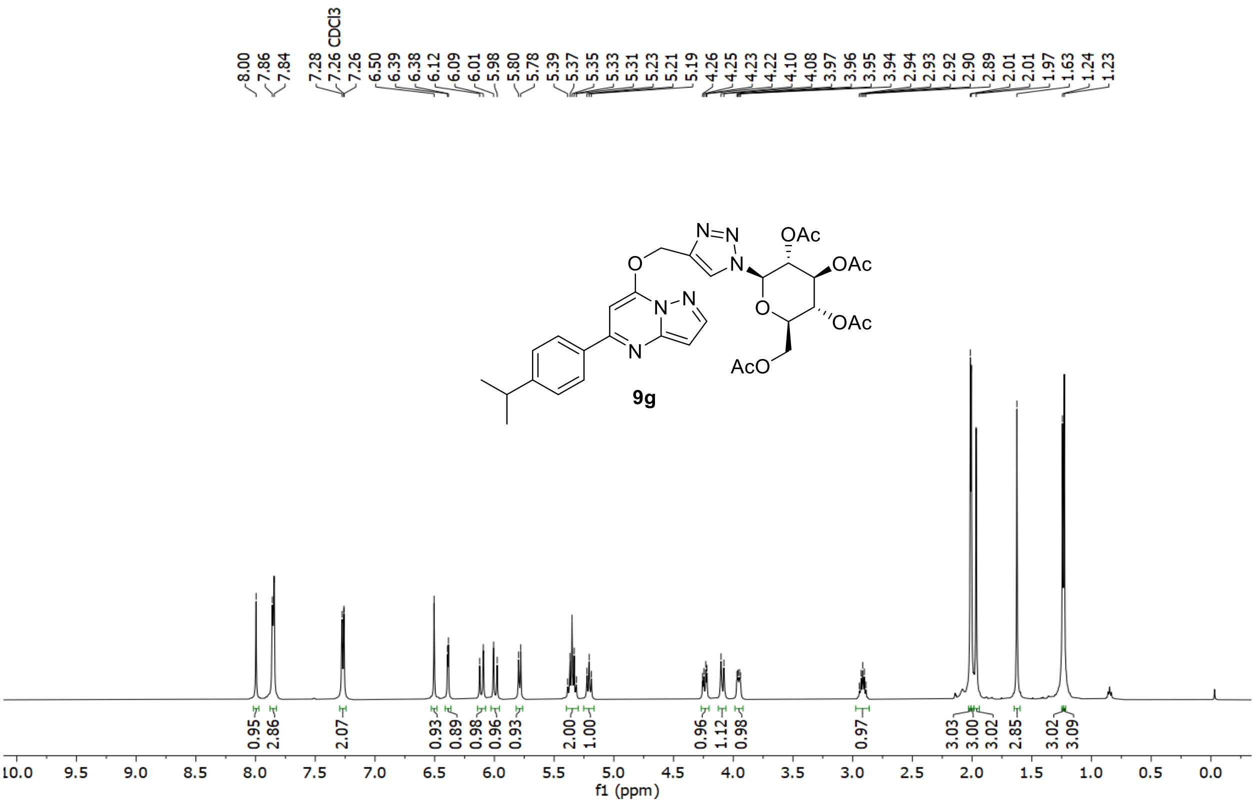

^{13}C NMR (126 MHz, $CDCl_3$)

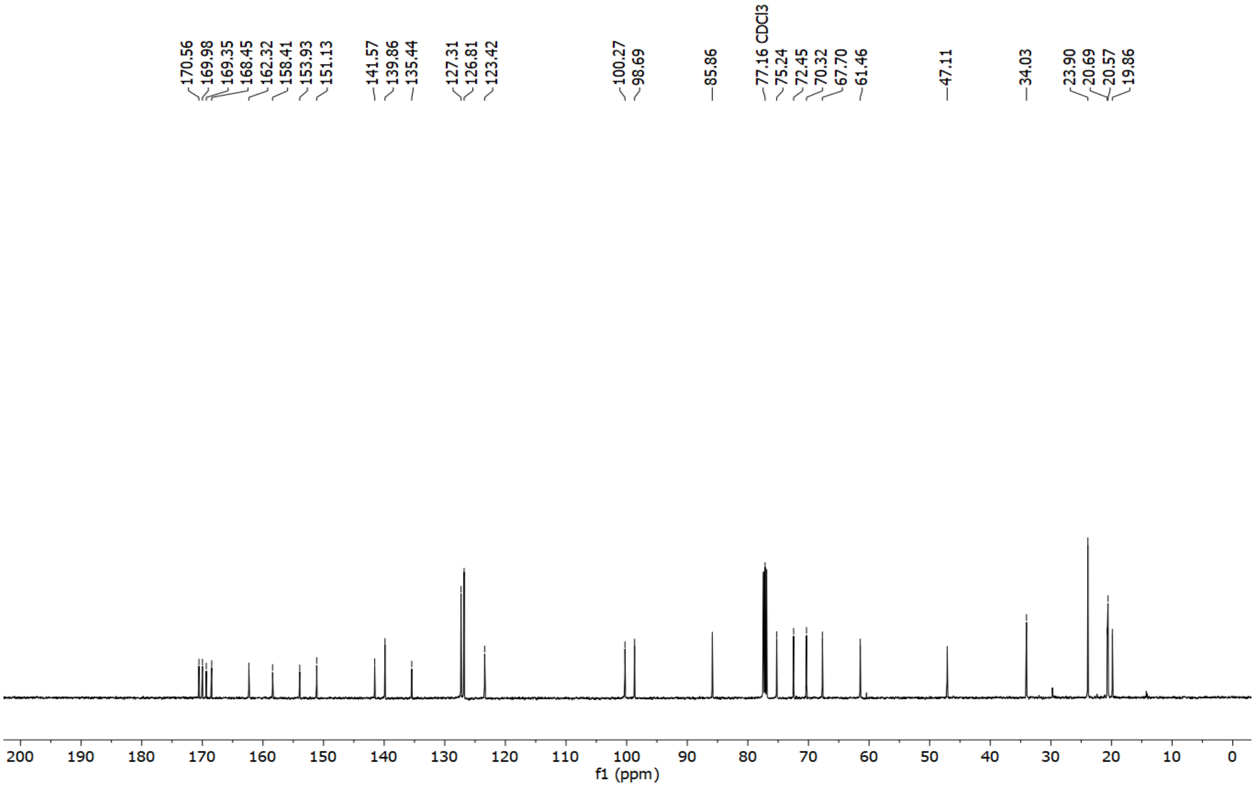

^{1}H-NMR (500 MHz, $CDCl_3$)

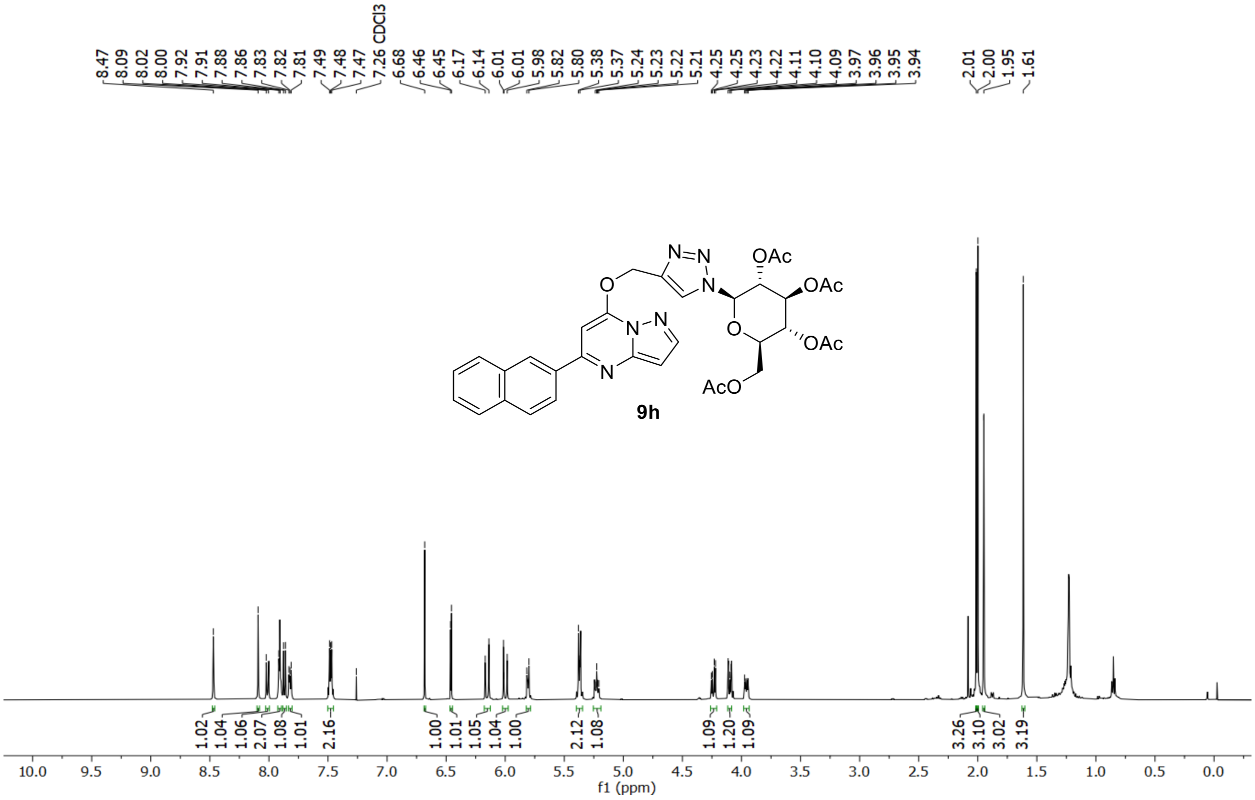

^{13}C NMR (126 MHz, $CDCl_3$)

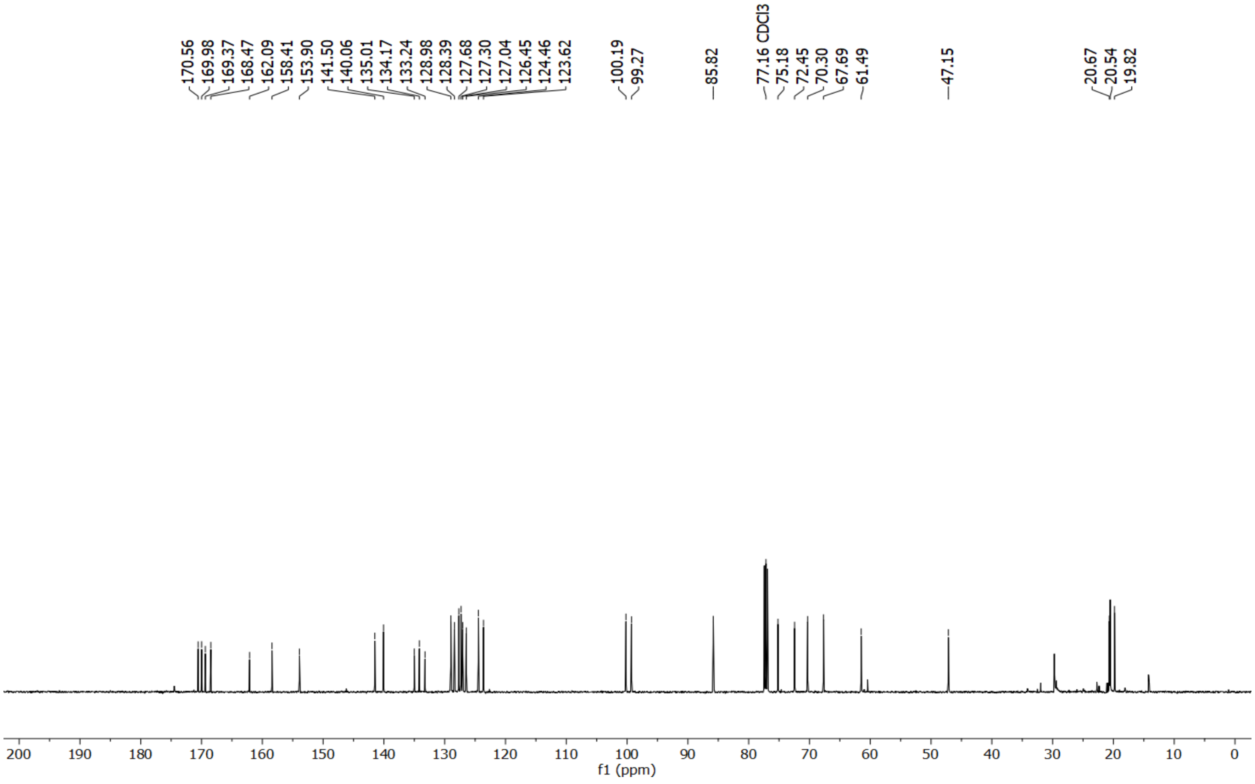

^{1}H-NMR (500 MHz, $CDCl_3$)

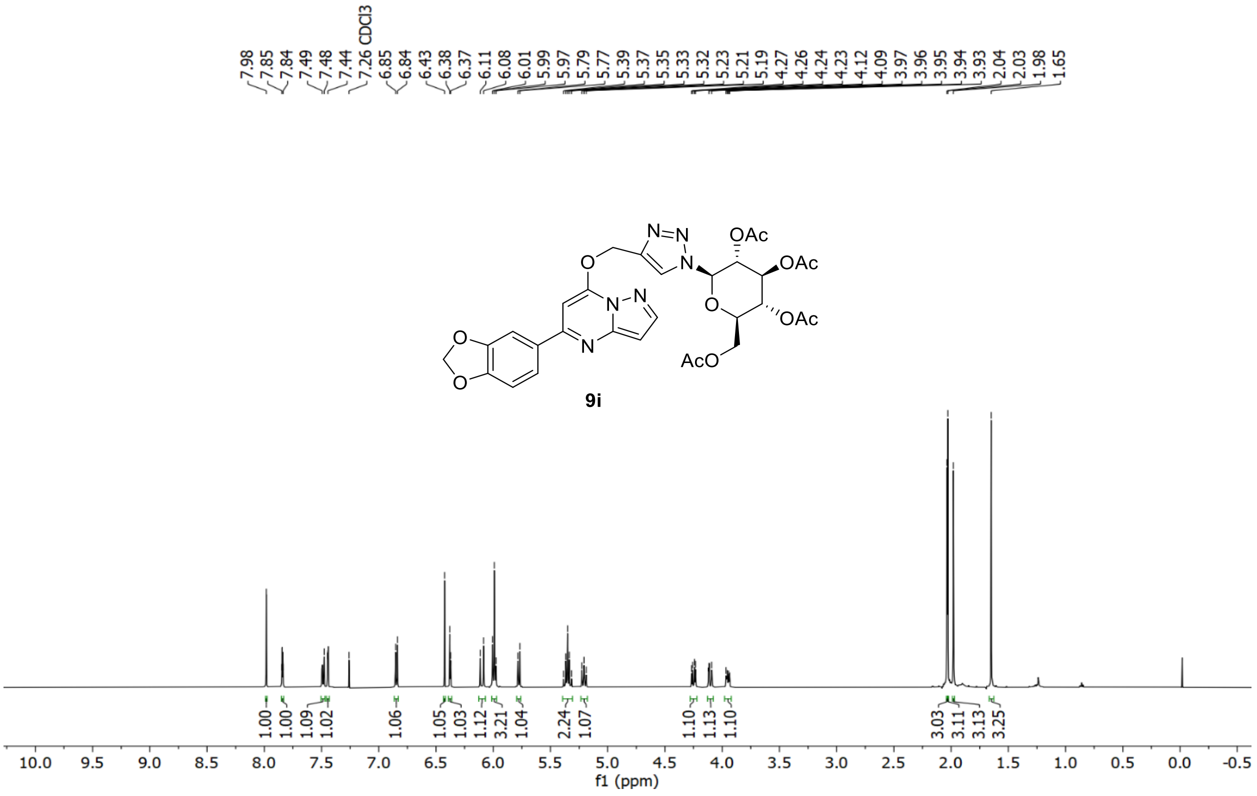

^{13}C NMR (126 MHz, $CDCl_3$)

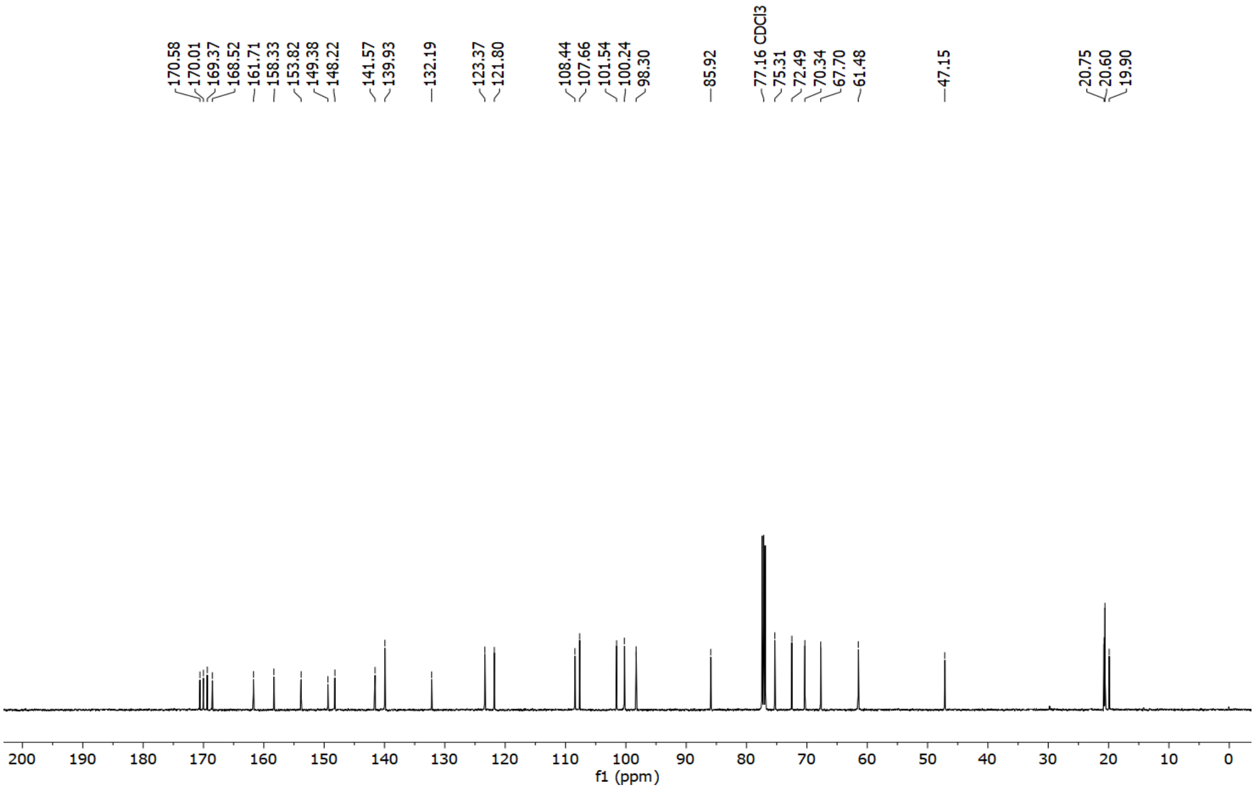

^{1}H-NMR (500 MHz, CDCl$_3$)

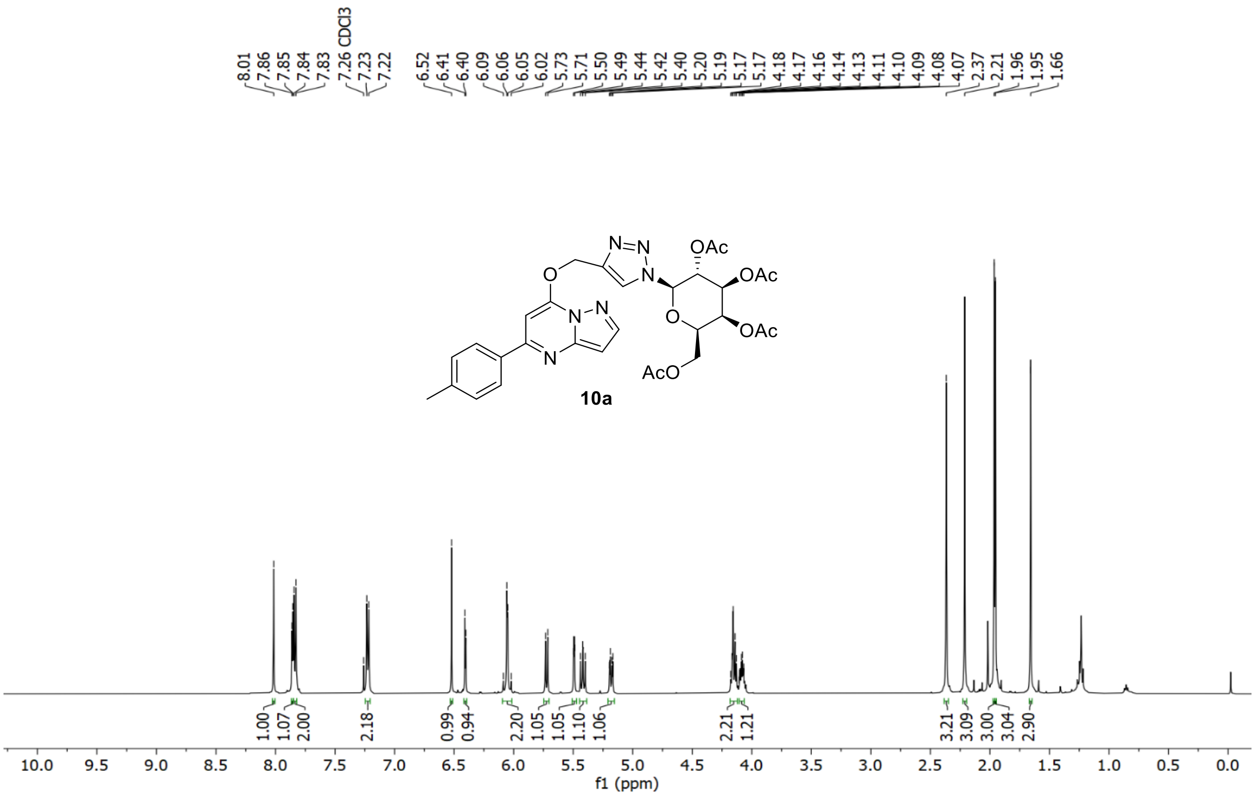

^{13}C NMR (126 MHz, CDCl$_3$)

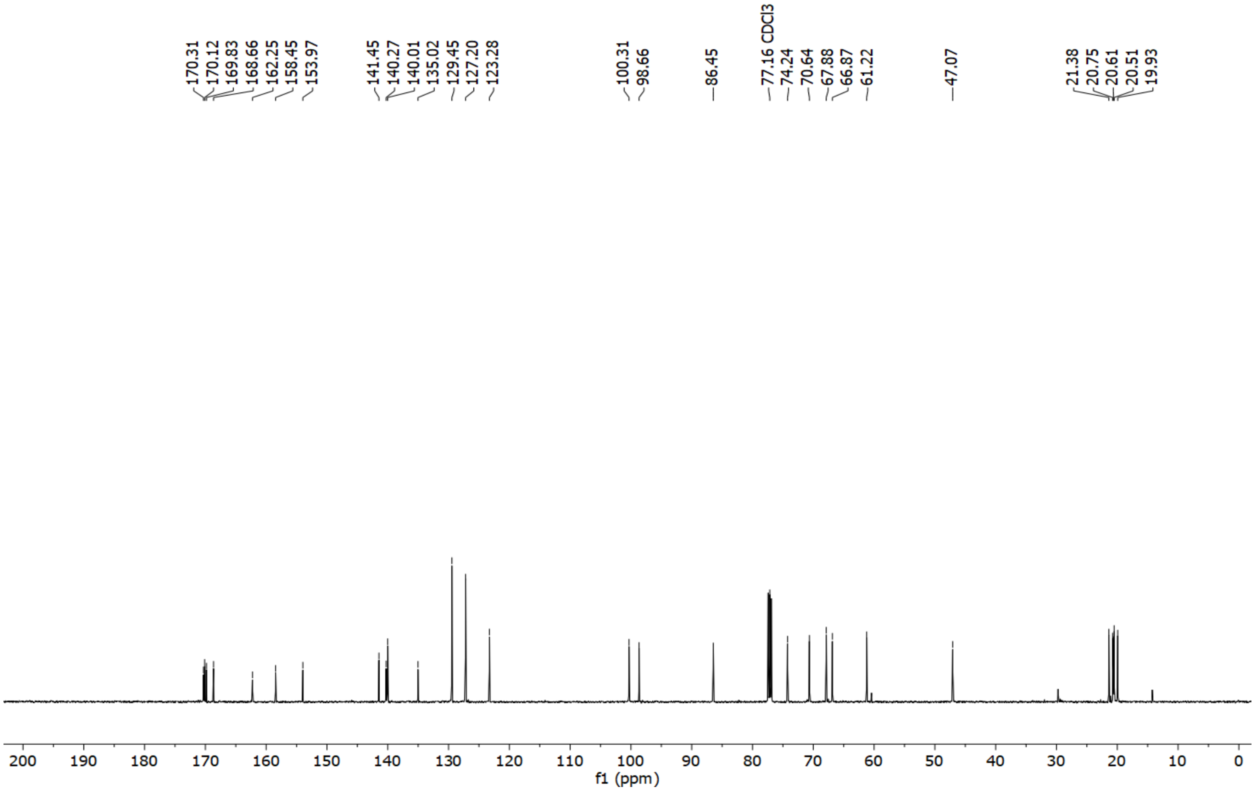

^{1}H-NMR (500 MHz, $CDCl_3$)

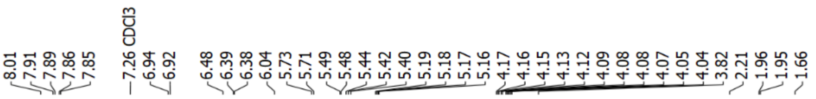

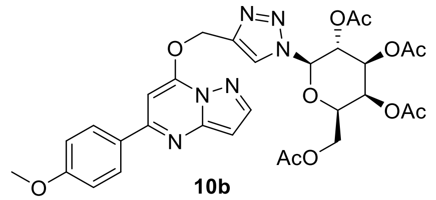

10b

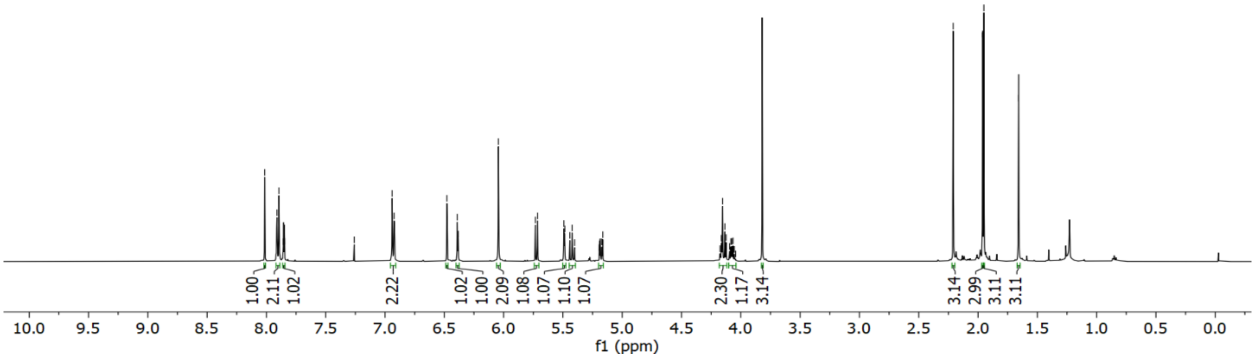

^{13}C NMR (126 MHz, $CDCl_3$)

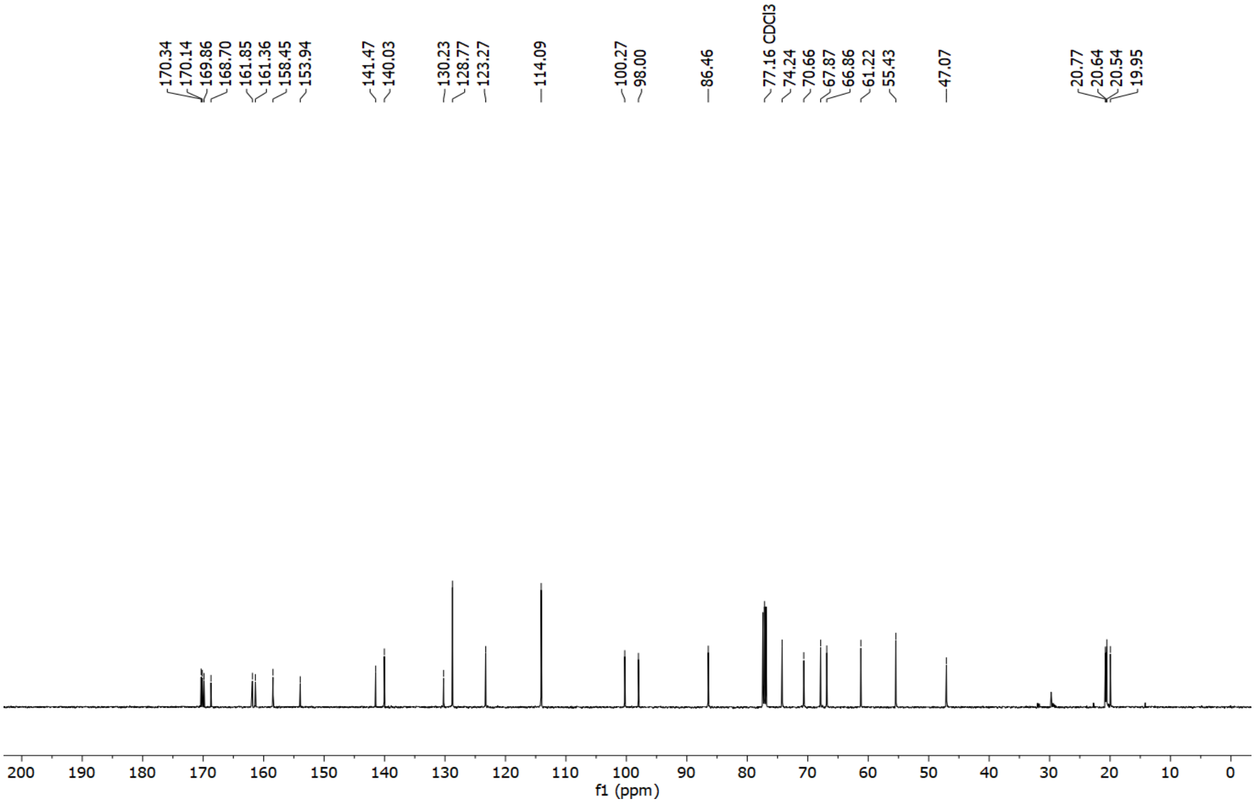

^{1}H-NMR (500 MHz, $CDCl_3$)

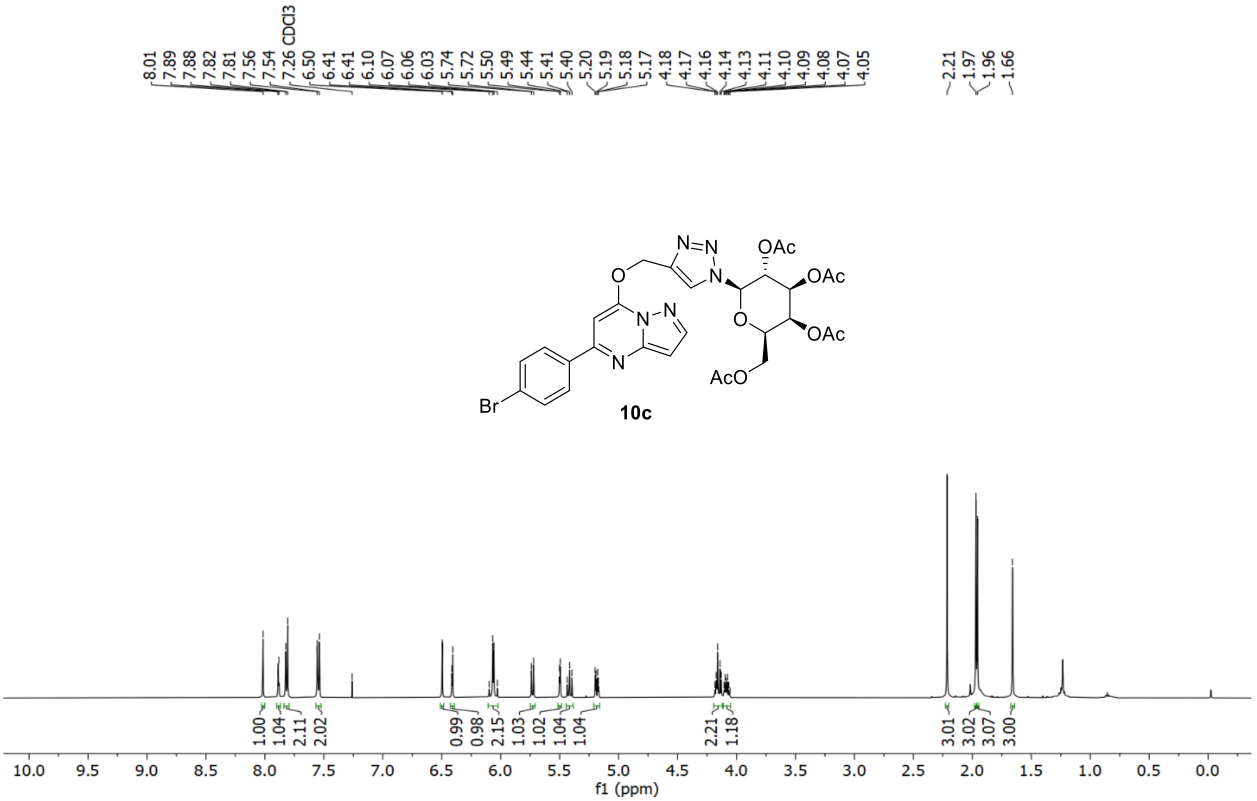

^{13}C NMR (126 MHz, $CDCl_3$)

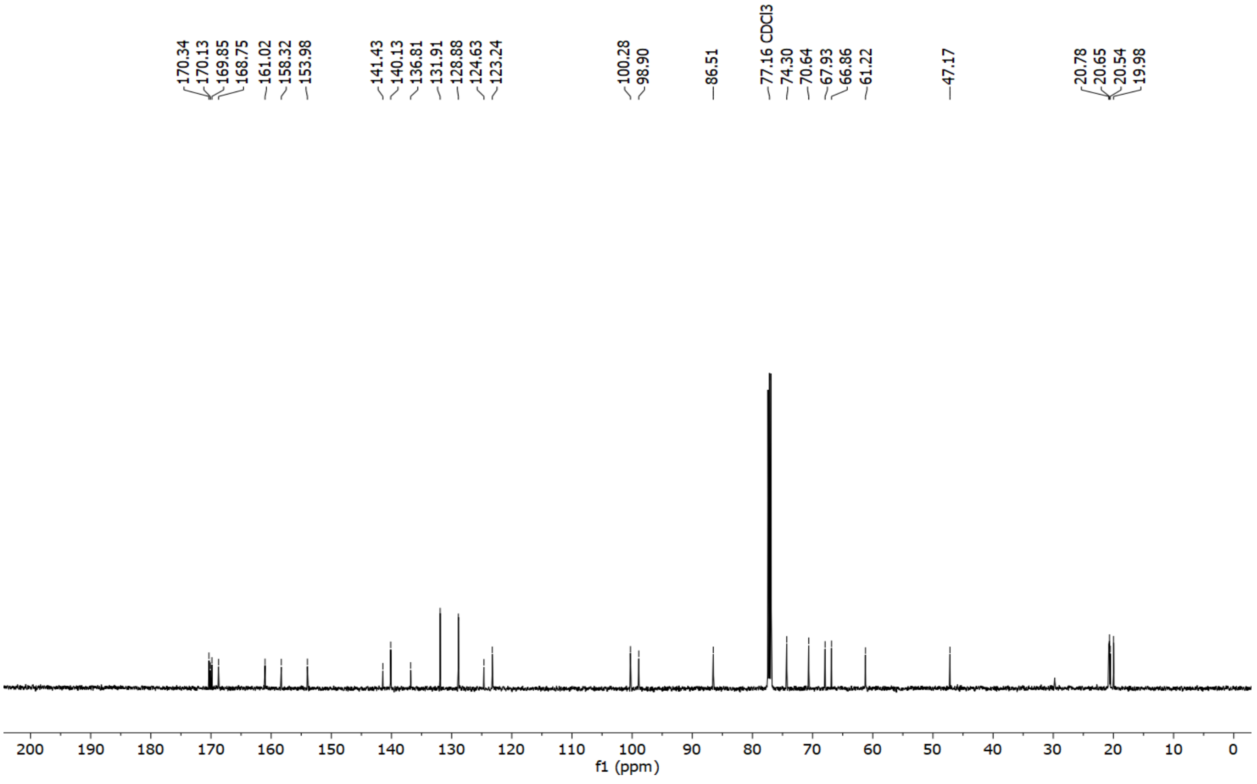

^{1}H-NMR (500 MHz, $CDCl_3$)

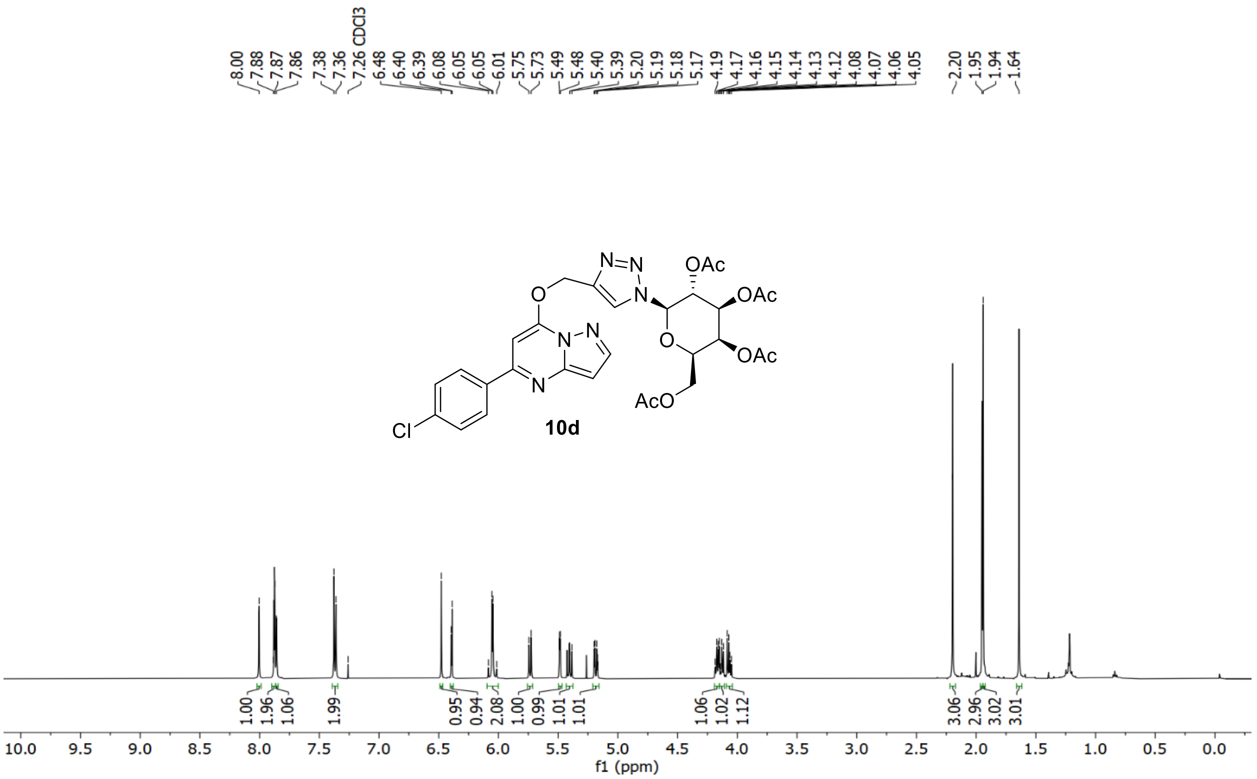

^{13}C NMR (126 MHz, $CDCl_3$)

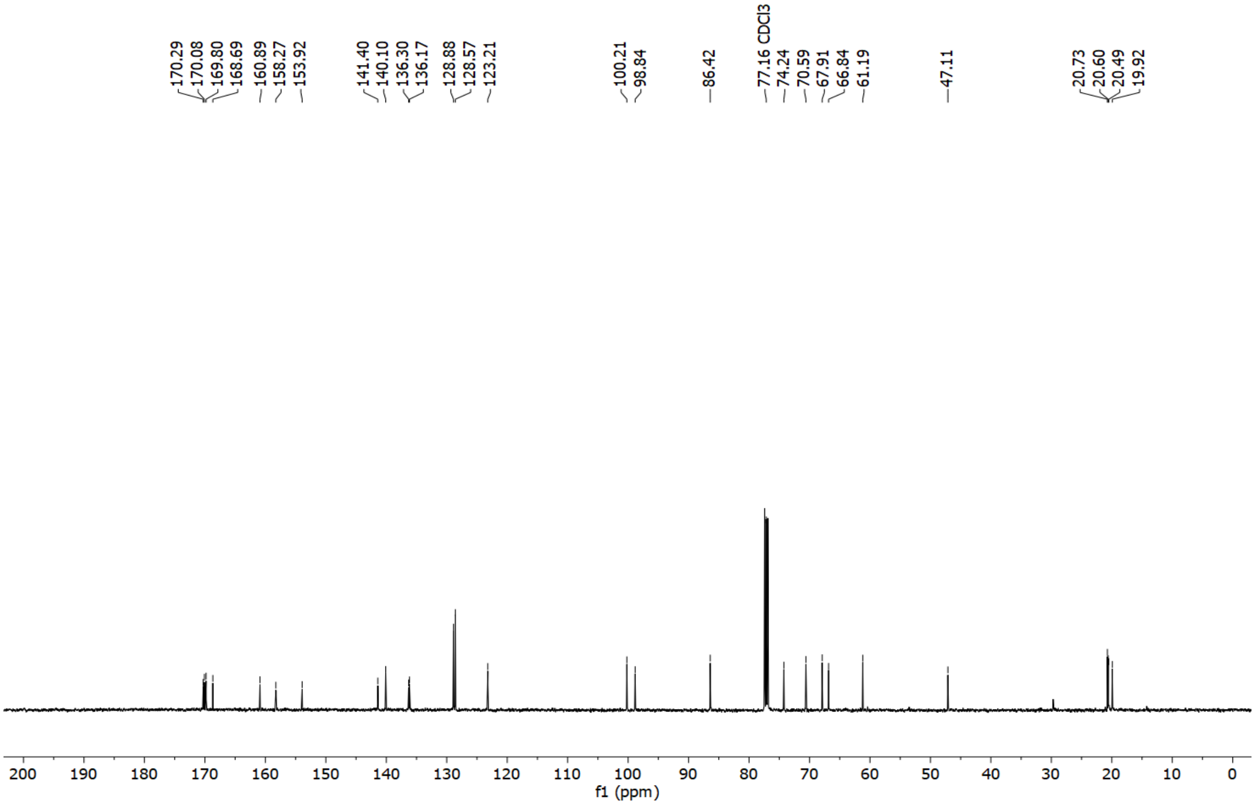

^{1}H-NMR (500 MHz, CDCl3)

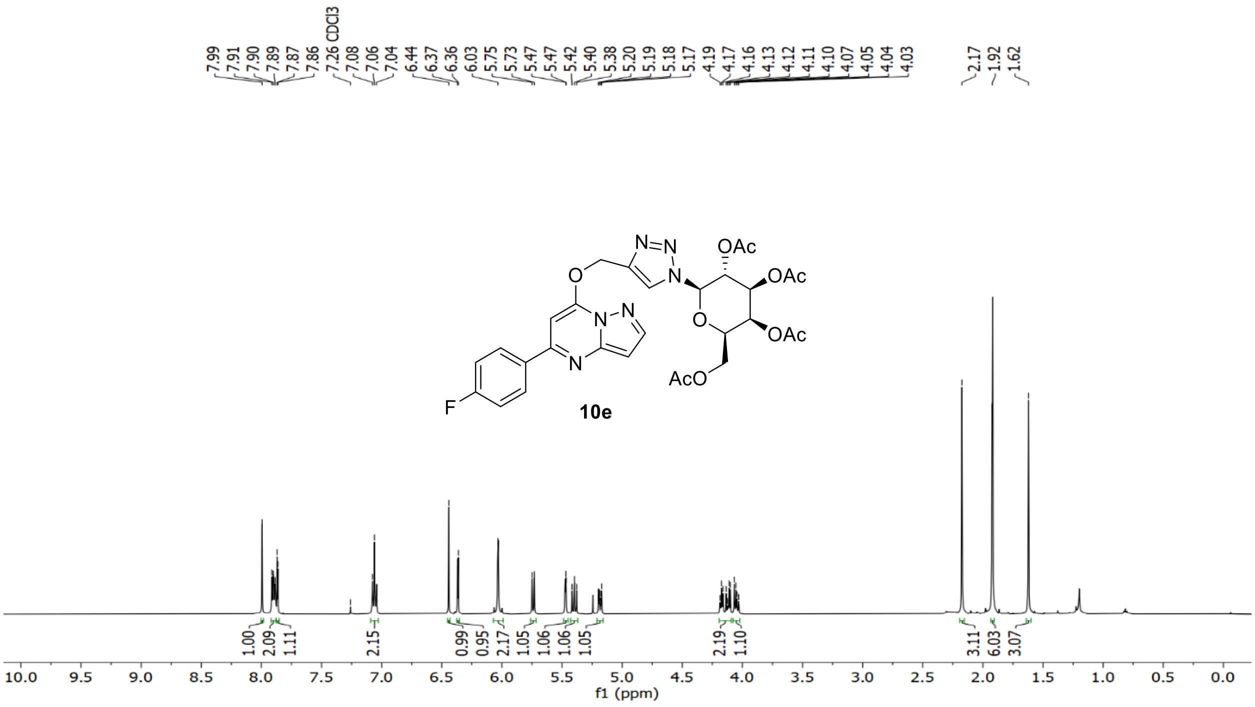

^{13}C NMR (126 MHz, CDCl3)

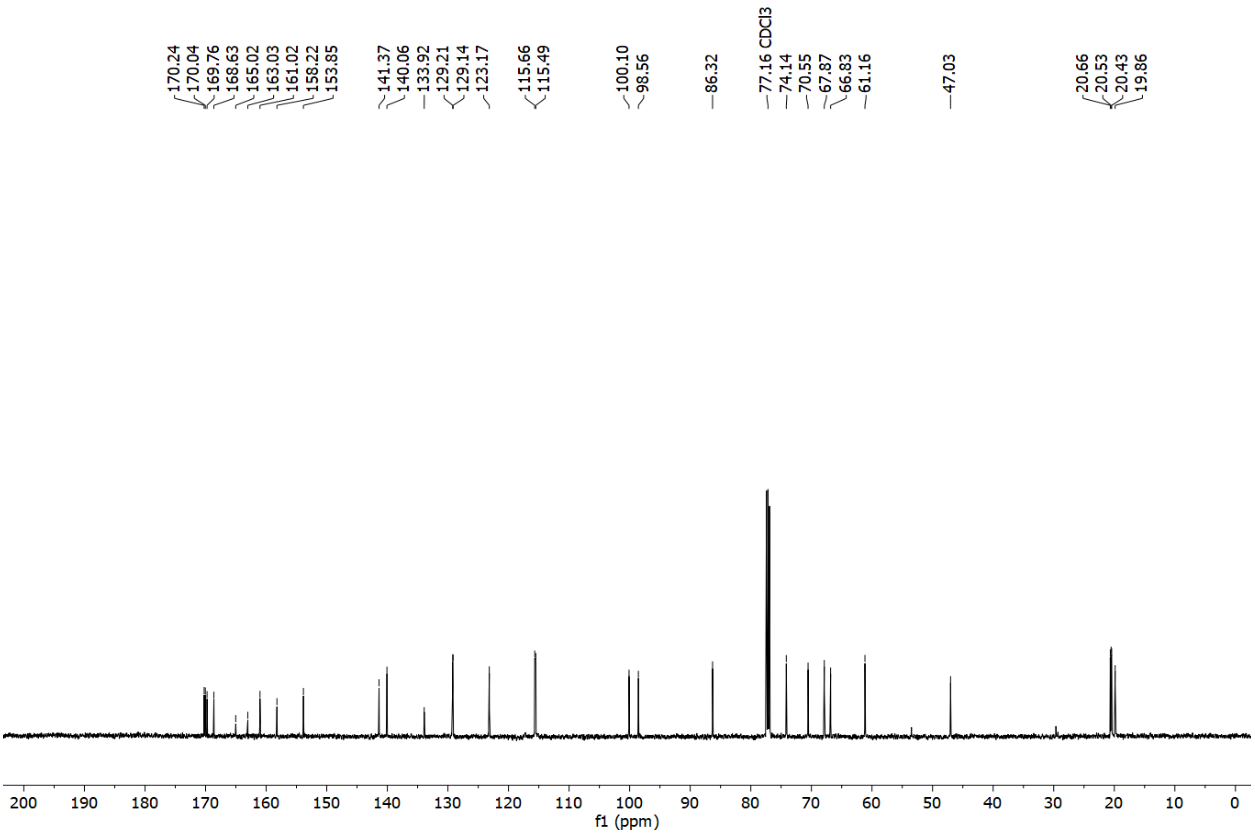

^{1}H-NMR (500 MHz, $CDCl_3$)

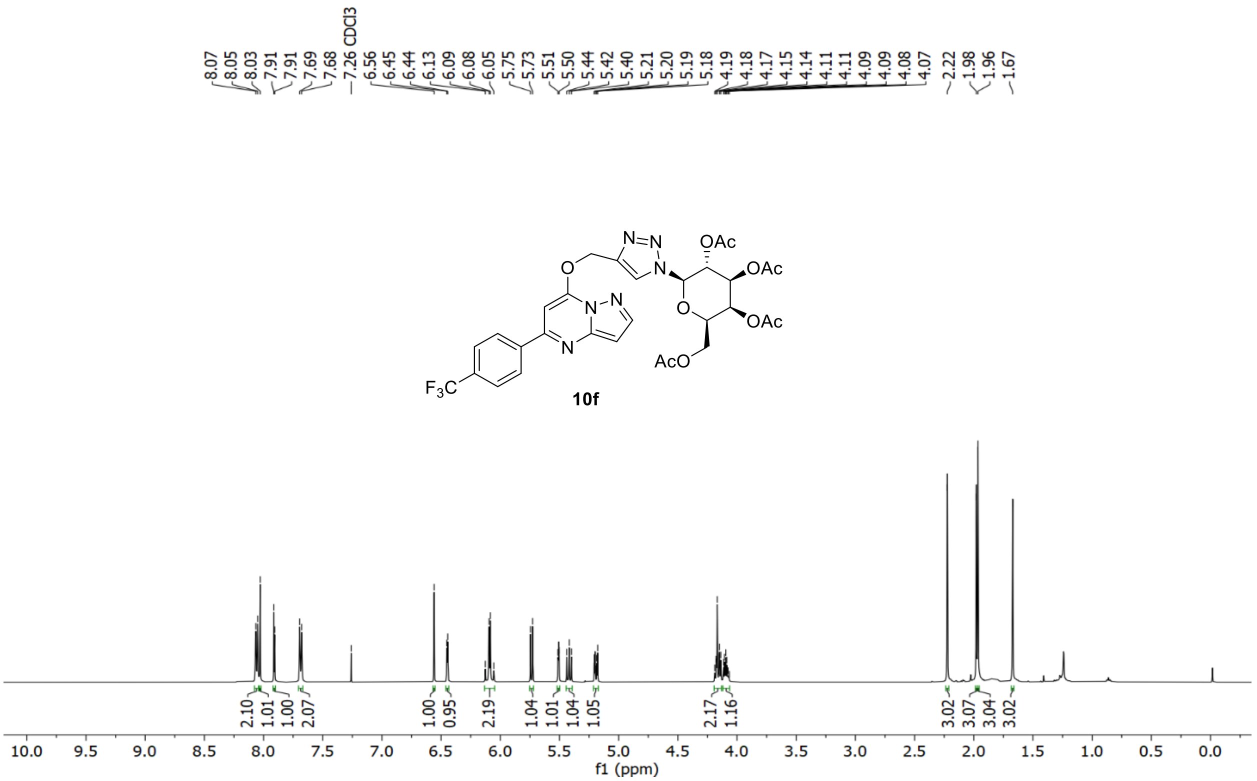

^{13}C NMR (126 MHz, $CDCl_3$)

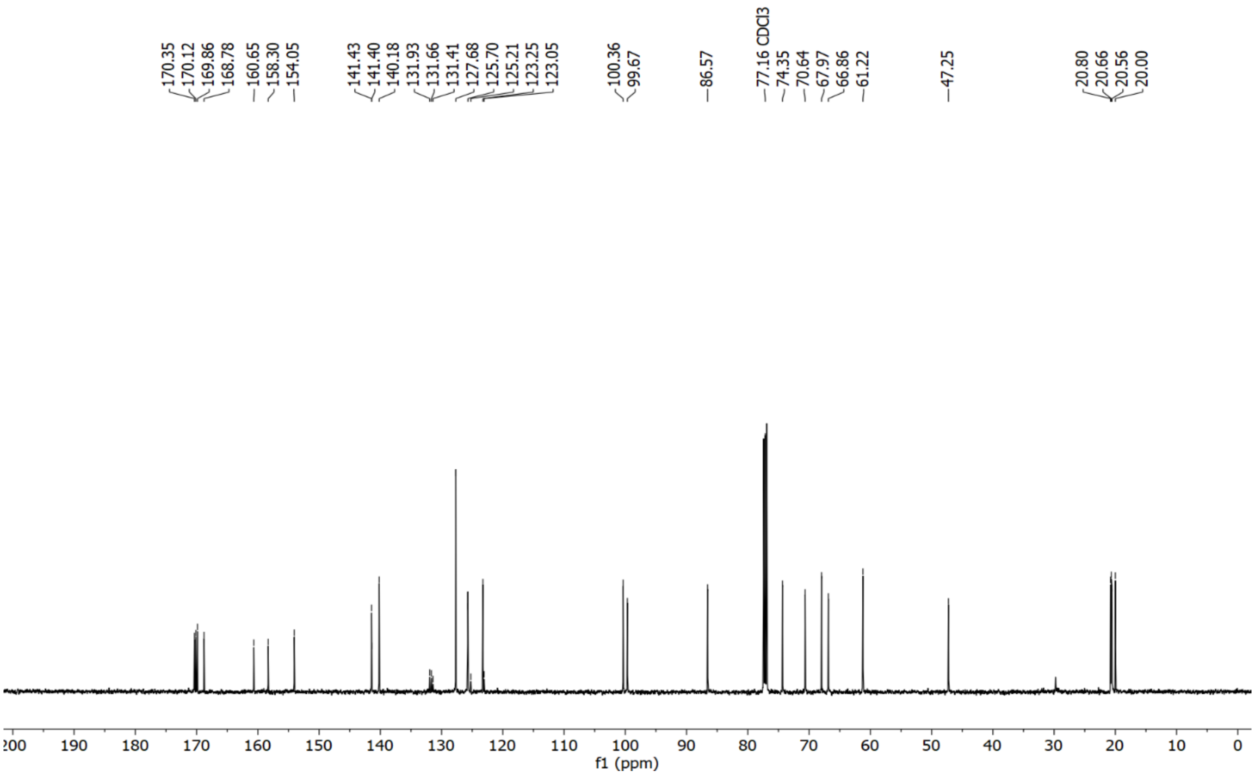

^{1}H-NMR (500 MHz, $CDCl_3$)

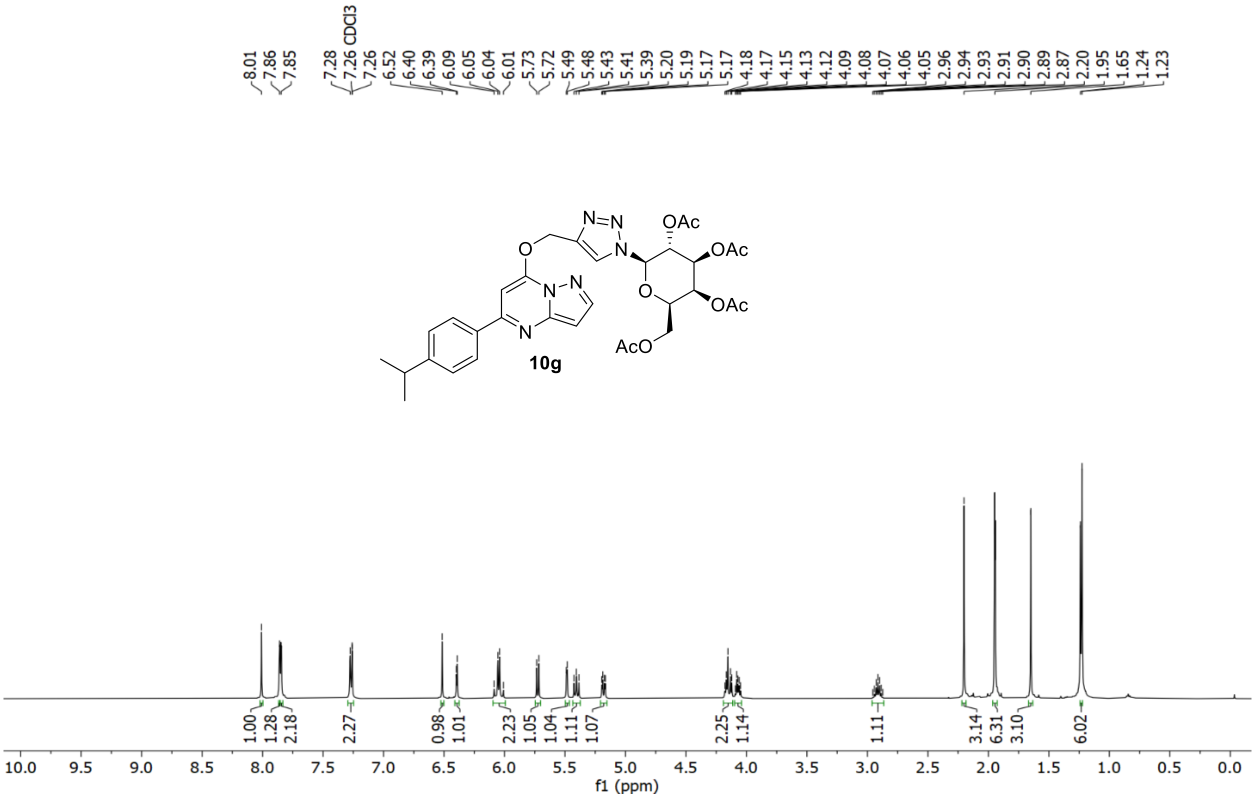

^{13}C NMR (126 MHz, $CDCl_3$)

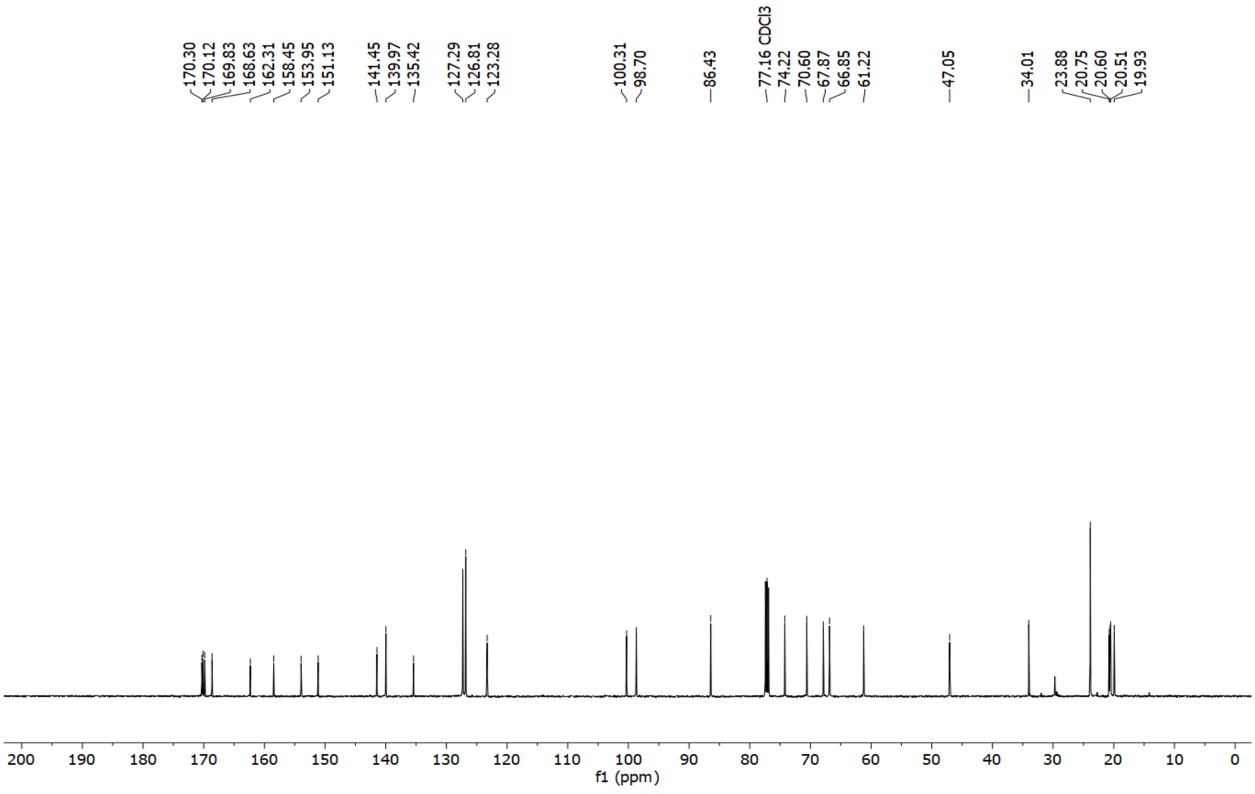

^{1}H-NMR (500 MHz, CDCl₃)

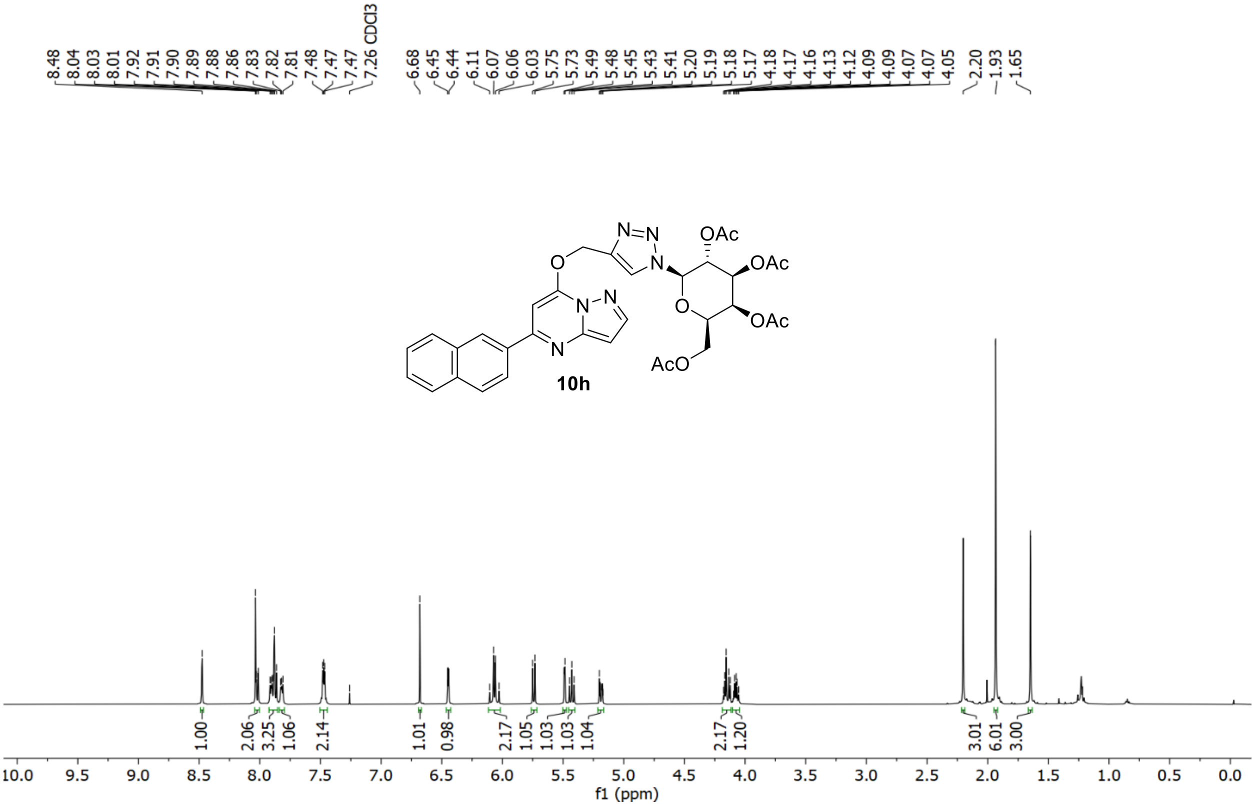

^{13}C NMR (126 MHz, CDCl₃)

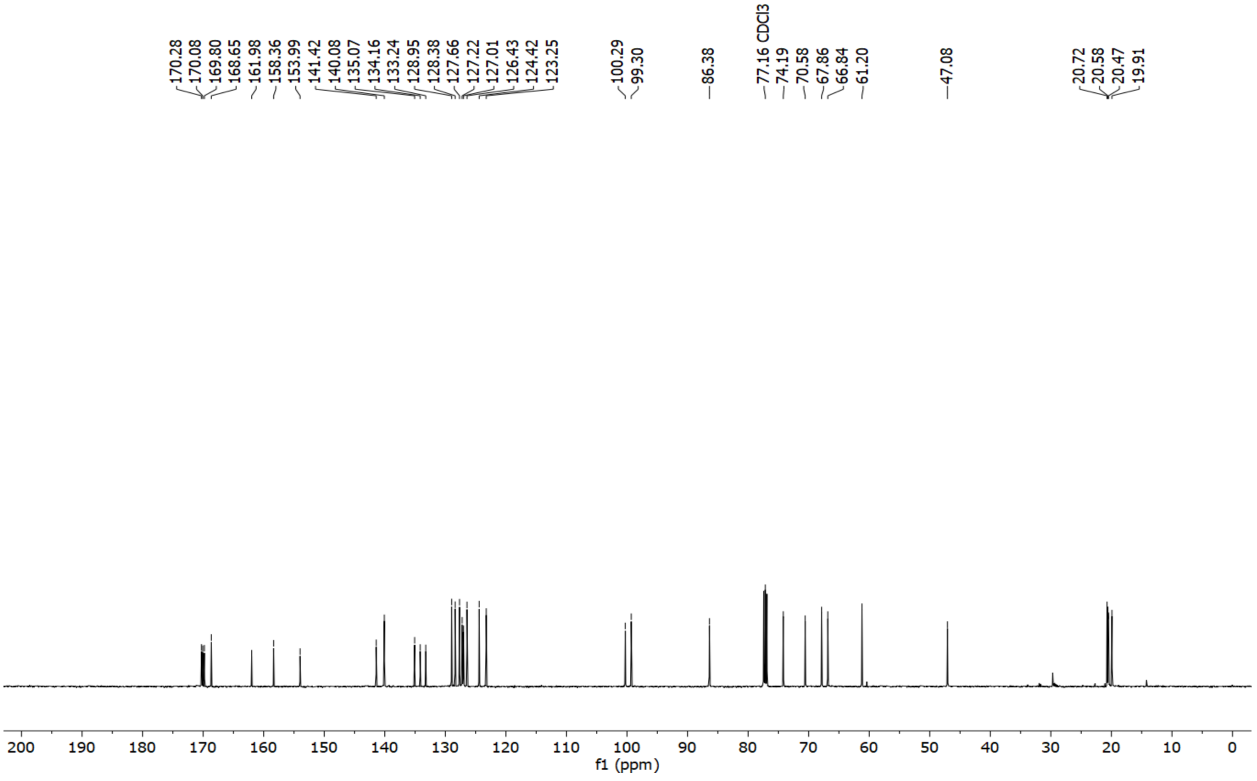

^{1}H-NMR (500 MHz, $CDCl_3$)

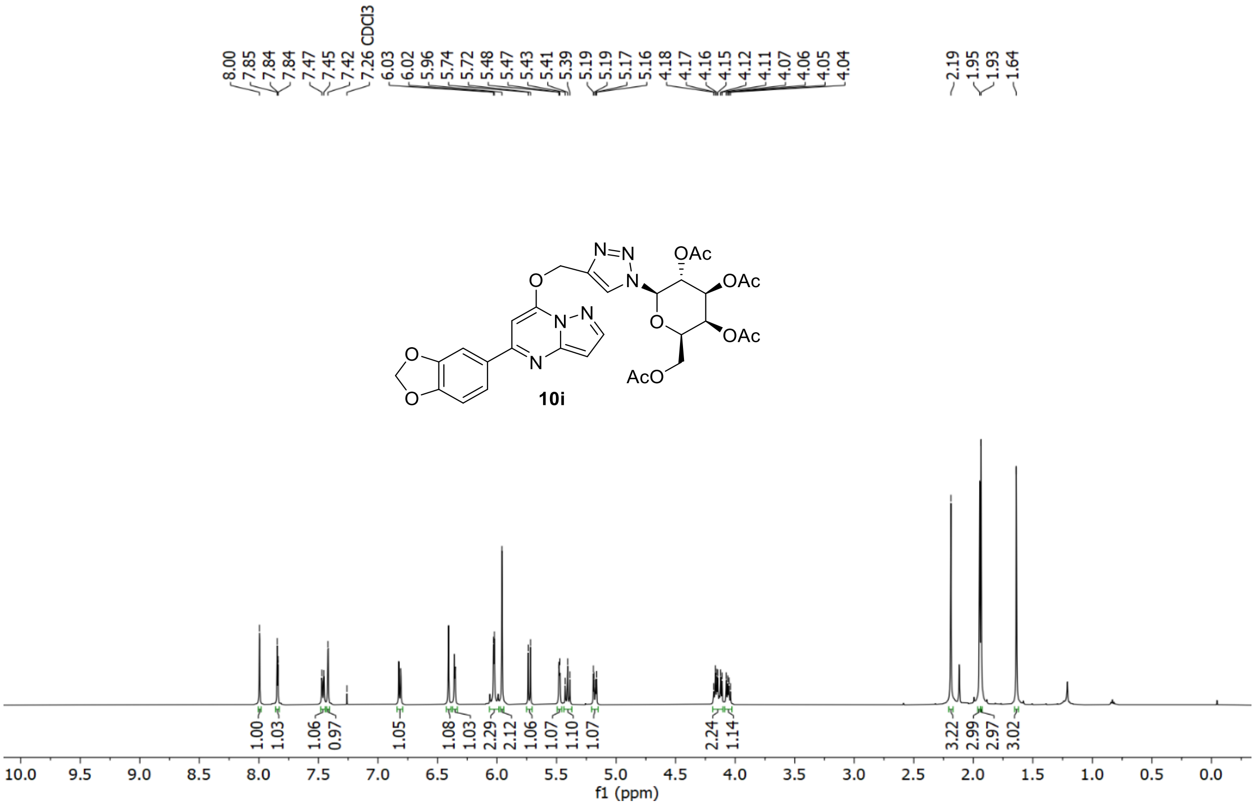

^{13}C NMR (126 MHz, $CDCl_3$)

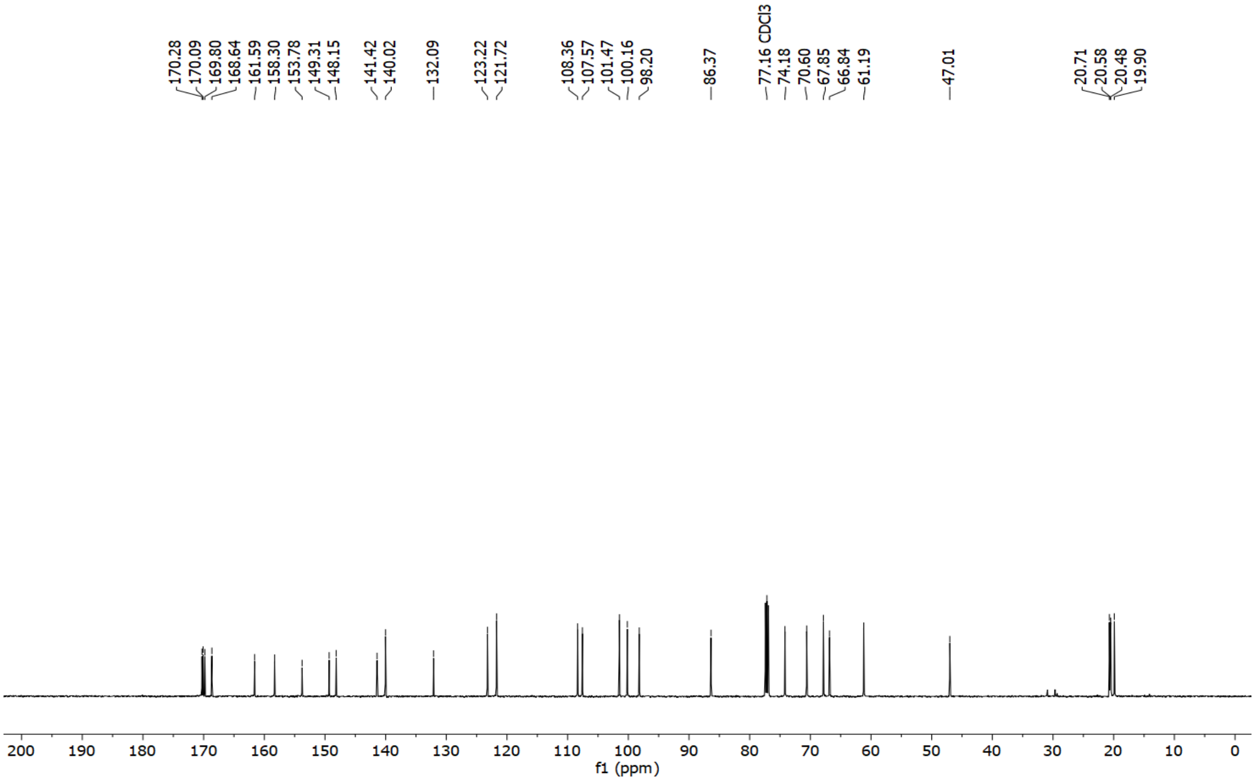

^{1}H-NMR (500 MHz, $CDCl_3$)

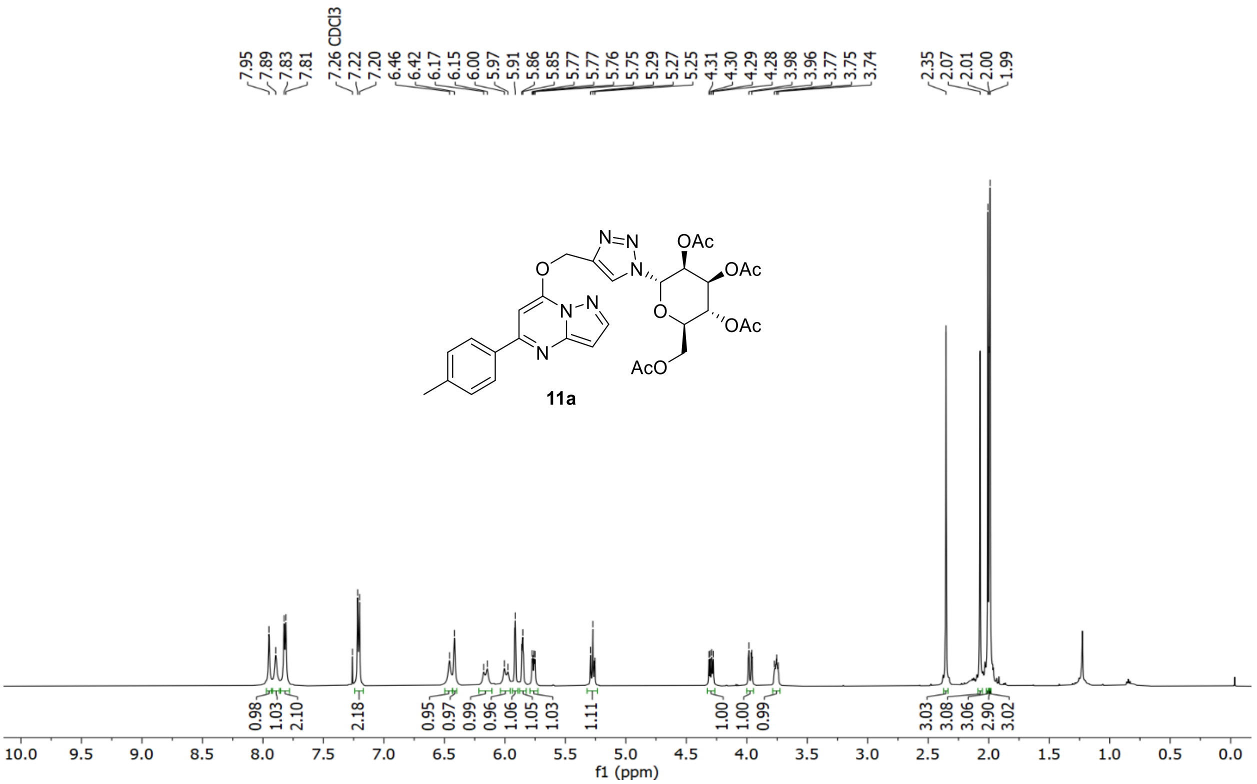

^{13}C NMR (126 MHz, $CDCl_3$)

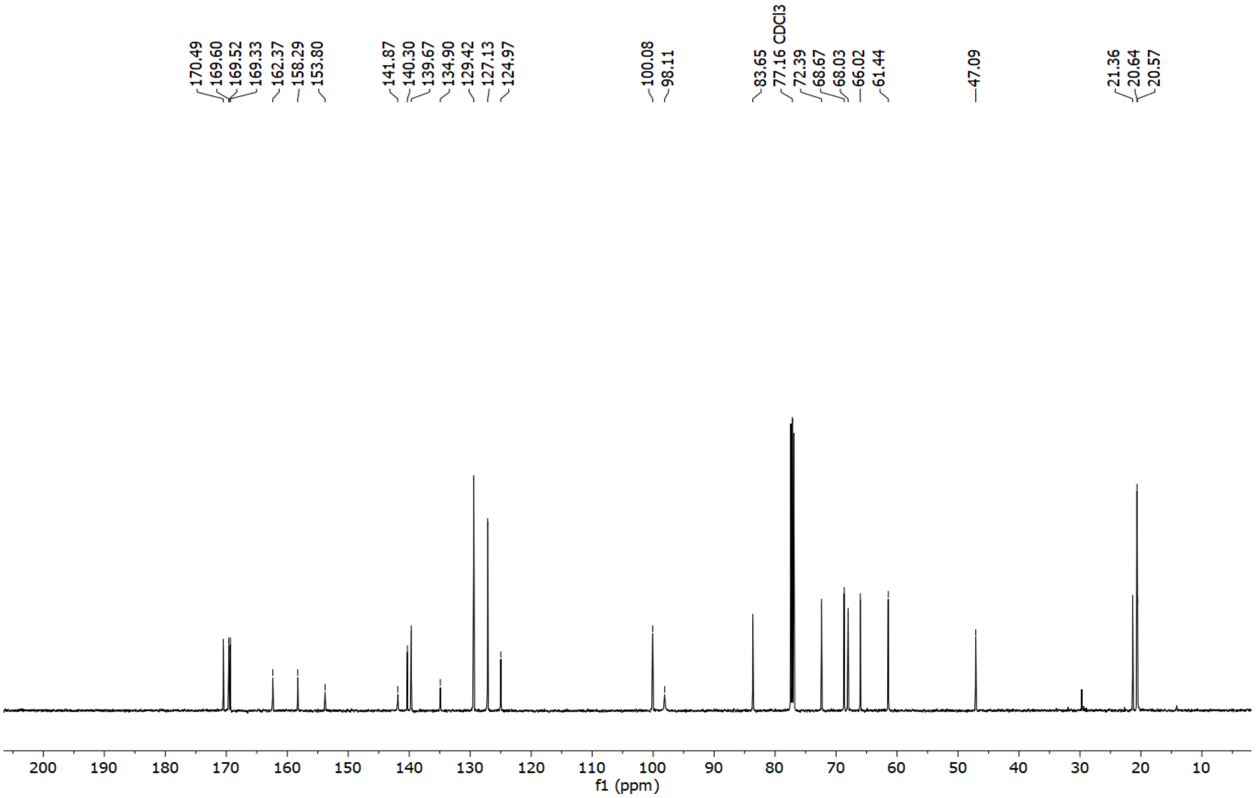

^{1}H-NMR (500 MHz, $CDCl_3$)

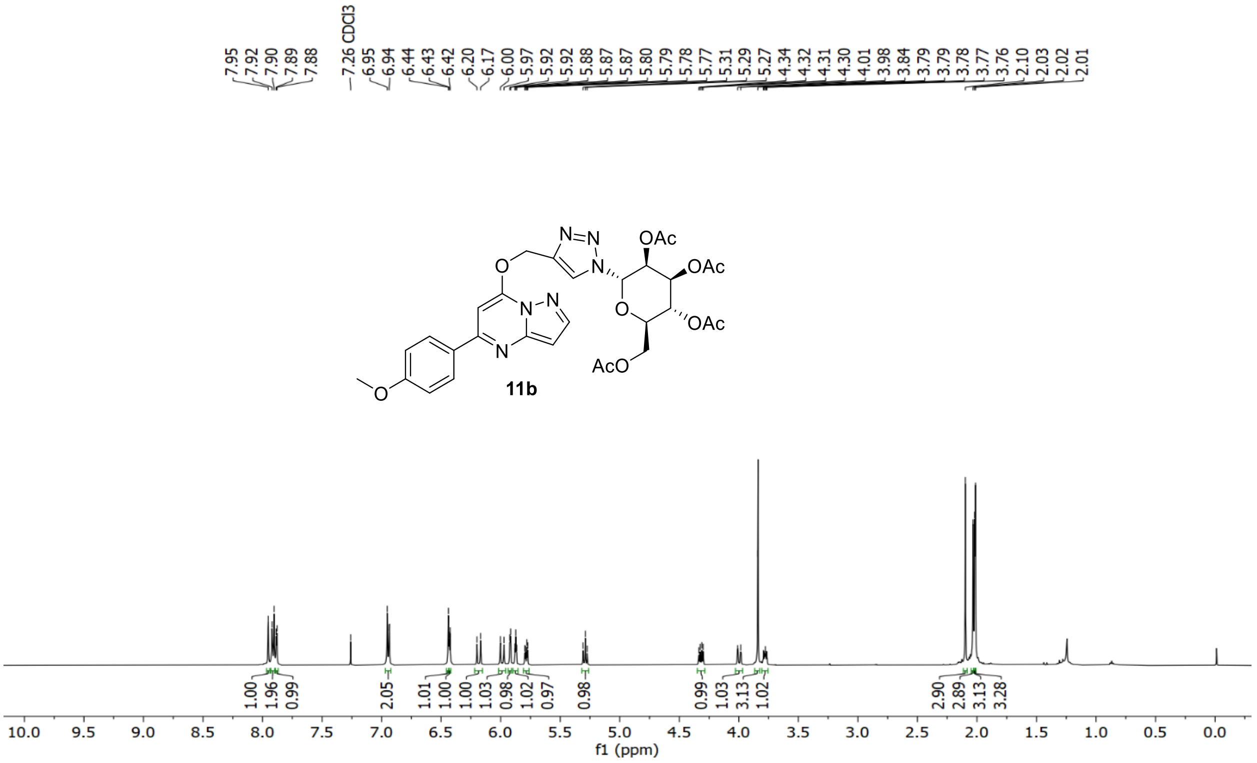

^{13}C NMR (126 MHz, $CDCl_3$)

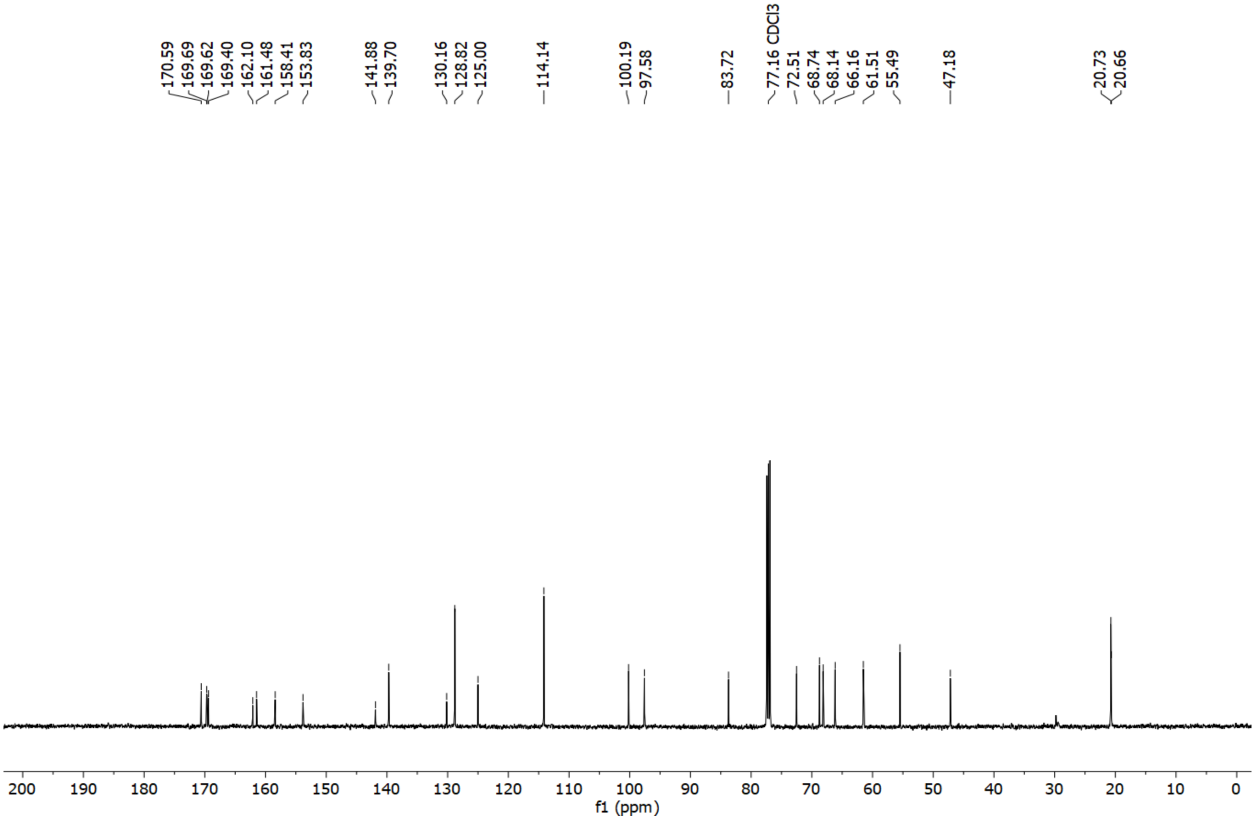

^{1}H-NMR (500 MHz, $CDCl_3$)

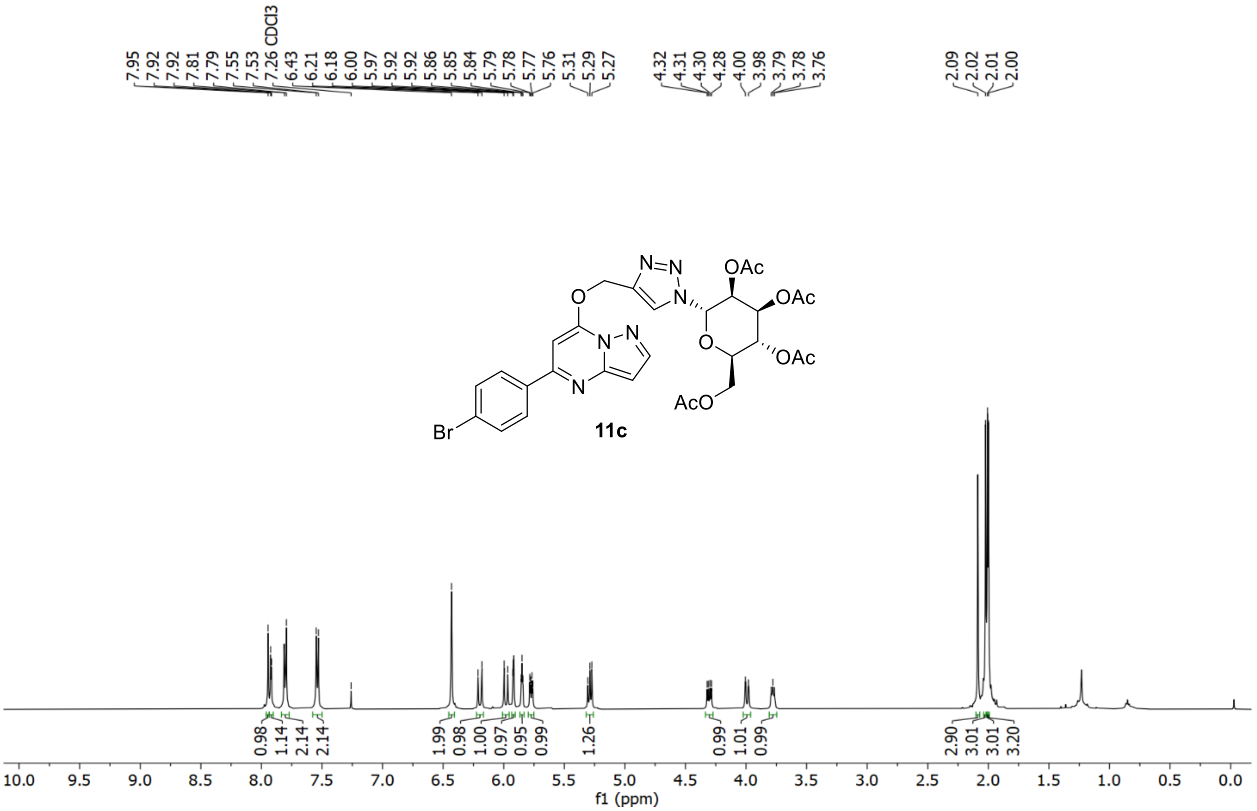

^{13}C NMR (126 MHz, $CDCl_3$)

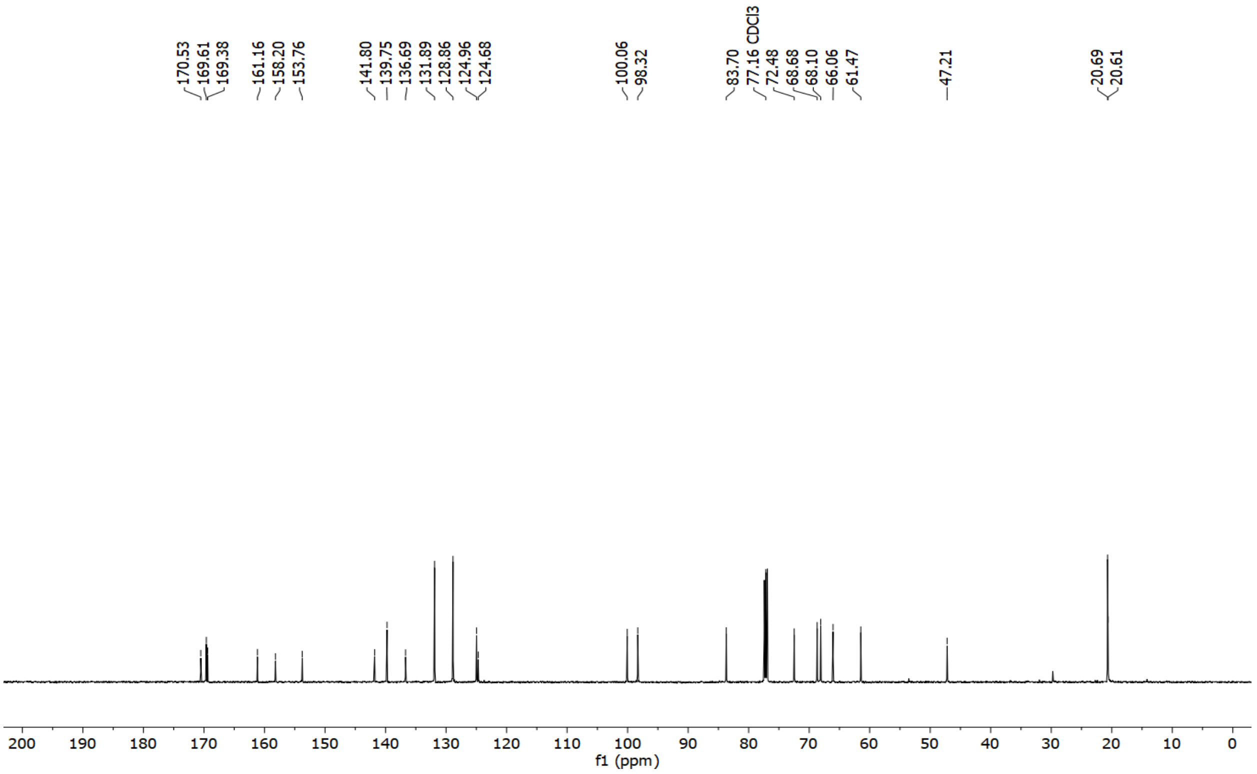

^{1}H-NMR (500 MHz, $CDCl_3$)

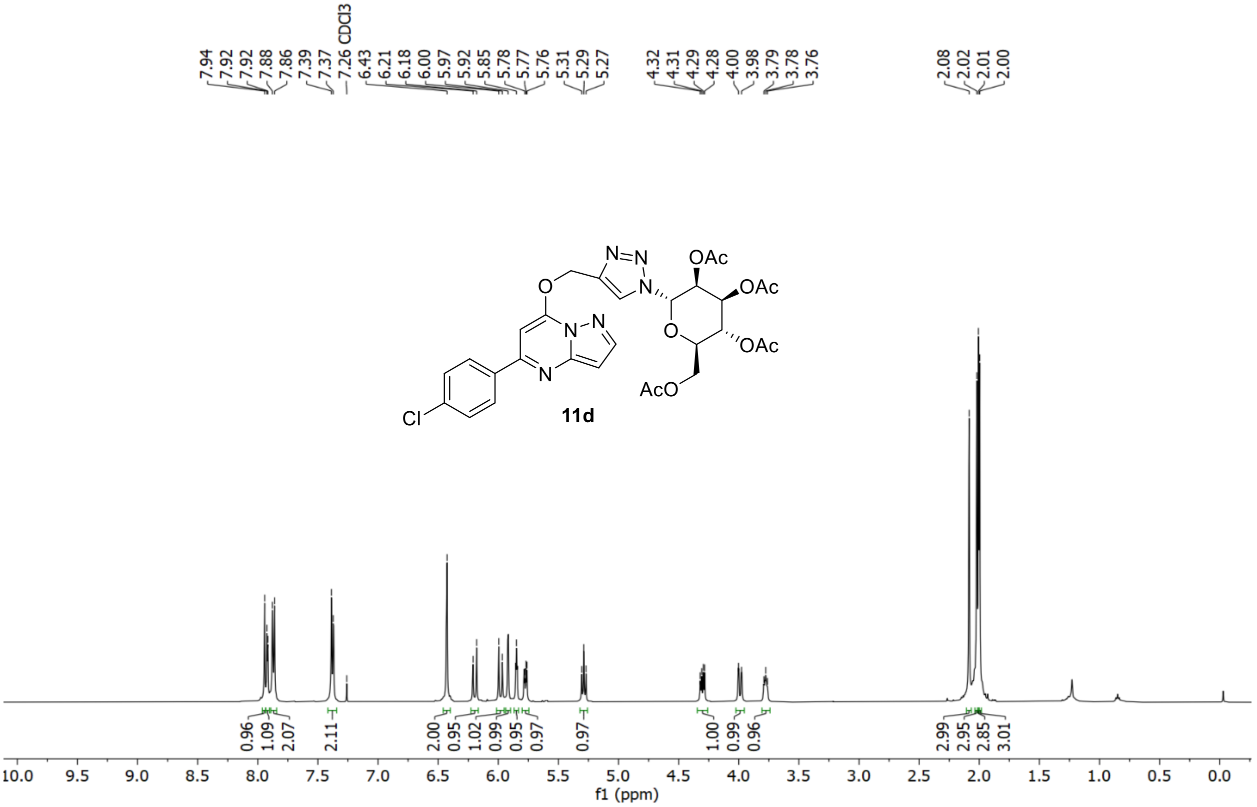

^{13}C NMR (126 MHz, $CDCl_3$)

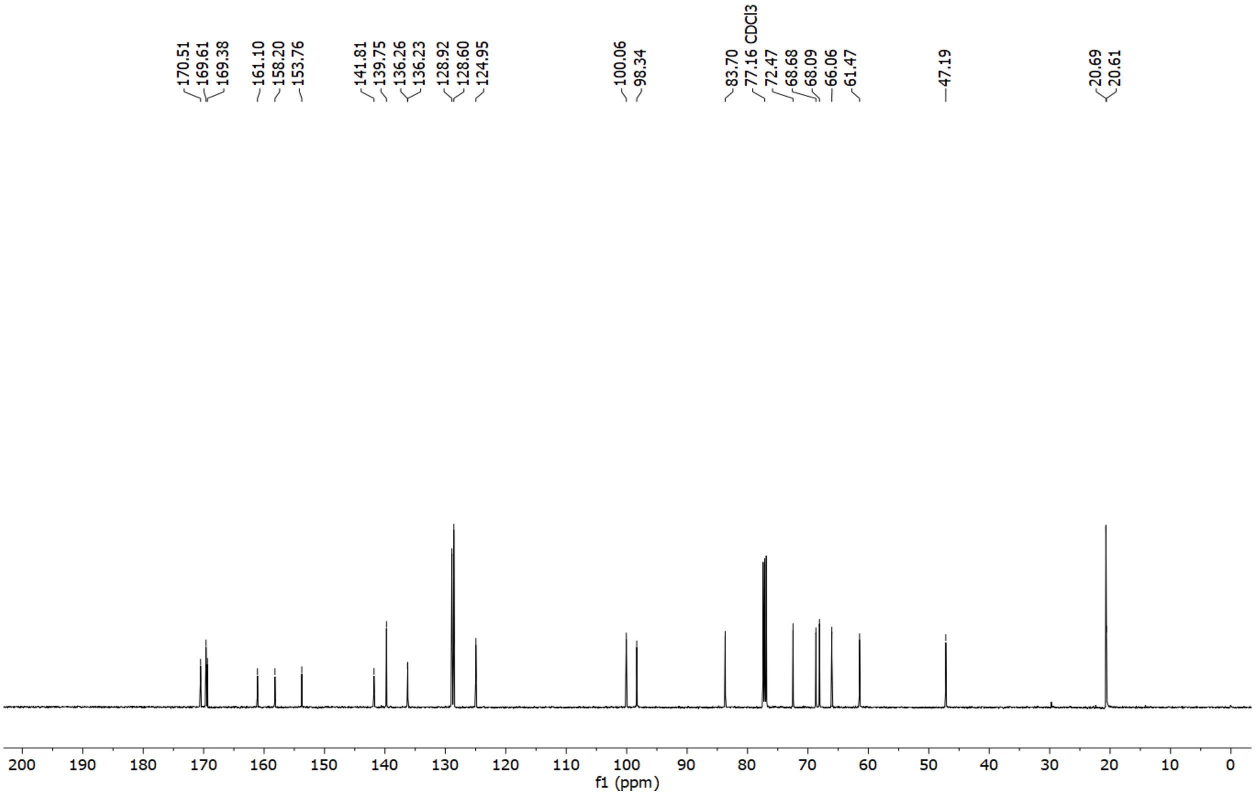

^{1}H-NMR (500 MHz, $CDCl_3$)

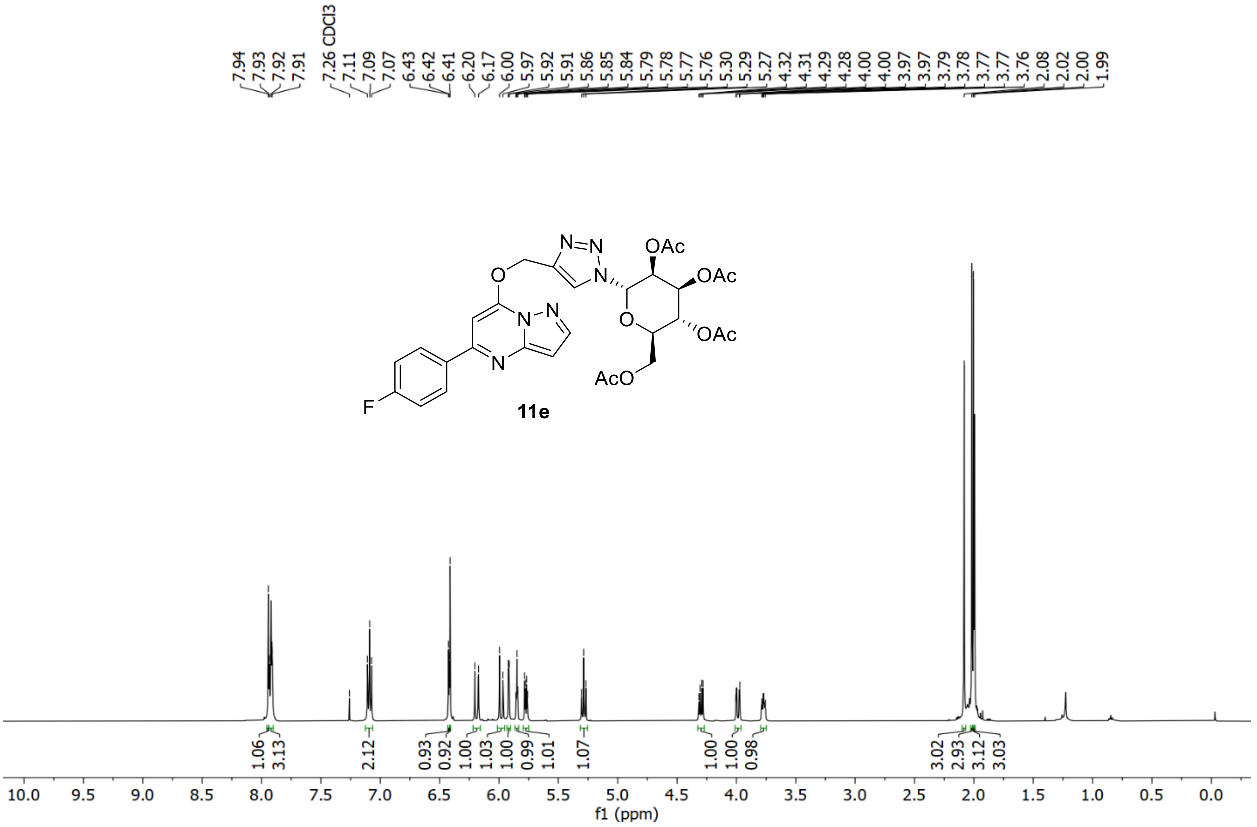

^{13}C NMR (126 MHz, $CDCl_3$)

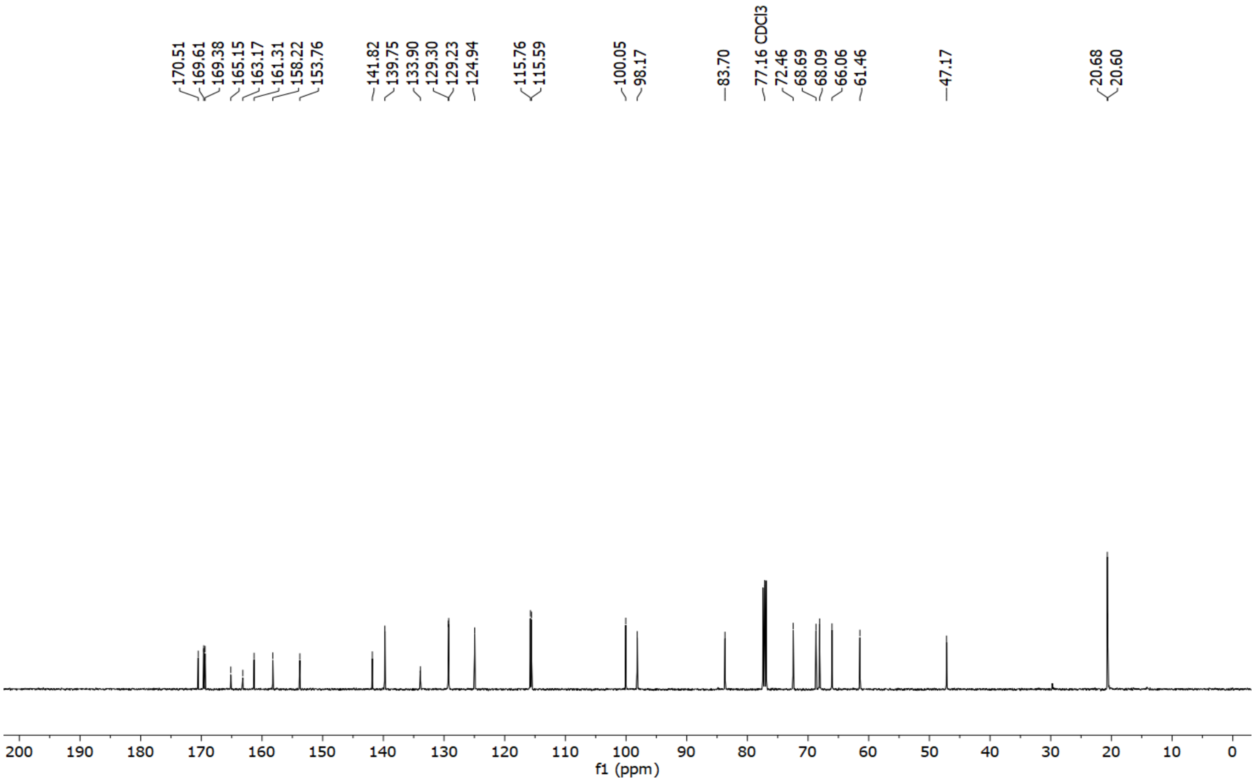

^{1}H-NMR (500 MHz, $CDCl_3$)

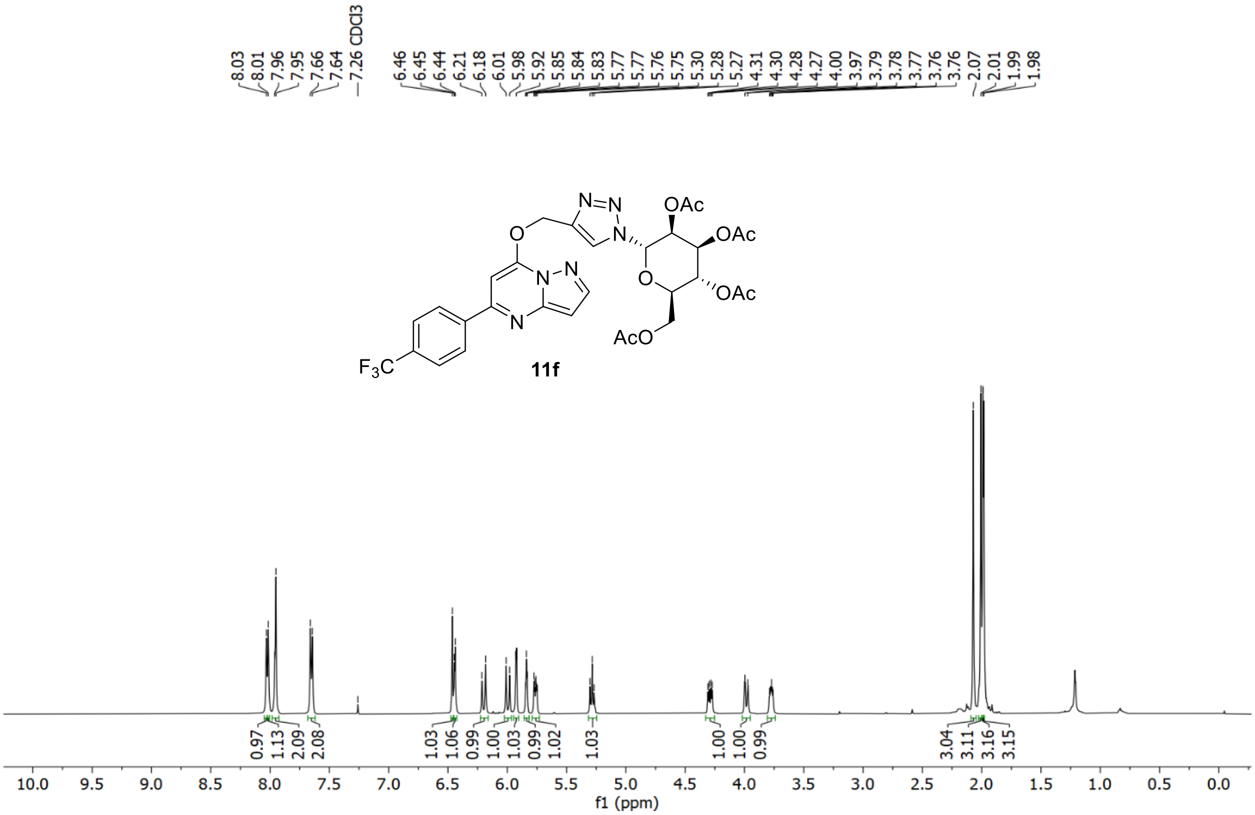

^{13}C NMR (126 MHz, $CDCl_3$)

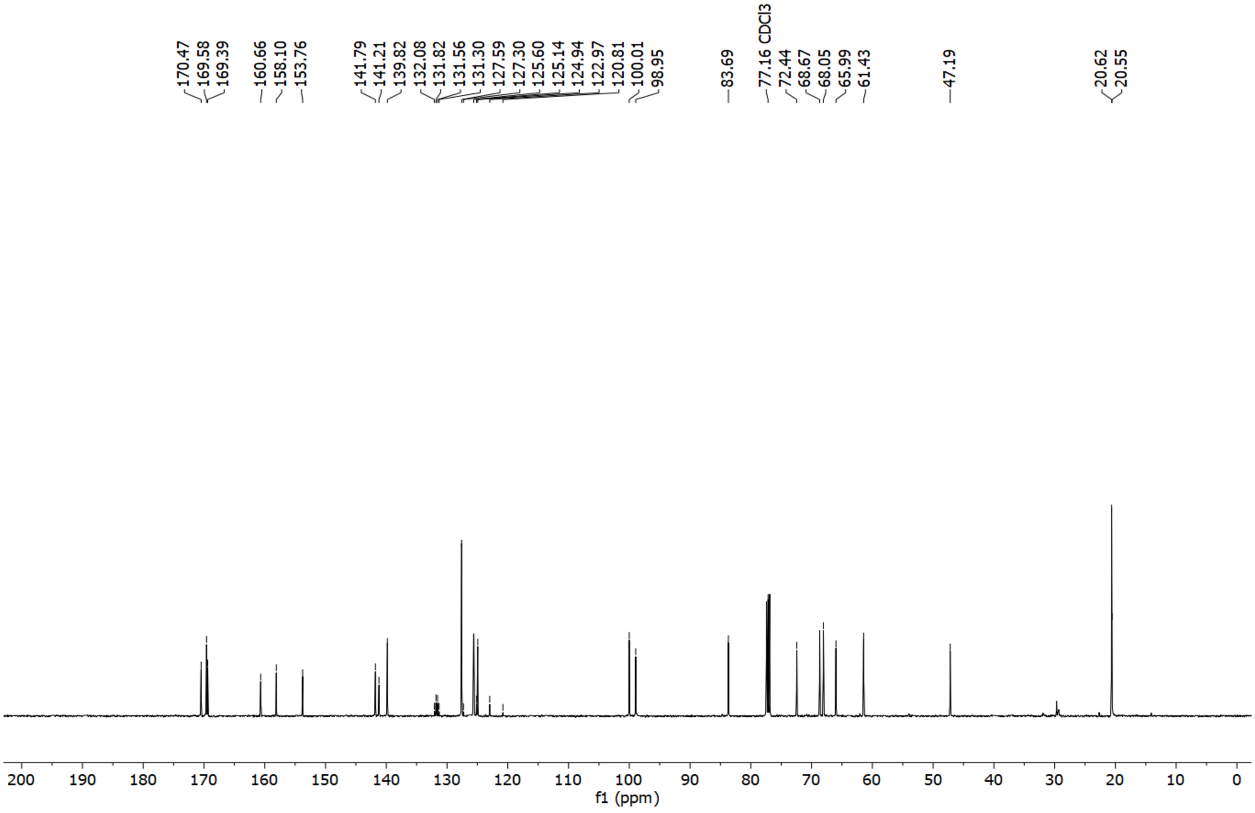

^{1}H-NMR (500 MHz, $CDCl_3$)

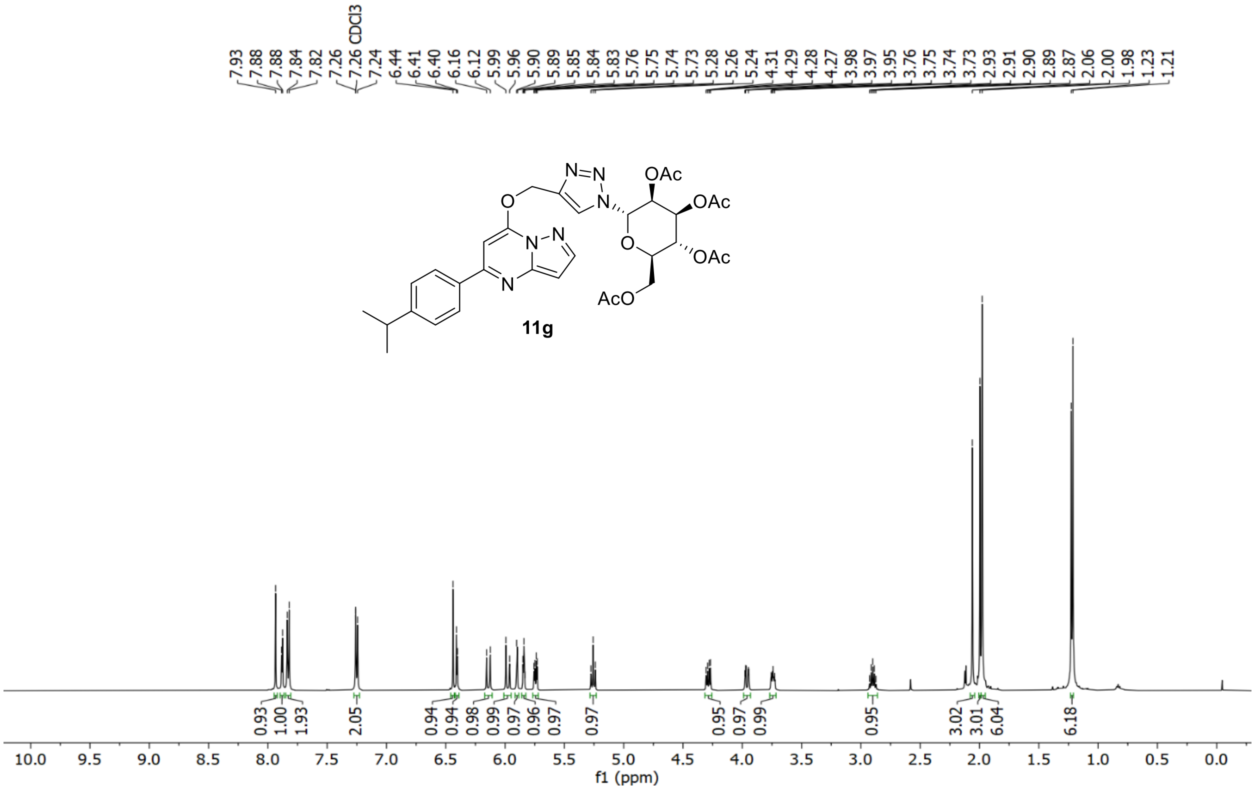

^{13}C NMR (126 MHz, $CDCl_3$)

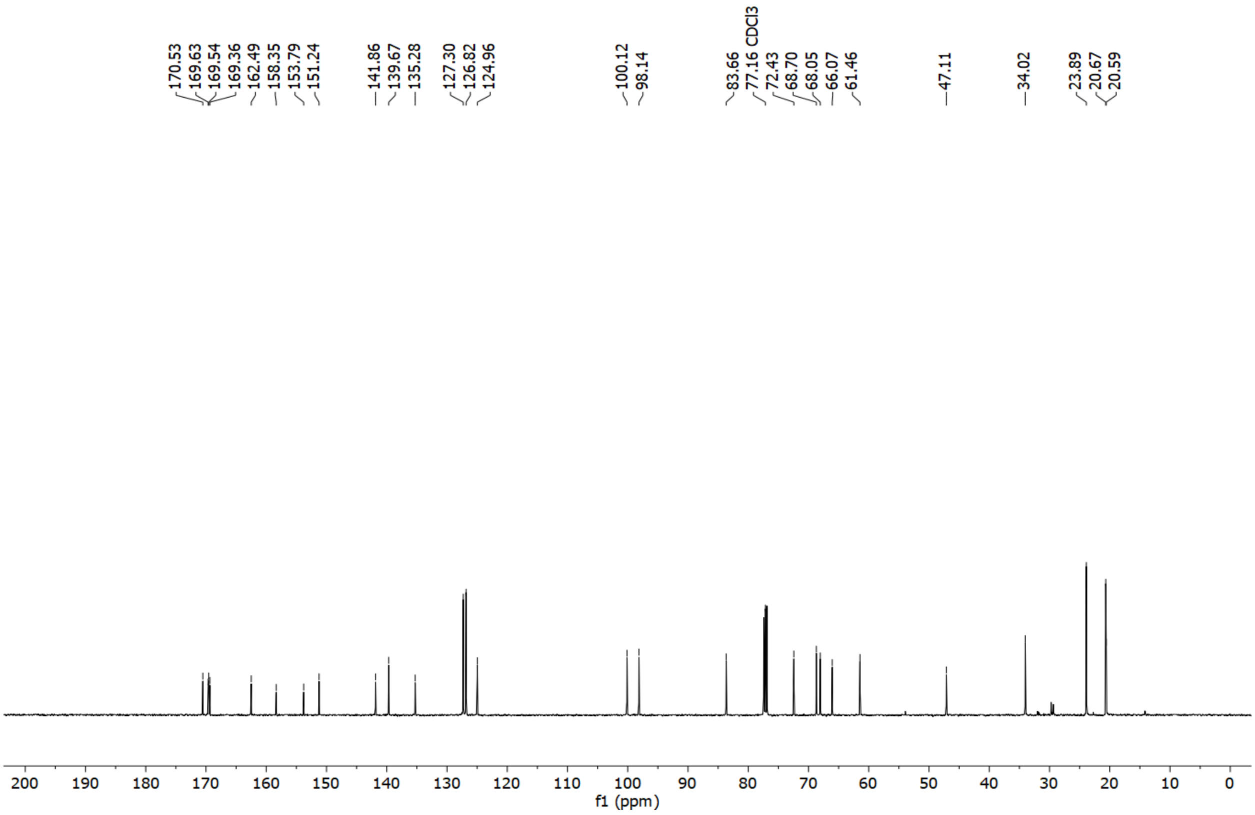

^{1}H-NMR (500 MHz, $CDCl_3$)

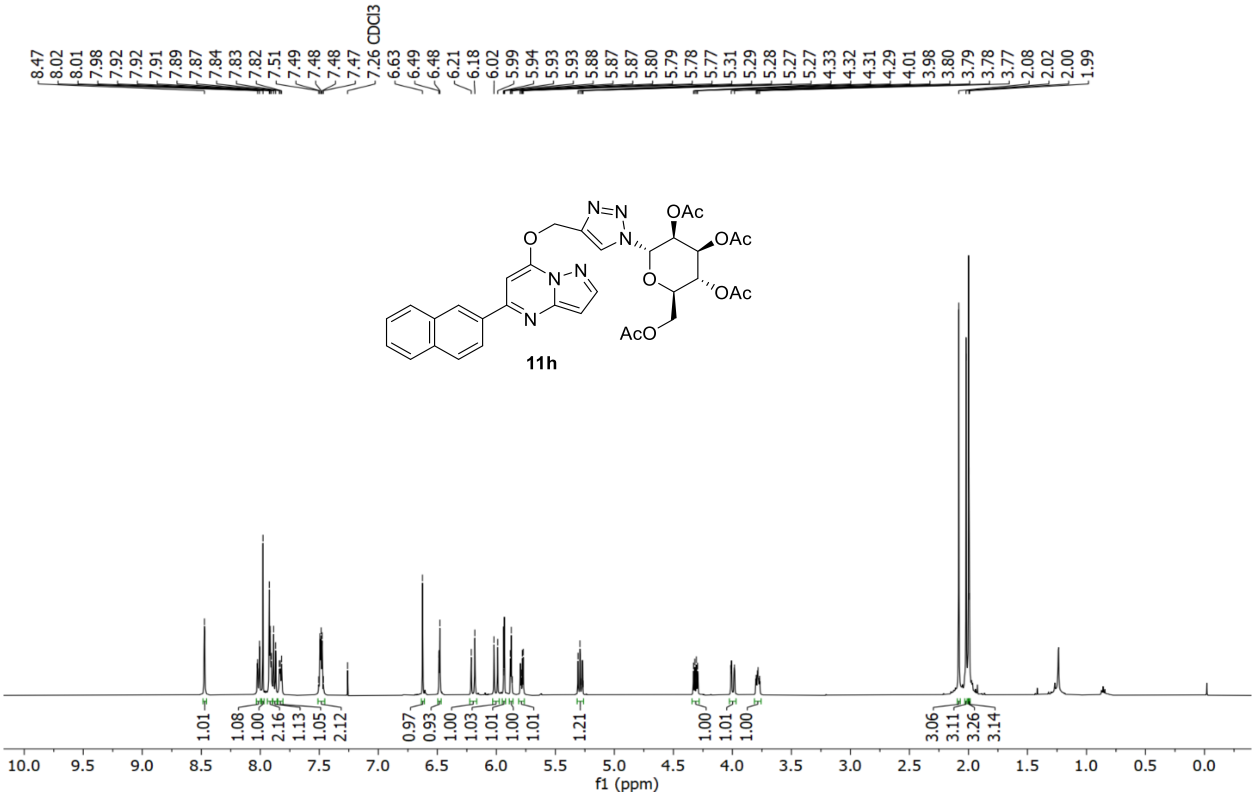

^{13}C NMR (126 MHz, $CDCl_3$)

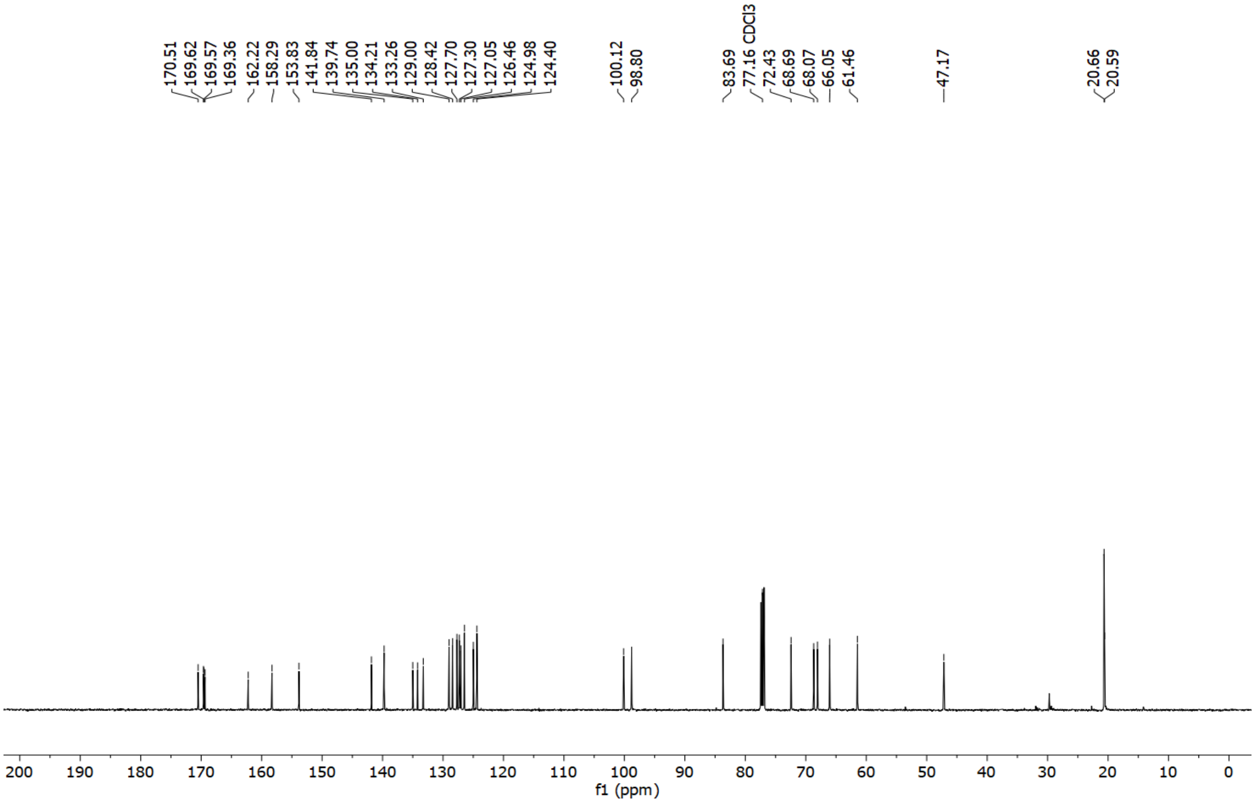

^{1}H-NMR (500 MHz, $CDCl_3$)

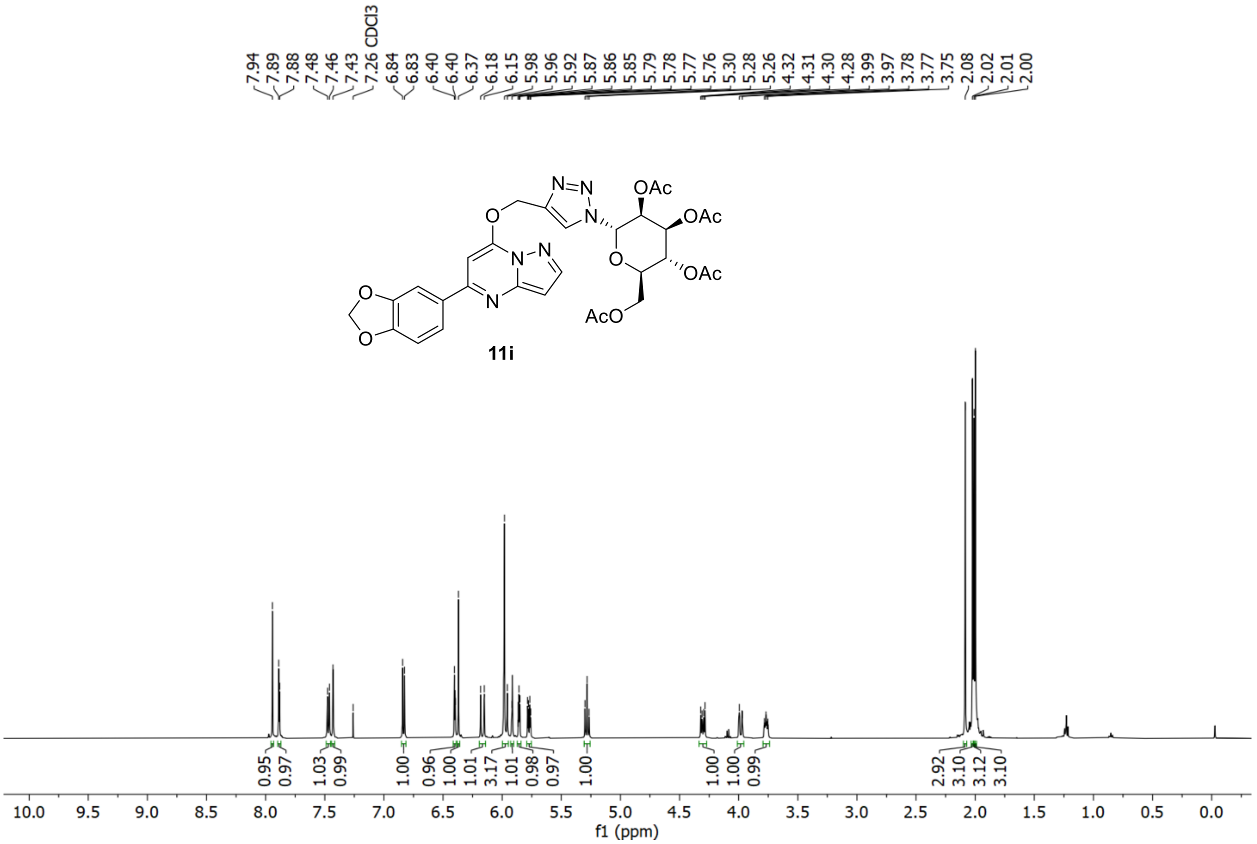

^{13}C NMR (126 MHz, $CDCl_3$)

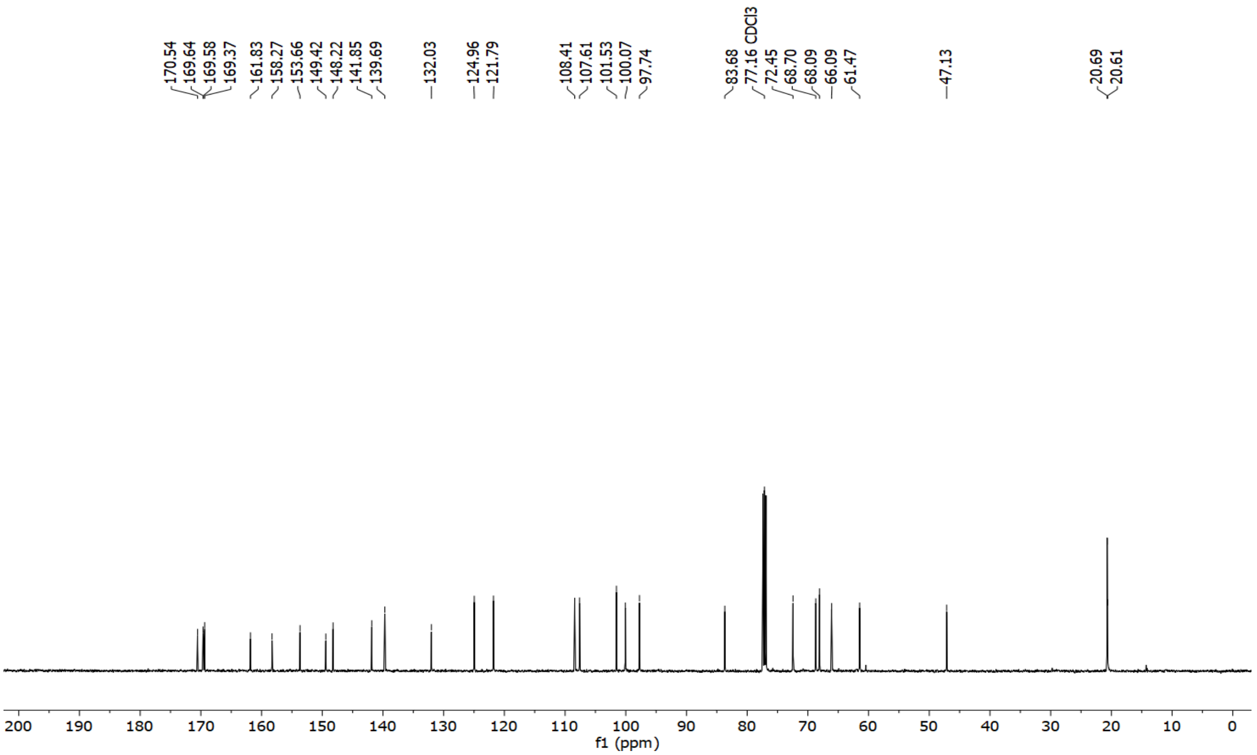

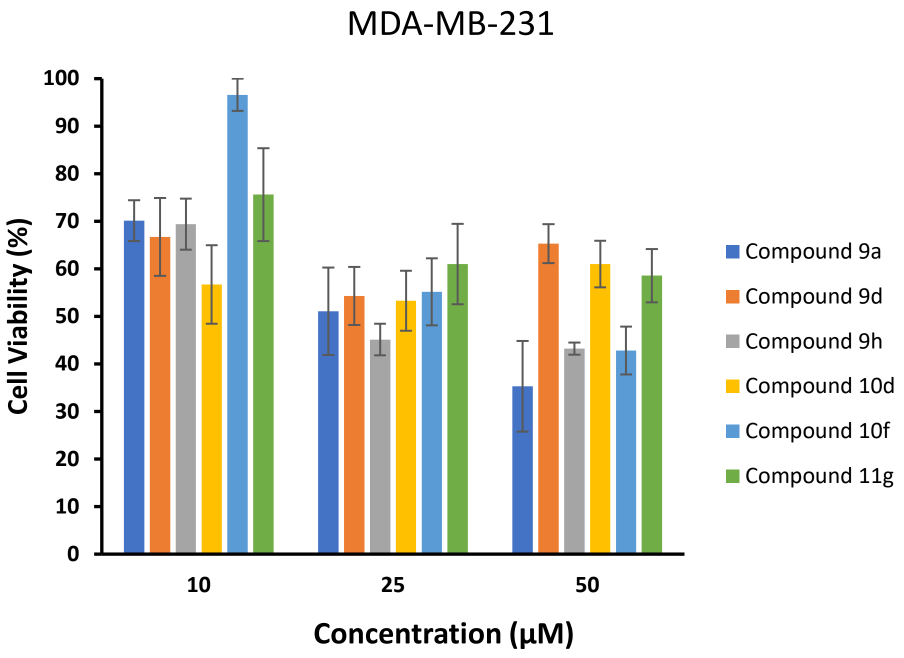

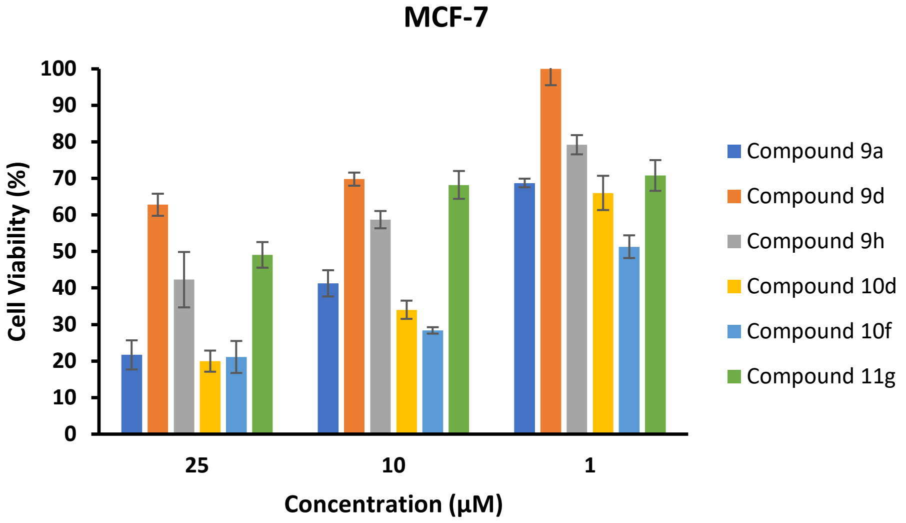

Figure S1. Results of cell viability assays with all the compounds (**9a, 9d, 9h, 10d, 10f and 11g**) in MDA-MB-231 (50 µM, 25 µM, 10 µM concentration) and MCF-7 cancer cell lines (25 µM, 10 µM, 1 µM concentration).

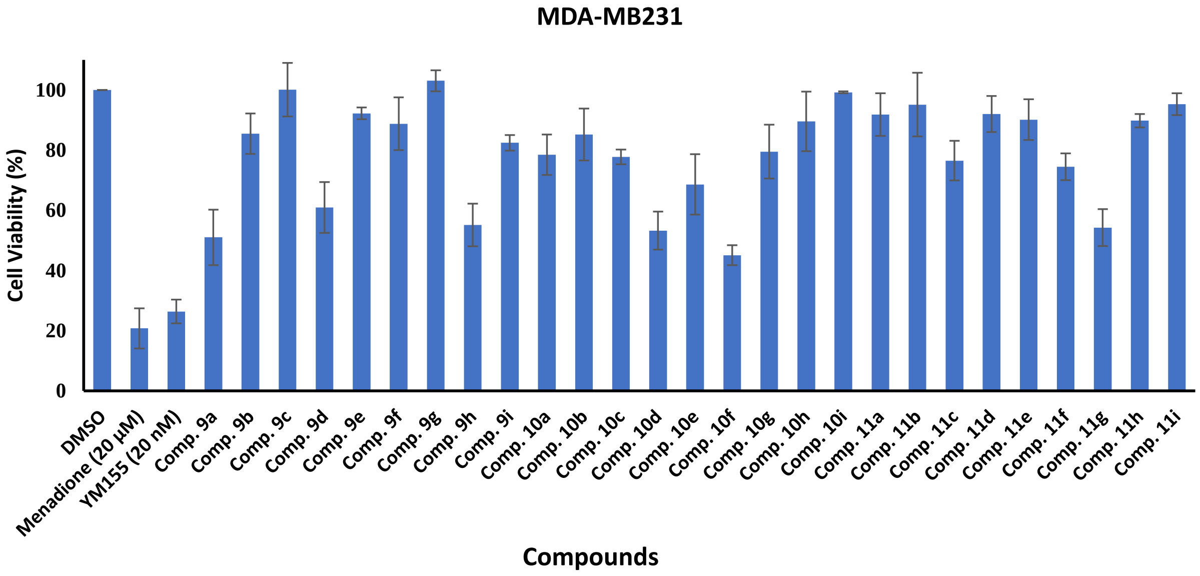

Figure S2. Combined results of cell viability assays with all the compounds (**9a-11i**) in MDA-MB-231 (10 μM concentration).
